# Complex causal association between genetically predicted 731 immunocyte phenotype and osteonecrosis: a bidirectional two-sample Mendelian randomization analysis

**DOI:** 10.1097/JS9.0000000000001327

**Published:** 2024-03-18

**Authors:** Wei Li, Jing-Wen Xu, Jin-Lian Chai, Cong-Cong Guo, Guang-Zheng Li, Mei Gao, Xue-Zhen Liang

**Affiliations:** a College of Traditional Chinese Medicine; b First College of Clinical Medicine; c College of Pharmacy; d Department of Endocrinology and Metabology, The First Affiliated Hospital of Shandong First Medical University & Shandong Provincial Qianfoshan Hospital; e Orthopaedic Microsurgery, Affiliated Hospital of Shandong University of Traditional Chinese Medicine, Shandong; f Department of Cardiology, The First Affiliated Hospital of Shandong First Medical University & Shandong Provincial Qianfoshan Hospital, Shandong Medicine and Health Key Laboratory of Cardiac Electrophysiology and Arrhythmia, Jinan, China

**Keywords:** causally association, immunocyte phenotype, immunocyte, mendelian randomization, osteonecrosis

## Abstract

**Purpose:**

Previous studies have explored the role of immune cells on osteonecrosis. This Mendelian randomization (MR) study further assessed 731 immunocyte phenotypes on osteonecrosis, whether a causal relationship exists, and provides some evidence of causality.

**Methods:**

The 731 immunocyte phenotypes and osteonecrosis data used in this study were obtained from their respective genome-wide association studies (GWAS). The authors used inverse variable weighting (IVW) as the primary analysis method. In addition, the authors simultaneously employed multiple analytical methods, including MR-Egger, weighted mode, simple mode, and weighted median, to strengthen the final results. Finally, sensitivity analyses were conducted to verify the stability and feasibility of the data.

**Results:**

The results of the IVW method of MR analysis showed that 8 immunocyte phenotypes were positively associated with osteonecrosis [*P*<0.05, odds ratio (OR) > 1]; 18 immunocyte phenotypes were negatively associated with osteonecrosis (*P*<0.05, OR<1), none of which were heterogeneous or horizontally pleiotropic (*P* > 0.05) or reverse causality. In addition to this, in reverse MR, osteonecrosis was positively associated with 10 additional immunocyte phenotypes (*P*<0.05, OR > 1) and negatively associated with 14 immunocyte phenotypes (*P*<0.05, OR<1). And none of them had heterogeneity and horizontal pleiotropy (*P* > 0.05) or reverse causality.

**Conclusions:**

The authors demonstrated a complex causal relationship between multiple immune phenotypes and osteonecrosis through a comprehensive two-way, two-sample MR analysis, highlighting the complex pattern of interactions between the immune system and osteonecrosis.

## Introduction

Highlights

- By bidirectional Mendelian randomization, we explored the complex causal associations between 731 immunocyte phenotypes and osteonecrosis. The results indicate that more than 30 immunocyte phenotypes are causally associated with osteonecrosis. This provides options and evidence for the development of osteonecrosis in the immune direction.

Osteonecrosis is a common and intractable disease in orthopaedics, with progressive development, high disability rate, aggravation of family burden, and great social impact, etc^1,2^. It is mainly caused by interruption of bone blood flow, bone ischaemia, and cell necrosis^3^. Its lesions mainly interrupt bone blood flow, bone ischaemia, and cell necrosis, eventually leading to trabecular fracture and femoral head necrosis collapse^4^. With the continuous progress of the lesion, patients may have symptoms such as pain and activity disorder of the affected hip joint, which seriously affects the quality of life, and may eventually face artificial total hip arthroplasty^5,6^. The unknown pathogenesis of osteonecrosis is one of the reasons why it is difficult to diagnose and treat osteonecrosis at an early stage^7^. Immune cells, commonly known as leucocytes, include lymphocytes and phagocytic cells^8^. They also refer specifically to lymphocytes that recognize antigens, produce specific immune responses, etc. It has been found that immune cells affect bone regeneration, osteoclast genesis, osteoblast function regulation, bone density, and other bone-related functions^9–11^.

Osteonecrosis is currently considered to be a multifactorial disease^12^, such as genetic susceptibility^13^, apoptosis of bone cells^14^, abnormal lipid metabolism^15^, osteoporosis^16^, oxidative stress^17^, intraosseous hypertension^18^, thrombosis^19^, and coagulation disorders^20^. In recent years, the influence of the interaction between innate and adaptive immune cells and osteoblasts on the balance of bone metabolism has attracted more and more attention in a variety of bone tissue diseases, which has developed into a new discipline, osteoimmunology^21,22^. Immune cells are the most important regulators of inflammation, and bone immune disorders may be an important cause of osteonecrosis^5,23^. Currently, immune cells have been studied extensively in osteonecrosis of the jaw^24–27^. However, less research has been done on femoral head necrosis. Ma *et al.* ^28^ explored the potential role of immunomodulatory cells in the pathogenesis of femoral head necrosis through a retrospective study of patients with femoral head necrosis versus healthy subjects and found that immunomodulatory cells, such as T and B cells, play an important role in femoral head necrosis, and that the progression of femoral head necrosis may be related to dysregulation of the immune system. Cai *et al.* ^29^ summarized the national and international literature on the immunological correlates of femoral head necrosis, and ultimately also found that there is a chronic inflammatory response and an imbalance between osteoblasts and osteoclasts in the region of necrosis of the femoral head, and that innate immune cells, such as macrophages, neutrophils, and dendritic cells, as well as immune effector cells, such as T-cells and B cells, are intimately associated with the maintenance of bone homoeostasis. In addition, the link between immune cells and osteonecrosis of the femoral head has also been explored through network pharmacology and bioinformatics^30,31^. All of the above suggests that there may be a complex association between immune cells and osteonecrosis. Therefore, in this study, we investigated the complex causal association and reverse causal association between 731 immunocyte phenotypes and 7 types of immune cells on osteonecrosis by MR analysis. This study will provide ideas for future research on the mechanism of osteonecrosis and clinical diagnosis and treatment.

MR is a data analysis technique for assessing aetiological inferences in epidemiological studies, which uses genetic variants with strong correlations with exposure factors as instrumental variables (IVs) to assess causality between exposure factors and outcomes^32,33^. Because the IVs are genetically based, confounding factors do not affect them^34^. Common confounders in this experiment were nutritional status, such as vitamin D and calcium intake; BMI: obesity or low body weight may affect bone health; alcohol consumption: excessive alcohol consumption has been associated with osteonecrosis; smoking status: smoking is a known risk factor for osteonecrosis; chronic diseases: such as diabetes and kidney disease may affect bone health; and history of use of specific medications: such as long-term corticosteroid use^13–20^. This study aimed to investigate the complex causal association between 731 immunocyte phenotypes and osteonecrosis through a MR study.

## Materials and methods

### Study design

**Figure 1:**
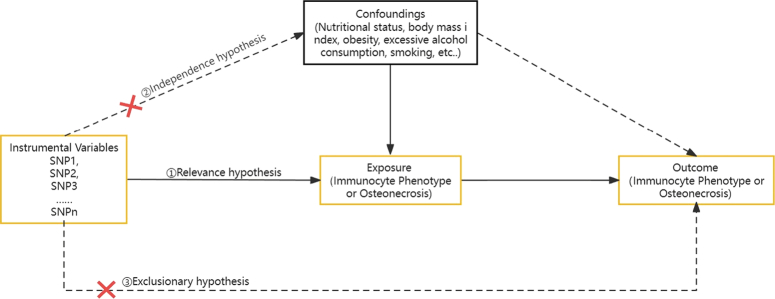
The three major assumptions of Mendelian randomization. ① Relevance hypothesis: instrumental variables (IVs) were strongly associated with exposure factors; ② Independence hypothesis: IVs should not be influenced by known or unknown confounders; and ③ Exclusionary hypothesis: IVs influenced outcome factors only through exposure factors.

We assessed the causal relationship between 731 immunocyte phenotypes (7 groups) and osteonecrosis by a bidirectional two-sample Mendelian randomization analysis. ① Relevance hypothesis: IVs were strongly associated with exposure factors; ② Independence hypothesis: IVs should not be influenced by known or unknown confounders; and ③ exclusionary hypothesis: IVs influenced outcome factors only through exposure factors^34,35^. Figure 1 shows the overall design. The data collected in this study came from Finngen and OPEN GWAS public databases; the data were desensitized before uploading and did not involve personal privacy or identifiable information. This study did not involve information and data and informed consent authorization of the institution, so no ethical review was allowed.

### Exposure and outcome data acquisition

The GWAS catalog (GCST90001391 to GCST90002121) provides an overview of GWAS statistics for every immunological characteristic^36^. It encompasses comprehensive data collected from 3757 Europeans and consisting of 731 immunophenotypes. Supplementary file 1, Supplemental Digital Content 1, http://links.lww.com/JS9/C164 provides specific information on 731 immunocyte phenotypes. We considered sample size, publication year, the number of single-nucleotide polymorphisms (SNPs), and ethnicity before choosing osteonecrosis data from the Finnish database (https://www.finngen.fi/en (accessed 17 October 2023)). A total of 359 399 European participants were included in this dataset, consisting of 1385 cases and 358014 controls.

### Selection of IVs

First, by screening the GWAS data, the inclusion of correlated SNPs satisfied a P 1×10^-5^ threshold^37^. In addition, to prevent linkage disequilibrium (LD) of SNPs from affecting the analysis results, the parameter r threshold was set to 0.001, and the distance of the SNPs was set to 10,000 Kb for the analysis. Secondly, the PhenoScanner V2 database was used to further validate whether the aforementioned included SNP loc and whether there were any other confounding variables linked to the included SNP sites. Finally, to assess whether the included SNPs were affected by weak IVs, the F statistic was used to exclude F values with a value greater than 10 (calculated as F = β^2^ / SE^2^, with β being the allelic effect value and SE being the standard error). If the F statistic of the SNPs was less than 10, it indicated that the SNPs had the possibility of weak instrumental variable bias, and thus, they were excluded to avoid the impact on the results. After that, the result information was extracted through the IEU OpenGWAS database or FinnGen database, and the relationships between SNPs satisfying the hypotheses were obtained from the results. The exposed and resultant datasets were merged, and the palindromic sequences were removed. The last remaining SNPs were the final IVs for the exposure.

### Statistical analysis

The MR analyses in this study were performed in R 4.2.1 software using the TwoSampleMR package. Firstly, the screened IVs were extracted from the ending factors and then analyzed by MR using the TwoSampleMR package. Five commonly used MR analysis methods were used: inverse variance weighted (IVW), weighted median, simple mode, weighted mode, and MR-Egger regression test, with IVW as the main analytical method, supplemented by other analytical methods. The IVW method is characterized by the fact that it does not take into account the presence of an intercept term and uses the inverse of the ending variance (the quadratic of se) as the weight for the fit^34^. A series of sensitivity analyses were conducted to further account for potential pleiotropy. At the end of the MR analysis, the results were subjected to sensitivity analyses such as heterogeneity and horizontal multiple validity tests. Cochran’s Q-test quantified the heterogeneity of the IVs, with *P* less than 0.05 indicating the presence of heterogeneity, and MR-Egger’s method was used as a weighted linear regression with intercepts to assess the presence of horizontal pleiotropy among the IVs. In addition, leave-one-out sensitivity test was used to assess whether the causal effect was significantly influenced by a single SNP. All results are presented as odds ratio (OR) and 95% CI, and results were considered statistically significant when *P* less than 0.05.

## Result

### Forward instrumental variable

In this study, the GWAS data of 731 immunocyte phenotypes were screened for IVs, and all of the IVs had F values greater than 10, and there was no weak instrumental variable bias. Table 1 provides the number of SNPs screened for all positive results.

**Table 1:** Number of SNPs screened in each step.

Immune traits	ID	No. SNPs after LD	No. SNPs after F>10	No. final IVs
CD62L− monocyte %monocyte	GCST90001451	25	25	21
CD11c+ CD62L− monocyte AC	GCST90001452	25	25	22
Resting Treg % CD4 Treg	GCST90001481	31	31	29
Secreting Treg % CD4 Treg	GCST90001493	31	31	29
Activated & resting Treg % CD4 Treg	GCST90001499	29	29	26
CM DN (CD4−CD8−) AC	GCST90001563	4	4	4
T cell %lymphocyte	GCST90001604	18	18	17
CD28− DN (CD4−CD8−) %DN	GCST90001653	28	28	27
CD28+ DN (CD4−CD8−) %DN	GCST90001656	28	28	27
CD45RA− CD28− CD8br %T cell	GCST90001697	190	183	173
CD45RA+ CD28− CD8br AC	GCST90001698	759	753	693
CD19 on IgD+ CD38−	GCST90001726	32	32	30
CD19 on IgD+ CD38− naive	GCST90001727	19	19	19
CD20 on IgD+ CD38−	GCST90001748	27	27	25
CD20 on IgD- CD24−	GCST90001753	29	29	26
CD20 on IgD− CD27−	GCST90001754	19	19	18
IgD on IgD+ CD38dim	GCST90001825	22	22	22
HVEM on CM CD4+	GCST90001876	19	19	16
HVEM on CD8br	GCST90001881	16	16	15
CD28 on secreting Treg	GCST90001887	17	17	15
CD28 on activated & secreting Treg	GCST90001889	26	25	23
CD28 on CD28+ DN (CD4−CD8−)	GCST90001895	3	3	3
CD45 on HLA DR+ CD8br	GCST90001921	22	22	17
CD40 on CD14− CD16+ monocyte	GCST90001989	29	29	26
CX3CR1 on monocyte	GCST90001995	26	26	26
CCR2 on monocyte	GCST90002008	25	25	24

LD, linkage disequilibrium; SNP, single-nucleotide polymorphism.

### Causal effects of immunocyte on osteonecrosis

**Figure 2:**
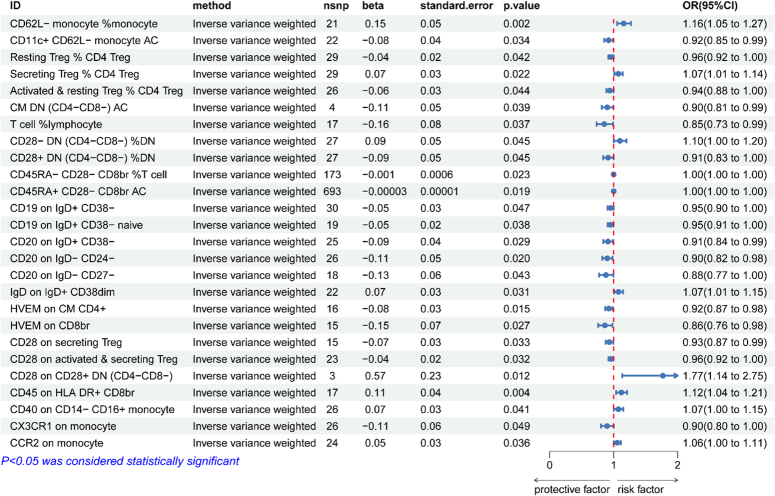
Forward Mendelian randomization analysis of inverse variance weighting method results.

The results of the genetically predicted IVW method for seven groups of immune cells against osteonecrosis are shown in Fig. 2, which indicates that the Trait of the following eight immune cells is positively correlated with the development of osteonecrosis (OR>1, *P*<0.05). cDC Panel: CD62L-monocyte %monocyte; Treg Panel: Secreting Treg % CD4 Treg, CD28−DN (CD4−CD8−) % DN and CD28 on CD28+DN (CD4−CD8−); B cell Panel: IgD on IgD+CD38dim; Monocyte Panel: CD40 on CD14−CD16+monocyte and CCR2 on monocyte; TBNK Panel: CD45 on HLADR+CD8br. Of these, the remaining 18 Traits reduces the incidence of osteonecrosis (OR<1, *P*<0.05). cDC Panel: CD11c+CD62L−monocyte AC; Treg Panel: Resting Treg%CD4 Treg, Activated & resting Treg%CD4 Treg, CD28+DN(CD4−CD8−)%DN,CD45RA-CD28−CD8br%T cell, CD45RA+CD28−CD8br AC, CD28 on secreting Treg and CD28 on activated & secreting Treg; B cell Panel :CD19 on IgD+CD38−, CD19 on IgD+CD38−naïve, CD20 on IgD+CD38−, CD20 on IgD−CD24−and CD20 on IgD−CD27−; Maturation stages of T cell Panel: CM DN (CD4−CD8−) AC, HVEM on CM CD4+and HVEM on CD8br; Monocyte Panel: CX3CR1 on monocyte; TBNK Panel :T cell %lymphocyte. The results of the five methods of MR analysis are provided in Supplementary file 2, Supplemental Digital Content 2, http://links.lww.com/JS9/C165. Supplementary file3, Supplemental Digital Content 3, http://links.lww.com/JS9/C166 provides scatter plots for 26 data items.

### Forward sensitivity analyses

The results of sensitivity analyses showed that none of the above 26 immunocyte phenotypes for MR analysis of osteonecrosis were heterogeneous (*P*>0.05 for Q-test), nor were they horizontally pleiotropic (*P*>0.05 for MR-Egger’s intercept method), which proved that causally robust results were credible (Table 2). The leave-one-out method and funnel plots all indicated reliable data (Supplementary file 3, Supplemental Digital Content 3, http://links.lww.com/JS9/C166).

**Table 2:** Forward MR sensitivity analysis.

		Inverse variance weighted		MR-Egger	
Panel	Immune traits	Q	*P*	Intercept	*P*
cDC	CD62L− monocyte %monocyte	17.81	0.600	0.005	0.850
Treg	Secreting Treg % CD4 Treg	32.24	0.265	0.009	0.575
Treg	CD28−DN (CD4−CD8−) % DN	28.29	0.344	0.015	0.404
B cell	CD28 on CD28+DN (CD4−CD8−)	0.33	0.847	−0.055	0.765
B cell	IgD on IgD+CD38dim	20.34	0.500	0.008	0.655
B cell	CD40 on CD14−CD16+monocyte	31.29	0.179	−0.008	0.705
B cell	CCR2 on monocyte	20.29	0.624	−0.019	0.303
TBNK	CD45 on HLADR+CD8br	4.71	0.997	0.009	0.592
cDC	CD11c+CD62L−monocyte AC	13.54	0.889	−0.006	0.019
Treg	Resting Treg%CD4 Treg	25.49	0.601	-0.038	0.062
Treg	Activated & resting Treg%CD4 Treg	29.27	0.253	−0.006	0.749
Treg	CD28+DN (CD4−CD8−)%DN	28.29	0.344	−0.015	0.404
Treg	CD45RA−CD28−CD8br%T cell	180.63	0.311	0.0005	0.964
Treg	CD45RA+CD28−CD8br AC	733.87	0.131	−0.009	0.167
Treg	CD28 on secreting Treg	15.73	0.330	−0.019	0.352
Treg	CD28 on activated & secreting Treg	19.45	0.617	−0.001	0.936
B cell	CD19 on IgD+CD38−	30.79	0.375	0.021	0.195
B cell	CD19 on IgD+CD38−naïve	17.57	0.484	−0.010	0.508
B cell	CD20 on IgD+CD38−	20.67	0.658	−0.033	0.061
B cell	CD20 on IgD−CD24−	11.80	0.988	−0.012	0.450
B cell	CD20 on IgD−CD27−	16.26	0.505	0.028	0.355
Maturation stages of T cell	CM DN (CD4−CD8−) AC	0.176	0.981	0.025	0.741
Maturation stages of T cell	HVEM on CM CD4+	14.58	0.482	0.019	0.401
Maturation stages of T cell	HVEM on CD8br	23.59	0.051	-0.010	0.814
Monocyte	CX3CR1 on monocyte	32.34	0.148	0.021	0.477
TBNK	T cell %lymphocyte	19.20	0.258	0.029	0.527

MR, Mendelian randomization.

### Reverse instrumental variable

In this study, the GWAS data on osteonecrosis were screened for IVs, and all IVs had F values greater than 10 without weak instrumental variable bias. Table 3 provides the number of SNPs screened by step.

**Table 3:** Number of SNPs screened in each step.

Disease	ID	No. SNPs after LD	No. SNPs after F>10	No. final IVs
Osteonecrosis	finngen_R9_M13_OSTEONECROSIS	20	20	20

IV, instrumental variable; LD, linkage disequilibrium; SNP, single-nucleotide polymorphism.

### Causal effects of osteonecrosis on immunocyte

**Figure 3:**
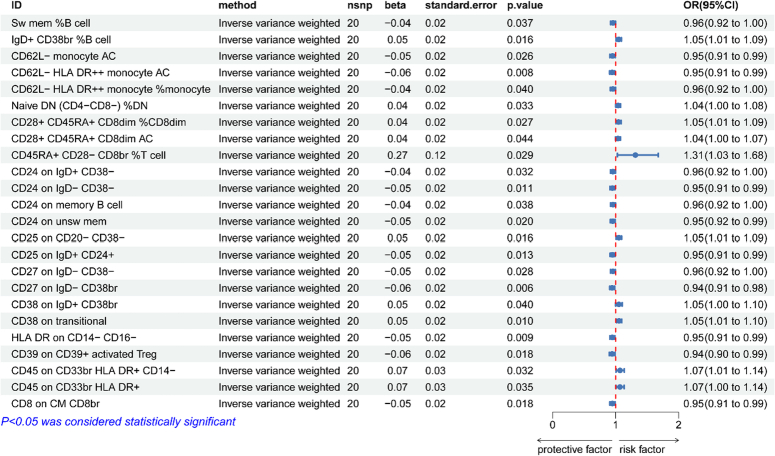
Reverse Mendelian randomization analysis of inverse variance weighting method results.

The results of the genetically predicted IVW method for seven groups of immune cells against osteonecrosis are shown in Fig. 3, which indicates that the Trait of the following ten immune cells is positively correlated with the development of osteonecrosis (OR>1, *P*<0.05). Treg Panel: CD45RA+ CD28−CD8br %T cell, CD28+CD45RA+CD8dim %CD8dim and CD28+CD45RA+CD8dim AC; B Cell Panel: CD38 on transitional, CD38 on IgD+CD38br, CD25 on CD20−CD38− and IgD+ CD38br %B cell; Myeloid Cell: CD45 on CD33br HLA DR+CD14− and CD45 on CD33br HLA DR+; Maturation stages of T Cell Panel: Naive DN (CD4-CD8-) %DN. Of these, the remaining 14 Traits reduces the incidence of osteonecrosis (OR<1, *P*<0.05). cDC Panel: CD62L− HLA DR++ monocyte %monocyte, CD62L− monocyte AC and CD62L−HLA DR++monocyte AC; B Cell Panel: CD24 on memory B cell, Sw mem %B cell, CD24 on IgD+CD38−, CD27 on IgD− CD38−, CD24 on unsw mem, CD25 on IgD+CD24+, CD24 on IgD−CD38− and CD27 on IgD−CD38br; Treg Panel: CD39 on CD39+activated Treg; Monocyte Panel: HLA DR on CD14−CD16−; Maturation stages of T Cell Panel: CD8 on CM CD8br. The results of the five methods of MR analysis are provided in Supplementary file 4, Supplemental Digital Content 4, http://links.lww.com/JS9/C167. Supplementary file 5, Supplemental Digital Content 5, http://links.lww.com/JS9/C168 provides scatter plots for 24 data items

### Reverse sensitivity analyses

The results of sensitivity analyses showed that none of the above 24 immunocyte phenotypes for MR analysis of osteonecrosis were heterogeneous (*P*>0.05 for Q-test), nor were they horizontally pleiotropic (*P*>0.05 for MR-Egger’s intercept method), which proved that causally robust results were credible (Table 4). The leave-one-out method and funnel plots all indicated reliable data (Supplementary file 5, Supplemental Digital Content 5, http://links.lww.com/JS9/C168).

**Table 4:** Reverse MR sensitivity analysis.

		Inverse variance weighted		MR-Egger	
Panel	Immune traits	Q	*P*	Intercept	*P*
Treg	CD45RA+CD28−CD8br %T cell	19.65	0.416	−0.039	0.618
Treg	CD28+CD45RA+CD8dim %CD8dim	15.87	0.666	−0.007	0.596
Treg	CD28+CD45RA+CD8dim AC	18.13	0.514	−0.011	0.350
B cell	CD38 on transitional	15.89	0.665	0.007	0.560
B cell	CD38 on IgD+CD38br	27.24	0.099	−0.0001	0.994
B cell	CD25 on CD20−CD38−	13.67	0.803	0.007	0.550
B cell	IgD+CD38br %B cell	16.61	0.616	0.012	0.332
Myeloid cell	CD45 on CD33br HLA DR+CD14−	12.88	0.845	−0.013	0.496
Myeloid cell	CD45 on CD33br HLA DR+	17.72	0.541	−0.003	0.869
Maturation stages of T cell	Naive DN (CD4−CD8−) %DN	18.39	0.497	0.005	0.690
cDC	CD62L−HLA DR++monocyte %monocyte	11.56	0.903	0.012	0.392
cDC	CD62L−monocyte AC	20.12	0.387	0.012	0.407
cDC	CD62L−HLA DR++monocyte AC	12.67	0.855	0.017	0.207
B cell	CD24 on memory B cell	11.02	0.923	−0.002	0.881
B cell	Sw mem %B cell	12.12	0.881	0.009	0.464
B cell	CD24 on IgD+CD38−	14.33	0.764	0.006	0.621
B cell	CD27 on IgD−CD38−	13.72	0.799	0.003	0.820
B cell	CD24 on unsw mem	10.08	0.951	0.009	0.492
B cell	CD25 on IgD+CD24+	15.99	0.657	0.007	0.562
B cell	CD24 on IgD−CD38−	17.97	0.525	0.012	0.352
B cell	CD27 on IgD−CD38br	16.83	0.601	0.005	0.673
Treg	CD39 on CD39+activated Treg	23.44	0.219	−0.006	0.698
Monocyte	HLA DR on CD14−CD16−	12.48	0.864	−0.009	0.465
Maturation stages of T cell	CD8 on CM CD8br	11.45	0.908	-0.009	0.489

MR, Mendelian randomization.

## Discussion

In this study, we first investigated the causal relationship between 731 immunocyte phenotypes and osteonecrosis using bidirectional MR analysis. We observed some evidence for a causal association between 26 immunocyte phenotypes in cDC, Treg, B cell, TBNK, Maturation stages of T cell, and monocyte cells in forward MR and osteonecrosis; and in reverse MR, Treg, B cell, myeloid cell, maturation stages of T cell, cDC, and monocyte cells. B cell, myeloid cell, maturation stages of T cell, cDC and monocyte cell; and 24 immunocyte phenotypes were causally associated with osteonecrosis in reverse MR. And there was no bidirectional causal association of the same immune cell phenotype.

Our results showed that eight immunocyte phenotypes were positively associated with the risk of developing osteonecrosis. Among them, Cdc cell CD62L− monocyte %monocyte; Treg cell CD28−DN (CD4−CD8−) % DN; B cell CD28 on CD28+DN (CD4−CD8−) and IgD on IgD+CD38dim; TANK cell CD45 on HLADR+CD8br are not yet studied. Treg cells Secreting Treg % CD4 Treg; B cells CD40 on CD14−CD16+monocyte and CCR2 on monocyte have also not been studied directly on osteonecrosis. However, Luo and colleagues demonstrated that CD4+CD25+Foxp3+ Treg cells inhibit osteoclast differentiation and bone resorption by secreting IL-10 and TGF-b1. There is an association between the development of osteonecrosis and osteoclasts and bone resorption, and inhibiting these mechanisms may increase the incidence of osteonecrosis^38^. Mediation analyses by Cao *et al.* ^39^ showed that CD40 on monocytes mediates a variety of immune features that CD40 on CD14-CD16+monocyte is negatively correlated with bone density, and that decreased bone density increases the probability of fracture, which may be related to the reason for our increased risk of osteonecrosis. In addition to this, studies have also shown that CCR2 may be a potential therapeutic target for steroid-induced osteonecrosis of the femoral head^40^.

MR analyses also showed a negative correlation between 18 immunocyte phenotype and osteonecrosis, which may provide ideas for future treatments for osteonecrosis. Rehnberg *et al.* ^41^ explored the effects of anti-CD20 treatment of rheumatoid arthritis and showed that anti-CD20 treatment for rheumatoid arthritis depleted igD+ B cells, suggesting that igD+ B cells may be a risk factor for rheumatoid arthritis, influencing the pathogenesis of rheumatoid arthritis through certain pathways, at the same time, the effect on osteonecrosis has not been studied. It has also been shown that HVEM deficiency induces osteoclast genesis, which increases bone mass and reduces the risk of osteonecrosis^42^. Not only that, it has been found that CX3CL1 plays a role in osteoblast-induced osteoclast differentiation, and the CX3CL1/CX3CR axis may serve as a target for new therapeutic interventions in bone resorption diseases^43^. This is consistent with our MR results. In addition to this, Chen *et al.* ^44^ found that imbalanced T-cell subsets may contribute to the development of osteonecrosis of the femoral head in a study that included 109 patients and that imbalance of T-cell subsets may be involved in the pathophysiological process of osteonecrosis of the femoral head, which is in contrast to our findings.

When we explored the reverse causality of the 731 immunocyte phenotypes on osteonecrosis, we found that the immunocyte phenotypes that were causally associated were not reverse causally associated. That is, certain immunocyte phenotypes that contribute to the development or mitigation of osteonecrosis do not, in turn, influence the development of osteonecrosis.

In this study, bidirectional two-sample MR analyses were performed based on the results of a large cohort of published genomic studies with large sample sizes and high statistical efficiency. In addition, the conclusions of this study are based on exploring the causal relationship between the two at the gene level and using multiple MR analyses for causal inference and validation of the results, so the results of the study are robust and not affected by horizontal pleiotropy and confounding.

However, our study has similar limitations. Firstly, we screened for IVs using a *p* value of *P* less than 1 × 10^-5^, so the IVs were not strong correlated enough, although they do allow for a more comprehensive assessment of the association between immune cell phenotype and osteonecrosis. Second, the study was based on a European database, and it is debatable whether it is applicable to other ethnic groups, which would limit the breadth of our results. Then, we verified heterogeneity and horizontal pleiotropy by the Q-test and the Egger intercept, which, although statistically considered to remove heterogeneity and horizontal pleiotropy, does not yet fully guarantee the absence of heterogeneity and horizontal pleiotropy in the clinical setting. In addition, the two-sample Mendelian randomization analysis method has limitations when dealing with multiple exposures. For instance, it cannot handle the correlation between exposures, which may affect the experiment’s results. Therefore, it is necessary to explore suitable methods for the analysis. Finally, to draw clinical conclusions, we also need to conduct comprehensive clinical trials for validation; therefore, we need a more comprehensive GWAS database and further analytical methods or experimental validation to clarify the association of individual immunocyte phenotypes on osteonecrosis and the mechanism of their influence.

## Conclusion

In conclusion, we demonstrated a causal relationship between multiple immune phenotypes and osteonecrosis through a comprehensive bidirectional two-sample MR analysis, highlighting the complex pattern of interactions between the immune system and osteonecrosis. This provides a new avenue for researchers to explore the biological mechanisms of osteonecrosis and helps to explore early intervention and treatment. Our results extend the findings on immunity and provide valuable clues for the prevention of osteonecrosis.

## Ethics approval and informed consent

All data used in this work are publicly available from studies with relevant participant consent and ethical approval.

## Consent for publication

All participating authors give their consent for this work to be published.

## Source of funding

This work was supported by grants from the National Natural Science Foundation of China (No. 82074453 and No.82205154); the National Natural Science Foundation of Shandong Province (No. ZR2021QH004 and No. ZR2021LZY002); National Natural Science Foundation of Qianfoshan Hospital, Shandong Province (QYPY2020NSFC1012).

## Author contribution

All authors made a significant contribution to the work reported and agreed to be accountable for all aspects of the work. L.W. and L.X.Z. designed the experiments and were responsible for subsequent revisions of the paper. L.W., X.J.W. and C.J.L. performed the experiments and prepared the initial draft of the manuscript. G.C.C., L.G.Z., G.M. and L.X.Z. gave critical feedback during the study or during the manuscript. All authors provided final approval of the version and agreed on the journal for publication.

## Conflicts of interest disclosure

L.W., J.-W.X., J.-L.C., C.-C.G., G.-Z.L., M.G. and X.-Z.L. declare that they have no conflict of interest.

## Research registration unique identifying number (UIN)

This study used publicly available data and therefore ethical approval and informed consent were not required.

## Guarantor

First author Li Wei; Corresponding author Xuezhen Liang.

## Data availability statement

Publicly available datasets were analyzed in this study. These datasets can be found at the following URLs: FinnGen (https://storage.googleapis.com/finngen-public-data-r9/summary_stats/finngen_R9_M13_OSTEONECROSIS.gz) and GWAS Catalog (https://www.ebi.ac.uk/gwas/downloads/summary-statistics).

## Provenance and peer review

Not invited.

## Supplementary Material
