# Supplementary material for: Complex causal association between genetically predicted 731 immunocyte phenotype and osteonecrosis: a bidirectional two-sample Mendelian randomization analysis
Source: Int J Surg. 2024 Mar 18;110(6):3285–93. doi: 10.1097/JS9.0000000000001327 (PMC11175804; doi:10.1097/JS9.0000000000001327)
Supplement: Supplementary file 2 [file js9-110-3285-s002.pdf]

ID	method	nsnp	b	se	pval	OR(95%CI)
CD62L− monocyte %monocyte	MR Egger	21	0.126872392	0.108538766	0.256895597	1.14(0.92 to 1.40)
CD62L− monocyte %monocyte	Weighted median	21	0.077535783	0.071361716	0.277249917	1.08(0.94 to 1.24)
CD62L− monocyte %monocyte	Inverse variance weighted	21	0.145516309	0.047801609	0.002333219	1.16(1.05 to 1.27)
CD62L− monocyte %monocyte	Simple mode	21	0.067647127	0.108059022	0.538381279	1.07(0.87 to 1.32)
CD62L− monocyte %monocyte	Weighted mode	21	0.054031490	0.095406988	0.577472300	1.06(0.88 to 1.27)
CD11c+ CD62L− monocyte AC	MR Egger	22	−0.070804857	0.054818974	0.211219413	0.93(0.84 to 1.04)
CD11c+ CD62L− monocyte AC	Weighted median	22	−0.082578856	0.051396812	0.108121912	0.92(0.83 to 1.02)
CD11c+ CD62L− monocyte AC	Inverse variance weighted	22	−0.083155209	0.039292150	0.034316633	0.92(0.85 to 0.99)
CD11c+ CD62L− monocyte AC	Simple mode	22	−0.100083264	0.078151637	0.214288055	0.90(0.78 to 1.05)
CD11c+ CD62L− monocyte AC	Weighted mode	22	−0.085154513	0.049741956	0.101640641	0.92(0.83 to 1.01)
Resting Treg % CD4 Treg	MR Egger	29	−0.012013518	0.027562625	0.666401477	0.99(0.94 to 1.04)
Resting Treg % CD4 Treg	Weighted median	29	−0.023340972	0.032422866	0.471591309	0.98(0.92 to 1.04)
Resting Treg % CD4 Treg	Inverse variance weighted	29	−0.044577456	0.021930648	0.042087325	0.96(0.92 to 1.00)
Resting Treg % CD4 Treg	Simple mode	29	−0.072636215	0.057347349	0.215733475	0.93(0.83 to 1.04)
Resting Treg % CD4 Treg	Weighted mode	29	−0.040813637	0.025277153	0.117601855	0.96(0.91 to 1.01)
Secreting Treg % CD4 Treg	MR Egger	29	0.056935985	0.039433860	0.160289607	1.06(0.98 to 1.14)
Secreting Treg % CD4 Treg	Weighted median	29	0.051922475	0.038570922	0.178252244	1.05(0.98 to 1.14)
Secreting Treg % CD4 Treg	Inverse variance weighted	29	0.070611622	0.030843383	0.022058415	1.07(1.01 to 1.14)
Secreting Treg % CD4 Treg	Simple mode	29	0.132840938	0.084157962	0.125689637	1.14(0.97 to 1.35)
Secreting Treg % CD4 Treg	Weighted mode	29	0.056525600	0.035043069	0.117953115	1.06(0.99 to 1.13)
Activated & resting Treg % CD4 Treg	MR Egger	26	−0.054977269	0.039998155	0.181987498	0.95(0.88 to 1.02)
Activated & resting Treg % CD4 Treg	Weighted median	26	−0.048562739	0.038241693	0.204123855	0.95(0.88 to 1.03)
Activated & resting Treg % CD4 Treg	Inverse variance weighted	26	−0.062857745	0.031153805	0.043626823	0.94(0.88 to 1.00)
Activated & resting Treg % CD4 Treg	Simple mode	26	−0.087840544	0.087582445	0.325495888	0.92(0.77 to 1.09)
Activated & resting Treg % CD4 Treg	Weighted mode	26	−0.056563732	0.035165431	0.120282035	0.95(0.88 to 1.01)
CM DN (CD4−CD8−) AC	MR Egger	4	−0.128804120	0.079063628	0.244841060	0.88(0.75 to 1.03)
CM DN (CD4−CD8−) AC	Weighted median	4	−0.109554967	0.060736389	0.071266102	0.90(0.80 to 1.01)
CM DN (CD4−CD8−) AC	Inverse variance weighted	4	−0.106033494	0.051293157	0.038714796	0.90(0.81 to 0.99)
CM DN (CD4−CD8−) AC	Simple mode	4	−0.096636048	0.071289254	0.268265681	0.91(0.79 to 1.04)
CM DN (CD4−CD8−) AC	Weighted mode	4	−0.109146950	0.054439223	0.138661904	0.90(0.81 to 1.00)
T cell %lymphocyte	MR Egger	17	−0.308152424	0.241087117	0.220611863	0.73(0.46 to 1.18)
T cell %lymphocyte	Weighted median	17	−0.125859443	0.106467606	0.237150798	0.88(0.72 to 1.09)
T cell %lymphocyte	Inverse variance weighted	17	−0.160512406	0.077059895	0.037255193	0.85(0.73 to 0.99)
T cell %lymphocyte	Simple mode	17	0.022832810	0.189449331	0.905570164	1.02(0.71 to 1.48)
T cell %lymphocyte	Weighted mode	17	−0.056264669	0.132976495	0.677841977	0.95(0.73 to 1.23)
CD28− DN (CD4−CD8−) %DN	MR Egger	27	0.050438874	0.067038543	0.458845000	1.05(0.92 to 1.20)
CD28− DN (CD4−CD8−) %DN	Weighted median	27	0.092560026	0.065316016	0.156450564	1.10(0.97 to 1.25)
CD28− DN (CD4−CD8−) %DN	Inverse variance weighted	27	0.091794078	0.045784394	0.044971530	1.10(1.00 to 1.20)
CD28− DN (CD4−CD8−) %DN	Simple mode	27	0.208169429	0.117179015	0.087353704	1.23(0.98 to 1.55)
CD28− DN (CD4−CD8−) %DN	Weighted mode	27	0.085366058	0.064173995	0.194994324	1.09(0.96 to 1.24)
CD28+ DN (CD4−CD8−) %DN	MR Egger	27	−0.050438874	0.067038543	0.458845000	0.95(0.83 to 1.08)
CD28+ DN (CD4−CD8−) %DN	Weighted median	27	−0.092560026	0.065651550	0.158578638	0.91(0.80 to 1.04)
CD28+ DN (CD4−CD8−) %DN	Inverse variance weighted	27	−0.091794078	0.045784394	0.044971530	0.91(0.83 to 1.00)
CD28+ DN (CD4−CD8−) %DN	Simple mode	27	−0.208169429	0.124715134	0.107084863	0.81(0.64 to 1.04)
CD28+ DN (CD4−CD8−) %DN	Weighted mode	27	−0.085366058	0.067027390	0.214073989	0.92(0.81 to 1.05)
CD45RA− CD28− CD8br %T cell	MR Egger	173	−0.001467573	0.000834034	0.080262530	1.00(1.00 to 1.00)
CD45RA− CD28− CD8br %T cell	Weighted median	173	−0.000987786	0.001069525	0.355707872	1.00(1.00 to 1.00)
CD45RA− CD28− CD8br %T cell	Inverse variance weighted	173	−0.001443538	0.000636150	0.023257563	1.00(1.00 to 1.00)
CD45RA− CD28− CD8br %T cell	Simple mode	173	−0.001578924	0.001849548	0.394469226	1.00(0.99 to 1.00)
CD45RA− CD28− CD8br %T cell	Weighted mode	173	−0.001578924	0.000926993	0.090322786	1.00(1.00 to 1.00)
CD45RA+ CD28− CD8br AC	MR Egger	693	−0.000012500	0.000014300	0.381919416	1.00(1.00 to 1.00)
CD45RA+ CD28− CD8br AC	Weighted median	693	−0.000010700	0.000017300	0.537732190	1.00(1.00 to 1.00)
CD45RA+ CD28− CD8br AC	Inverse variance weighted	693	−0.000025400	0.000010800	0.018961857	1.00(1.00 to 1.00)
CD45RA+ CD28− CD8br AC	Simple mode	693	−0.000064800	0.000046300	0.162135978	1.00(1.00 to 1.00)
CD45RA+ CD28− CD8br AC	Weighted mode	693	0.000011800	0.000034600	0.733552013	1.00(1.00 to 1.00)
CD19 on IgD+ CD38−	MR Egger	30	−0.074884153	0.031072872	0.022772359	0.93(0.87 to 0.99)
CD19 on IgD+ CD38−	Weighted median	30	−0.043608825	0.034527611	0.206584473	0.96(0.89 to 1.02)
CD19 on IgD+ CD38−	Inverse variance weighted	30	−0.051645827	0.026024838	0.047202114	0.95(0.90 to 1.00)
CD19 on IgD+ CD38−	Simple mode	30	−0.053741196	0.071148018	0.456134316	0.95(0.82 to 1.09)
CD19 on IgD+ CD38−	Weighted mode	30	−0.049182798	0.029392543	0.105021559	0.95(0.90 to 1.01)
CD19 on IgD+ CD38− naive	MR Egger	19	−0.039815365	0.028200042	0.176024870	0.96(0.91 to 1.02)
CD19 on IgD+ CD38− naive	Weighted median	19	−0.039644538	0.032382149	0.220849878	0.96(0.90 to 1.02)
CD19 on IgD+ CD38− naive	Inverse variance weighted	19	−0.049731001	0.024004781	0.038292272	0.95(0.91 to 1.00)
CD19 on IgD+ CD38− naive	Simple mode	19	−0.034472261	0.085856034	0.692769801	0.97(0.82 to 1.14)
CD19 on IgD+ CD38− naive	Weighted mode	19	−0.038593661	0.025723926	0.150872819	0.96(0.91 to 1.01)
CD20 on IgD+ CD38−	MR Egger	25	−0.012533381	0.059191572	0.834172910	0.99(0.88 to 1.11)
CD20 on IgD+ CD38−	Weighted median	25	−0.068719961	0.070007589	0.326293346	0.93(0.81 to 1.07)
CD20 on IgD+ CD38−	Inverse variance weighted	25	−0.093427926	0.042704759	0.028686811	0.91(0.84 to 0.99)
CD20 on IgD+ CD38−	Simple mode	25	−0.063089662	0.120820486	0.606333865	0.94(0.74 to 1.19)
CD20 on IgD+ CD38−	Weighted mode	25	−0.058626196	0.059925493	0.337676268	0.94(0.84 to 1.06)
CD20 on IgD− CD24−	MR Egger	26	−0.071830234	0.064189320	0.274199639	0.93(0.82 to 1.06)
CD20 on IgD− CD24−	Weighted median	26	−0.066035444	0.064365606	0.304918443	0.94(0.83 to 1.06)
CD20 on IgD− CD24−	Inverse variance weighted	26	−0.106443281	0.045667322	0.019761729	0.90(0.82 to 0.98)
CD20 on IgD− CD24−	Simple mode	26	−0.150085891	0.102111524	0.154083310	0.86(0.70 to 1.05)
CD20 on IgD− CD24−	Weighted mode	26	−0.080997868	0.058966585	0.181752054	0.92(0.82 to 1.04)
CD20 on IgD− CD27−	MR Egger	18	−0.261340742	0.152149124	0.105147203	0.77(0.57 to 1.04)
CD20 on IgD− CD27−	Weighted median	18	−0.220365728	0.090391449	0.014772673	0.80(0.67 to 0.96)
CD20 on IgD− CD27−	Inverse variance weighted	18	−0.130154232	0.064438917	0.043403320	0.88(0.77 to 1.00)
CD20 on IgD− CD27−	Simple mode	18	−0.243682451	0.141808503	0.103882088	0.78(0.59 to 1.03)
CD20 on IgD− CD27−	Weighted mode	18	−0.223368084	0.122695899	0.086333278	0.80(0.63 to 1.02)
IgD on IgD+ CD38dim	MR Egger	22	0.054290348	0.050225283	0.292587763	1.06(0.96 to 1.17)
IgD on IgD+ CD38dim	Weighted median	22	0.031228851	0.045245348	0.490061307	1.03(0.94 to 1.13)
IgD on IgD+ CD38dim	Inverse variance weighted	22	0.071355756	0.033175433	0.031487147	1.07(1.01 to 1.15)
IgD on IgD+ CD38dim	Simple mode	22	0.053625373	0.077292269	0.495411314	1.06(0.91 to 1.23)
IgD on IgD+ CD38dim	Weighted mode	22	0.032666636	0.040204287	0.425611131	1.03(0.95 to 1.12)
HVEM on CM CD4+	MR Egger	16	−0.108919545	0.047664821	0.038418665	0.90(0.82 to 0.98)
HVEM on CM CD4+	Weighted median	16	−0.065645581	0.047211855	0.164393186	0.94(0.85 to 1.03)
HVEM on CM CD4+	Inverse variance weighted	16	−0.078555884	0.032277768	0.014943481	0.92(0.87 to 0.98)
HVEM on CM CD4+	Simple mode	16	−0.133650950	0.088521394	0.151865977	0.87(0.74 to 1.04)
HVEM on CM CD4+	Weighted mode	16	−0.069278199	0.056948989	0.242592342	0.93(0.83 to 1.04)
HVEM on CD8br	MR Egger	15	−0.112098693	0.164448830	0.507414913	0.89(0.65 to 1.23)
HVEM on CD8br	Weighted median	15	−0.183451377	0.083574539	0.028159080	0.83(0.71 to 0.98)
HVEM on CD8br	Inverse variance weighted	15	−0.147861700	0.066704560	0.026645956	0.86(0.76 to 0.98)
HVEM on CD8br	Simple mode	15	0.076439222	0.187571935	0.689789569	1.08(0.75 to 1.56)
HVEM on CD8br	Weighted mode	15	−0.279397703	0.200167061	0.184511454	0.76(0.51 to 1.12)
CD28 on secreting Treg	MR Egger	15	−0.048767513	0.040145594	0.246059390	0.95(0.88 to 1.03)
CD28 on secreting Treg	Weighted median	15	−0.046569543	0.045684914	0.308030335	0.95(0.87 to 1.04)
CD28 on secreting Treg	Inverse variance weighted	15	−0.070670892	0.033056397	0.032525790	0.93(0.87 to 0.99)
CD28 on secreting Treg	Simple mode	15	−0.145715170	0.080811854	0.092924931	0.86(0.74 to 1.01)
CD28 on secreting Treg	Weighted mode	15	−0.061952317	0.039110112	0.135505053	0.94(0.87 to 1.01)
CD28 on activated & secreting Treg	MR Egger	23	−0.043858442	0.022966317	0.069929313	0.96(0.91 to 1.00)
CD28 on activated & secreting Treg	Weighted median	23	−0.044929176	0.028469991	0.114537132	0.96(0.90 to 1.01)
CD28 on activated & secreting Treg	Inverse variance weighted	23	−0.044640598	0.020842079	0.032205590	0.96(0.92 to 1.00)
CD28 on activated & secreting Treg	Simple mode	23	−0.090780338	0.067266112	0.190878867	0.91(0.80 to 1.04)
CD28 on activated & secreting Treg	Weighted mode	23	−0.047913545	0.023645912	0.055027991	0.95(0.91 to 1.00)
CD28 on CD28+ DN (CD4−CD8−)	MR Egger	3	0.927294188	0.950054054	0.507717626	2.53(0.39 to 16.27)
CD28 on CD28+ DN (CD4−CD8−)	Weighted median	3	0.511919413	0.268672554	0.056732578	1.67(0.99 to 2.83)
CD28 on CD28+ DN (CD4−CD8−)	Inverse variance weighted	3	0.569773531	0.226053816	0.011718104	1.77(1.14 to 2.75)
CD28 on CD28+ DN (CD4−CD8−)	Simple mode	3	0.510881965	0.325007073	0.256586943	1.67(0.88 to 3.15)
CD28 on CD28+ DN (CD4−CD8−)	Weighted mode	3	0.511615606	0.289646802	0.219376831	1.67(0.95 to 2.94)
CD45 on HLA DR+ CD8br	MR Egger	17	0.097241685	0.049323396	0.067400390	1.10(1.00 to 1.21)
CD45 on HLA DR+ CD8br	Weighted median	17	0.090012592	0.057008554	0.114351810	1.09(0.98 to 1.22)
CD45 on HLA DR+ CD8br	Inverse variance weighted	17	0.113488460	0.039359846	0.003934621	1.12(1.04 to 1.21)
CD45 on HLA DR+ CD8br	Simple mode	17	0.155746195	0.087505972	0.094100595	1.17(0.98 to 1.39)
CD45 on HLA DR+ CD8br	Weighted mode	17	0.091605040	0.051657595	0.095209741	1.10(0.99 to 1.21)
CD40 on CD14− CD16+ monocyte	MR Egger	26	0.083577075	0.048436034	0.097290119	1.09(0.99 to 1.20)
CD40 on CD14− CD16+ monocyte	Weighted median	26	0.088710068	0.044158628	0.044548220	1.09(1.00 to 1.19)
CD40 on CD14− CD16+ monocyte	Inverse variance weighted	26	0.070907717	0.034781439	0.041483431	1.07(1.00 to 1.15)
CD40 on CD14− CD16+ monocyte	Simple mode	26	0.063552007	0.082907751	0.450533940	1.07(0.91 to 1.25)
CD40 on CD14− CD16+ monocyte	Weighted mode	26	0.072338623	0.038633745	0.072890418	1.08(1.00 to 1.16)
CX3CR1 on monocyte	MR Egger	26	−0.184967853	0.118461245	0.131515376	0.83(0.66 to 1.05)
CX3CR1 on monocyte	Weighted median	26	−0.085946308	0.075558920	0.255340181	0.92(0.79 to 1.06)
CX3CR1 on monocyte	Inverse variance weighted	26	−0.109573494	0.055694034	0.049134940	0.90(0.80 to 1.00)
CX3CR1 on monocyte	Simple mode	26	−0.111324900	0.142366309	0.441581074	0.89(0.68 to 1.18)
CX3CR1 on monocyte	Weighted mode	26	−0.090312350	0.082365989	0.283320563	0.91(0.78 to 1.07)
CCR2 on monocyte	MR Egger	24	0.071887608	0.030870615	0.029462499	1.07(1.01 to 1.14)
CCR2 on monocyte	Weighted median	24	0.056821391	0.039056234	0.145707752	1.06(0.98 to 1.14)
CCR2 on monocyte	Inverse variance weighted	24	0.054100379	0.025849194	0.036356009	1.06(1.00 to 1.11)
CCR2 on monocyte	Simple mode	24	0.008845987	0.080119505	0.913042070	1.01(0.86 to 1.18)
CCR2 on monocyte	Weighted mode	24	0.053102552	0.031311556	0.103393219	1.05(0.99 to 1.12)

P<0.05 was considered statistically significant

0 1 2

protective factor risk factor
