# Supplementary material for: Complex causal association between genetically predicted 731 immunocyte phenotype and osteonecrosis: a bidirectional two-sample Mendelian randomization analysis
Source: Int J Surg. 2024 Mar 18;110(6):3285–93. doi: 10.1097/JS9.0000000000001327 (PMC11175804; doi:10.1097/JS9.0000000000001327)
Supplement: Supplementary file 3 [file js9-110-3285-s003.pdf]

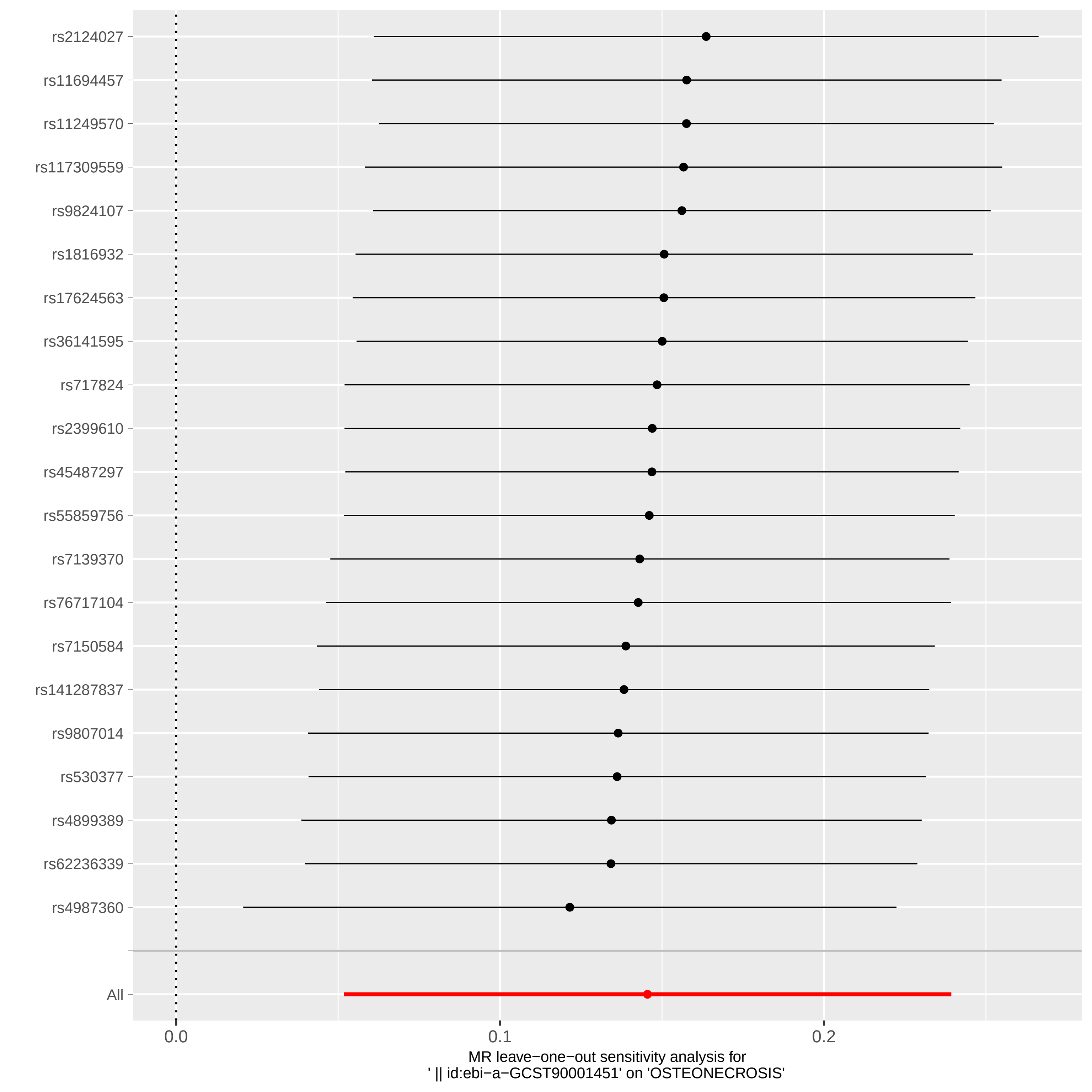
rs2124027
rs11694457
rs11249570
rs117309559
rs9824107
rs1816932
rs17624563
rs36141595
rs717824
rs2399610
rs45487297
rs55859756
rs7139370
rs76717104
rs7150584
rs141287837
rs9807014
rs530377
rs4899389
rs62236339
rs4987360
All
0.0
0.1
0.2
MR leave−one−out sensitivity analysis for
' || id:ebi−a−GCST90001451' on 'OSTEONECROSIS'

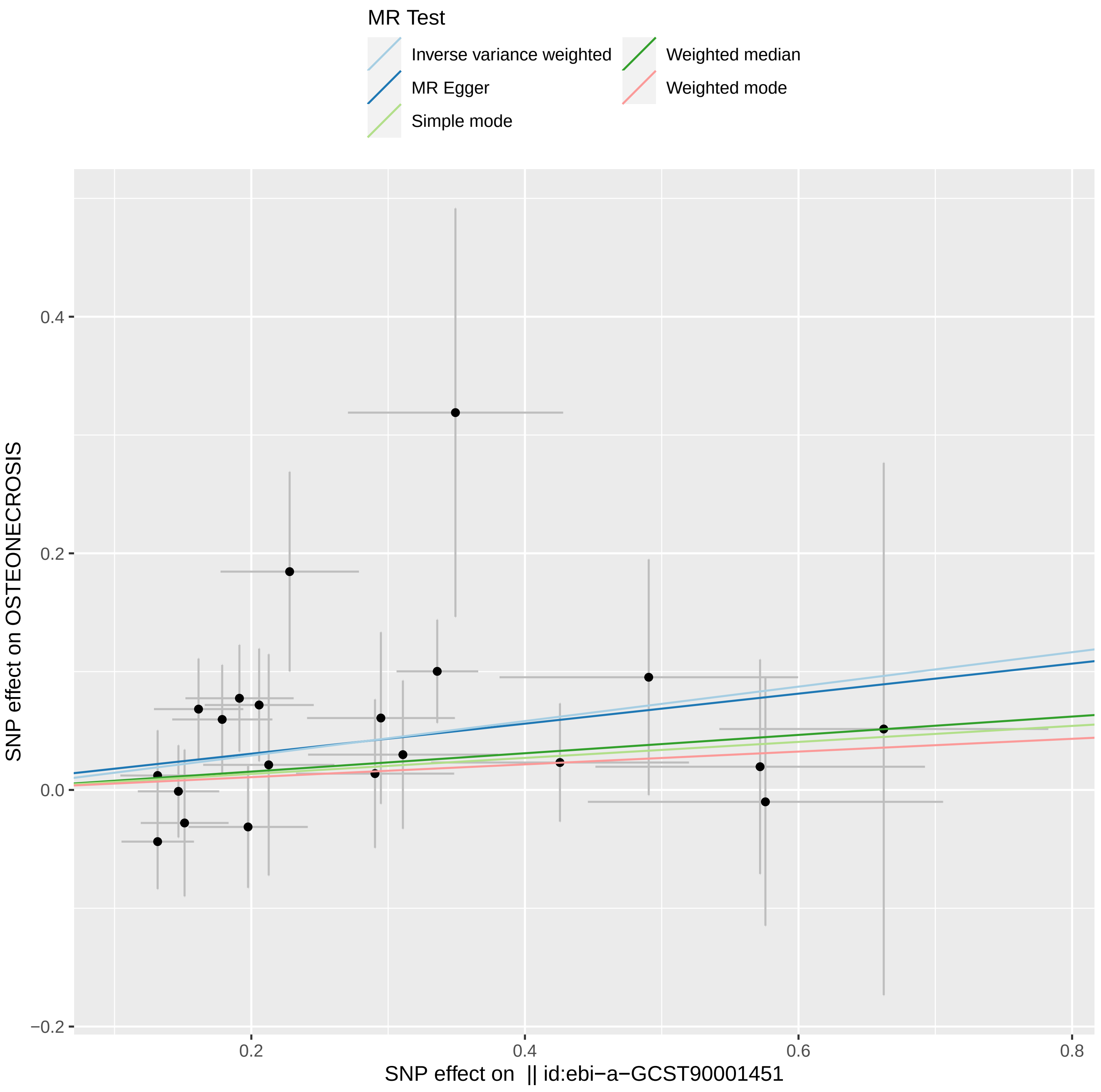

MR Test
Inverse variance weighted
MR Egger
Simple mode
Weighted median
Weighted mode
0.4
0.2
0.0
−0.2
SNP effect on OSTEONECROSIS
0.2
0.4
0.6
0.8
SNP effect on || id:ebi−a−GCST90001451

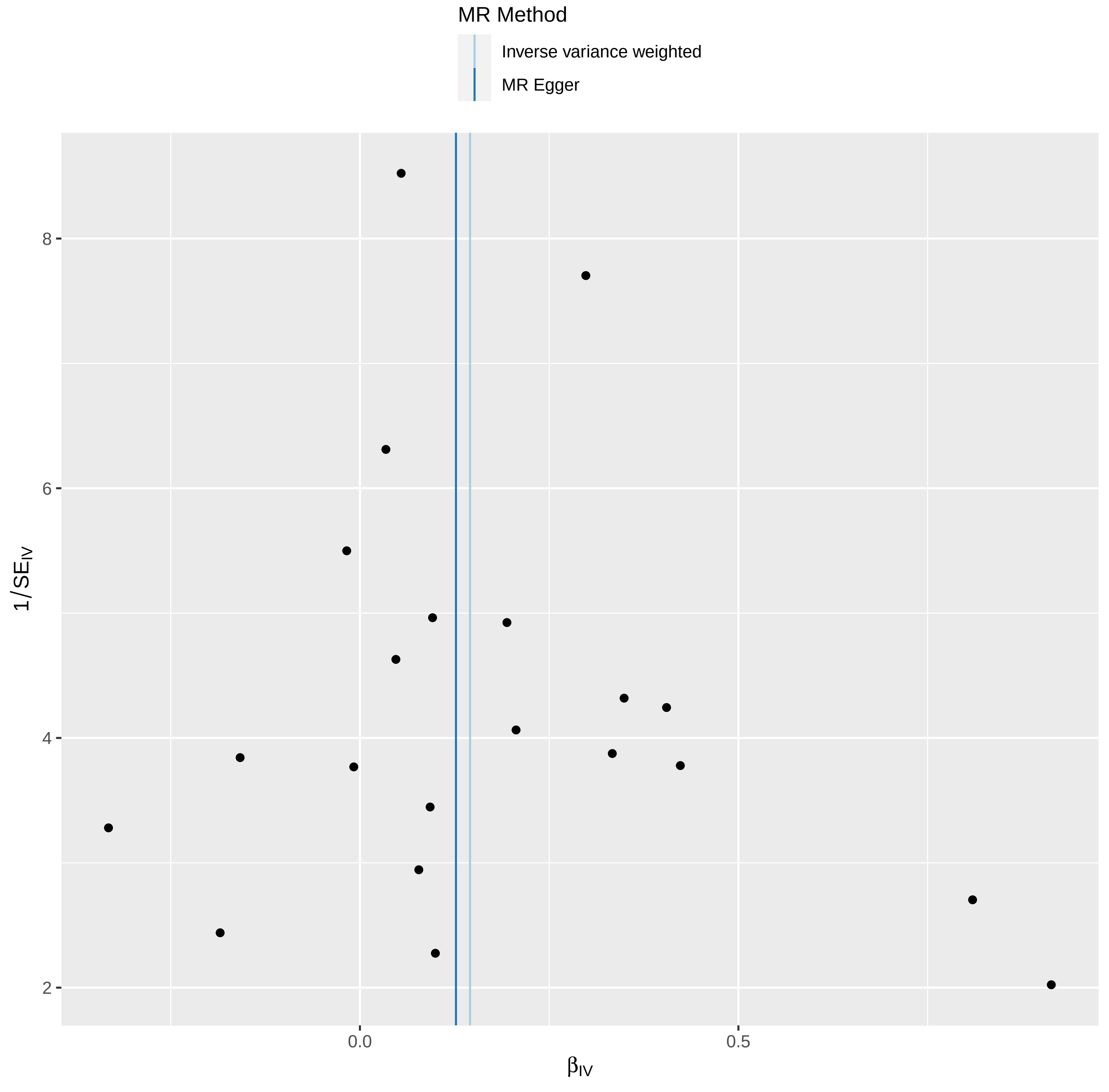

MR Method
Inverse variance weighted
MR Egger
8
6
4
2
1/SE_IV
0.0
0.5
β_IV

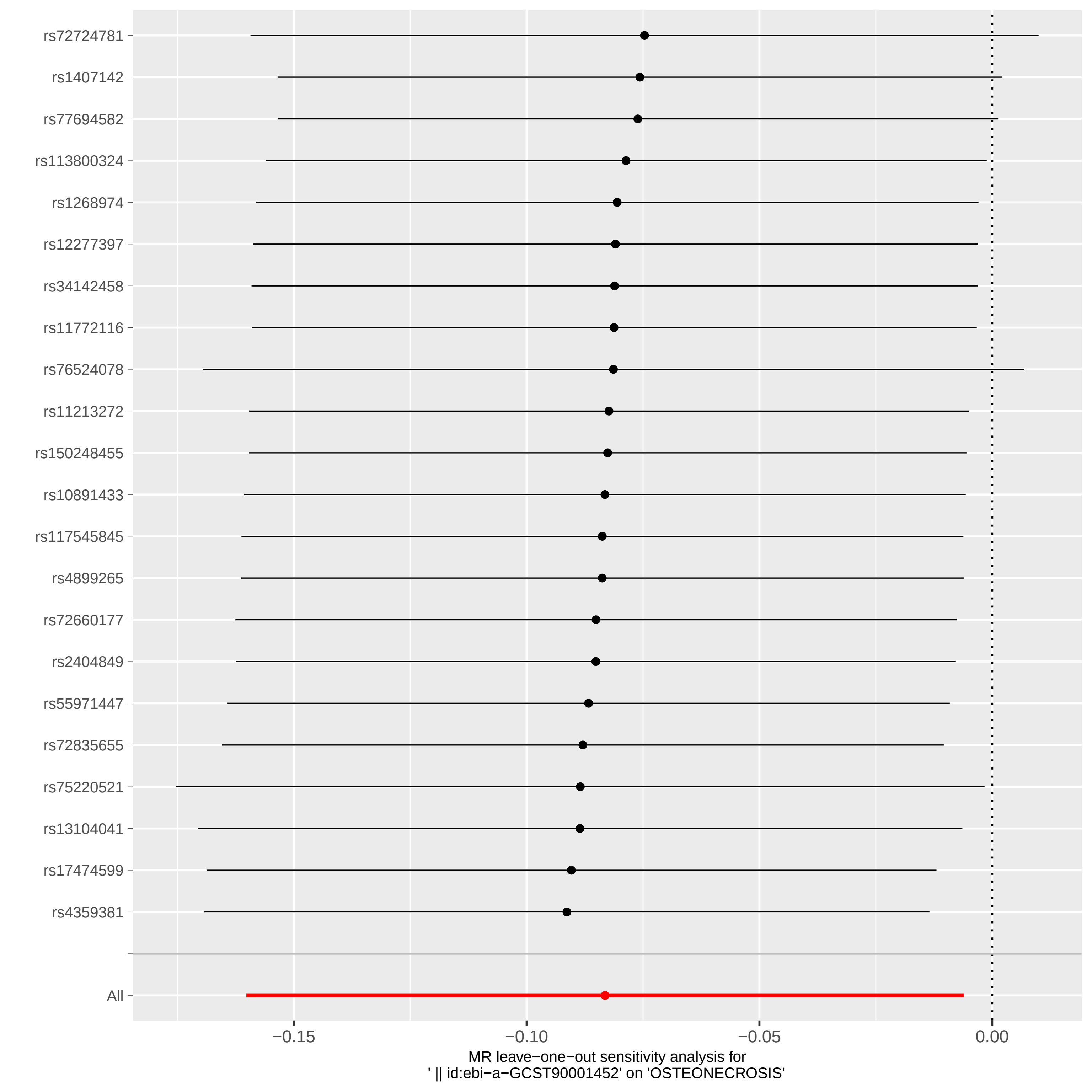

rs72724781
rs1407142
rs77694582
rs113800324
rs1268974
rs12277397
rs34142458
rs11772116
rs76524078
rs11213272
rs150248455
rs10891433
rs117545845
rs4899265
rs72660177
rs2404849
rs55971447
rs72835655
rs75220521
rs13104041
rs17474599
rs4359381
All
−0.15
−0.10
−0.05
0.00
MR leave−one−out sensitivity analysis for
' || id:ebi−a−GCST90001452' on 'OSTEONECROSIS'

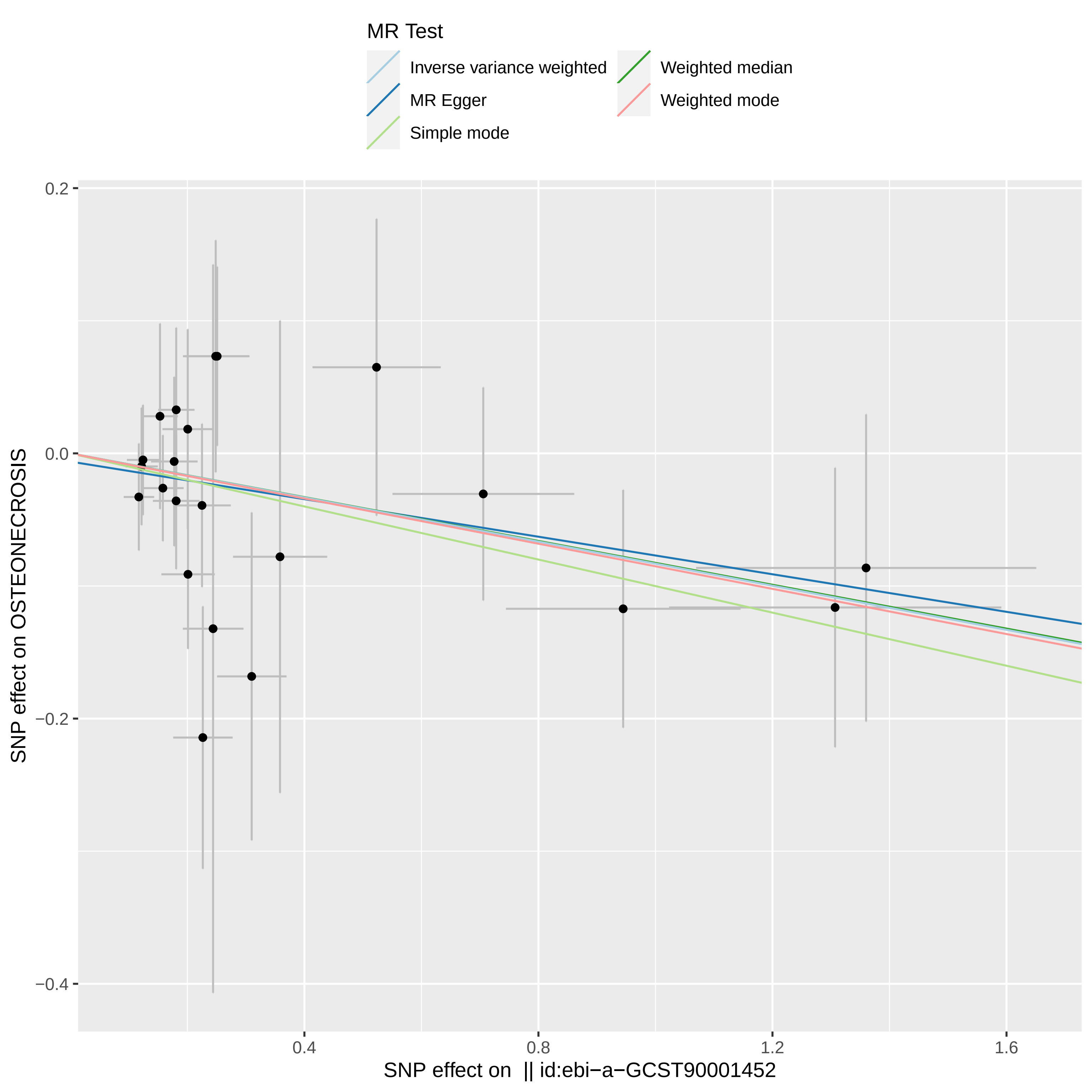

MR Test
Inverse variance weighted
MR Egger
Simple mode
Weighted median
Weighted mode
0.2
0.0
−0.2
−0.4
SNP effect on OSTEONECROSIS
0.4
0.8
1.2
1.6
SNP effect on || id:ebi−a−GCST90001452

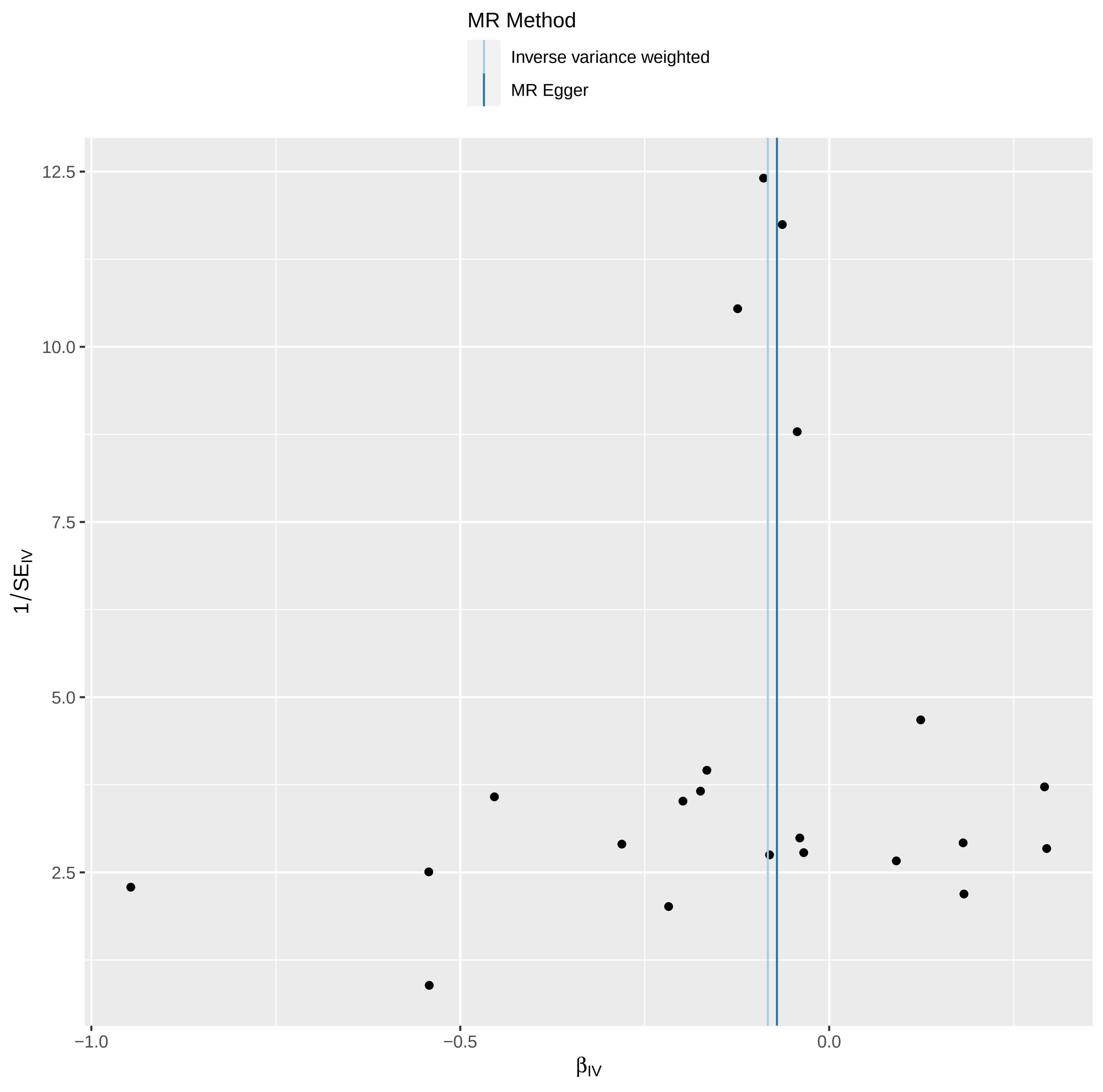

MR Method
Inverse variance weighted
MR Egger
12.5
10.0
7.5
5.0
2.5
1/SE_IV
−1.0
−0.5
0.0
β_IV

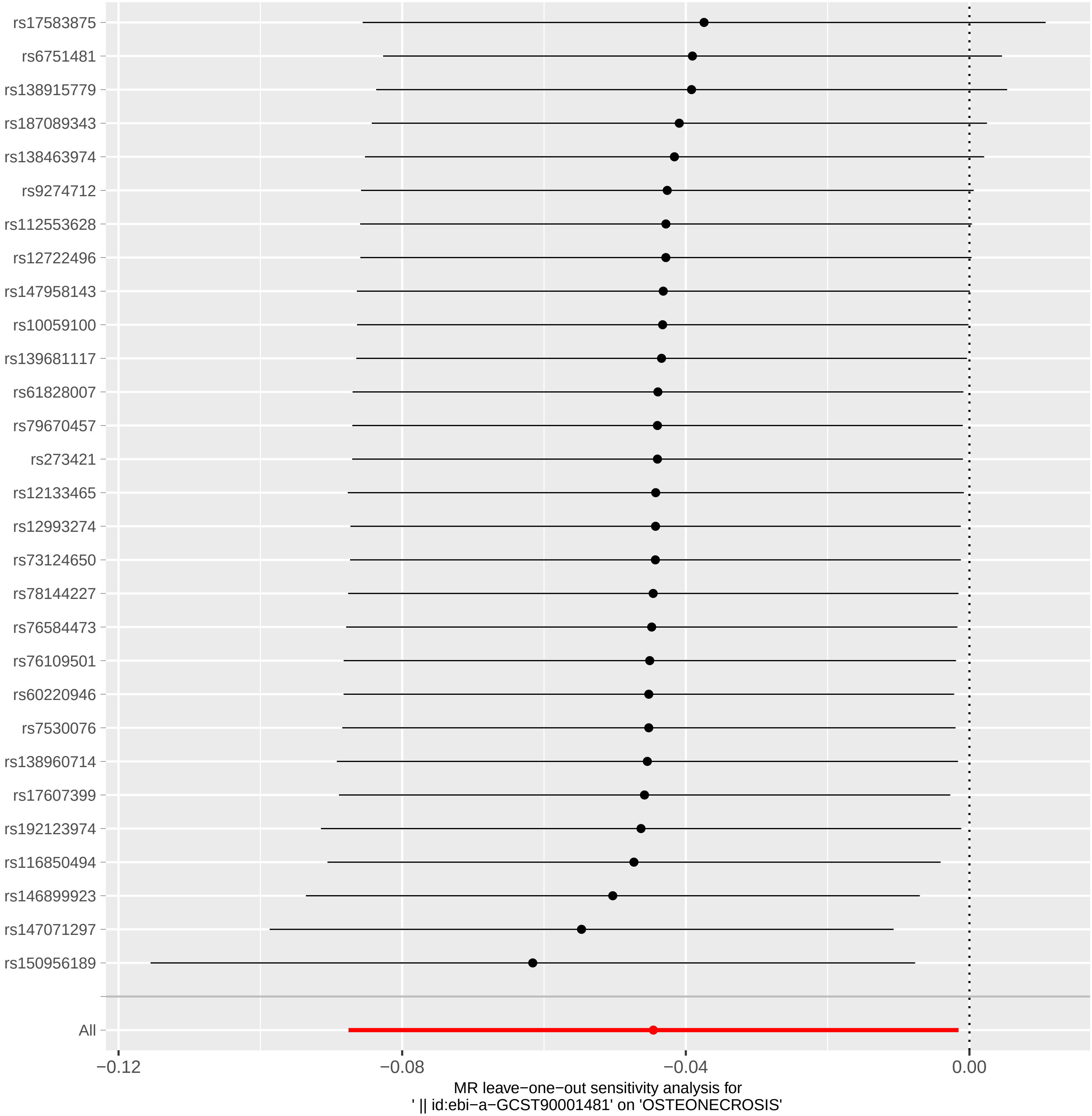

rs17583875
rs6751481
rs138915779
rs187089343
rs138463974
rs9274712
rs112553628
rs12722496
rs147958143
rs10059100
rs139681117
rs61828007
rs79670457
rs273421
rs12133465
rs12993274
rs73124650
rs78144227
rs76584473
rs76109501
rs60220946
rs7530076
rs138960714
rs17607399
rs192123974
rs116850494
rs146899923
rs147071297
rs150956189
All
−0.12
−0.08
−0.04
0.00
MR leave−one−out sensitivity analysis for
' || id:ebi−a−GCST90001481' on 'OSTEONECROSIS'

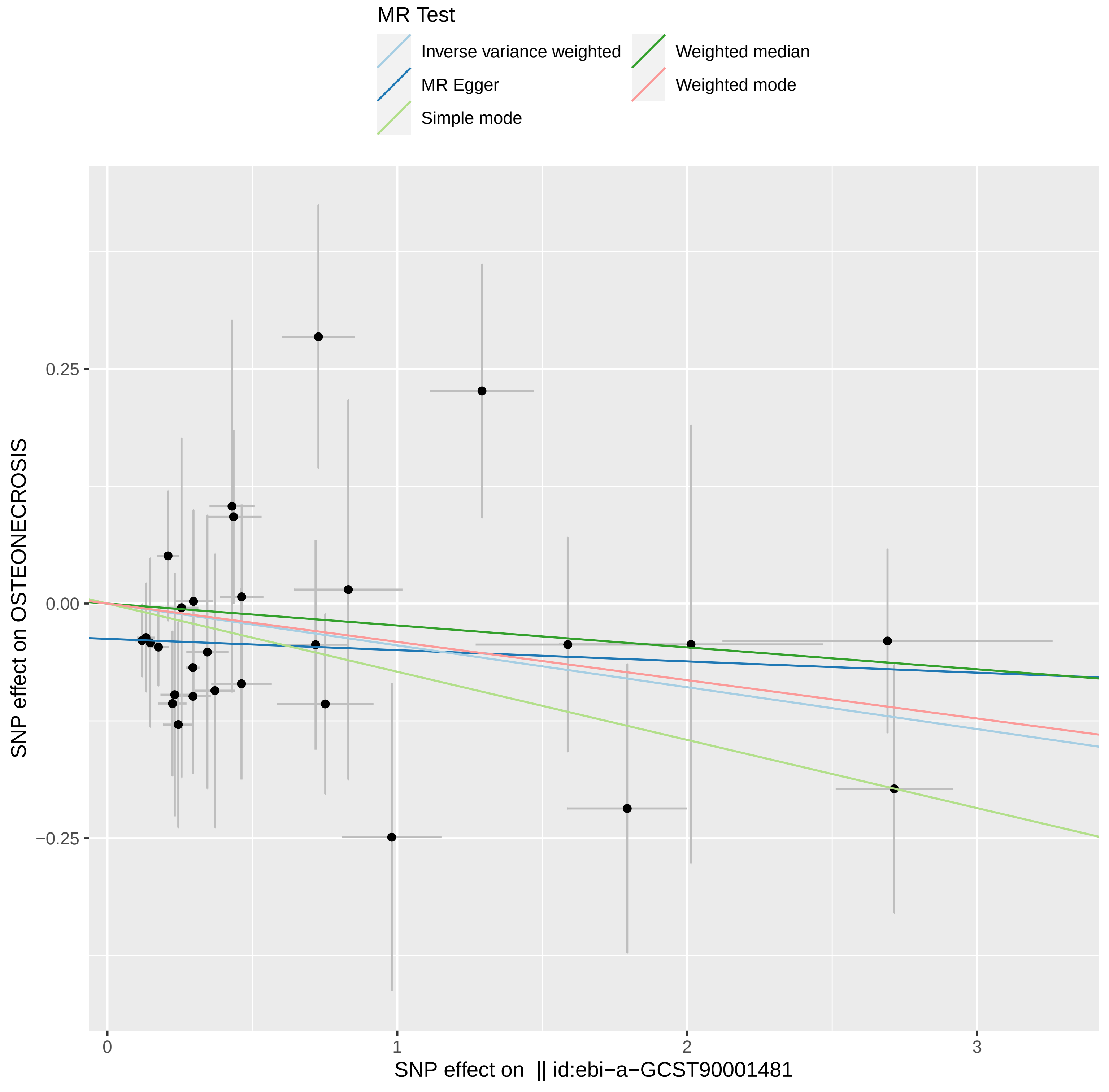
MR Test
Inverse variance weighted
MR Egger
Simple mode
Weighted median
Weighted mode
SNP effect on OSTEONECROSIS
0.25
0.00
−0.25
0
1
2
3
SNP effect on || id:ebi−a−GCST90001481

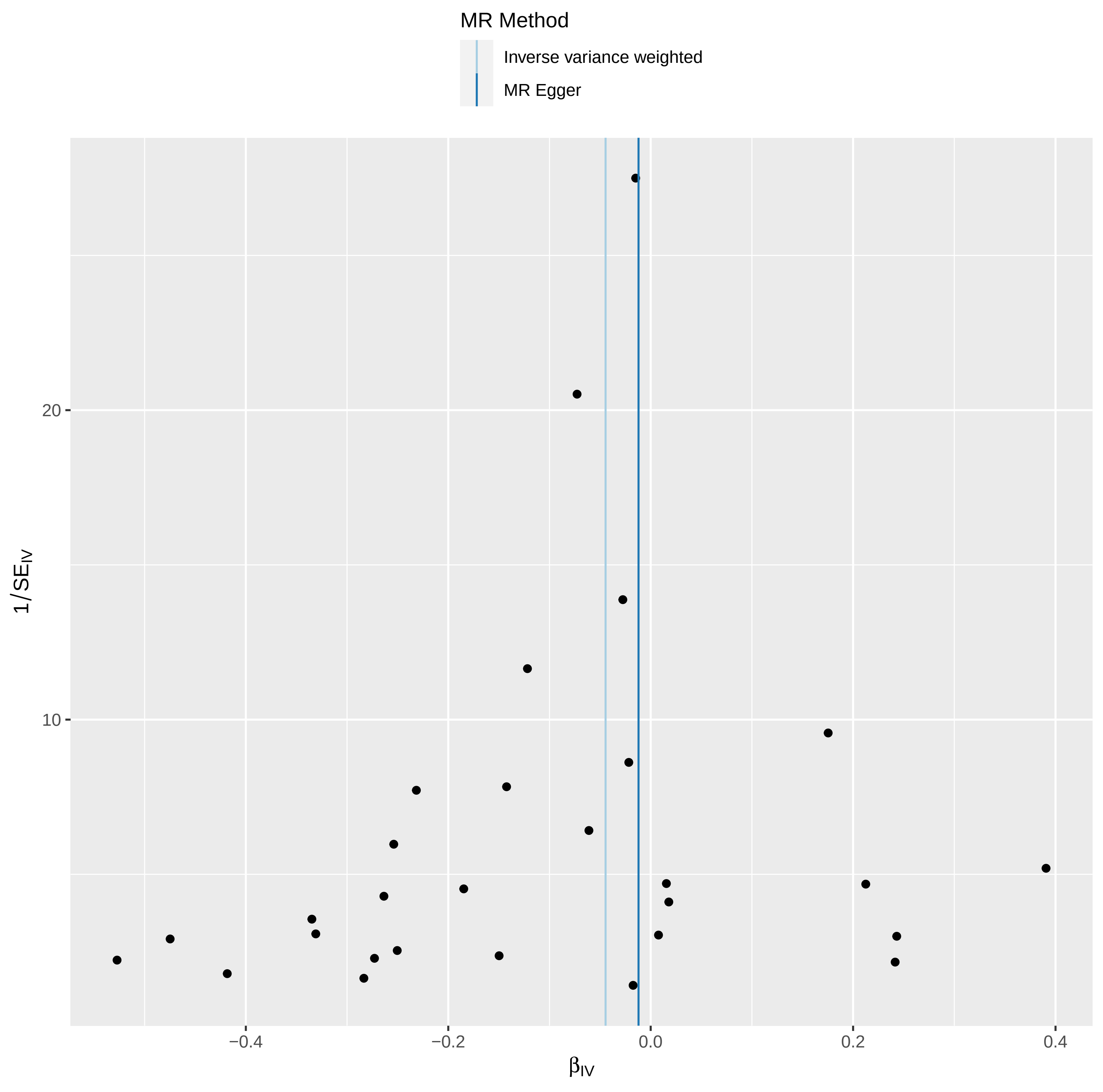
MR Method
Inverse variance weighted
MR Egger
20
10
1/SE$_{IV}$
−0.4
−0.2
0.0
0.2
0.4
β$_{IV}$

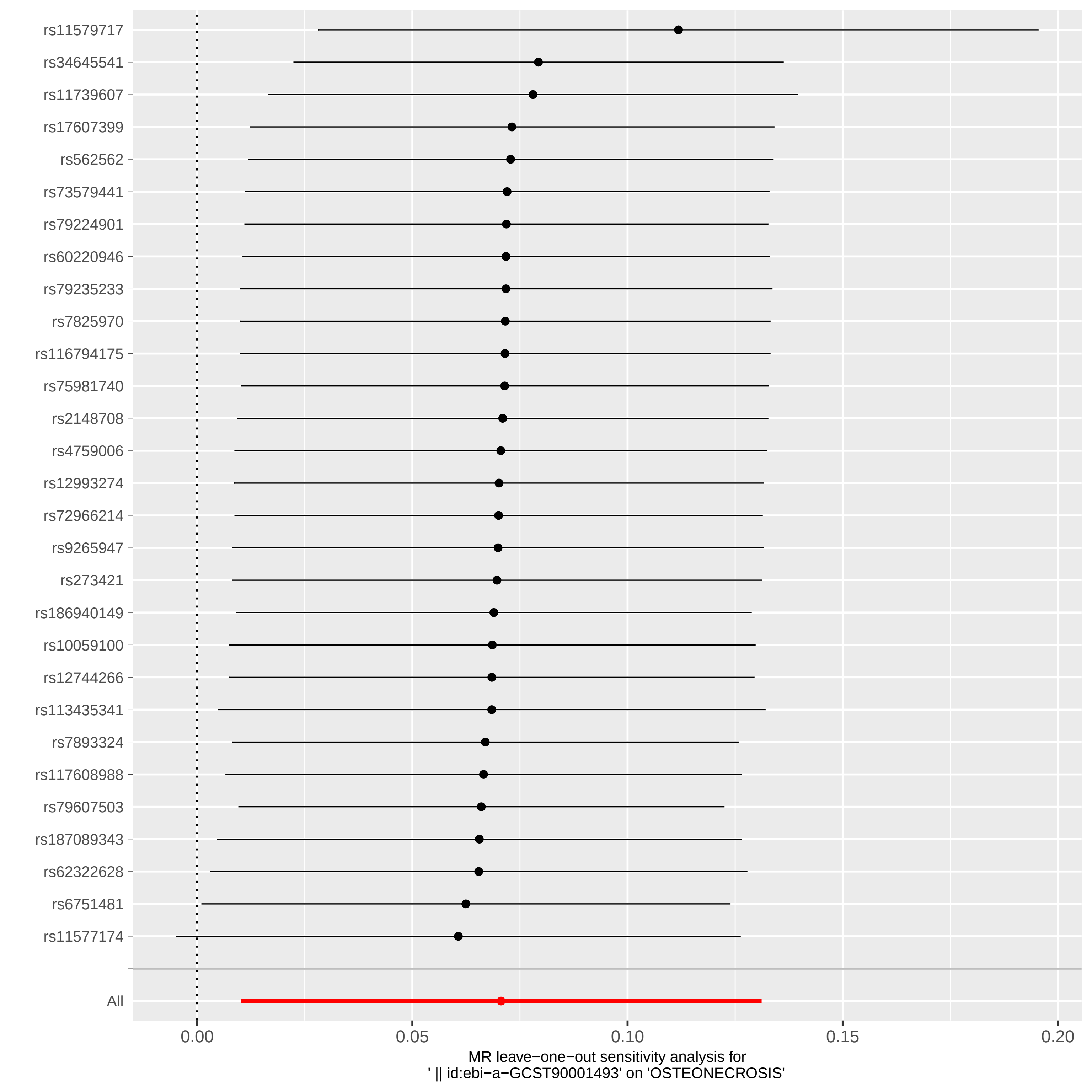
rs11579717
rs34645541
rs11739607
rs17607399
rs562562
rs73579441
rs79224901
rs60220946
rs79235233
rs7825970
rs116794175
rs75981740
rs2148708
rs4759006
rs12993274
rs72966214
rs9265947
rs273421
rs186940149
rs10059100
rs12744266
rs113435341
rs7893324
rs117608988
rs79607503
rs187089343
rs62322628
rs6751481
rs11577174
All
0.00
0.05
0.10
0.15
0.20
MR leave−one−out sensitivity analysis for
' || id:ebi−a−GCST90001493' on 'OSTEONECROSIS'

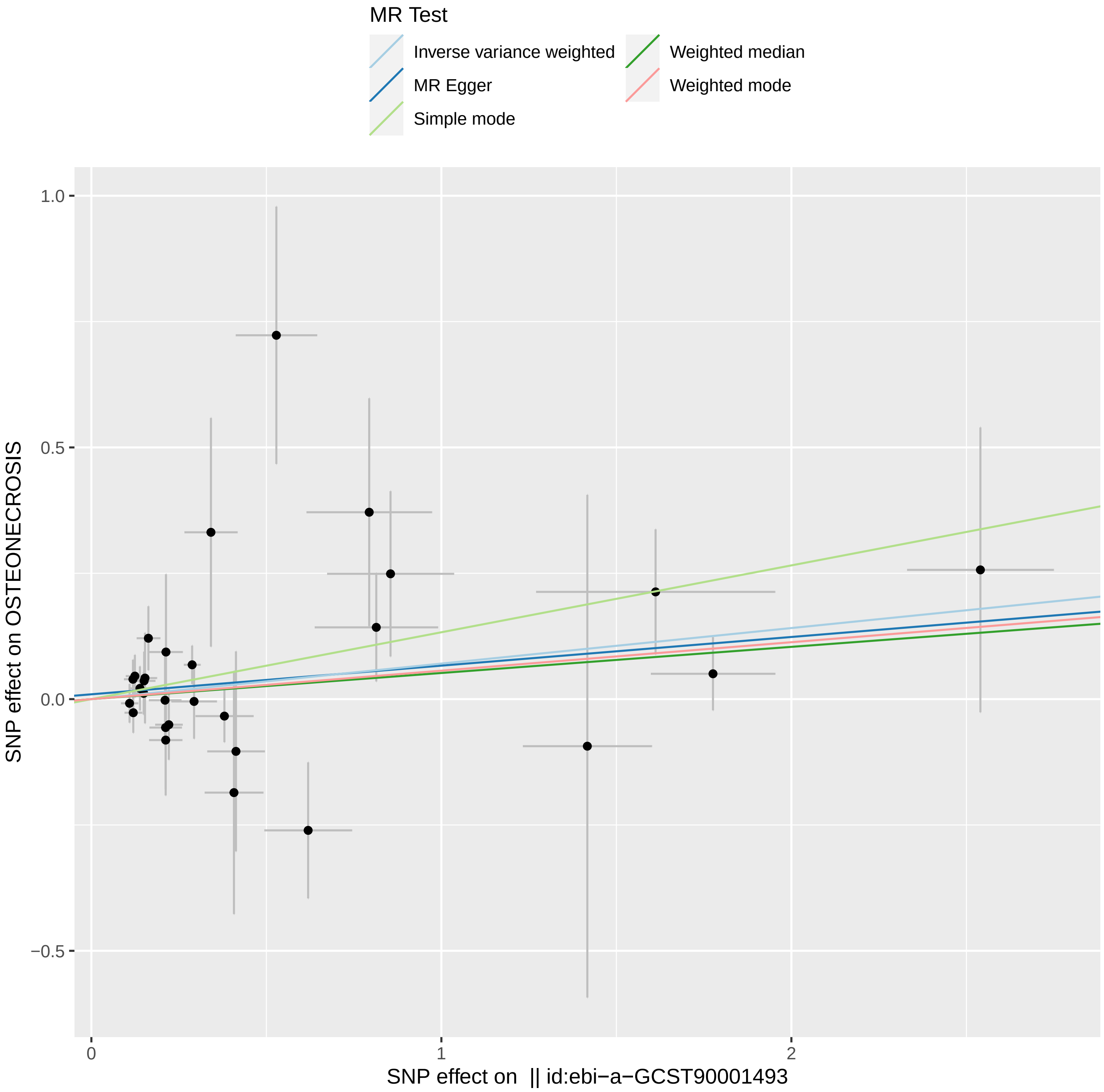

MR Test
Inverse variance weighted
MR Egger
Simple mode
Weighted median
Weighted mode
1.0
0.5
0.0
−0.5
0
1
2
SNP effect on OSTEONECROSIS
SNP effect on || id:ebi−a−GCST90001493

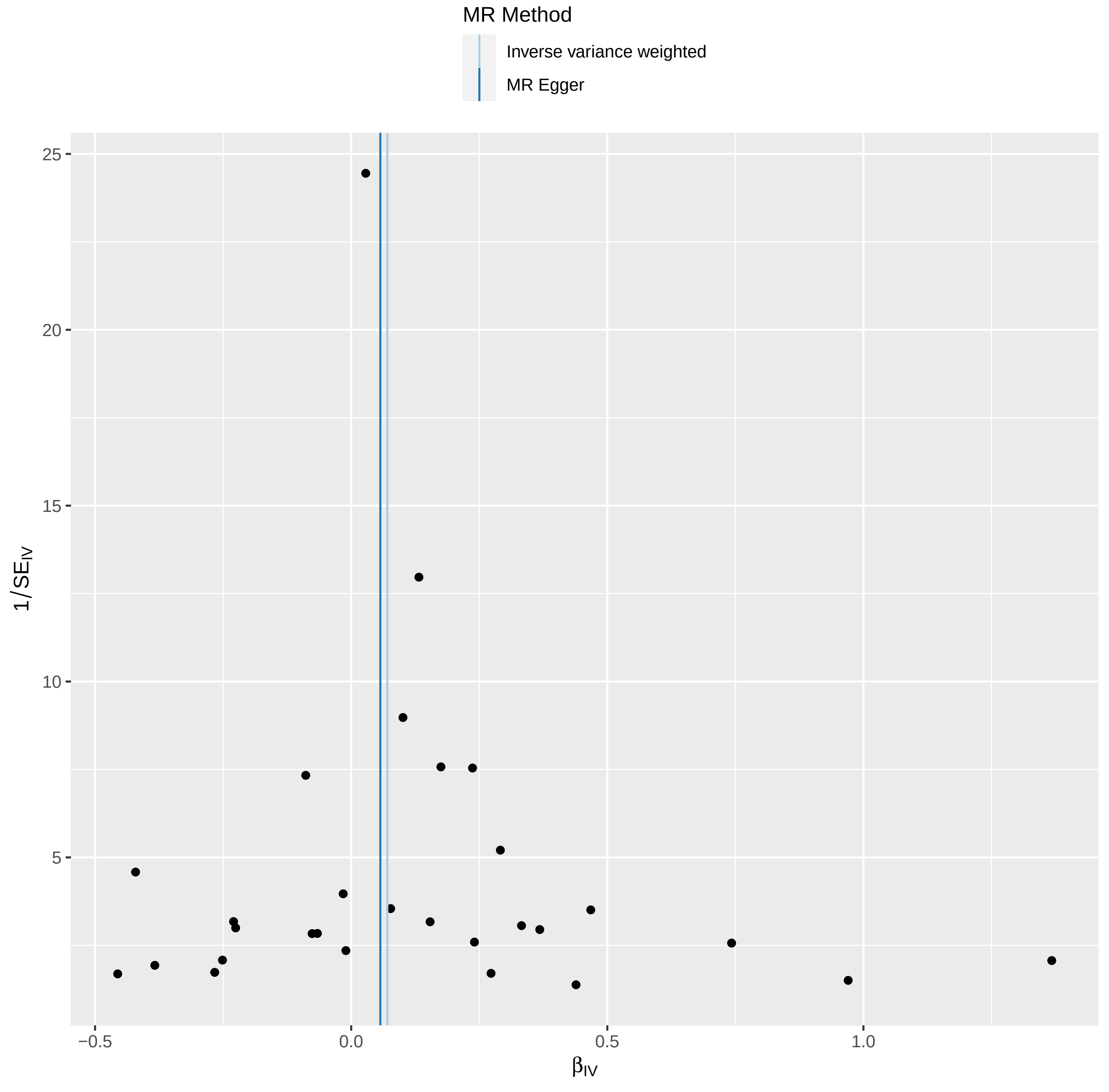

MR Method
Inverse variance weighted
MR Egger
25
20
15
10
5
$1/SE_{IV}$
−0.5
0.0
0.5
1.0
β_{IV}

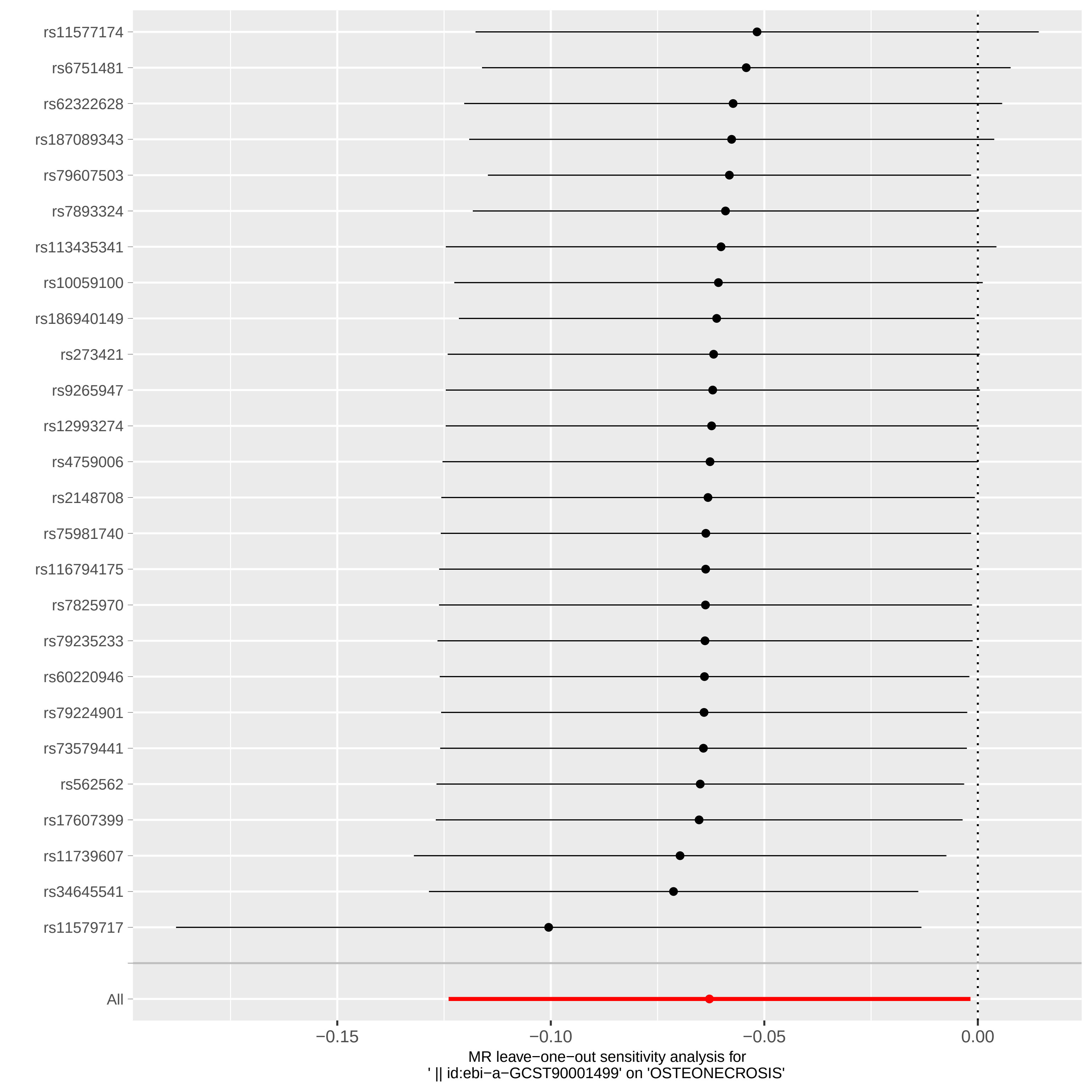
rs11577174
rs6751481
rs62322628
rs187089343
rs79607503
rs7893324
rs113435341
rs10059100
rs186940149
rs273421
rs9265947
rs12993274
rs4759006
rs2148708
rs75981740
rs116794175
rs7825970
rs79235233
rs60220946
rs79224901
rs73579441
rs562562
rs17607399
rs11739607
rs34645541
rs11579717
All
−0.15
−0.10
−0.05
0.00
MR leave−one−out sensitivity analysis for
' || id:ebi−a−GCST90001499' on 'OSTEONECROSIS'

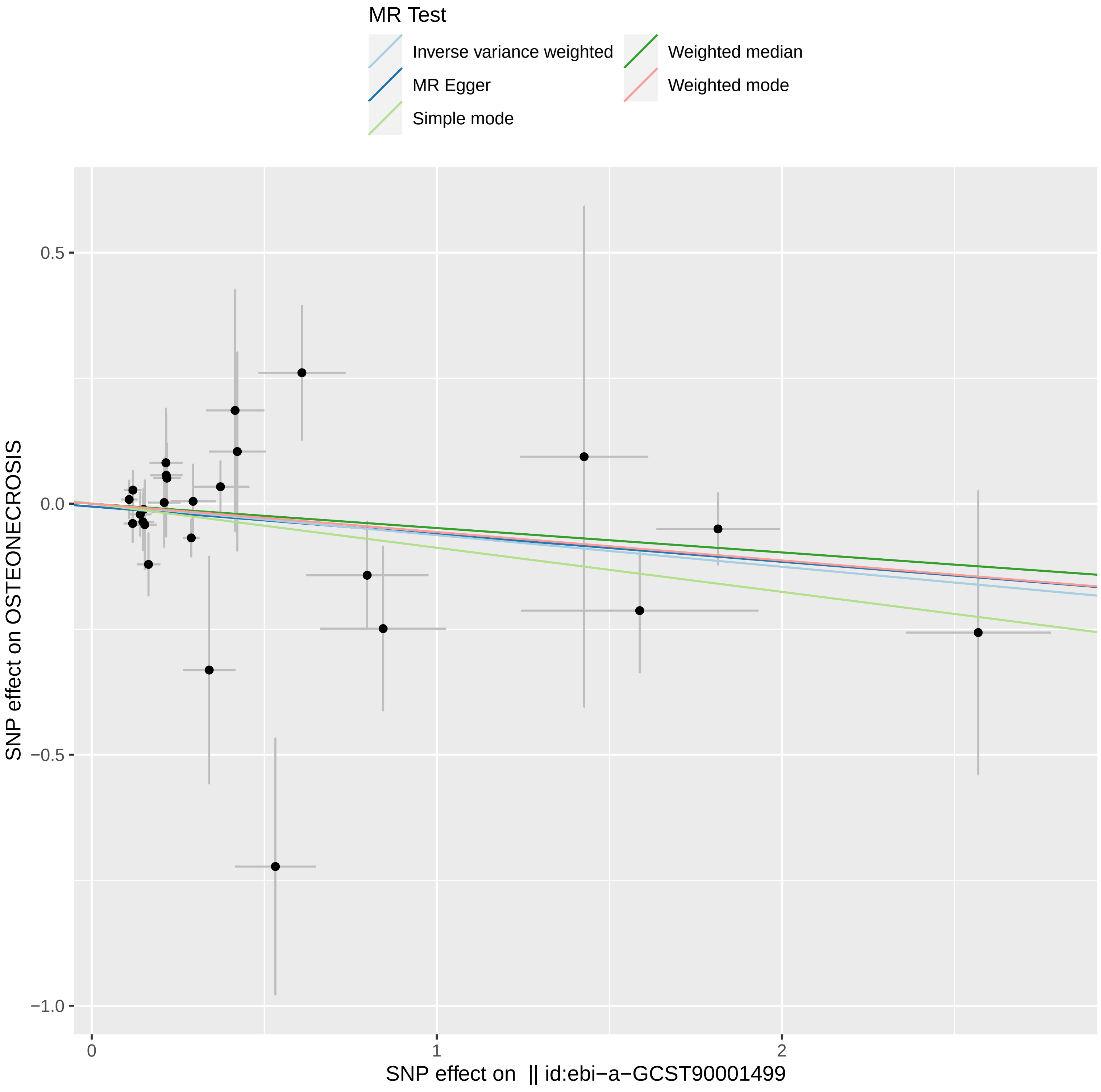
MR Test
Inverse variance weighted
MR Egger
Simple mode
Weighted median
Weighted mode
0.5
0.0
−0.5
−1.0
0
1
2
SNP effect on OSTEONECROSIS
SNP effect on || id:ebi−a−GCST90001499

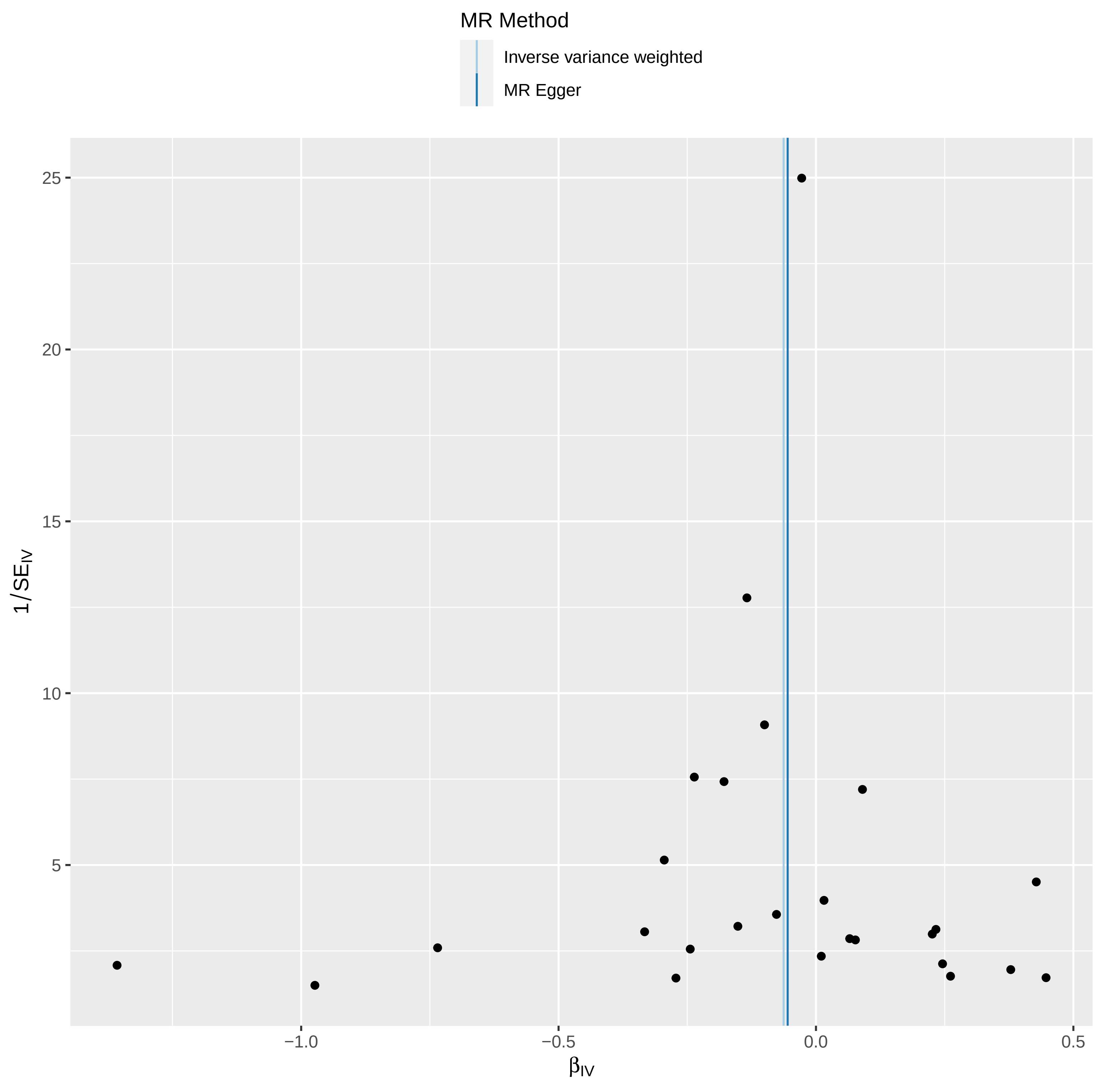
MR Method
Inverse variance weighted
MR Egger
25
20
15
10
5
1/SE_IV
−1.0
−0.5
0.0
0.5
β_IV

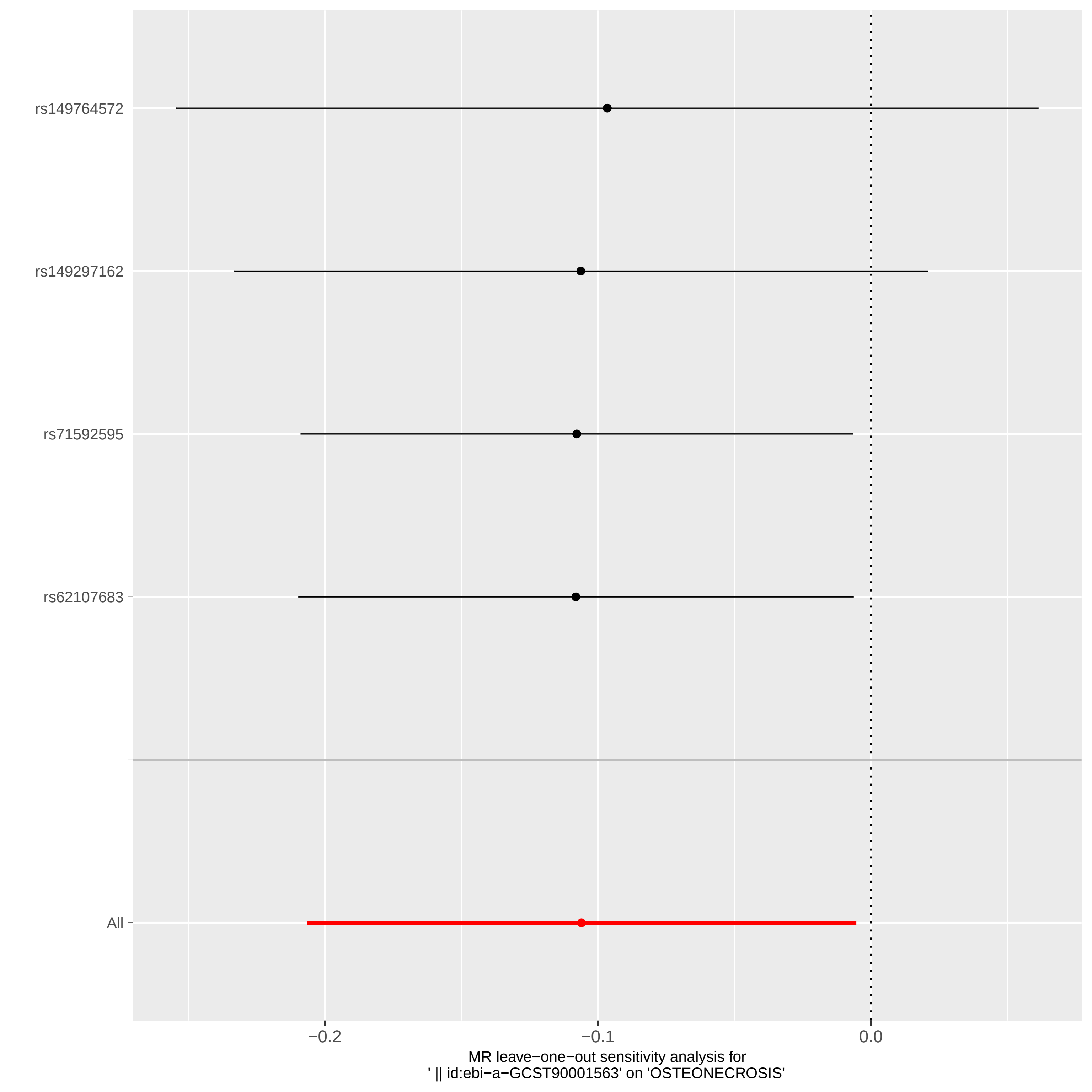
rs149764572
rs149297162
rs71592595
rs62107683
All
−0.2
−0.1
0.0
MR leave−one−out sensitivity analysis for
' || id:ebi−a−GCST90001563' on 'OSTEONECROSIS'

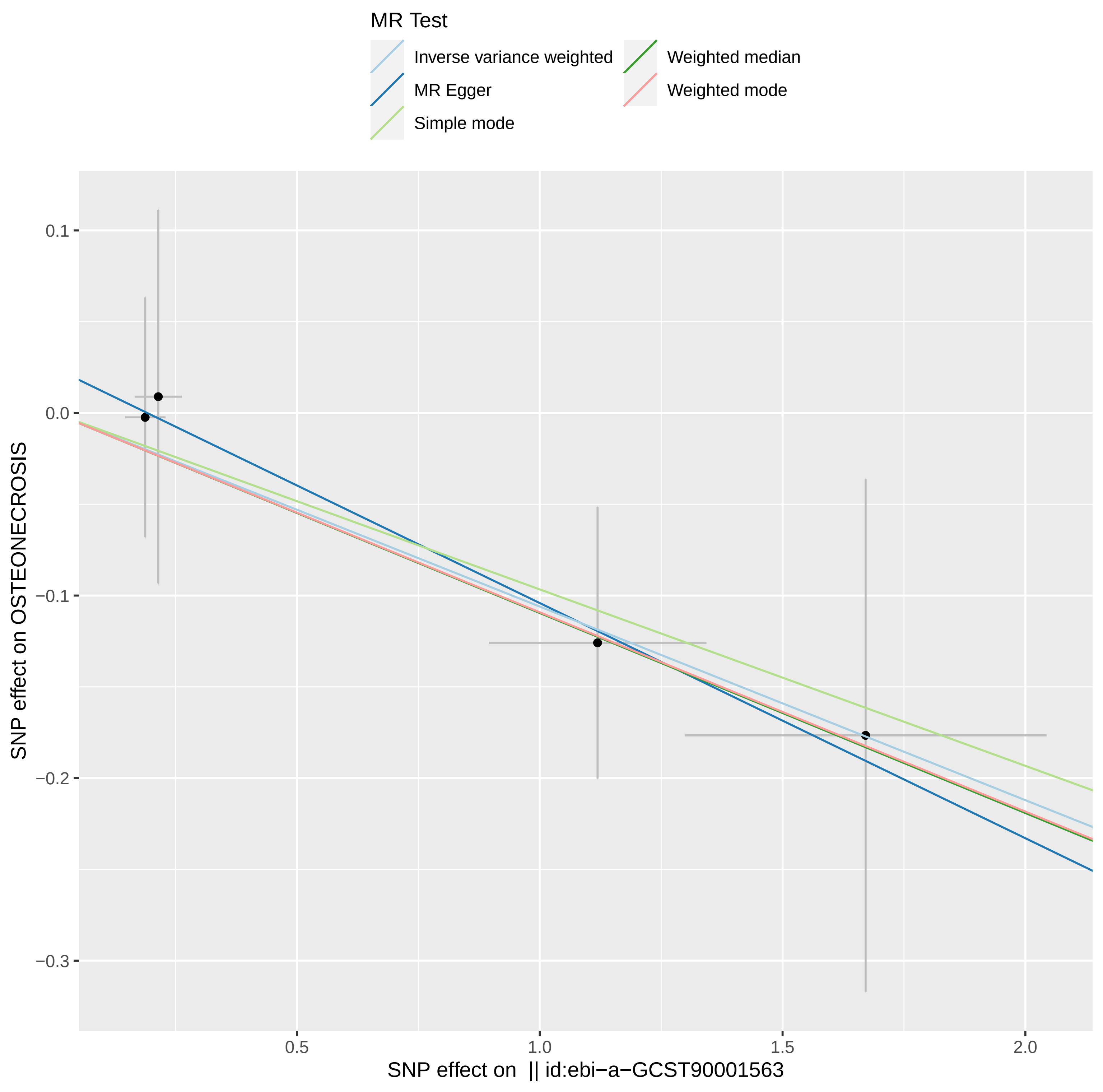
MR Test
Inverse variance weighted
MR Egger
Simple mode
Weighted median
Weighted mode
0.1
0.0
−0.1
−0.2
−0.3
0.5
1.0
1.5
2.0
SNP effect on OSTEONECROSIS
SNP effect on || id:ebi−a−GCST90001563

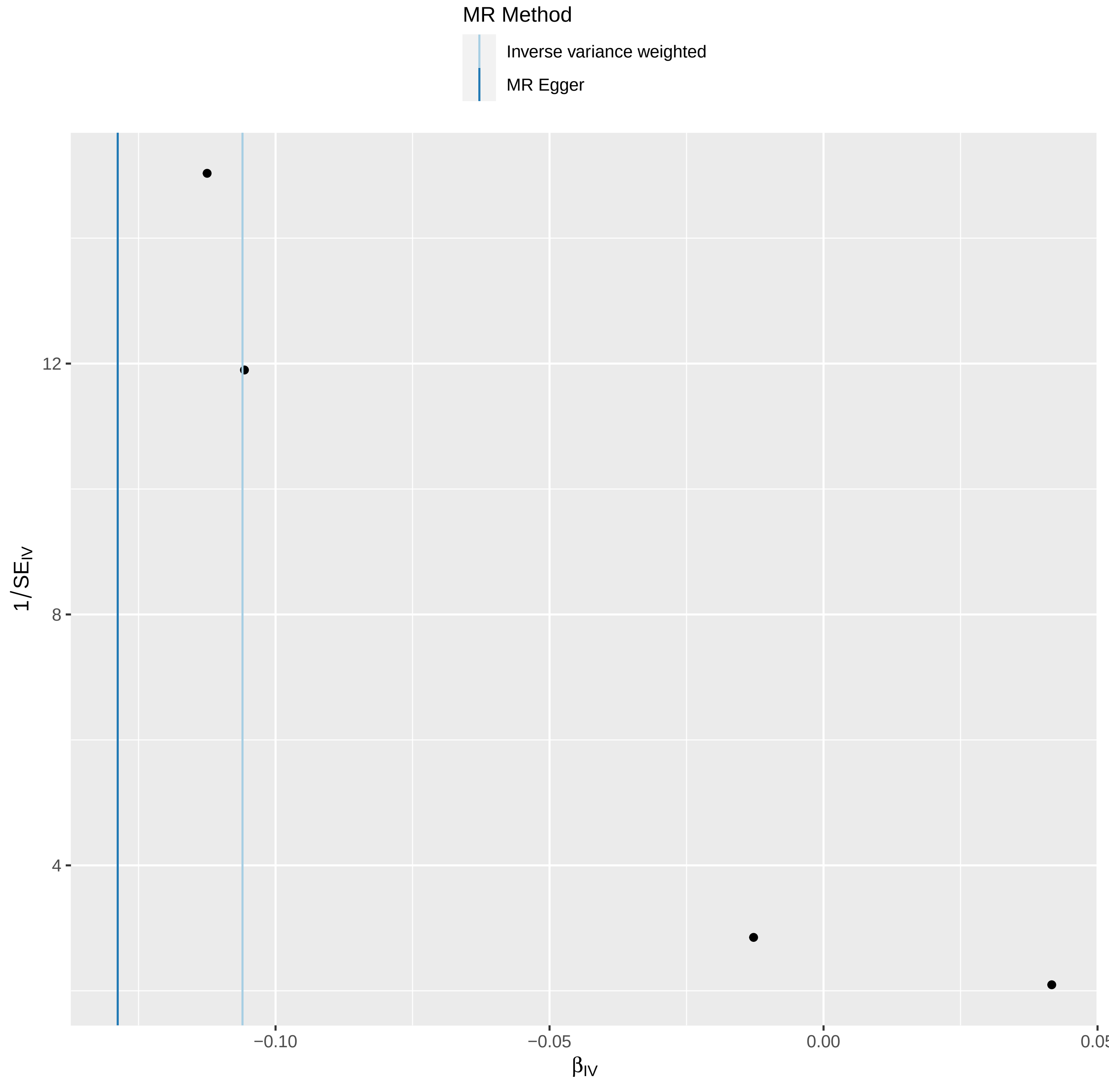
MR Method
Inverse variance weighted
MR Egger
12
8
4
1/SE_IV
−0.10
−0.05
0.00
0.05
β_IV

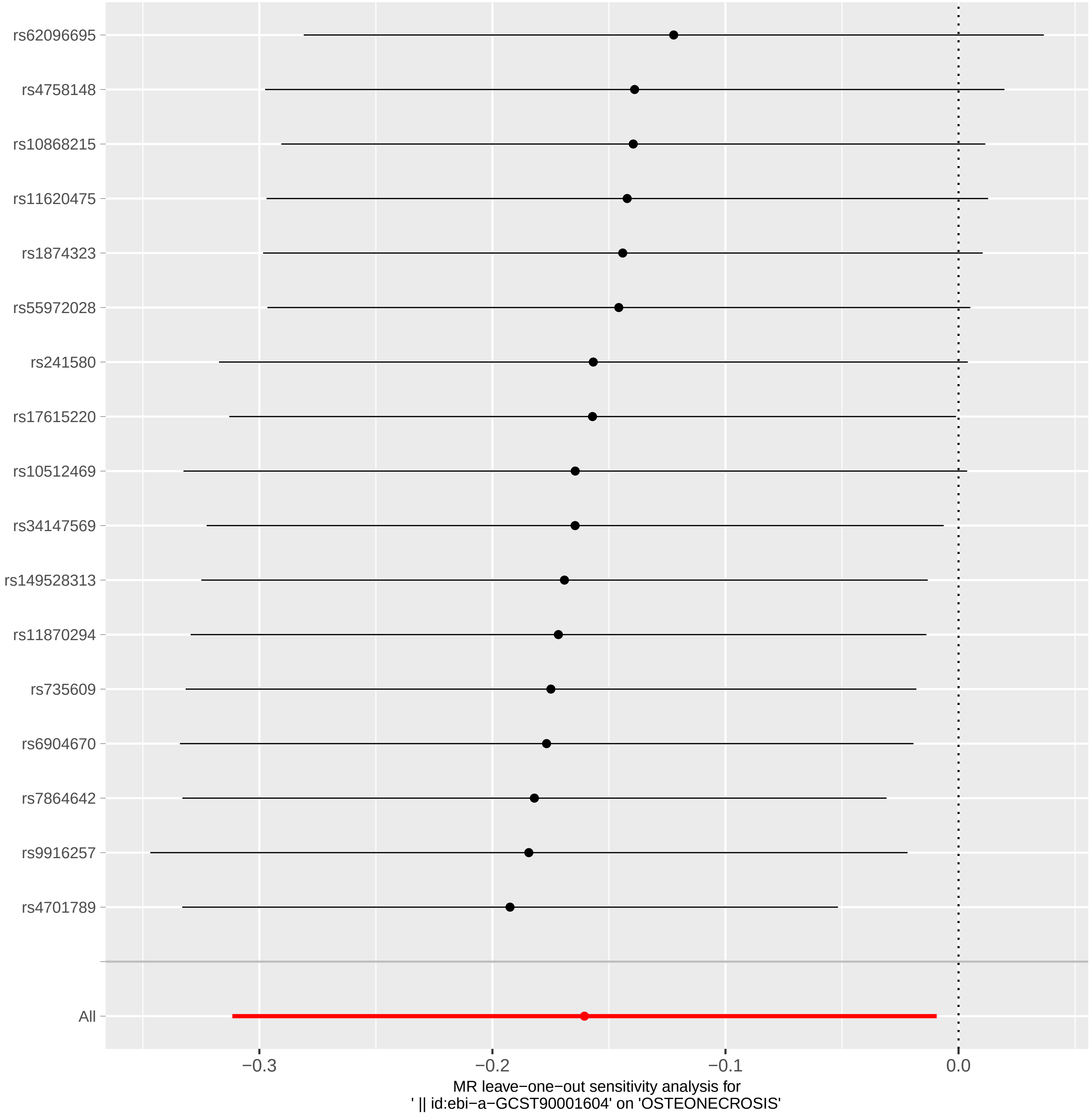
rs62096695
rs4758148
rs10868215
rs11620475
rs1874323
rs55972028
rs241580
rs17615220
rs10512469
rs34147569
rs149528313
rs11870294
rs735609
rs6904670
rs7864642
rs9916257
rs4701789
All
−0.3
−0.2
−0.1
0.0
MR leave−one−out sensitivity analysis for
' || id:ebi−a−GCST90001604' on 'OSTEONECROSIS'

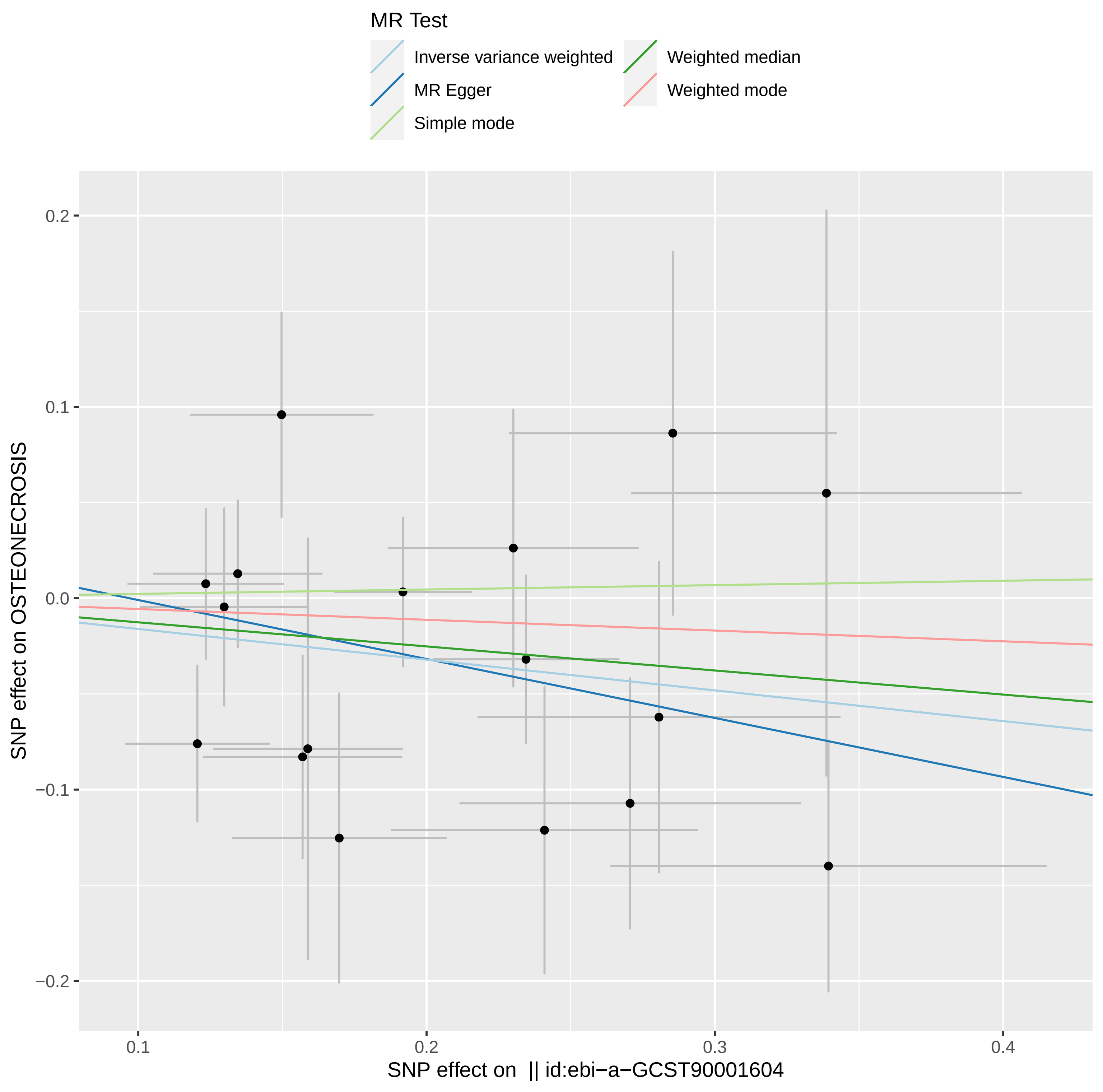
MR Test
Inverse variance weighted
MR Egger
Simple mode
Weighted median
Weighted mode
0.2
0.1
0.0
−0.1
−0.2
0.1
0.2
0.3
0.4
SNP effect on OSTEONECROSIS
SNP effect on || id:ebi−a−GCST90001604

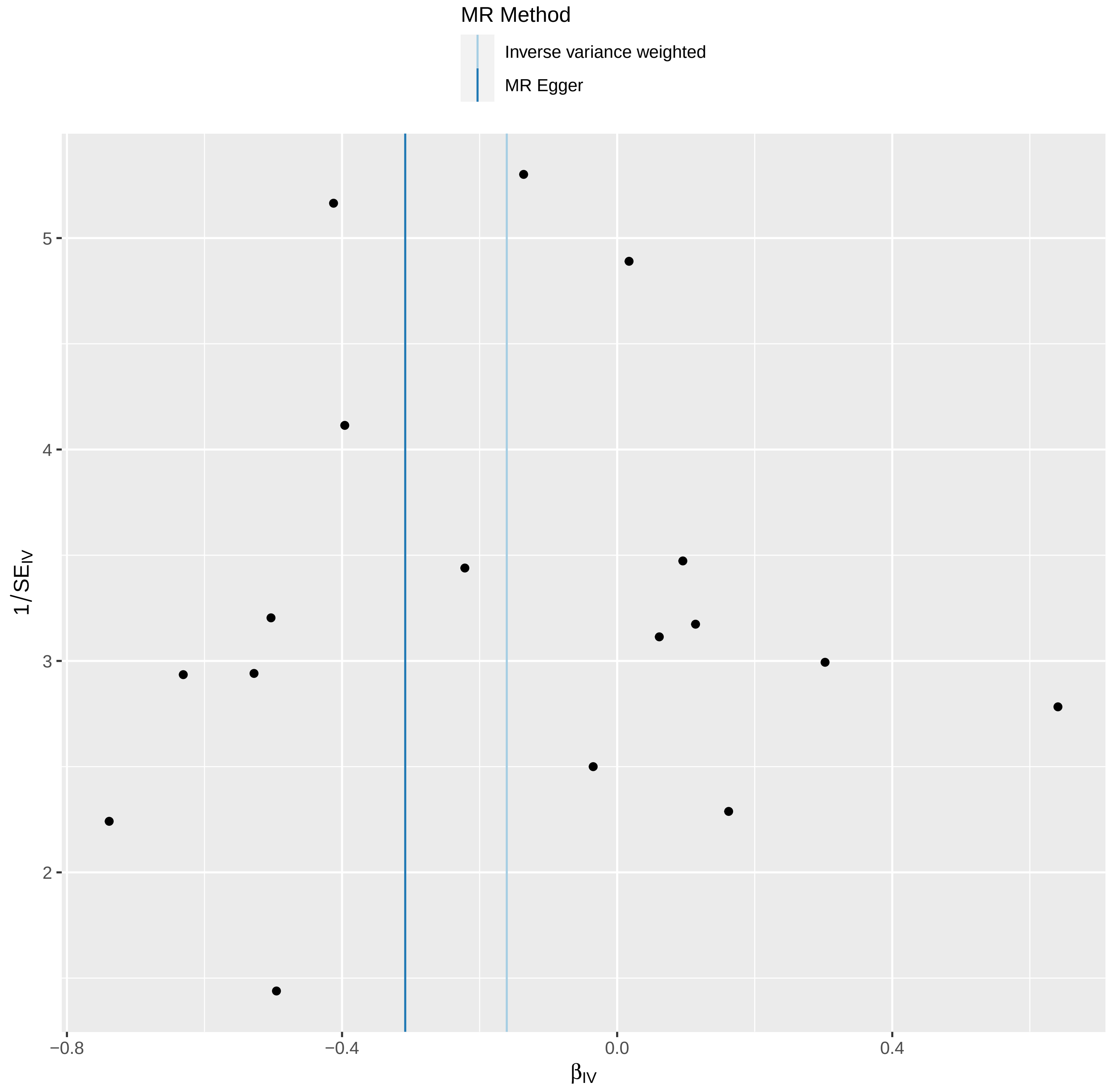

MR Method
Inverse variance weighted
MR Egger
5
4
3
2
1/SE_IV
−0.8
−0.4
0.0
0.4
β_IV

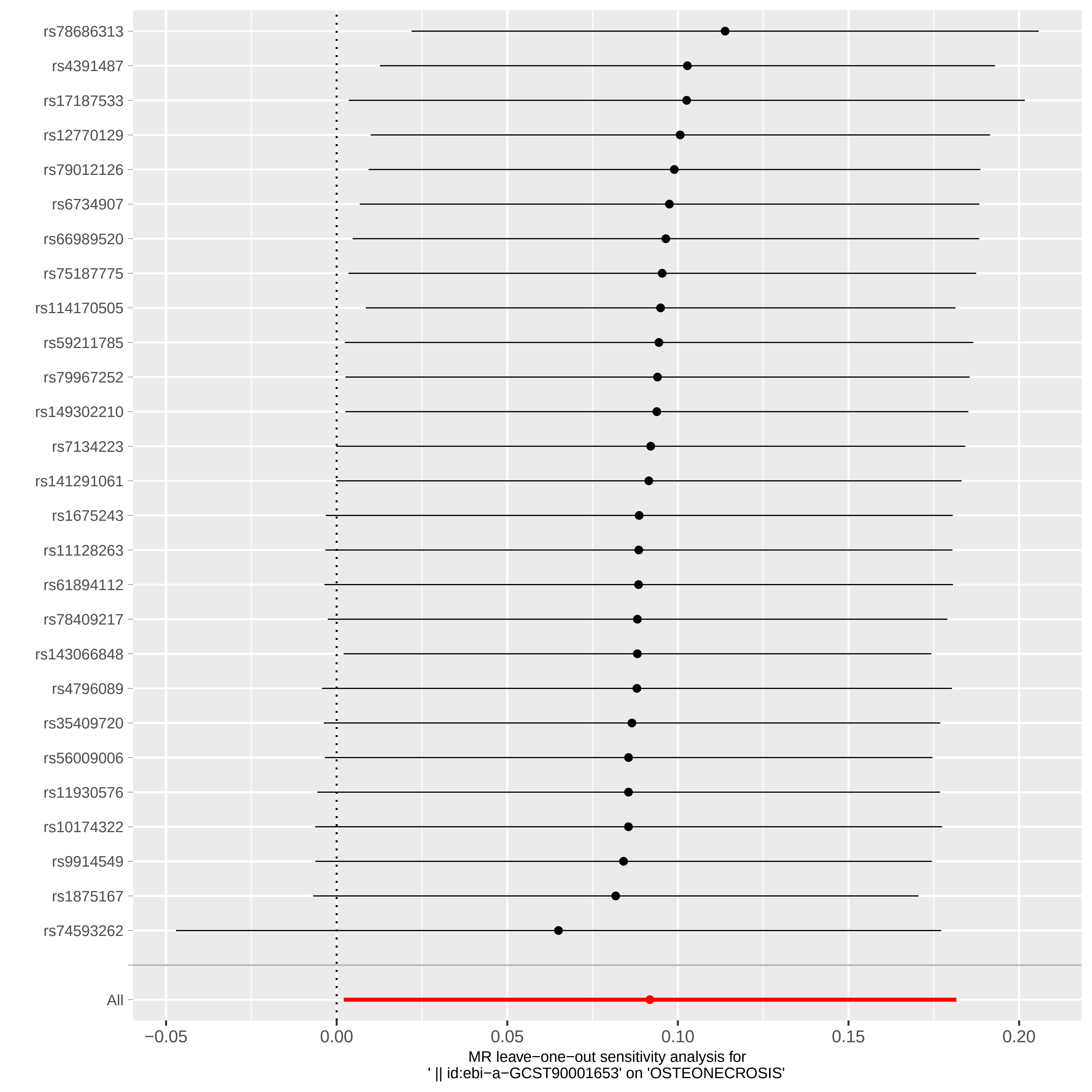
rs78686313
rs4391487
rs17187533
rs12770129
rs79012126
rs6734907
rs66989520
rs75187775
rs114170505
rs59211785
rs79967252
rs149302210
rs7134223
rs141291061
rs1675243
rs11128263
rs61894112
rs78409217
rs143066848
rs4796089
rs35409720
rs56009006
rs11930576
rs10174322
rs9914549
rs1875167
rs74593262
All
−0.05
0.00
0.05
0.10
0.15
0.20
MR leave−one−out sensitivity analysis for
' || id:ebi−a−GCST90001653' on 'OSTEONECROSIS'

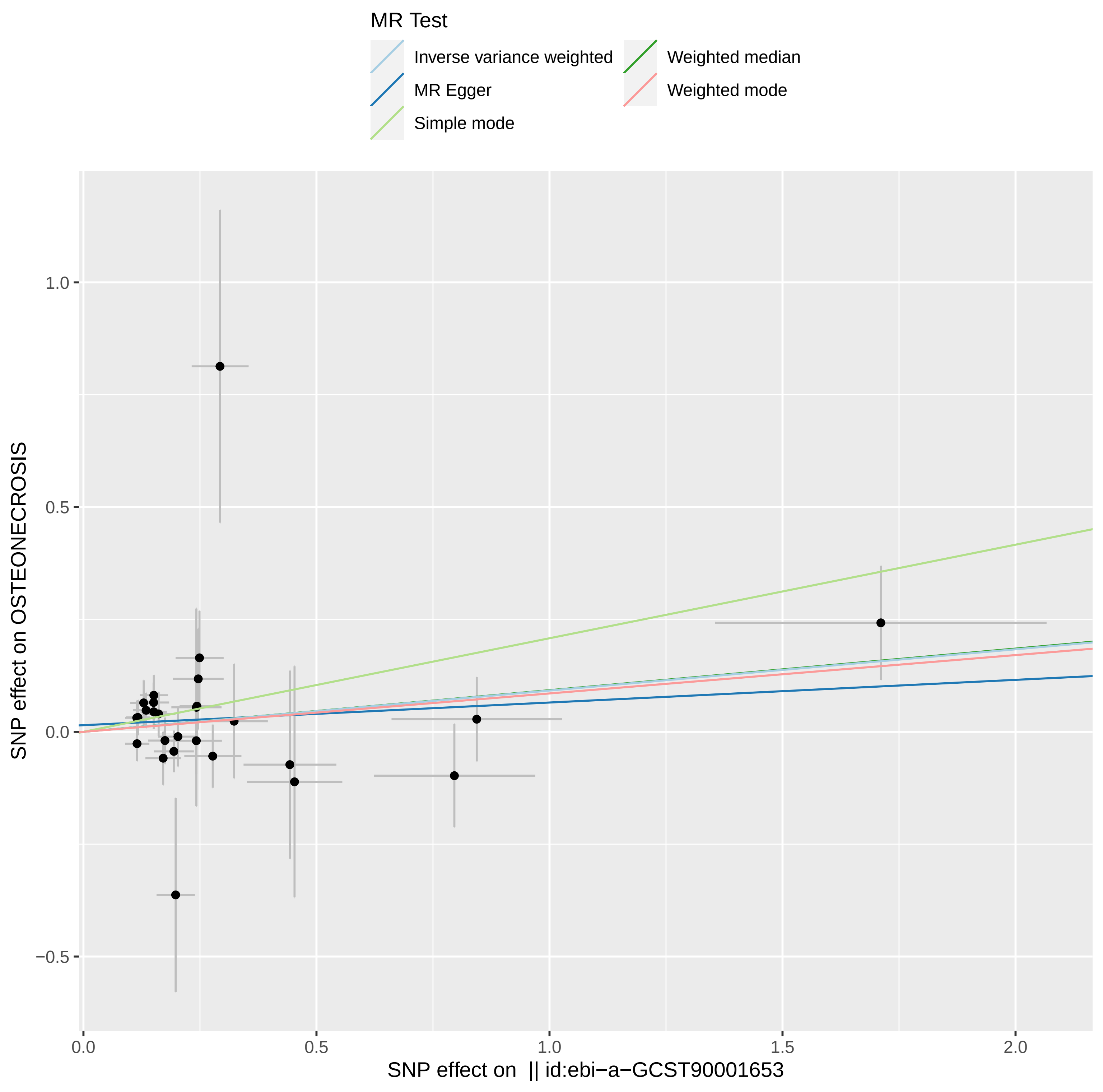
MR Test
Inverse variance weighted
MR Egger
Simple mode
Weighted median
Weighted mode
SNP effect on OSTEONECROSIS
1.0
0.5
0.0
−0.5
0.0
0.5
1.0
1.5
2.0
SNP effect on || id:ebi−a−GCST90001653

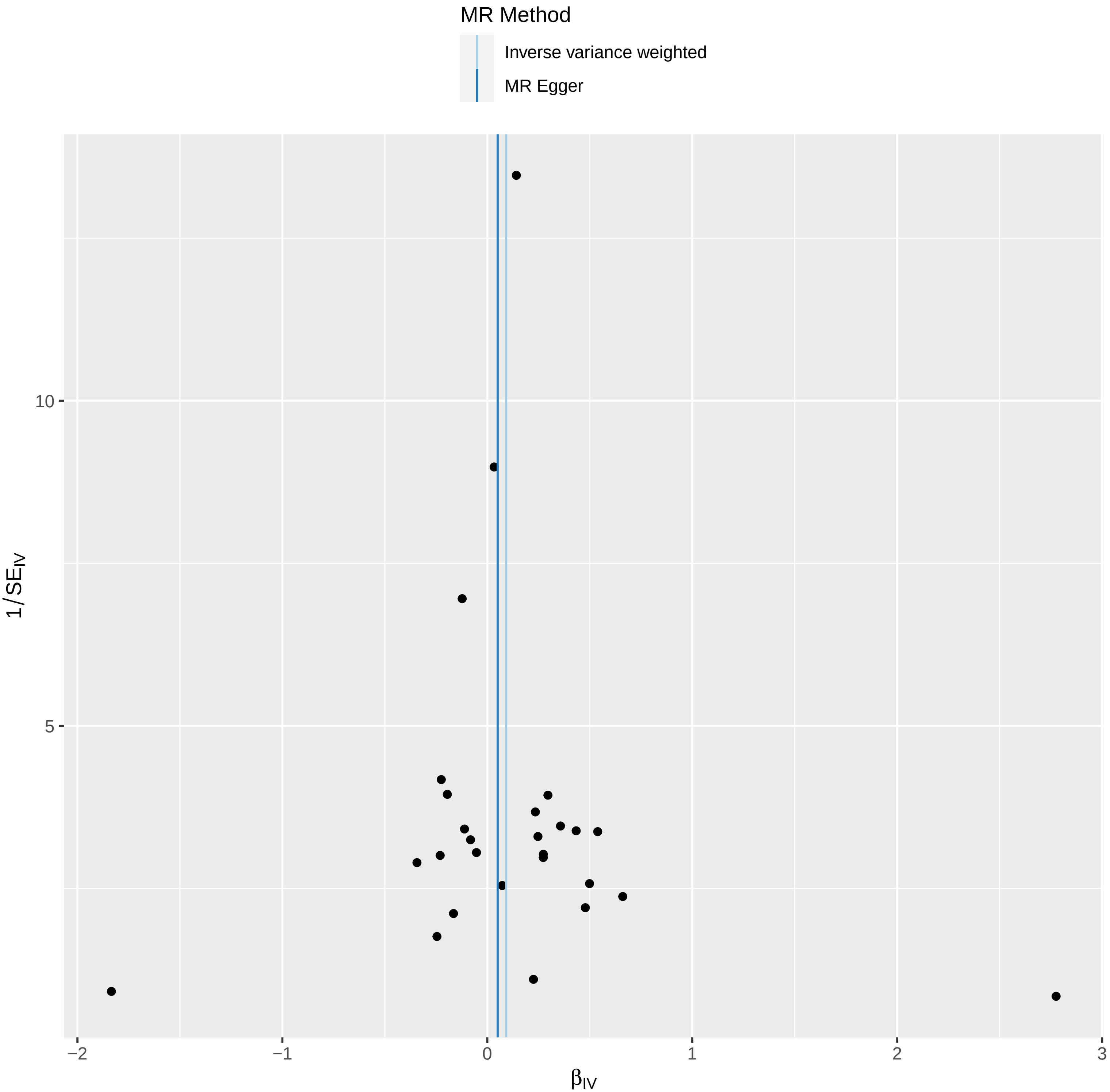

MR Method
Inverse variance weighted
MR Egger
10
5
$1/SE_{IV}$
−2
−1
0
1
2
3
β_{IV}

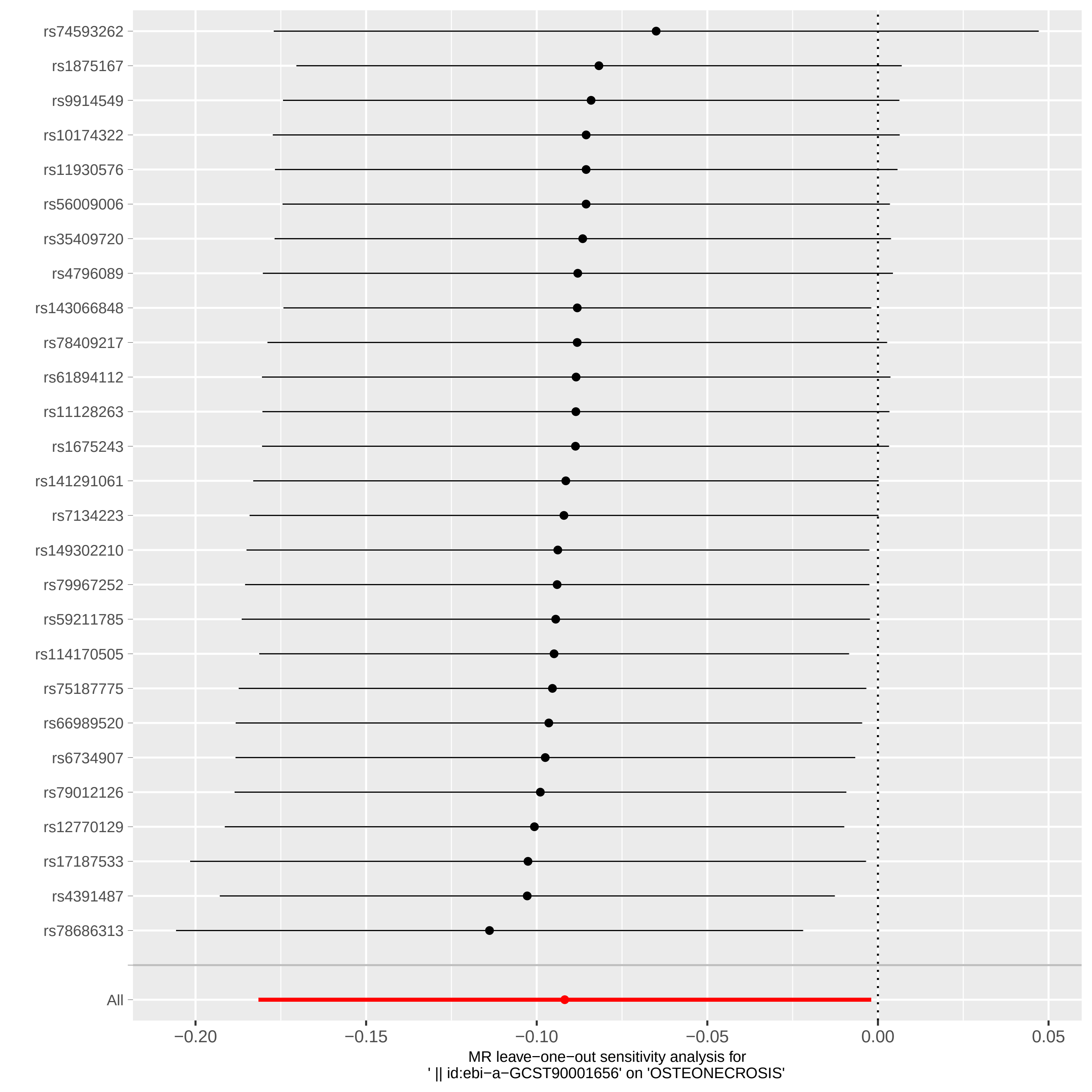

rs74593262
rs1875167
rs9914549
rs10174322
rs11930576
rs56009006
rs35409720
rs4796089
rs143066848
rs78409217
rs61894112
rs11128263
rs1675243
rs141291061
rs7134223
rs149302210
rs79967252
rs59211785
rs114170505
rs75187775
rs66989520
rs6734907
rs79012126
rs12770129
rs17187533
rs4391487
rs78686313
All
−0.20
−0.15
−0.10
−0.05
0.00
0.05
MR leave−one−out sensitivity analysis for
' || id:ebi−a−GCST90001656' on 'OSTEONECROSIS'

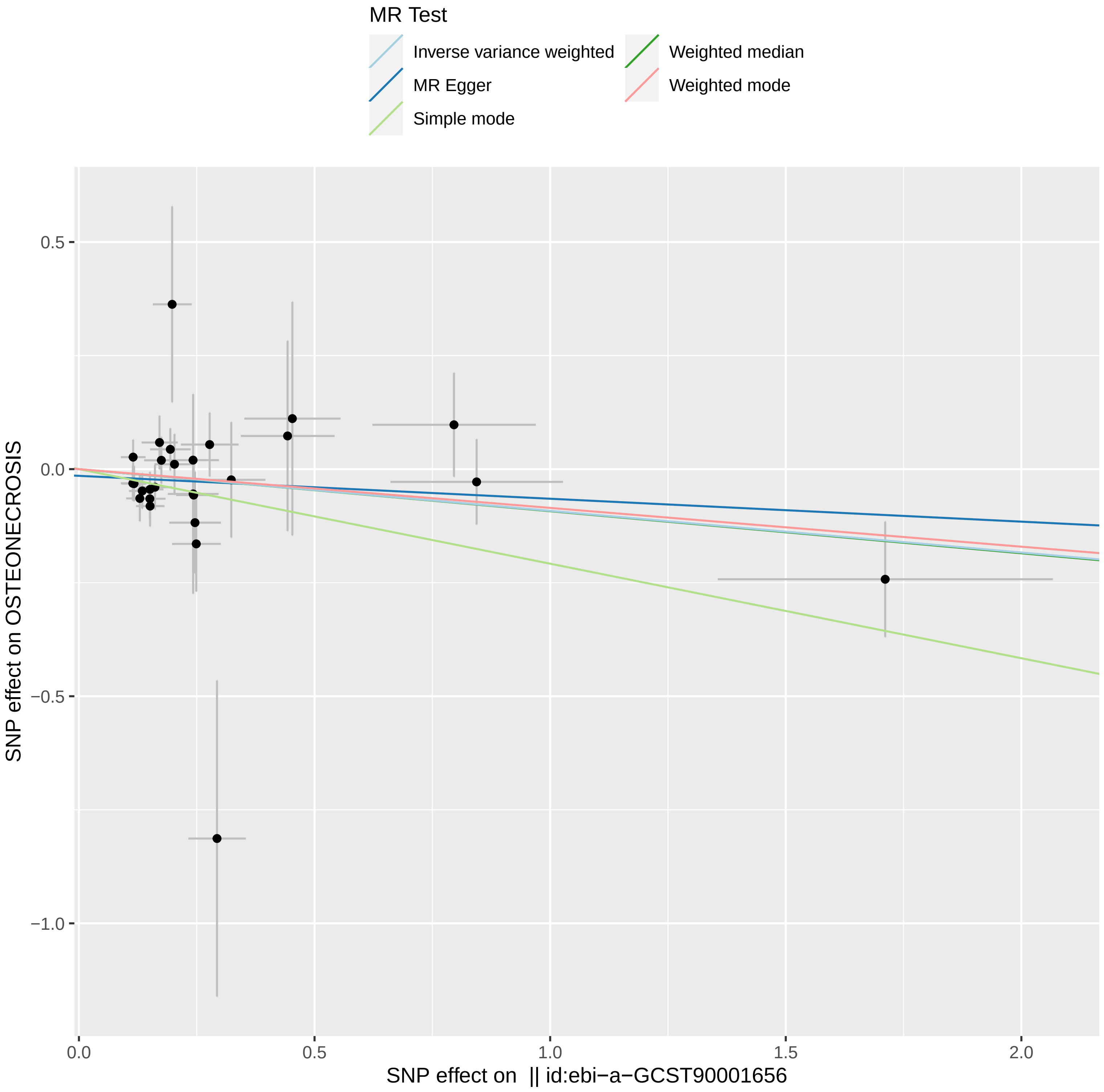
MR Test
Inverse variance weighted
MR Egger
Simple mode
Weighted median
Weighted mode
0.5
0.0
−0.5
−1.0
0.0
0.5
1.0
1.5
2.0
SNP effect on OSTEONECROSIS
SNP effect on || id:ebi−a−GCST90001656

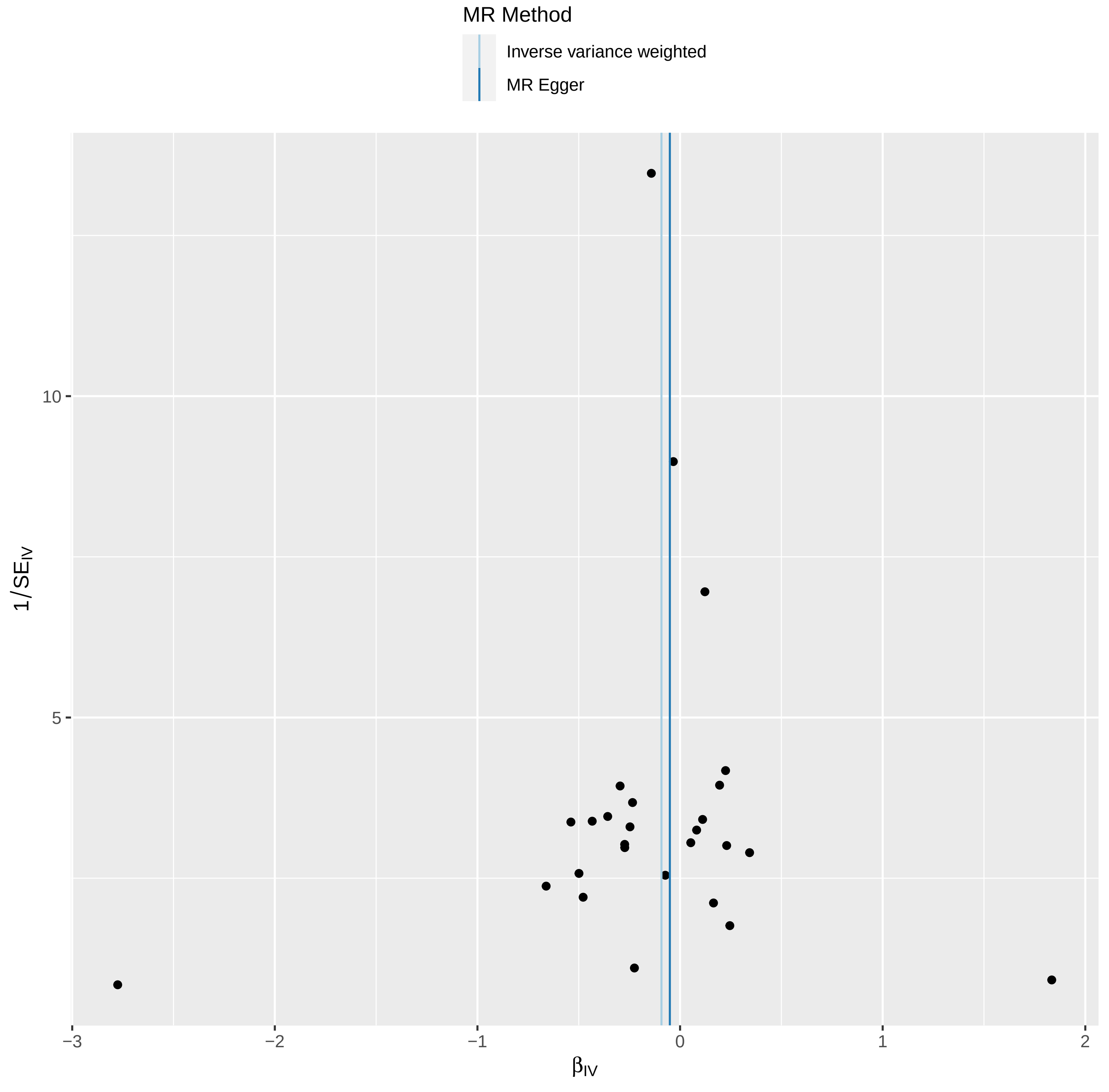

MR Method
Inverse variance weighted
MR Egger
10
5
1/SE_IV
−3
−2
−1
0
1
2
β_IV

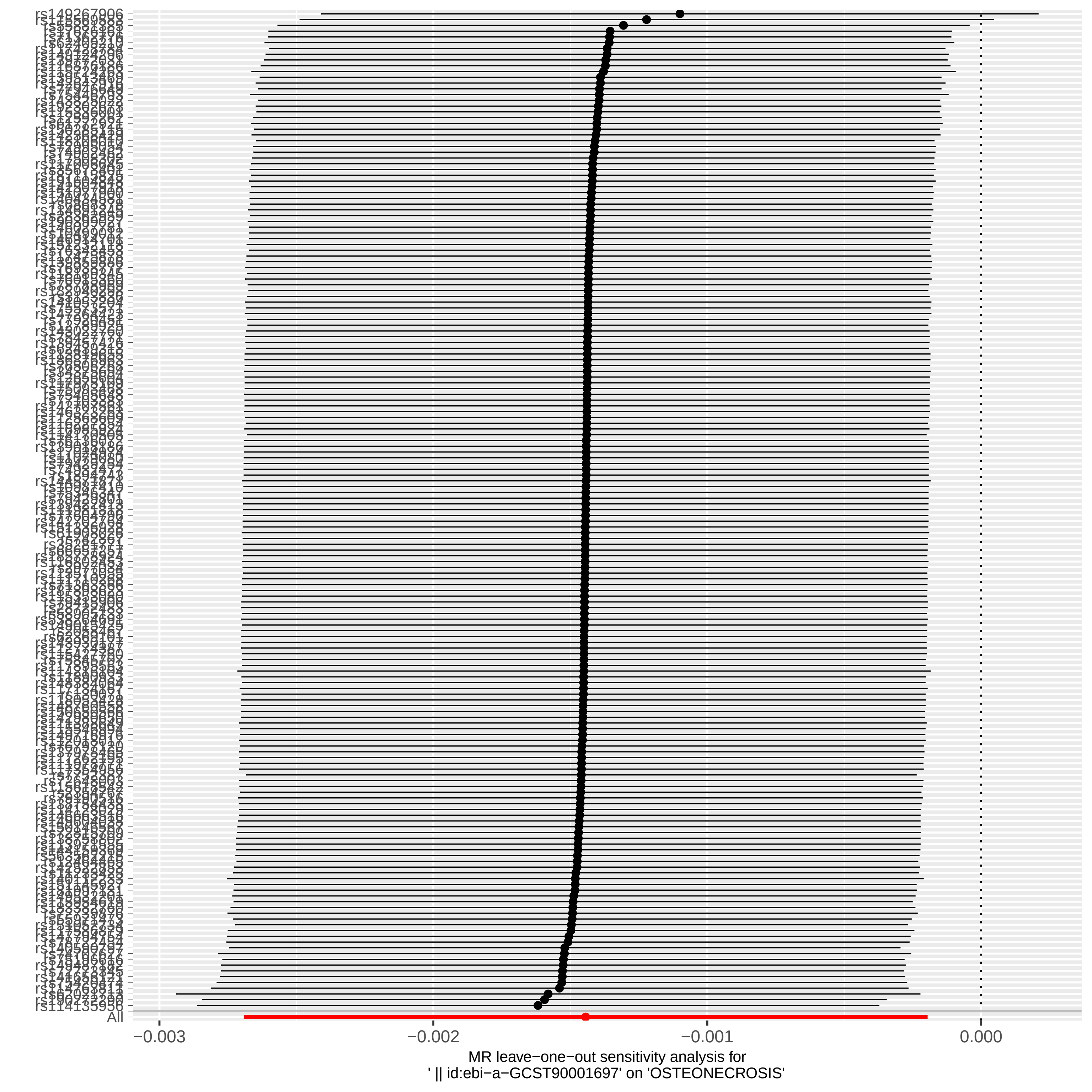
rs149267906
rs114135956
All
−0.003
−0.002
−0.001
0.000
MR leave−one−out sensitivity analysis for
' || id:ebi−a−GCST90001697' on 'OSTEONECROSIS'

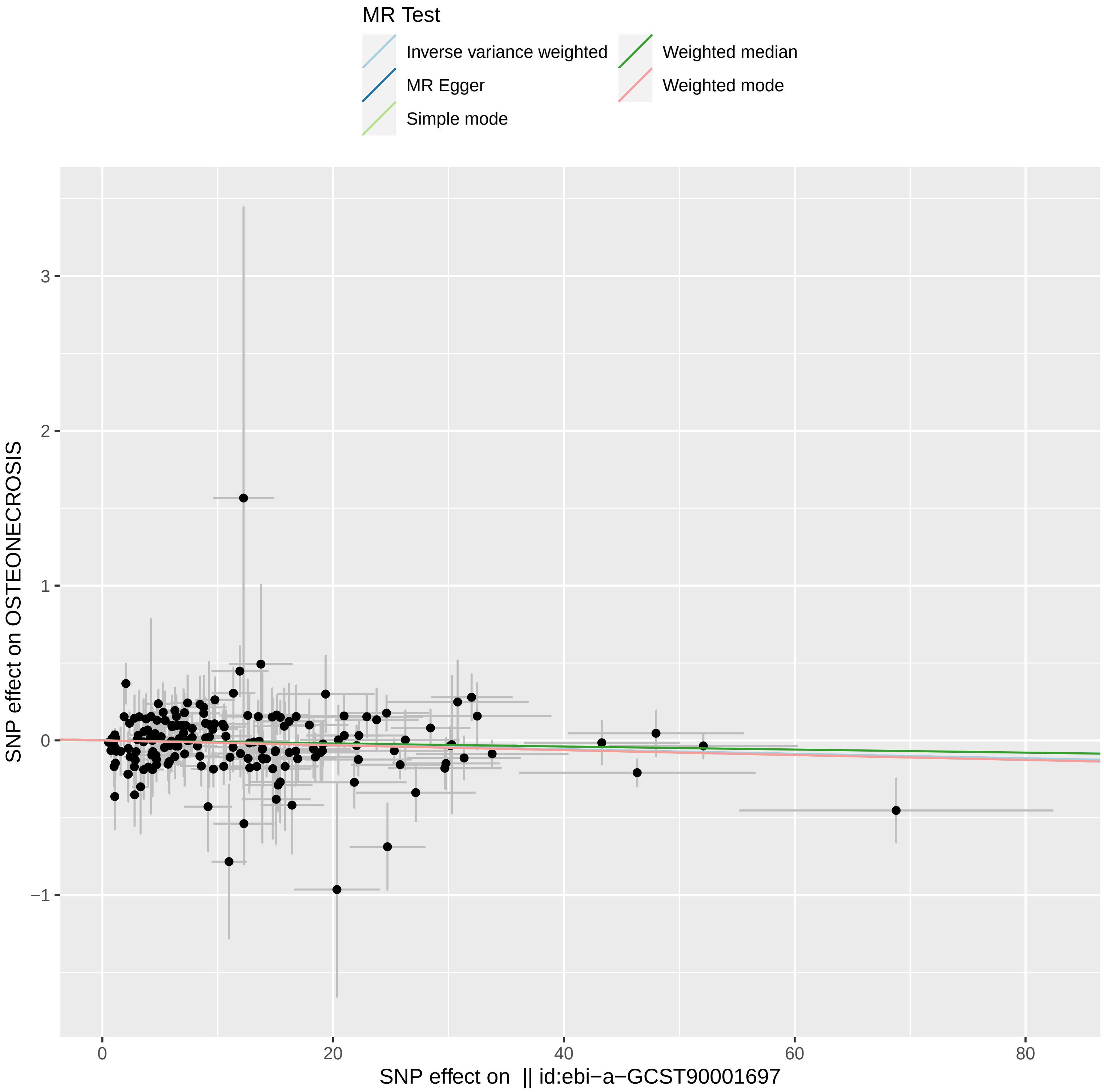
MR Test
Inverse variance weighted
MR Egger
Simple mode
Weighted median
Weighted mode
SNP effect on OSTEONECROSIS
SNP effect on || id:ebi−a−GCST90001697
3
2
1
0
−1
0
20
40
60
80

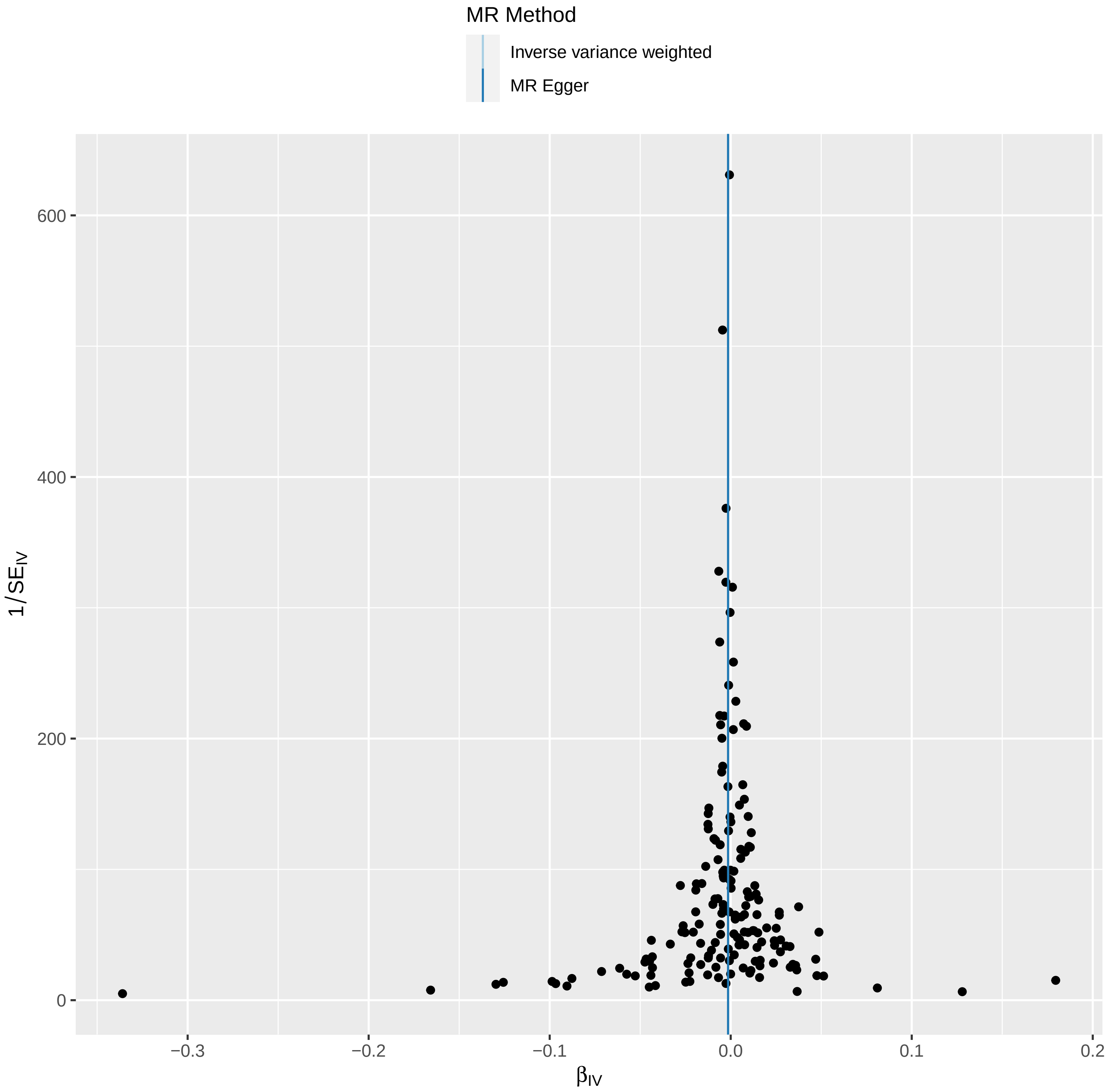
MR Method
Inverse variance weighted
MR Egger
600
400
200
0
1/SE_IV
−0.3
−0.2
−0.1
0.0
0.1
0.2
β_IV

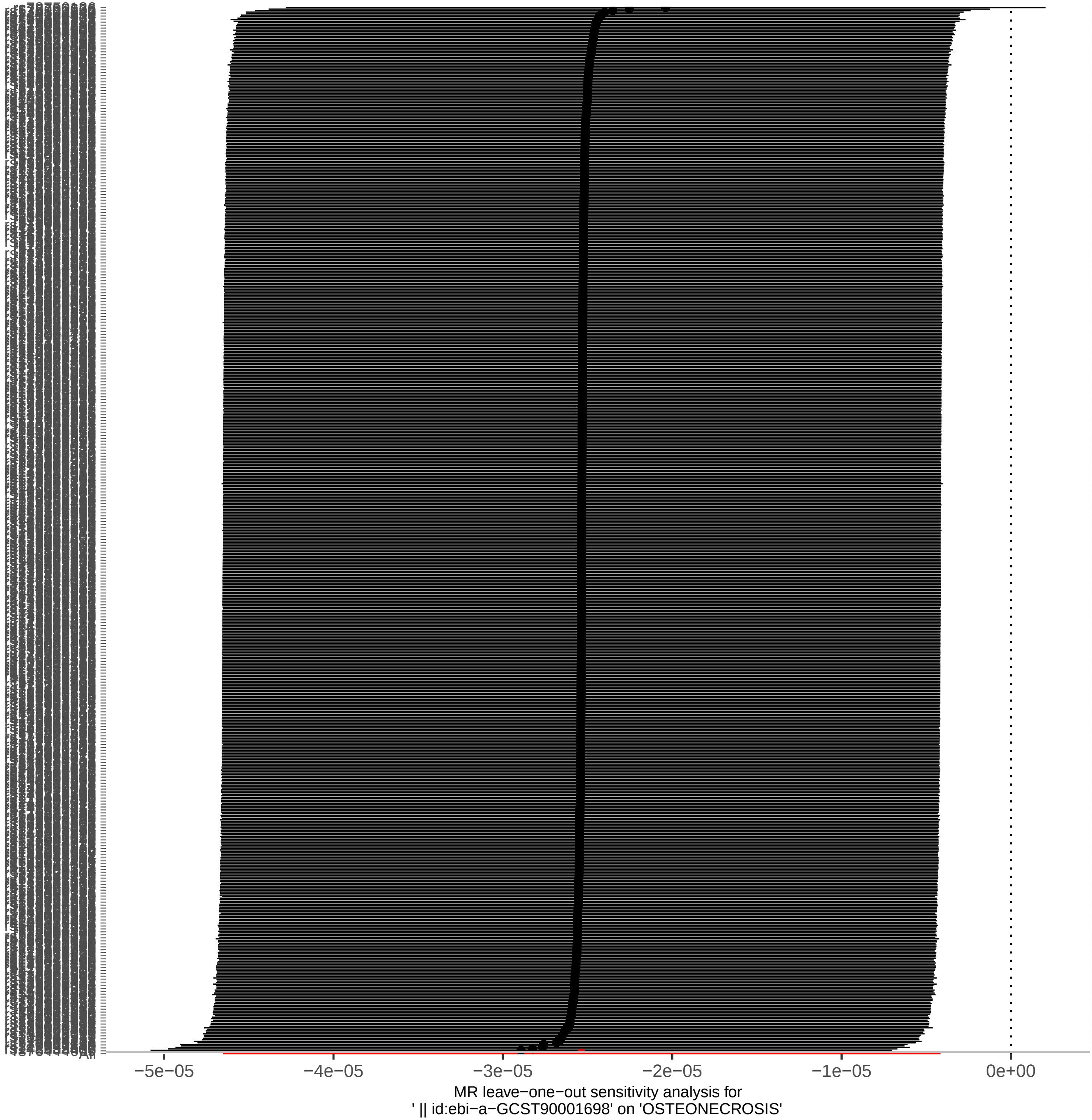

All
−5e−05
−4e−05
−3e−05
−2e−05
−1e−05
0e+00
MR leave−one−out sensitivity analysis for
' || id:ebi−a−GCST90001698' on 'OSTEONECROSIS'

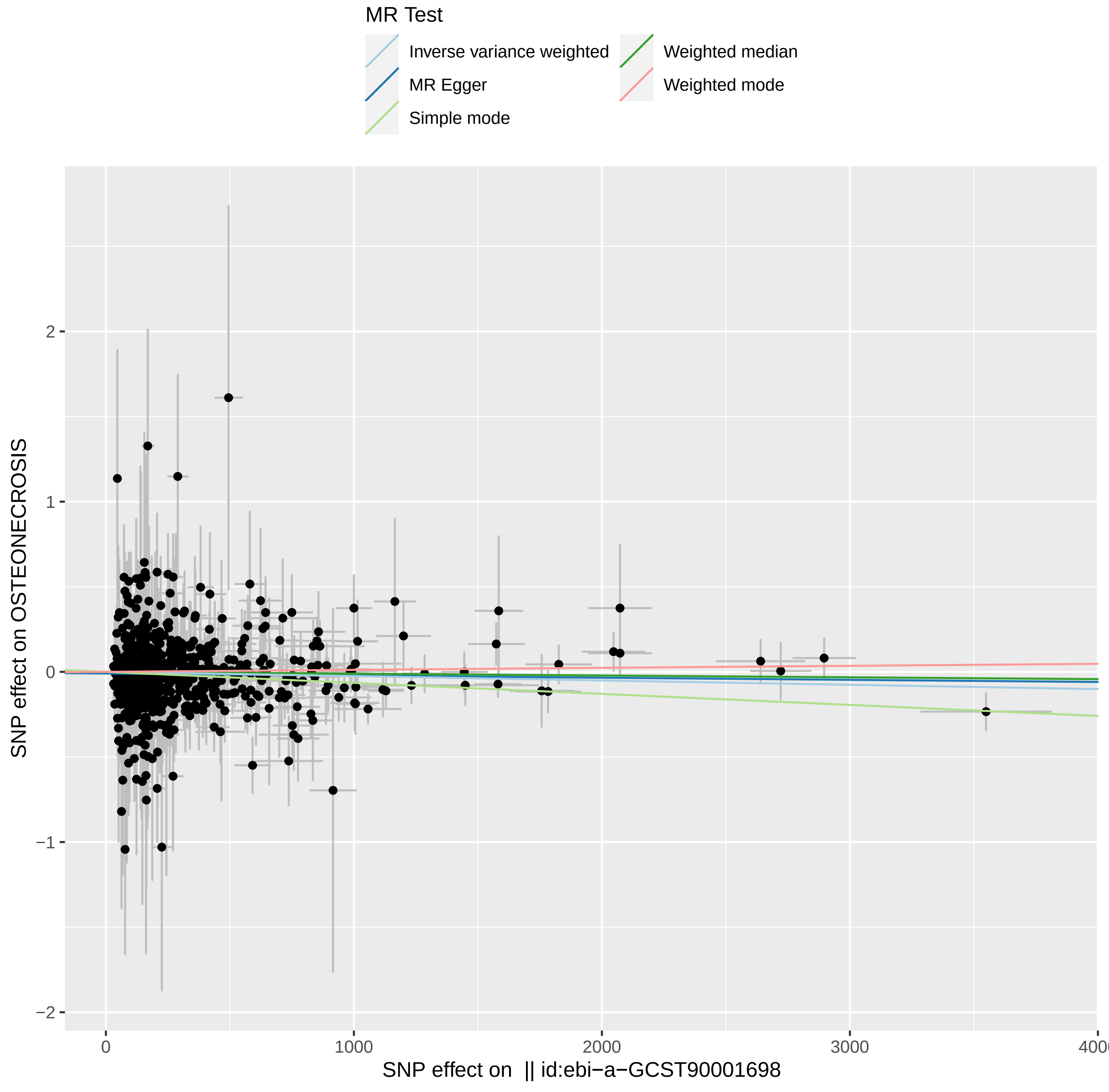
MR Test
Inverse variance weighted
MR Egger
Simple mode
Weighted median
Weighted mode
SNP effect on OSTEONECROSIS
2
1
0
−1
−2
0
1000
2000
3000
SNP effect on || id:ebi−a−GCST90001698

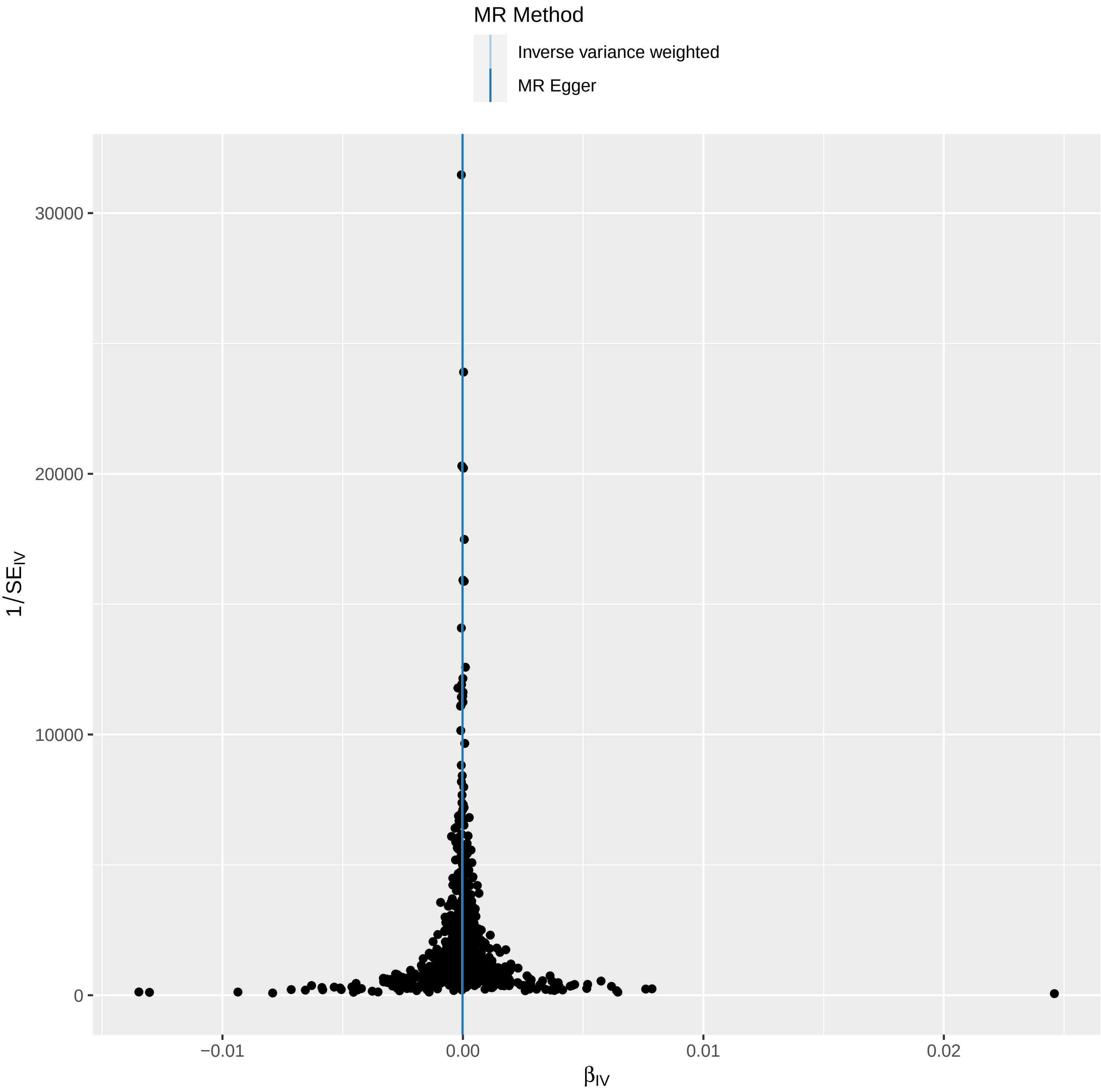
MR Method
Inverse variance weighted
MR Egger
30000
20000
10000
0
1/SE_IV
−0.01
0.00
0.01
0.02
β_IV

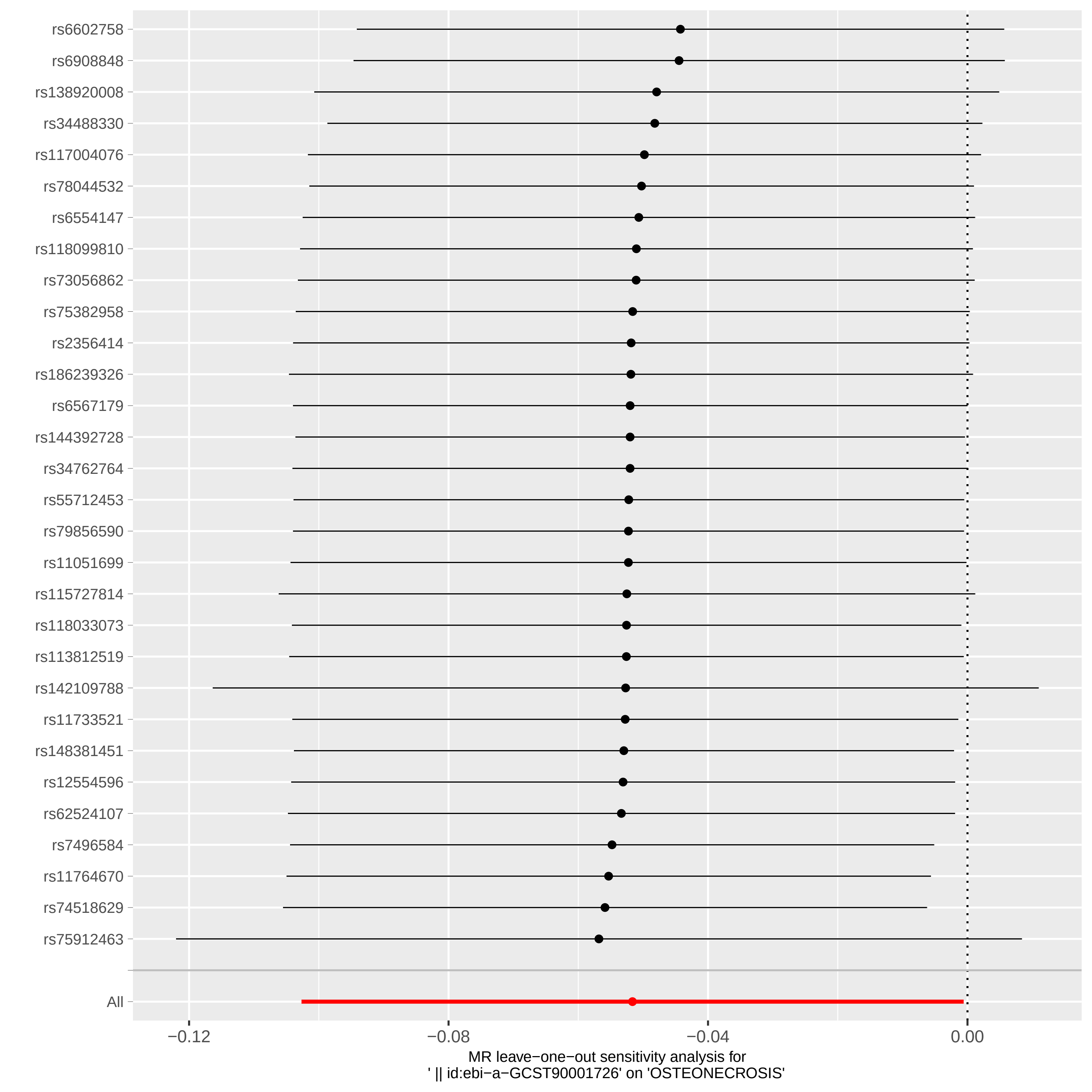
rs6602758
rs6908848
rs138920008
rs34488330
rs117004076
rs78044532
rs6554147
rs118099810
rs73056862
rs75382958
rs2356414
rs186239326
rs6567179
rs144392728
rs34762764
rs55712453
rs79856590
rs11051699
rs115727814
rs118033073
rs113812519
rs142109788
rs11733521
rs148381451
rs12554596
rs62524107
rs7496584
rs11764670
rs74518629
rs75912463
All
−0.12
−0.08
−0.04
0.00
MR leave−one−out sensitivity analysis for
' || id:ebi−a−GCST90001726' on 'OSTEONECROSIS'

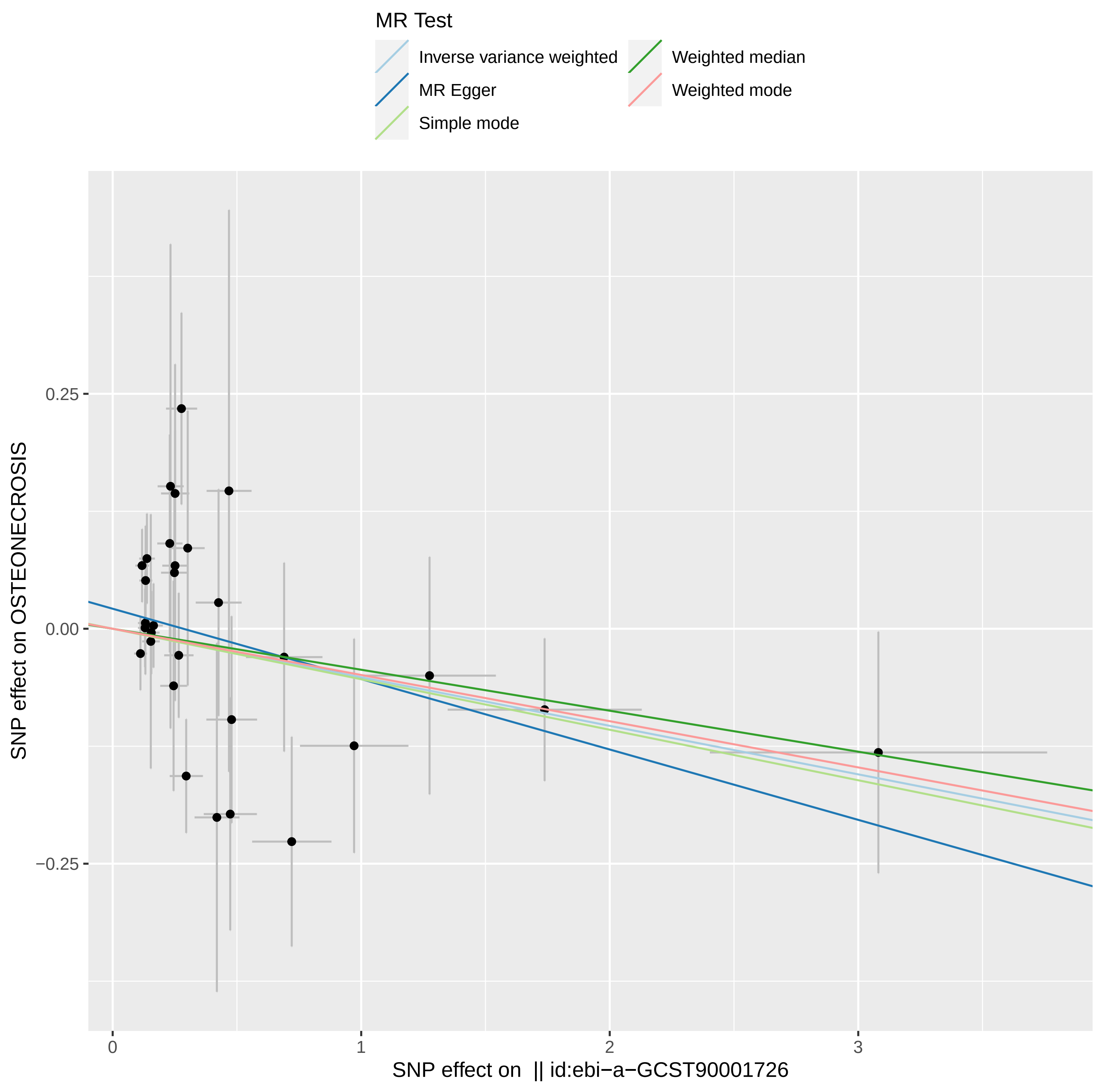
MR Test
Inverse variance weighted
MR Egger
Simple mode
Weighted median
Weighted mode
SNP effect on OSTEONECROSIS
0.25
0.00
−0.25
0
1
2
3
SNP effect on || id:ebi−a−GCST90001726

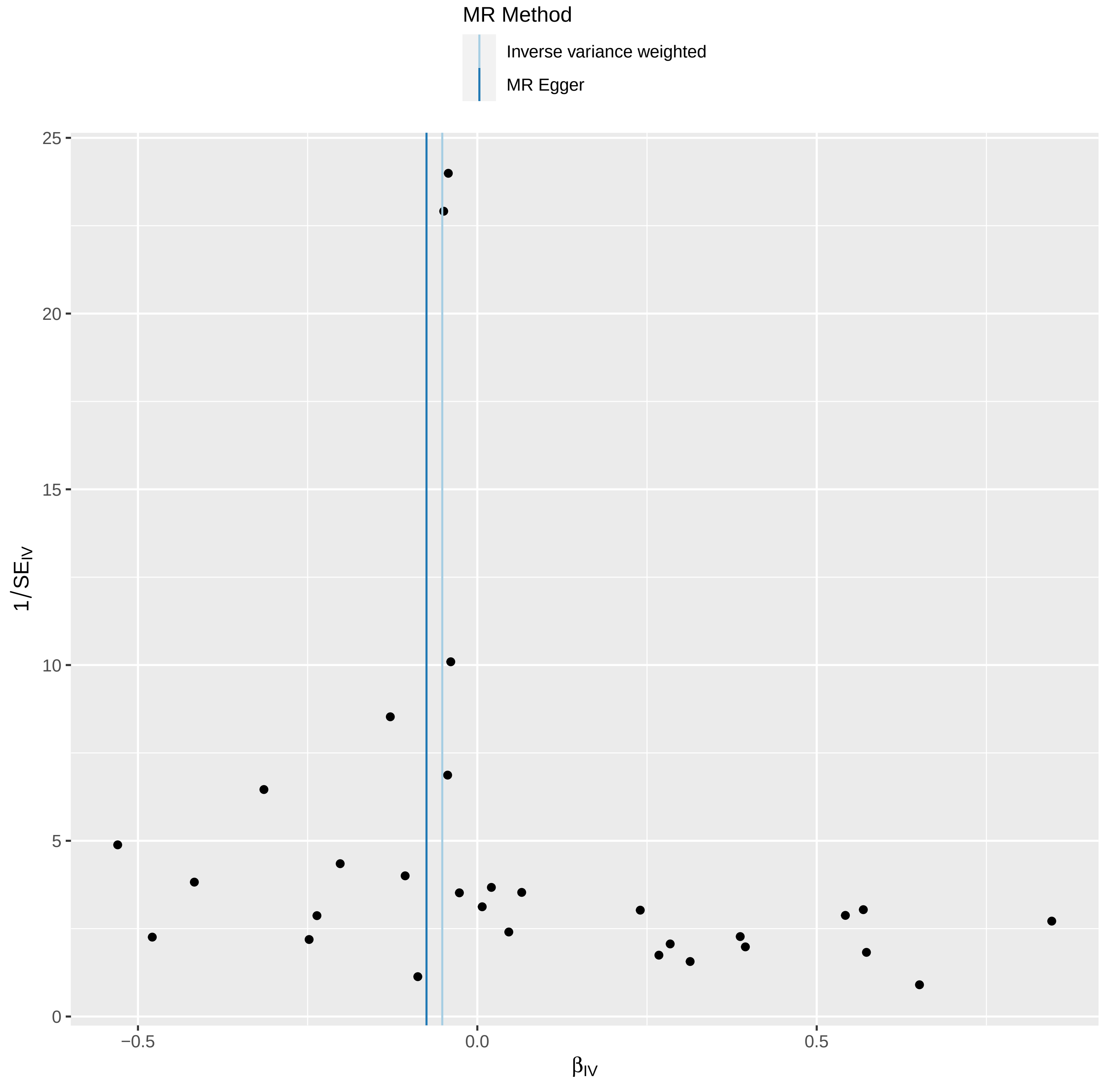

MR Method
Inverse variance weighted
MR Egger
25
20
15
10
5
0
1/SE$_{IV}$
−0.5
0.0
0.5
β$_{IV}$

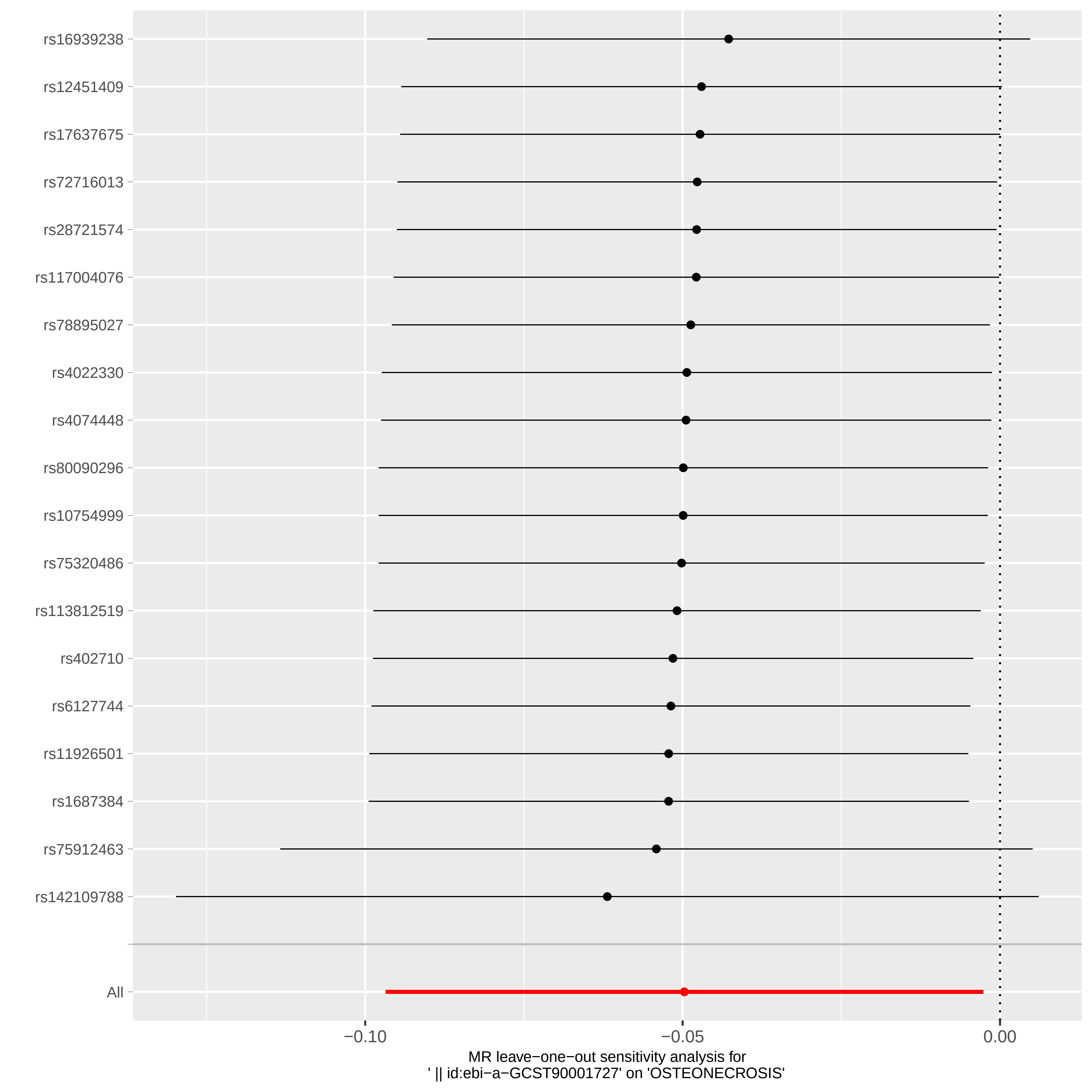
rs16939238
rs12451409
rs17637675
rs72716013
rs28721574
rs117004076
rs78895027
rs4022330
rs4074448
rs80090296
rs10754999
rs75320486
rs113812519
rs402710
rs6127744
rs11926501
rs1687384
rs75912463
rs142109788
All
−0.10
−0.05
0.00
MR leave−one−out sensitivity analysis for
' || id:ebi−a−GCST90001727' on 'OSTEONECROSIS'

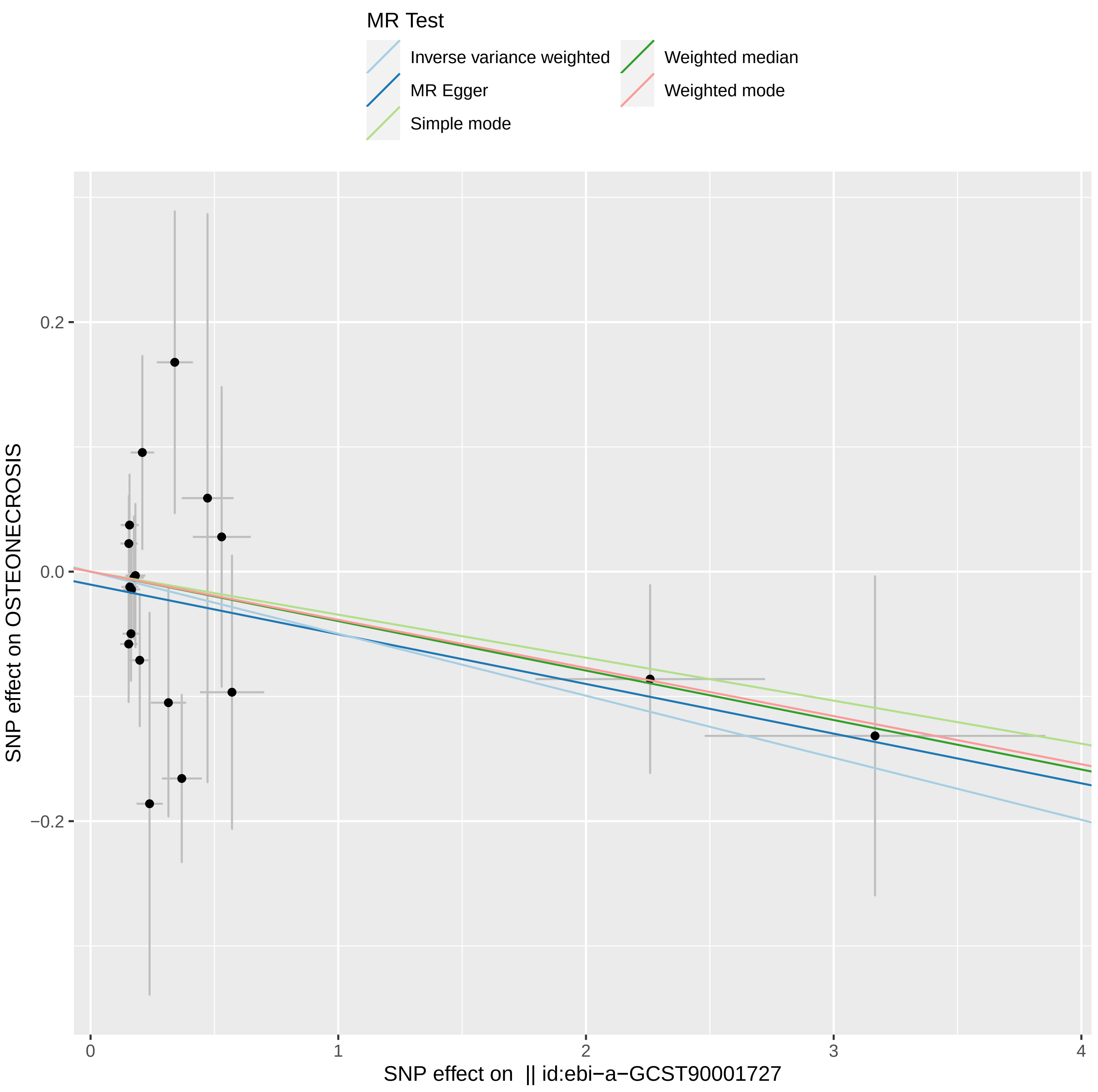
MR Test
Inverse variance weighted
MR Egger
Simple mode
Weighted median
Weighted mode
0.2
0.0
−0.2
SNP effect on OSTEONECROSIS
0
1
2
3
4
SNP effect on || id:ebi−a−GCST90001727

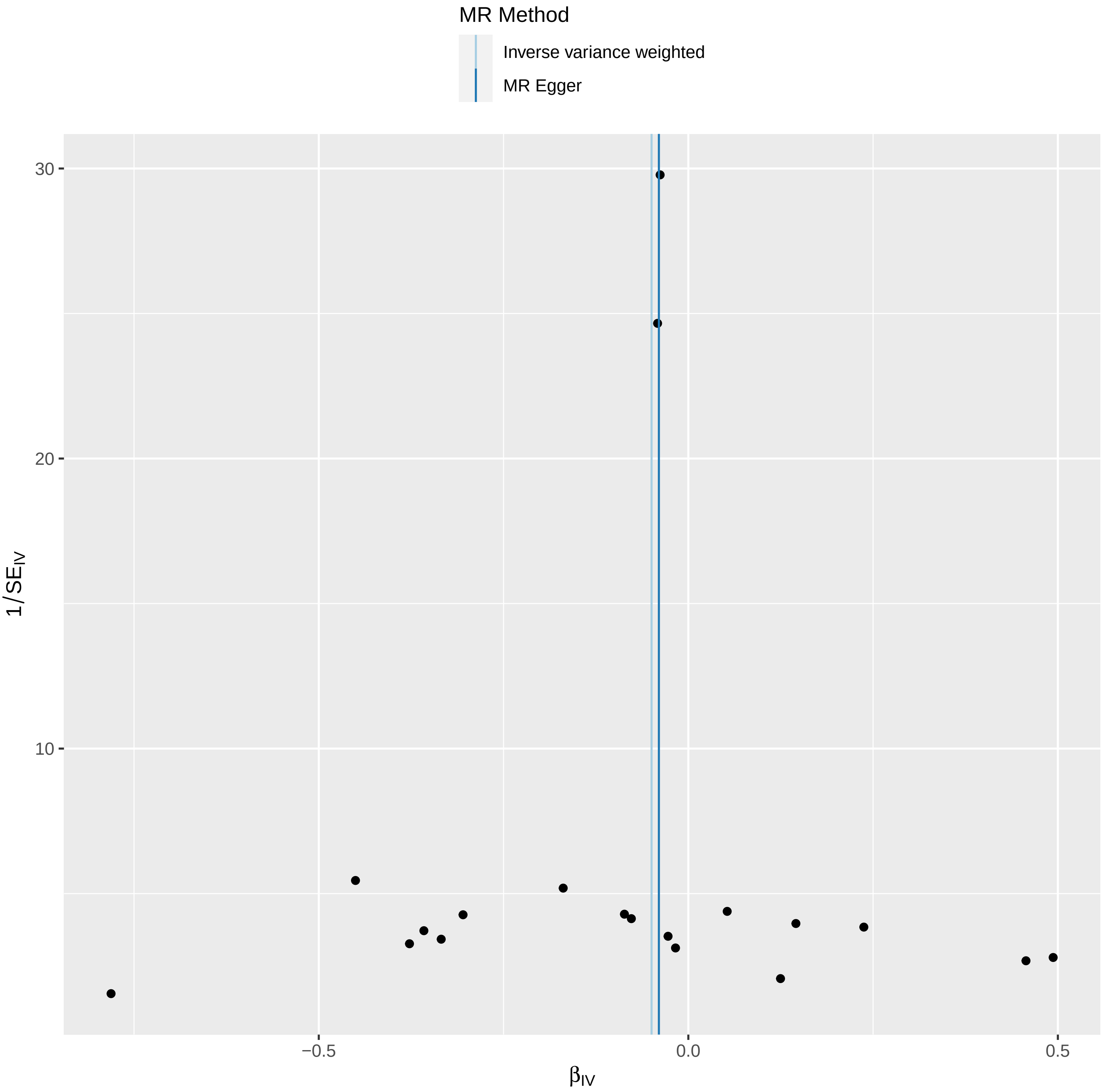
MR Method
Inverse variance weighted
MR Egger
30
20
10
1/SE_IV
−0.5
0.0
0.5
β_IV

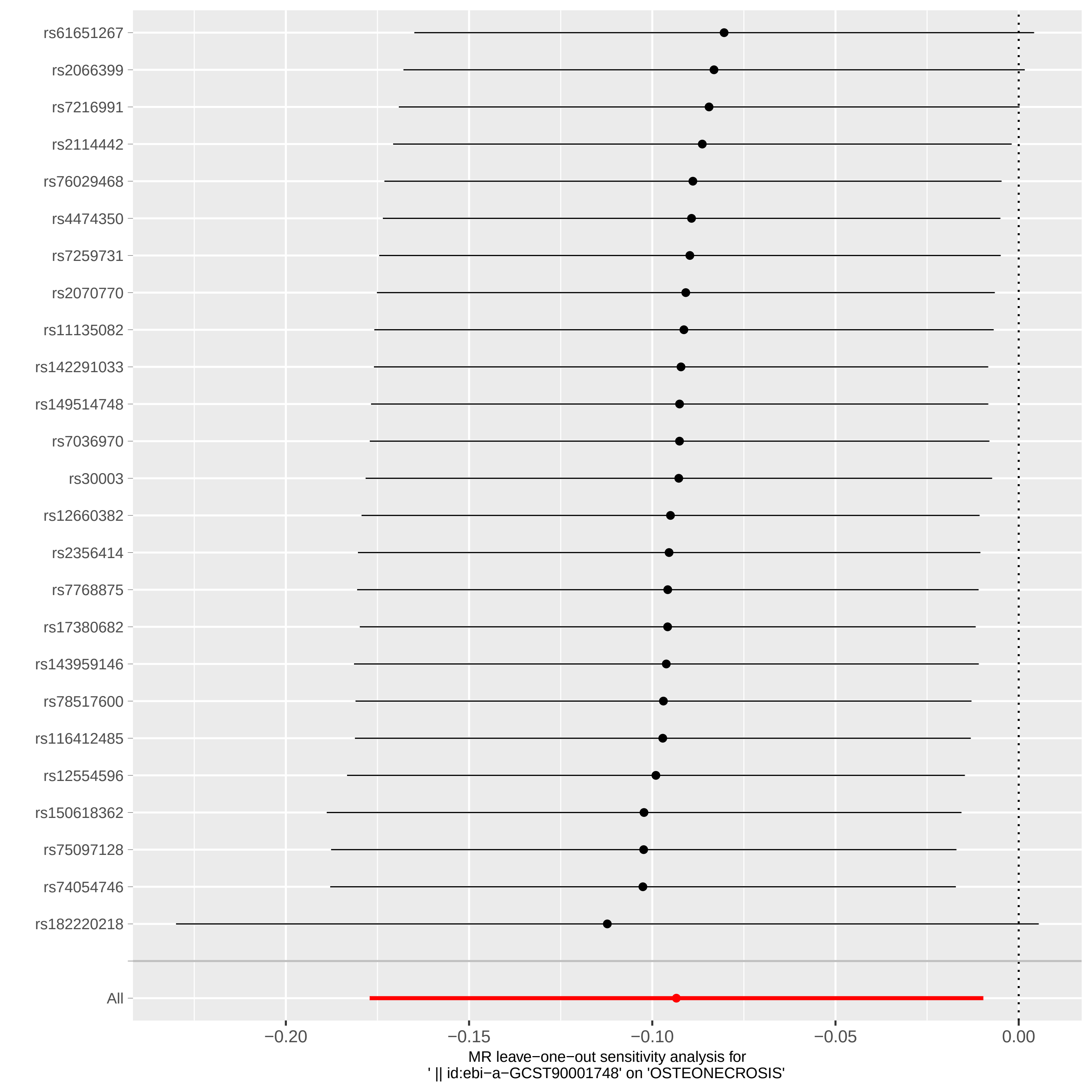

rs61651267
rs2066399
rs7216991
rs2114442
rs76029468
rs4474350
rs7259731
rs2070770
rs11135082
rs142291033
rs149514748
rs7036970
rs30003
rs12660382
rs2356414
rs7768875
rs17380682
rs143959146
rs78517600
rs116412485
rs12554596
rs150618362
rs75097128
rs74054746
rs182220218
All
−0.20
−0.15
−0.10
−0.05
0.00
MR leave−one−out sensitivity analysis for
' || id:ebi−a−GCST90001748' on 'OSTEONECROSIS'

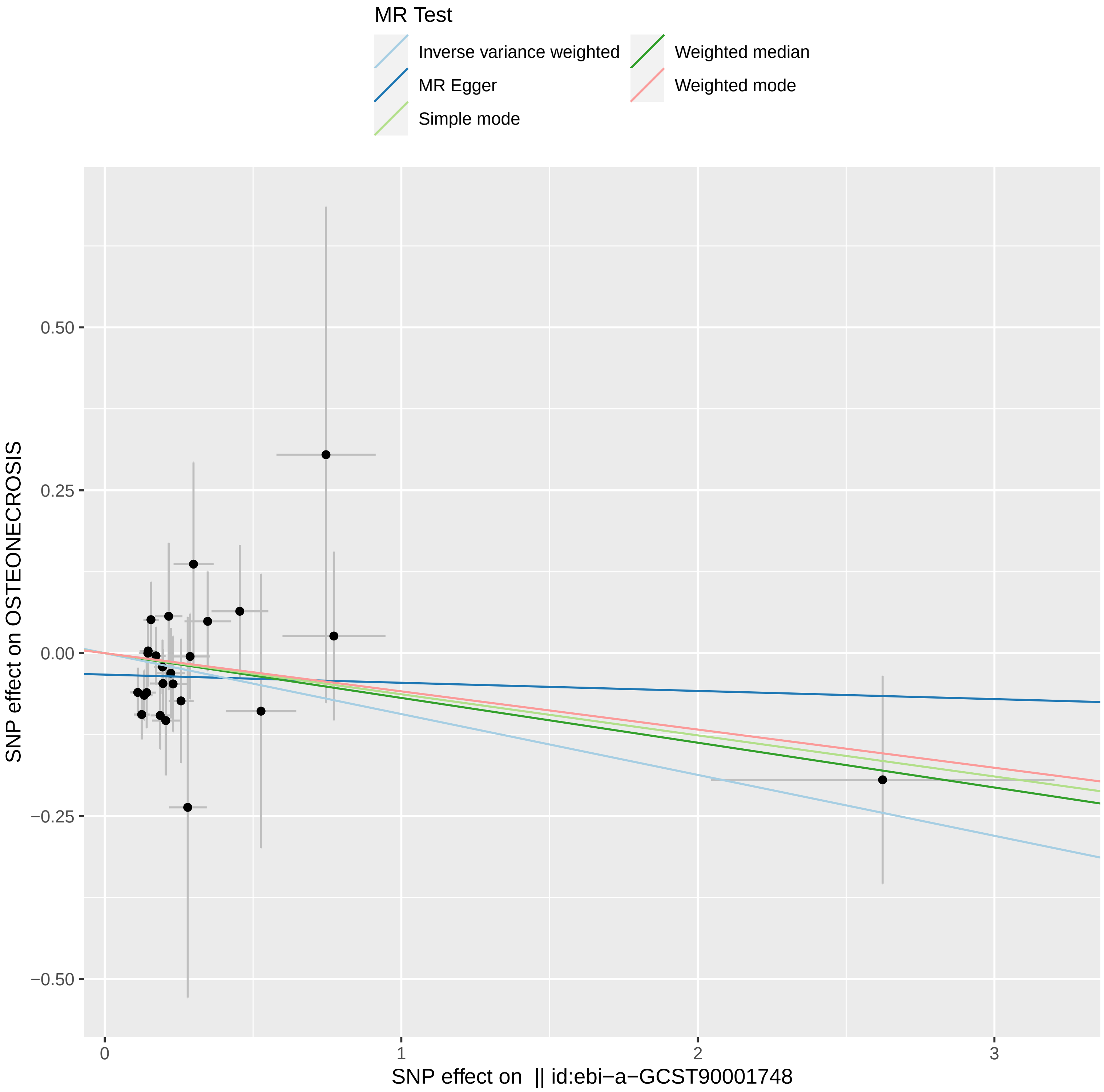
MR Test
Inverse variance weighted
MR Egger
Simple mode
Weighted median
Weighted mode
0.50
0.25
0.00
−0.25
−0.50
0
1
2
3
SNP effect on OSTEONECROSIS
SNP effect on || id:ebi−a−GCST90001748

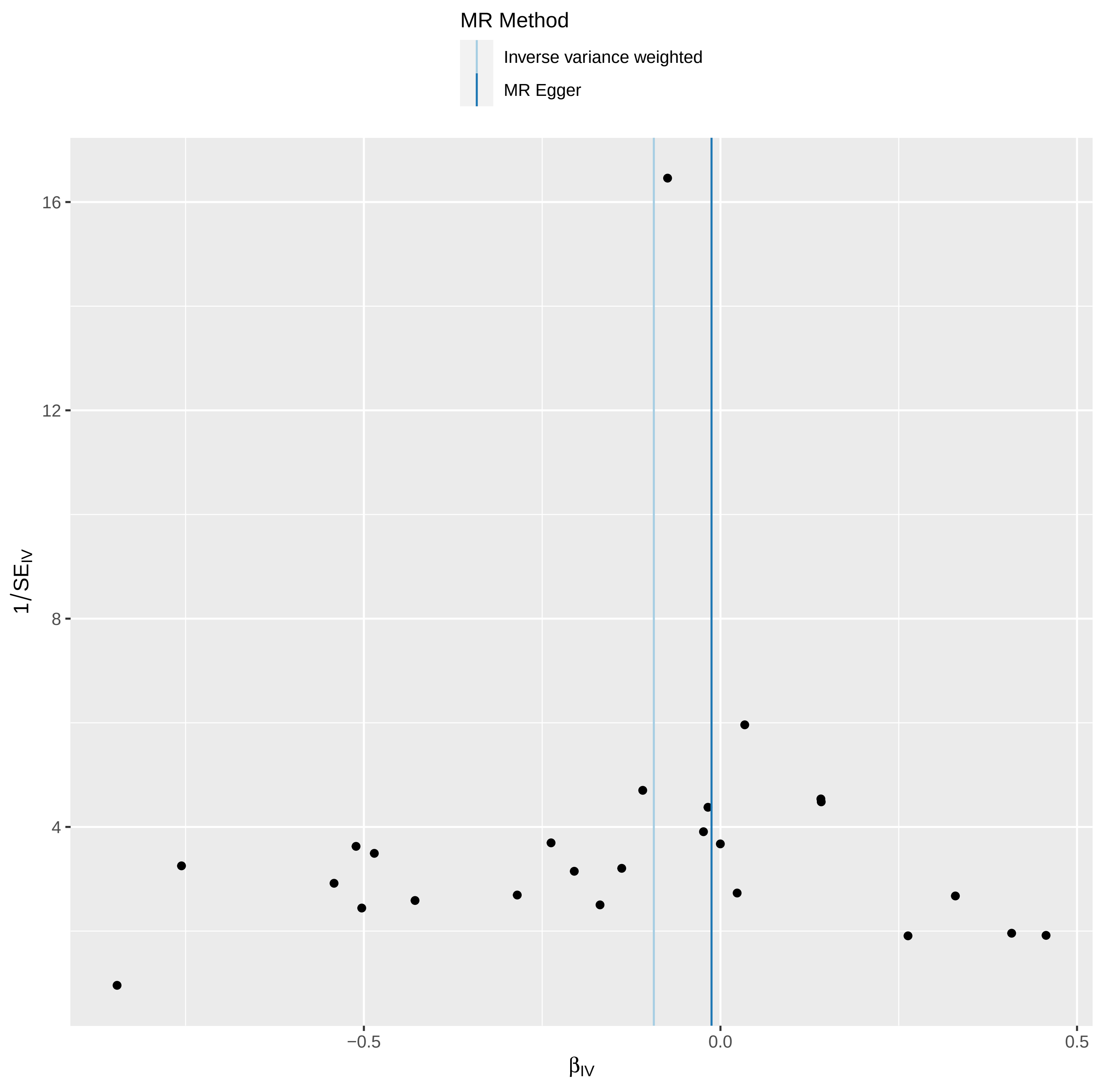

MR Method
Inverse variance weighted
MR Egger
16
12
8
4
1/SE$_{IV}$
−0.5
0.0
0.5
β$_{IV}$

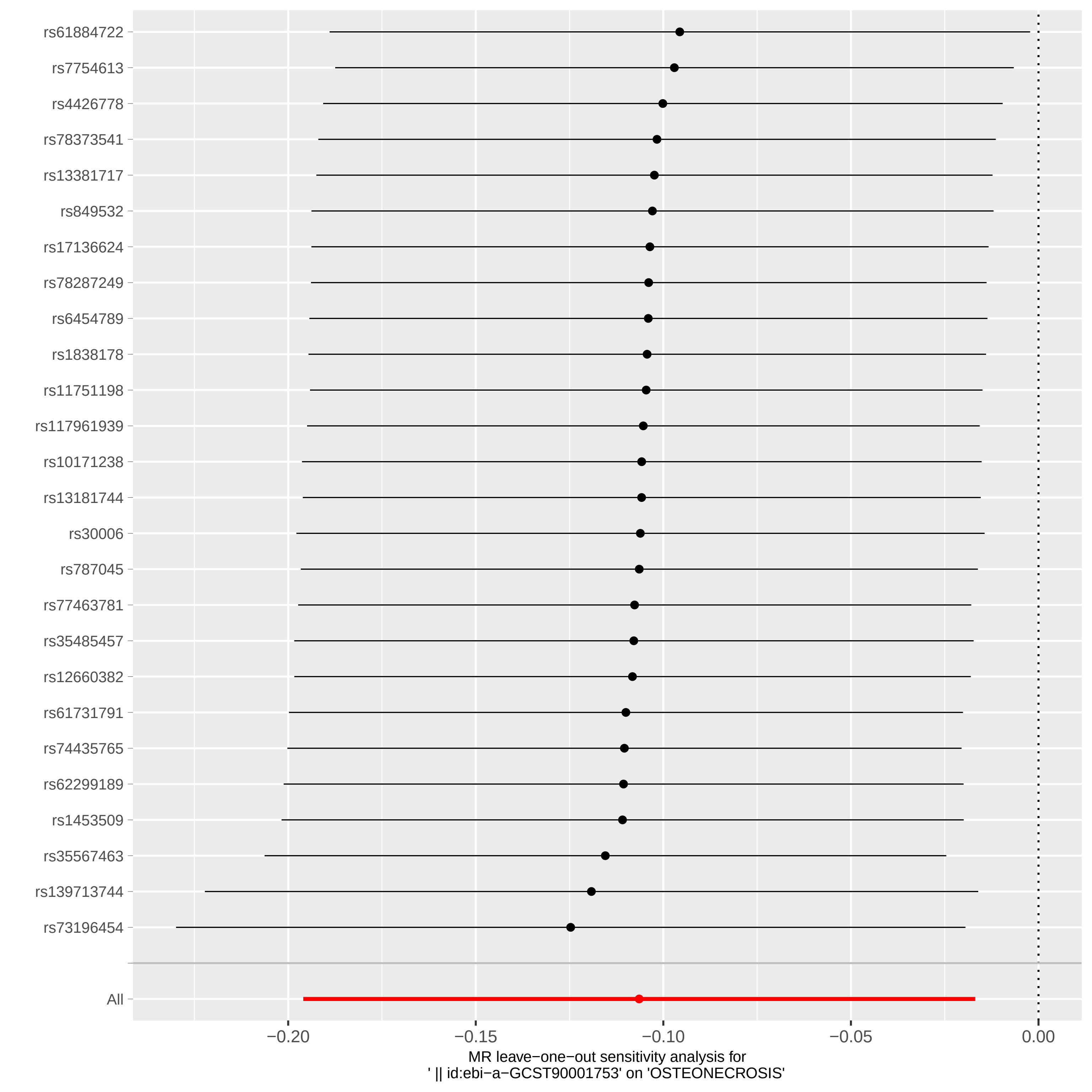
rs61884722
rs7754613
rs4426778
rs78373541
rs13381717
rs849532
rs17136624
rs78287249
rs6454789
rs1838178
rs11751198
rs117961939
rs10171238
rs13181744
rs30006
rs787045
rs77463781
rs35485457
rs12660382
rs61731791
rs74435765
rs62299189
rs1453509
rs35567463
rs139713744
rs73196454
All
−0.20
−0.15
−0.10
−0.05
0.00
MR leave−one−out sensitivity analysis for
' || id:ebi−a−GCST90001753' on 'OSTEONECROSIS'

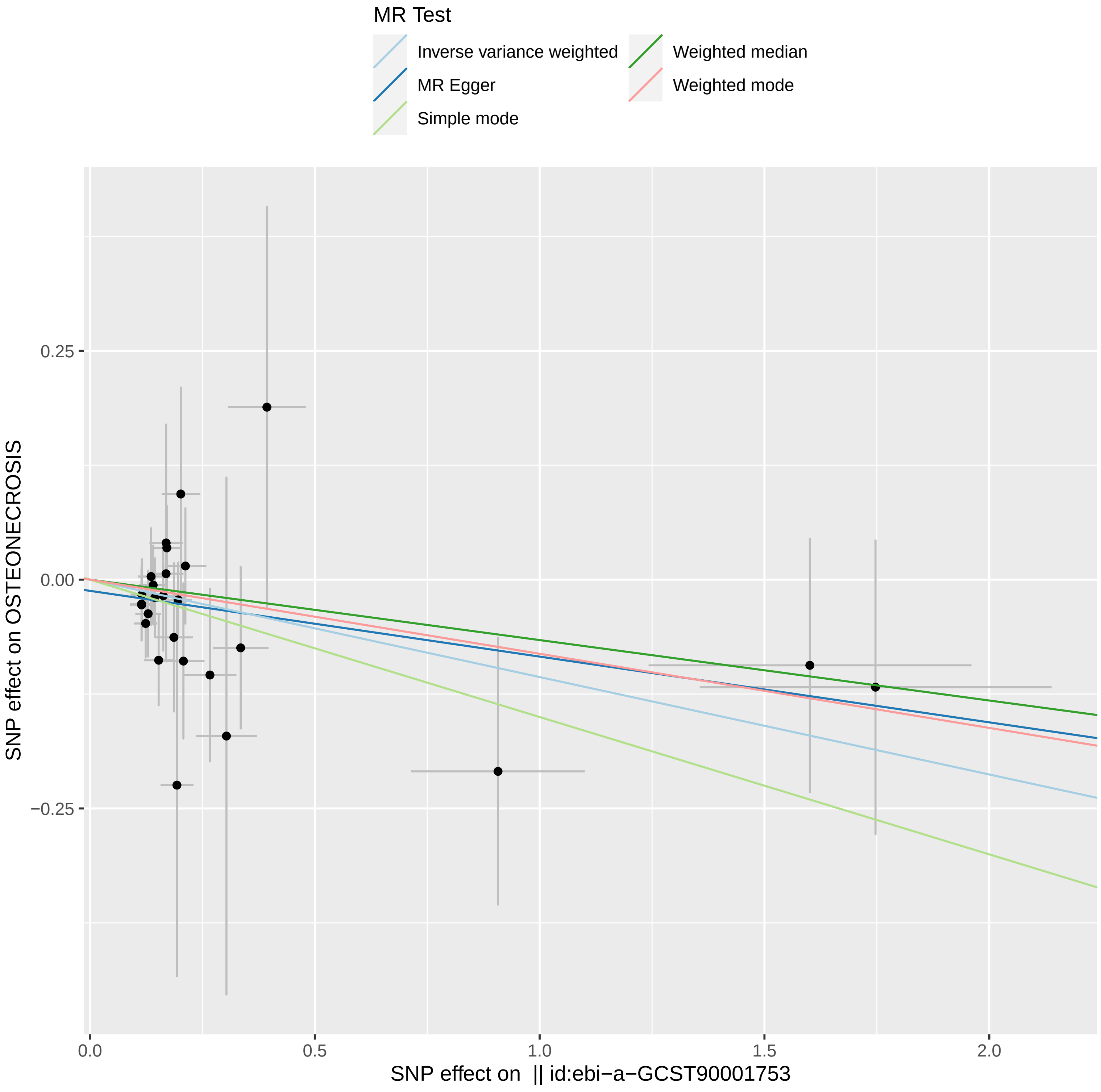
MR Test
Inverse variance weighted
MR Egger
Simple mode
Weighted median
Weighted mode
0.25
0.00
−0.25
SNP effect on OSTEONECROSIS
0.0
0.5
1.0
1.5
2.0
SNP effect on || id:ebi−a−GCST90001753

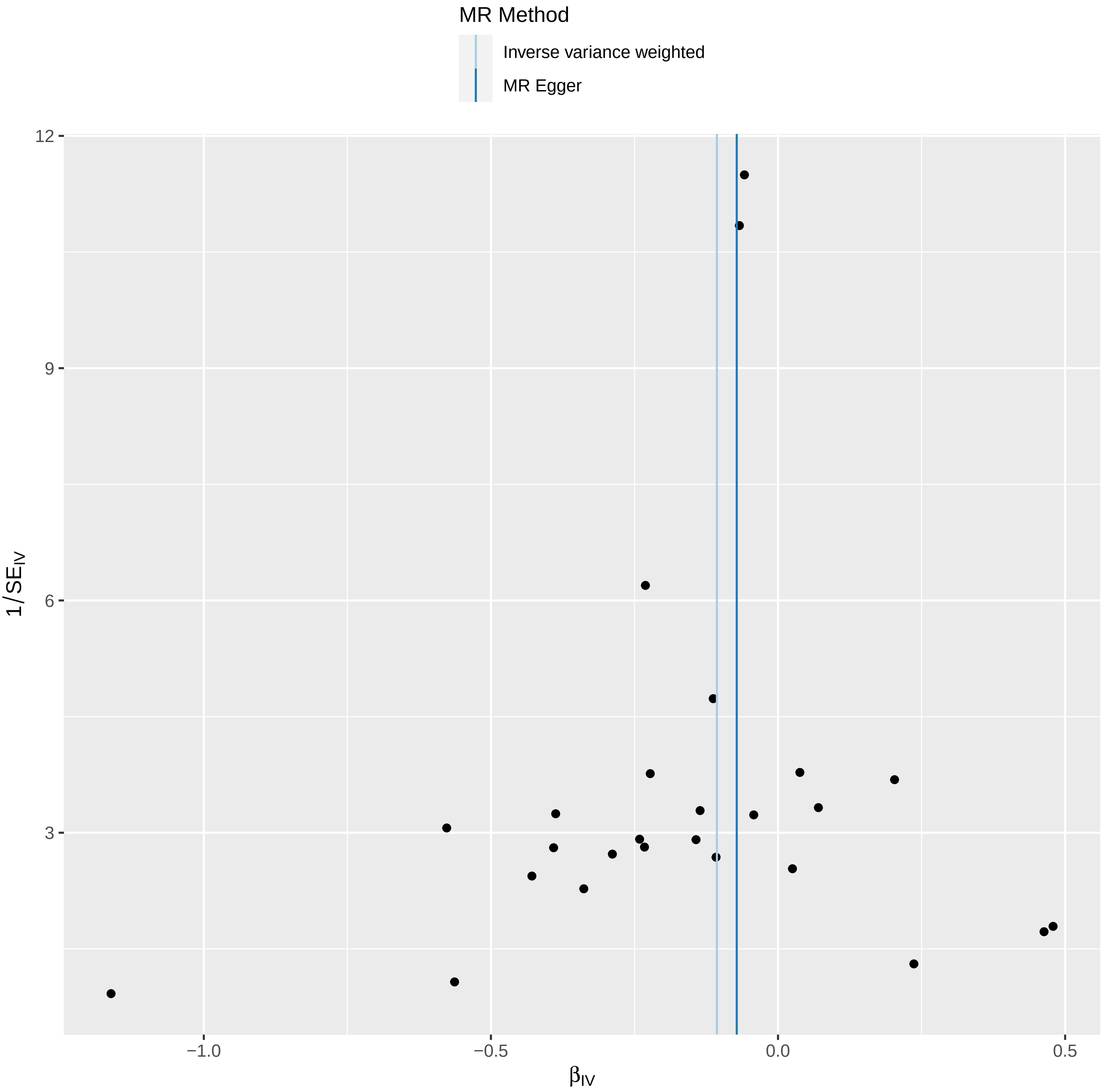

MR Method
Inverse variance weighted
MR Egger
12
9
6
3
1/SE$_{IV}$
−1.0
−0.5
0.0
0.5
β$_{IV}$

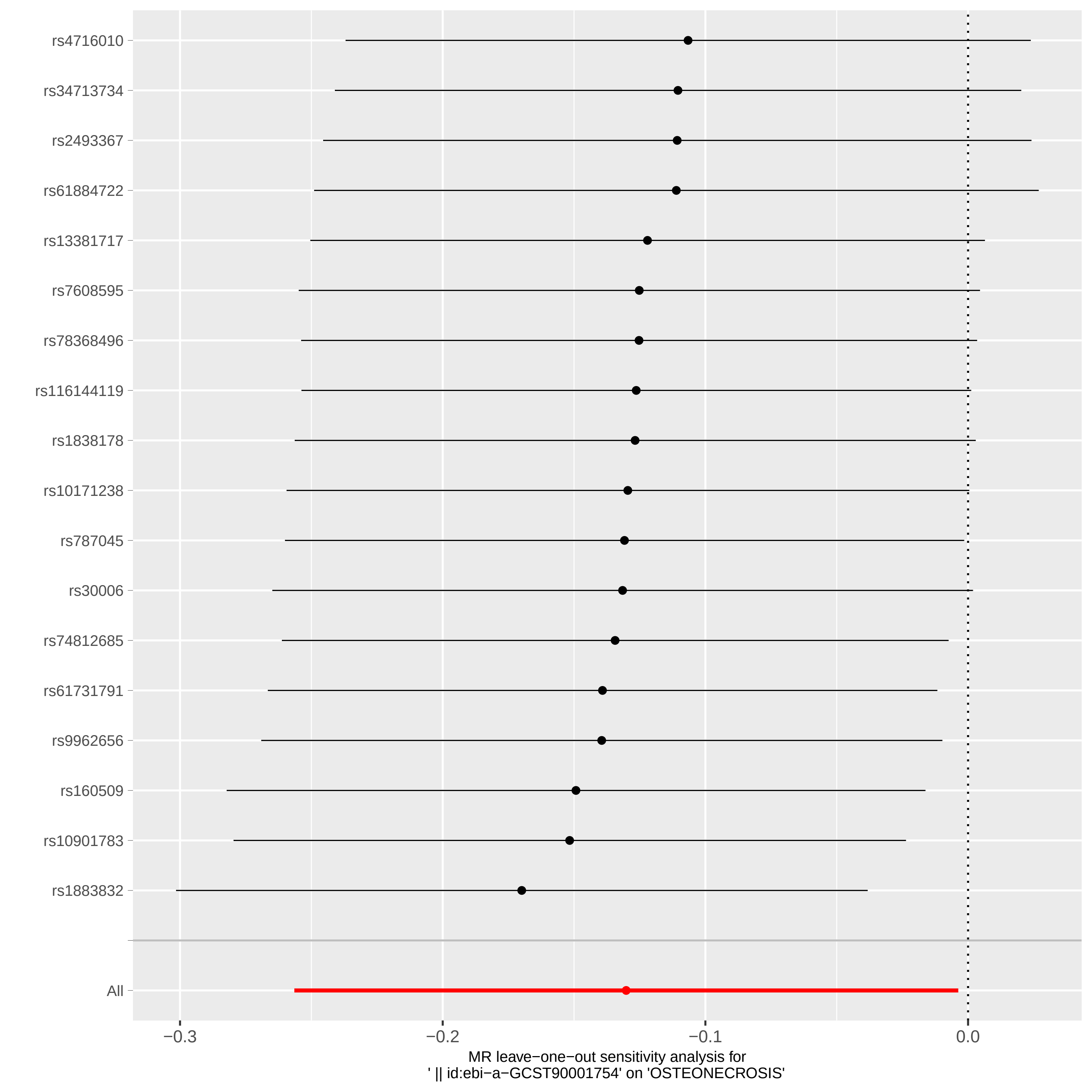
rs4716010
rs34713734
rs2493367
rs61884722
rs13381717
rs7608595
rs78368496
rs116144119
rs1838178
rs10171238
rs787045
rs30006
rs74812685
rs61731791
rs9962656
rs160509
rs10901783
rs1883832
All
−0.3
−0.2
−0.1
0.0
MR leave−one−out sensitivity analysis for
' || id:ebi−a−GCST90001754' on 'OSTEONECROSIS'

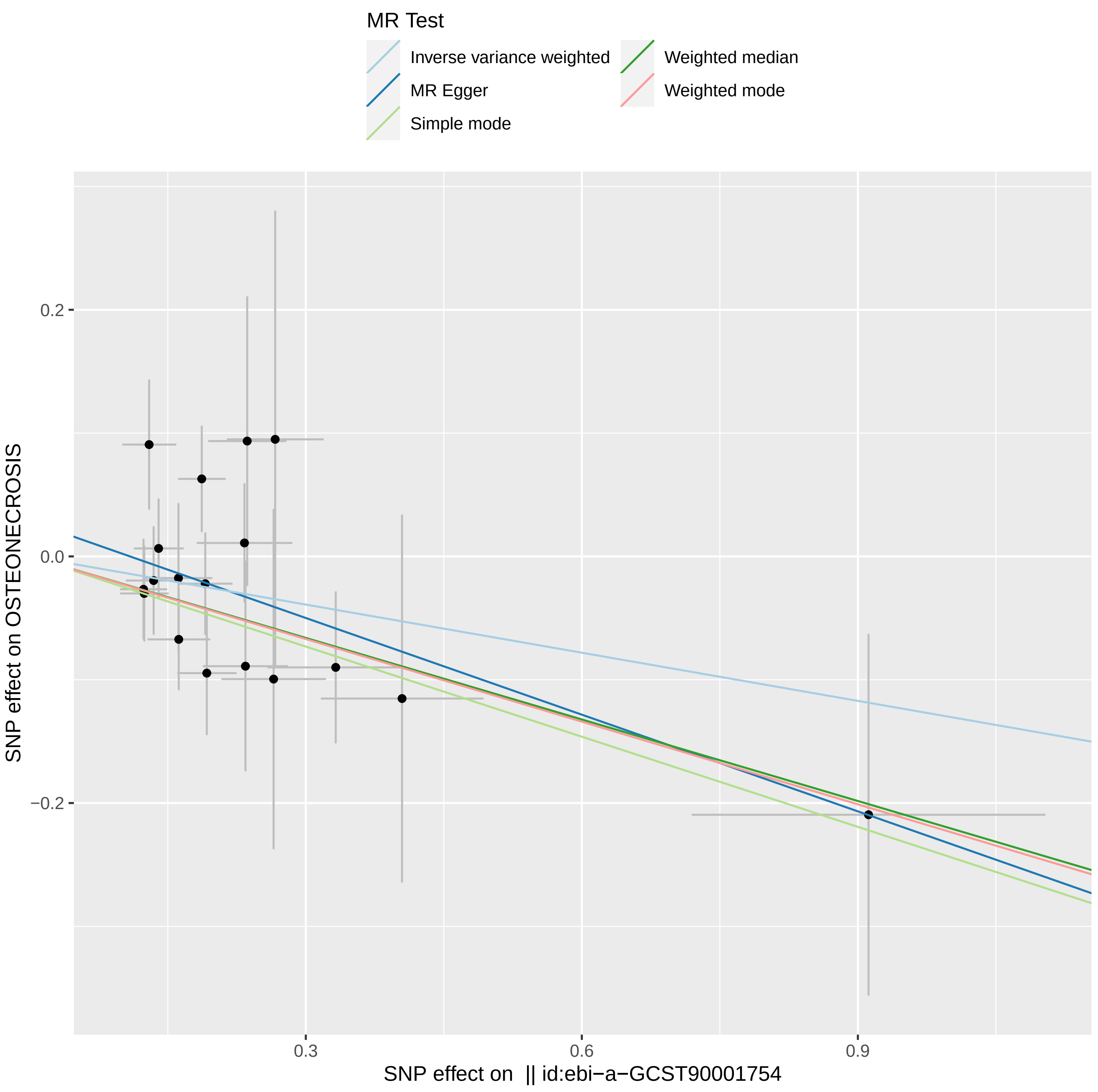

MR Test
Inverse variance weighted
MR Egger
Simple mode
Weighted median
Weighted mode
0.2
0.0
−0.2
SNP effect on OSTEONECROSIS
0.3
0.6
0.9
SNP effect on || id:ebi−a−GCST90001754

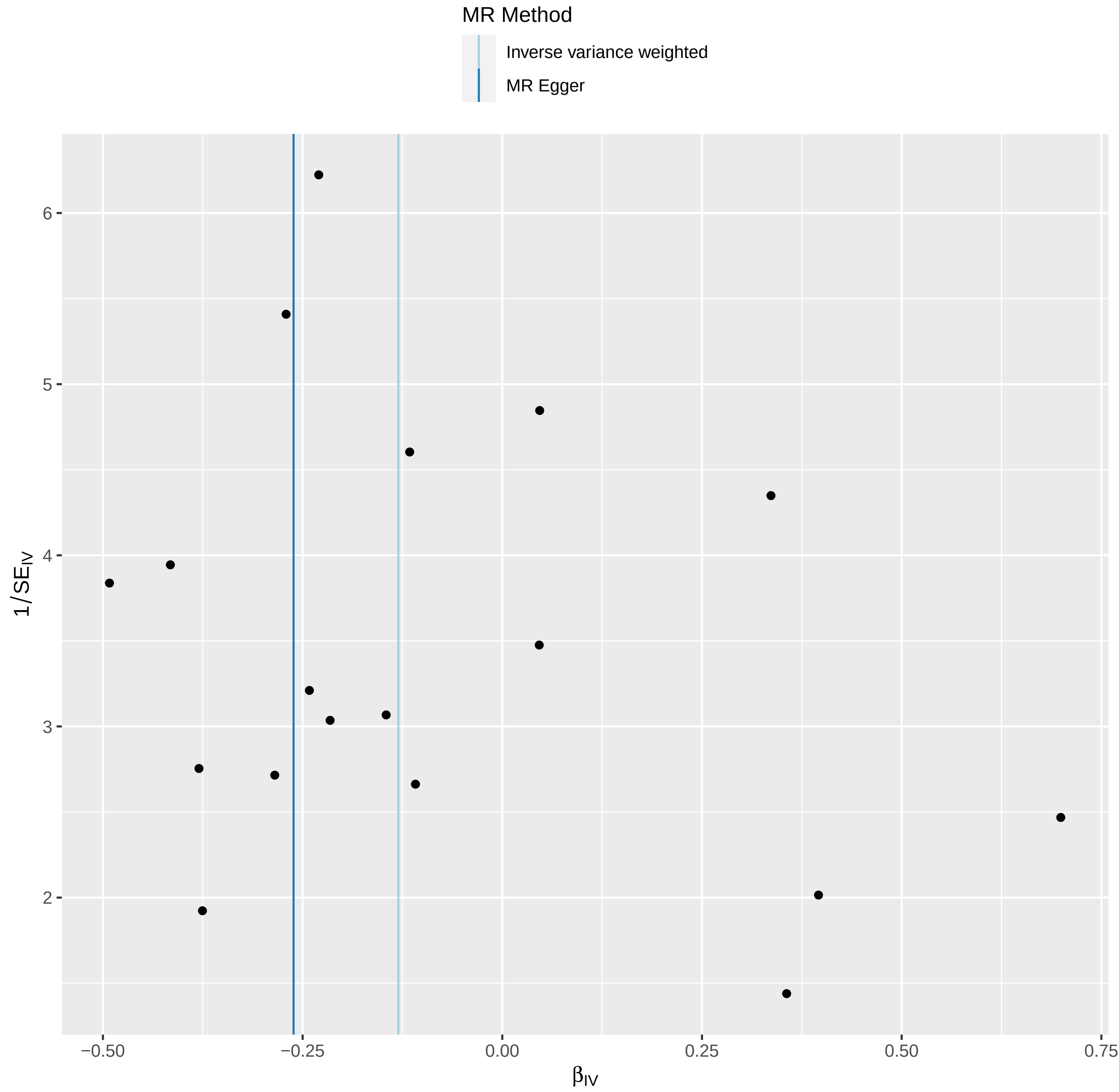

MR Method
Inverse variance weighted
MR Egger
6
5
4
3
2
1/SE_IV
−0.50
−0.25
0.00
0.25
0.50
0.75
β_IV

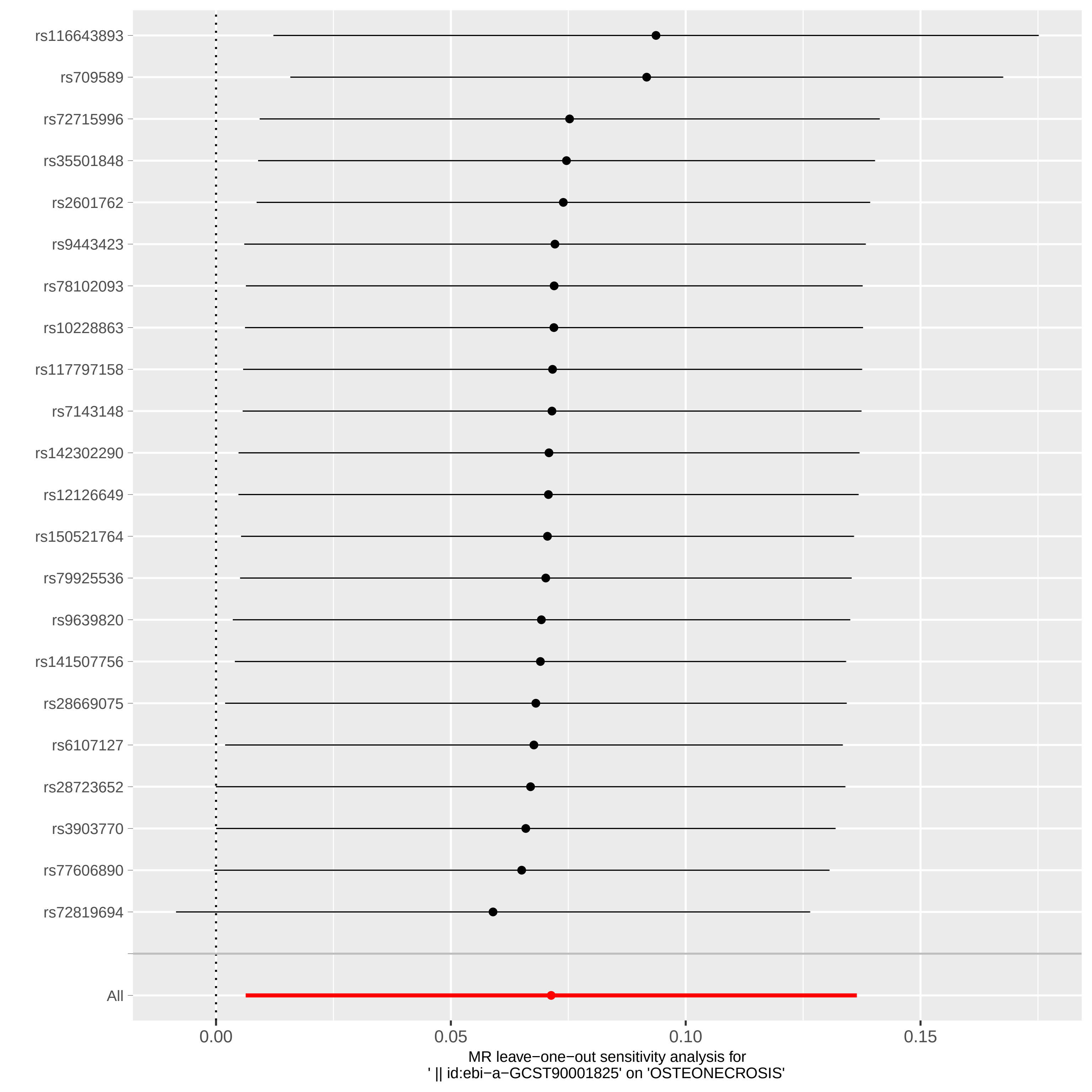
rs116643893
rs709589
rs72715996
rs35501848
rs2601762
rs9443423
rs78102093
rs10228863
rs117797158
rs7143148
rs142302290
rs12126649
rs150521764
rs79925536
rs9639820
rs141507756
rs28669075
rs6107127
rs28723652
rs3903770
rs77606890
rs72819694
All
0.00
0.05
0.10
0.15
MR leave−one−out sensitivity analysis for
' || id:ebi−a−GCST90001825' on 'OSTEONECROSIS'

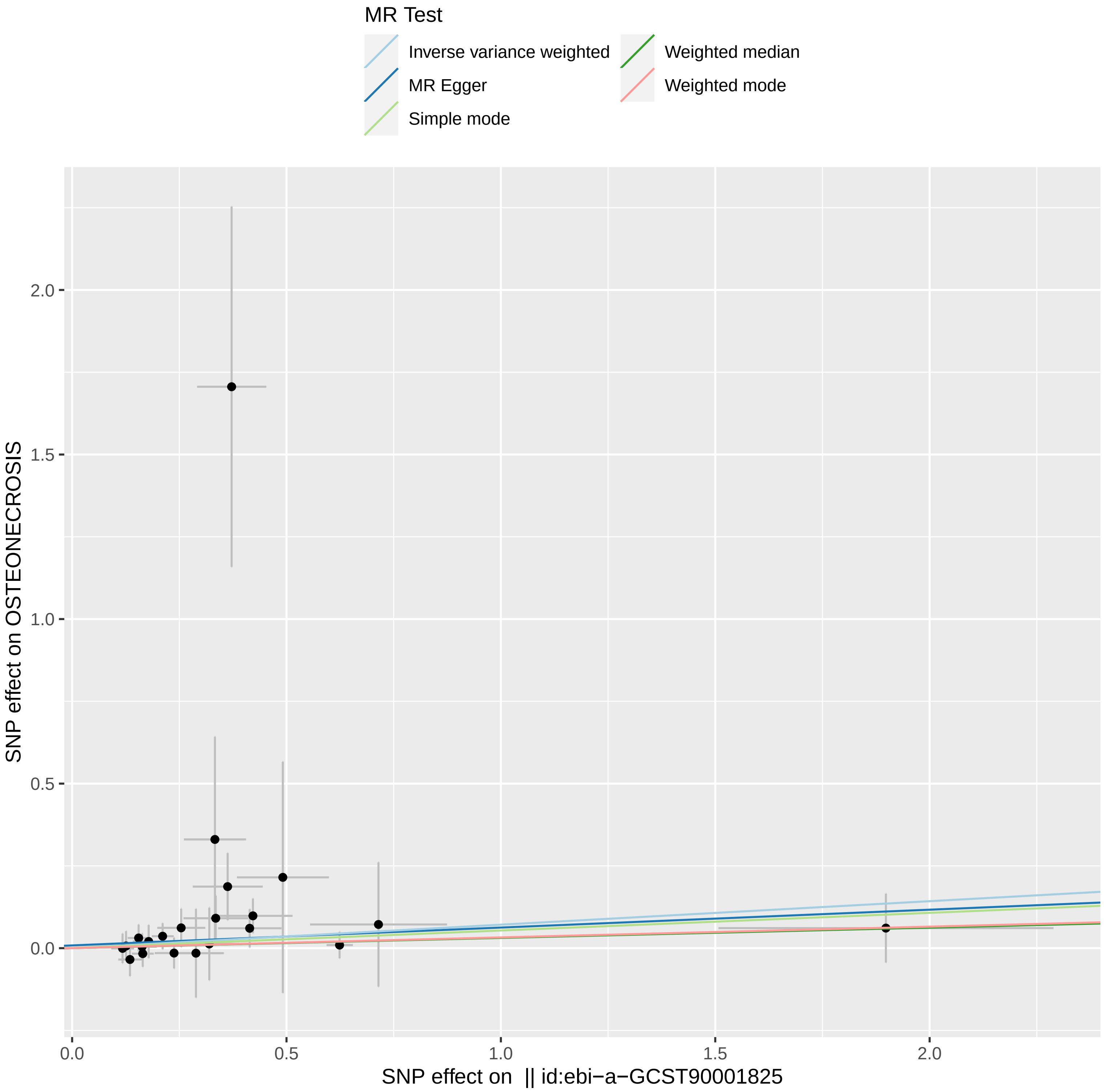

MR Test
Inverse variance weighted
MR Egger
Simple mode
Weighted median
Weighted mode
SNP effect on OSTEONECROSIS
2.0
1.5
1.0
0.5
0.0
0.0
0.5
1.0
1.5
2.0
SNP effect on || id:ebi−a−GCST90001825

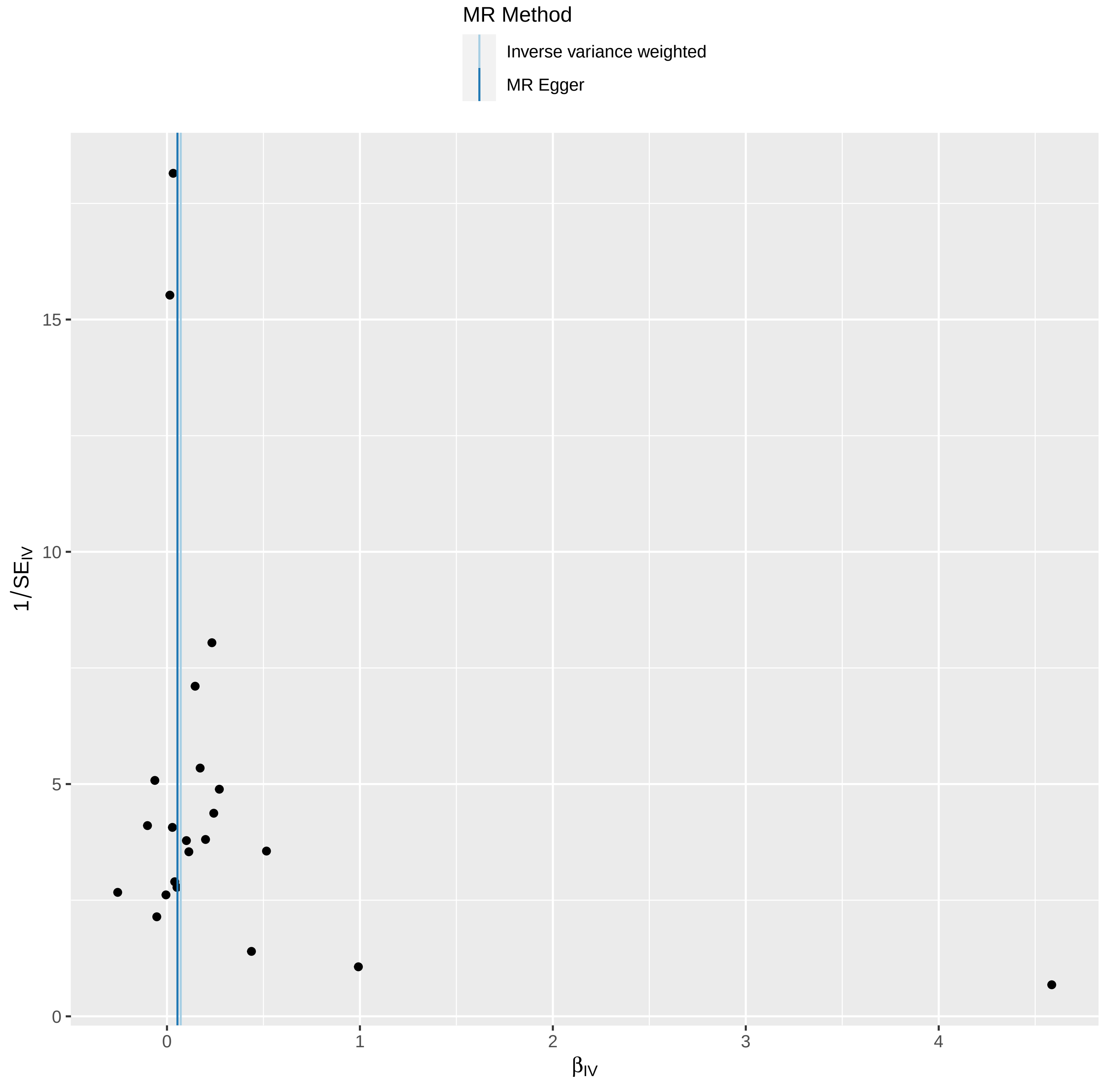
MR Method
Inverse variance weighted
MR Egger
15
10
5
0
1/SE_IV
0
1
2
3
4
β_IV

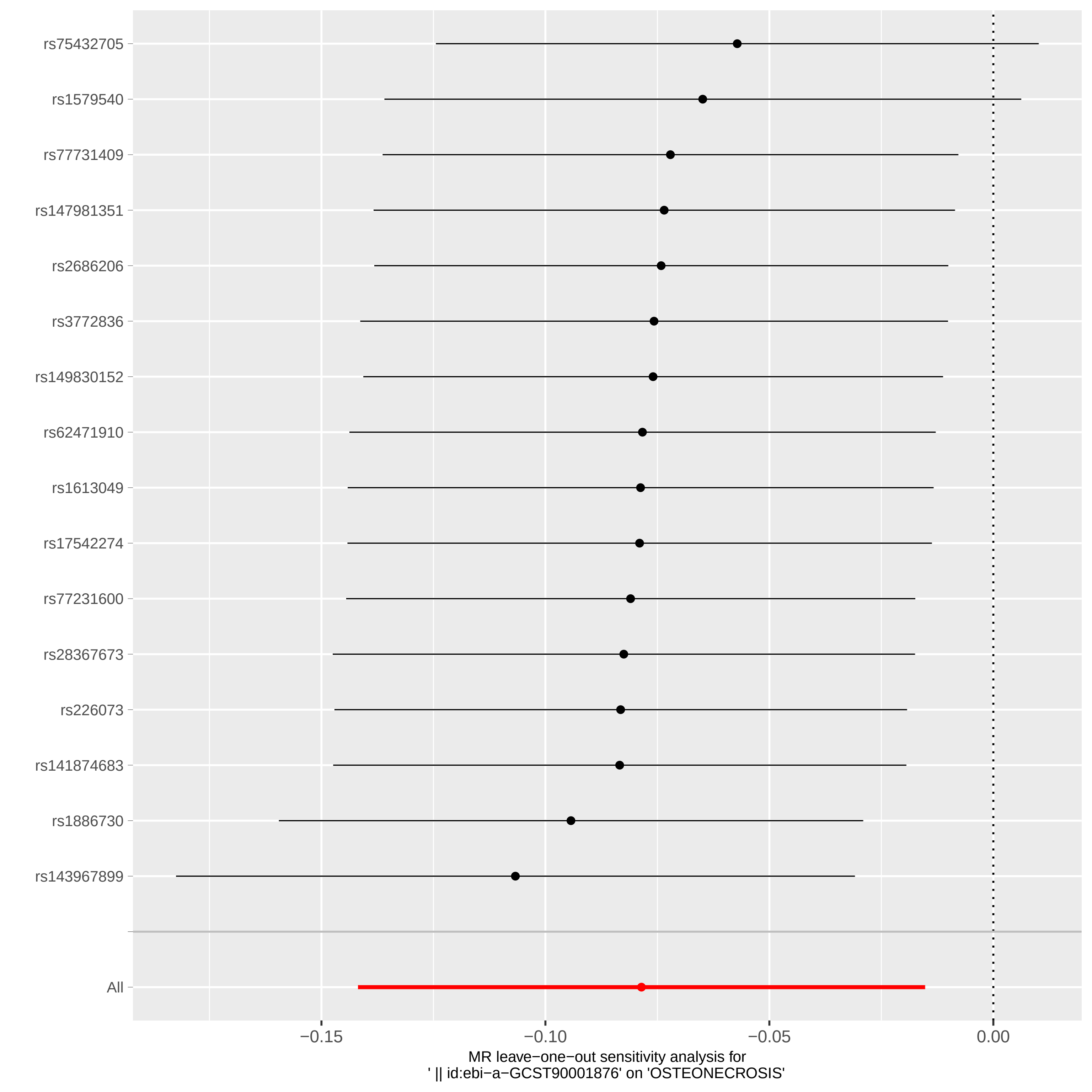
rs75432705
rs1579540
rs77731409
rs147981351
rs2686206
rs3772836
rs149830152
rs62471910
rs1613049
rs17542274
rs77231600
rs28367673
rs226073
rs141874683
rs1886730
rs143967899
All
−0.15
−0.10
−0.05
0.00
MR leave−one−out sensitivity analysis for
' || id:ebi−a−GCST90001876' on 'OSTEONECROSIS'

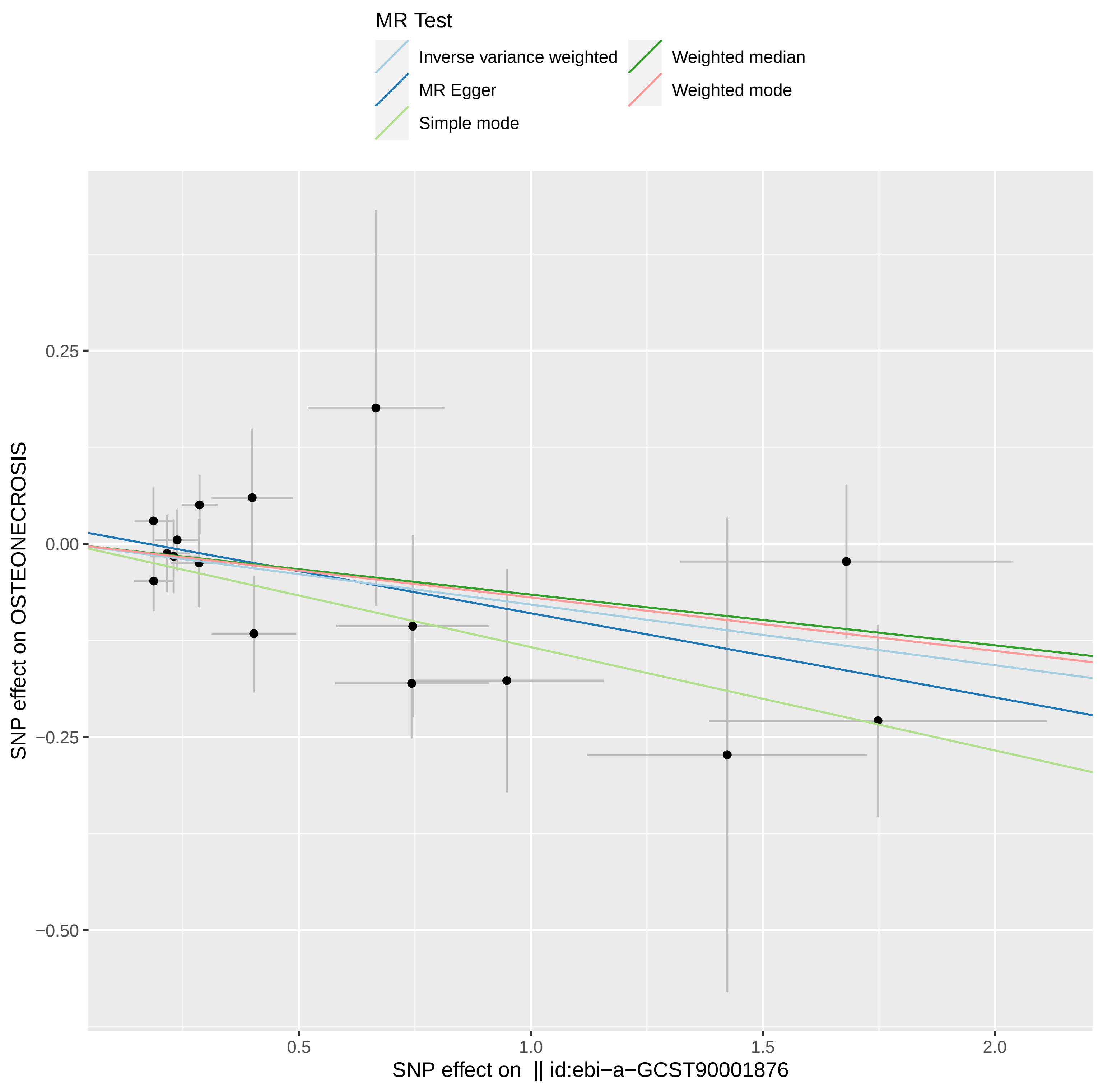

MR Test
Inverse variance weighted
MR Egger
Simple mode
Weighted median
Weighted mode
0.25
0.00
−0.25
−0.50
0.5
1.0
1.5
2.0
SNP effect on OSTEONECROSIS
SNP effect on || id:ebi−a−GCST90001876

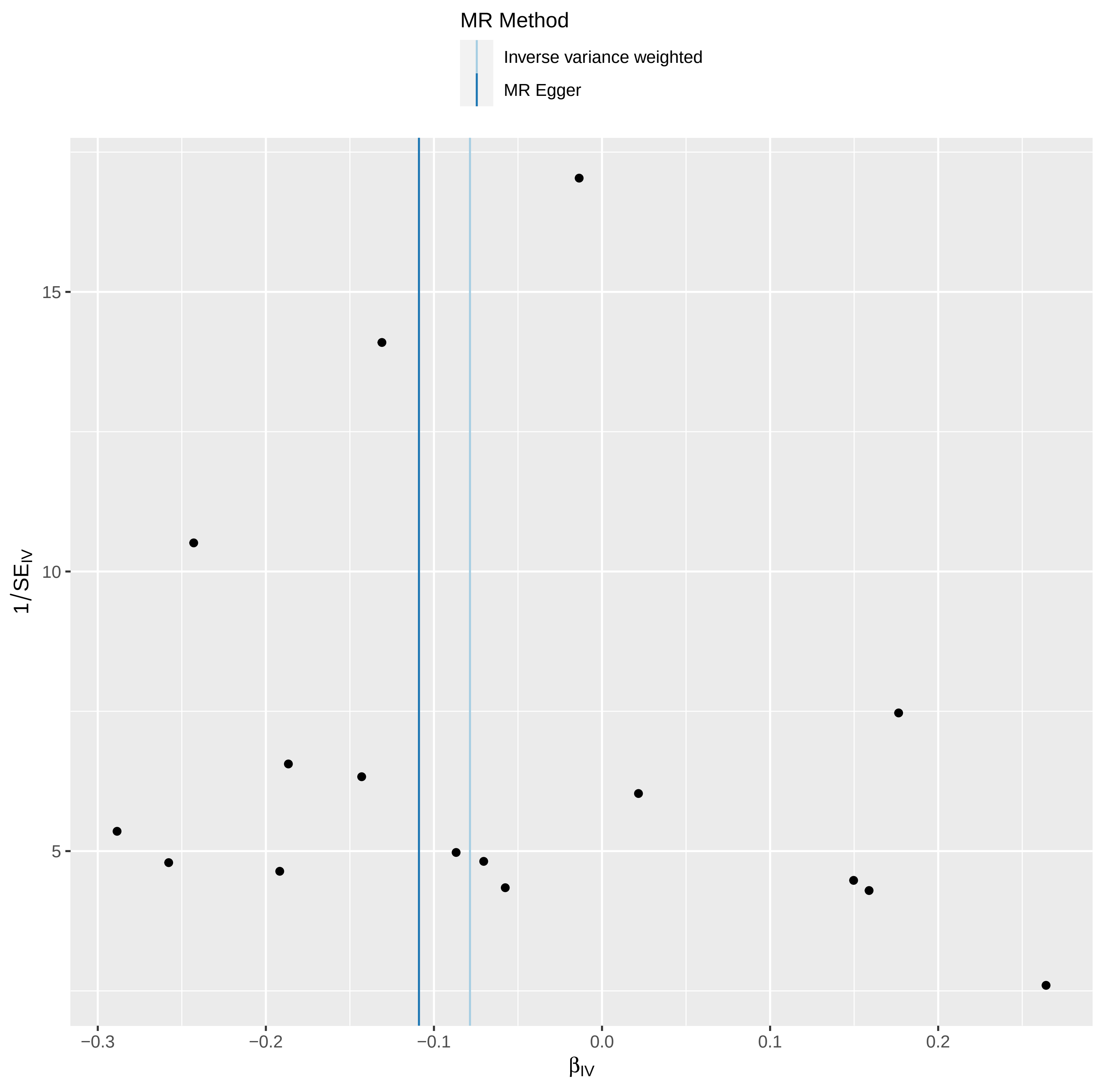
MR Method
Inverse variance weighted
MR Egger
15
10
5
1/SE_IV
−0.3
−0.2
−0.1
0.0
0.1
0.2
β_IV

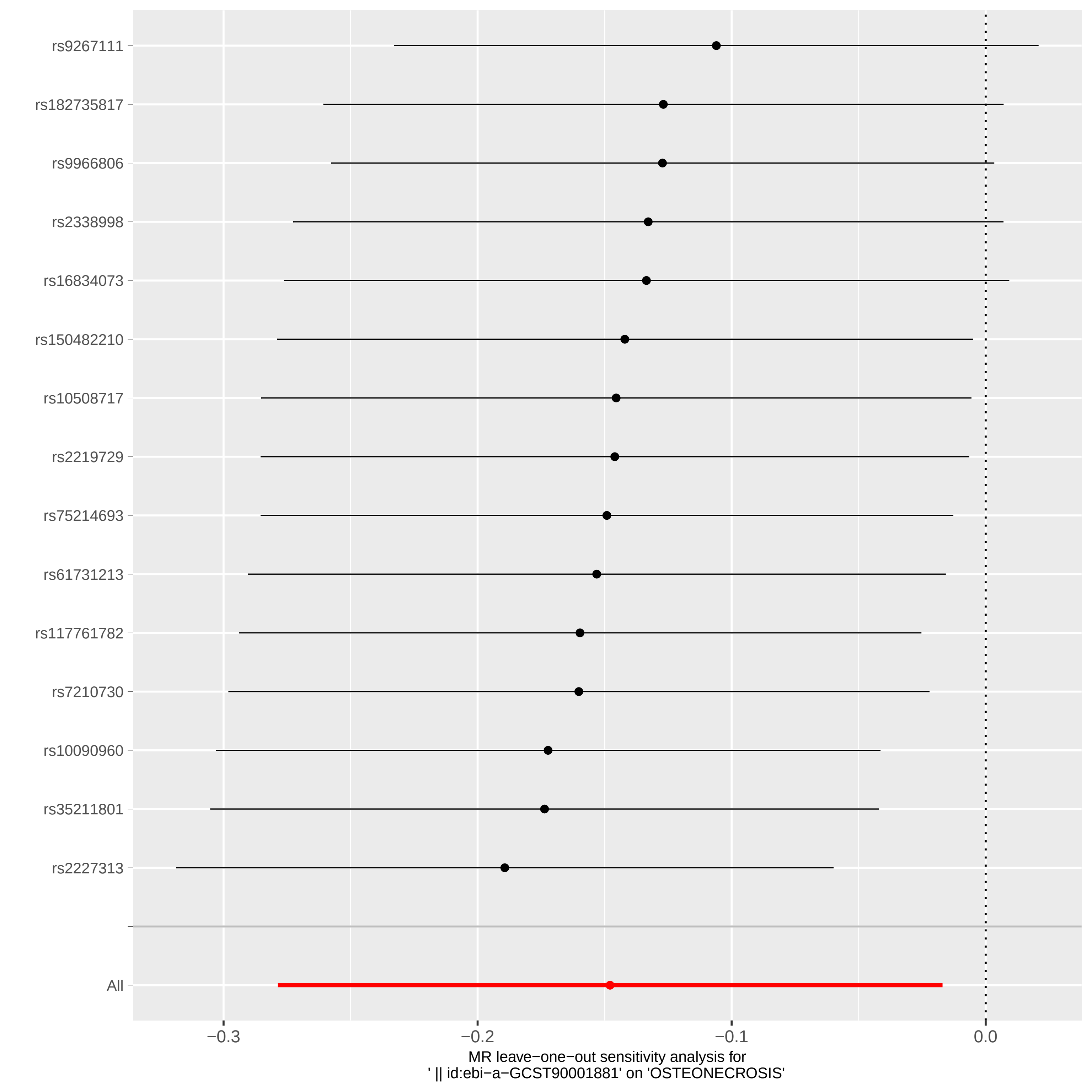
rs9267111
rs182735817
rs9966806
rs2338998
rs16834073
rs150482210
rs10508717
rs2219729
rs75214693
rs61731213
rs117761782
rs7210730
rs10090960
rs35211801
rs2227313
All
−0.3
−0.2
−0.1
0.0
MR leave−one−out sensitivity analysis for
' || id:ebi−a−GCST90001881' on 'OSTEONECROSIS'

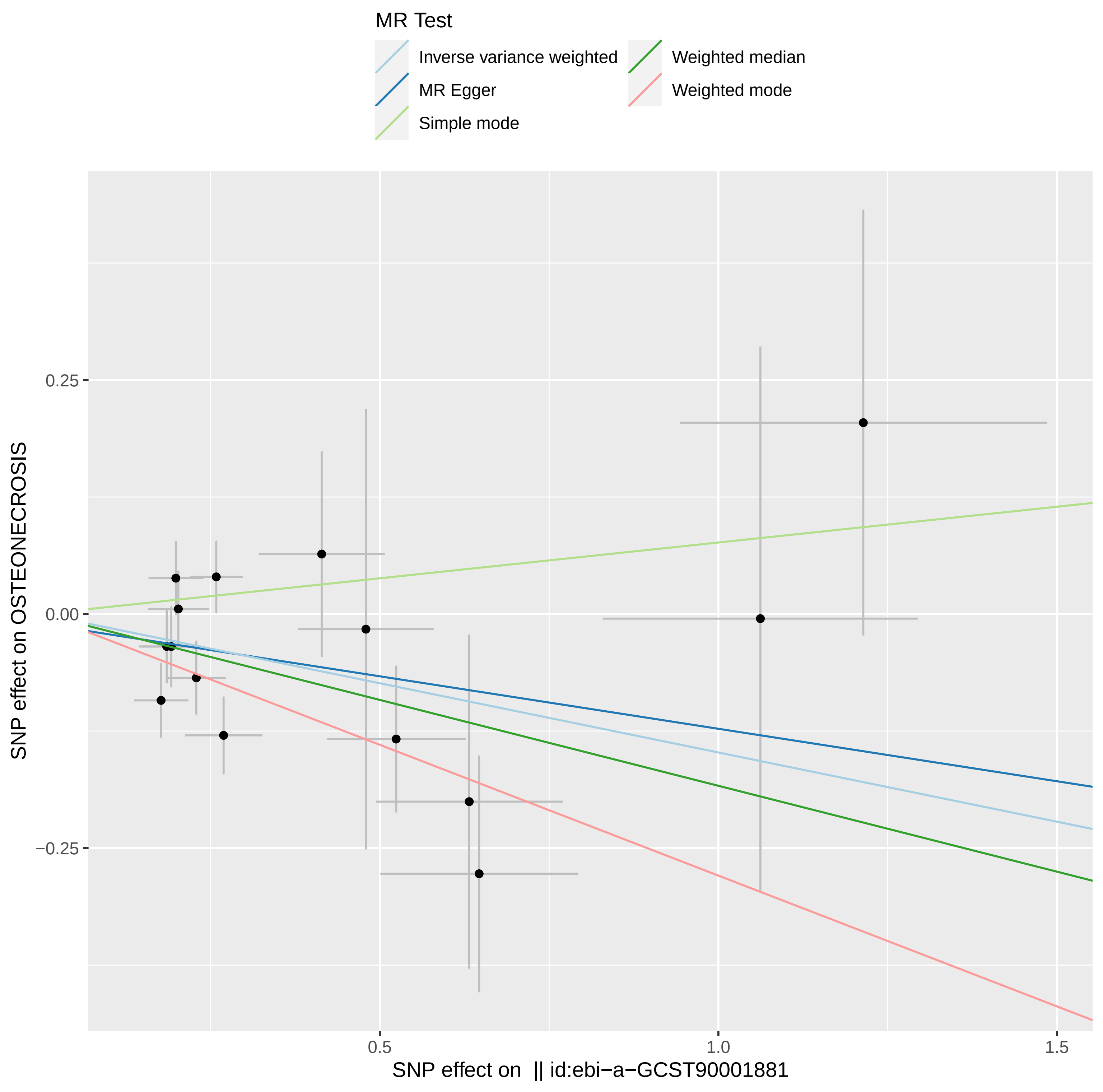
MR Test
Inverse variance weighted
MR Egger
Simple mode
Weighted median
Weighted mode
0.25
0.00
−0.25
SNP effect on OSTEONECROSIS
0.5
1.0
1.5
SNP effect on || id:ebi−a−GCST90001881

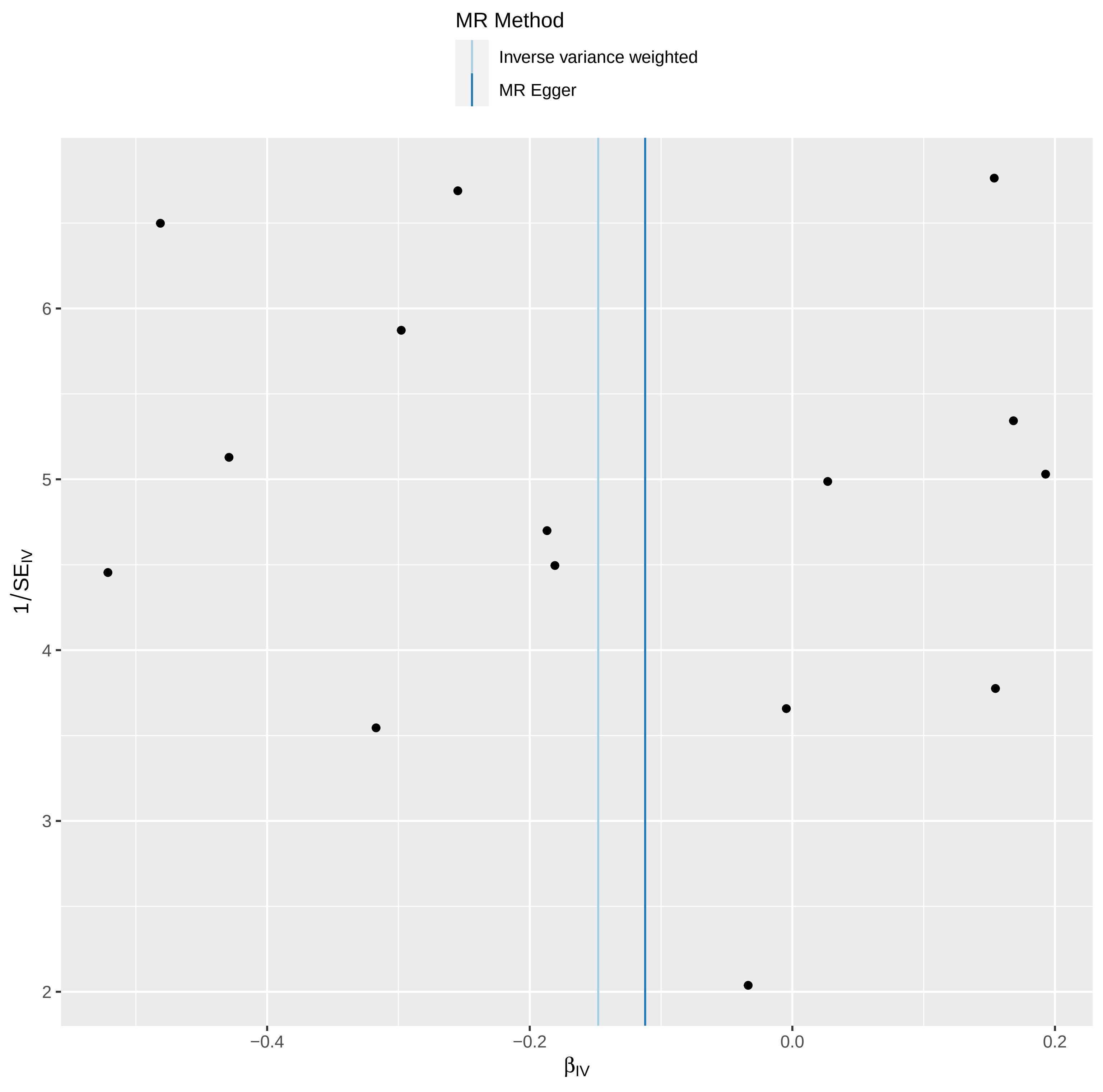

MR Method
Inverse variance weighted
MR Egger
6
5
4
3
2
1/SE_IV
−0.4
−0.2
0.0
0.2
β_IV

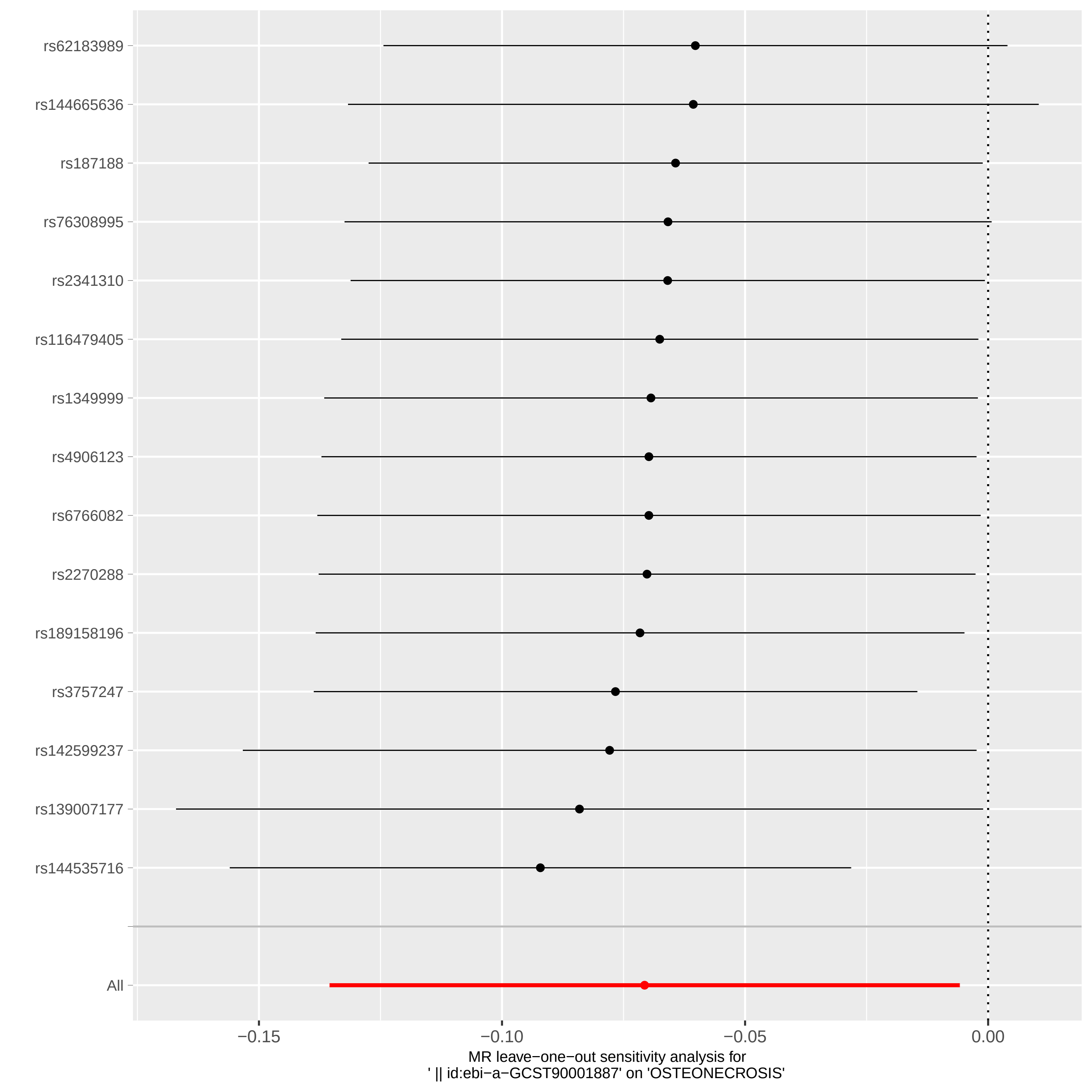
rs62183989
rs144665636
rs187188
rs76308995
rs2341310
rs116479405
rs1349999
rs4906123
rs6766082
rs2270288
rs189158196
rs3757247
rs142599237
rs139007177
rs144535716
All
−0.15
−0.10
−0.05
0.00
MR leave−one−out sensitivity analysis for
' || id:ebi−a−GCST90001887' on 'OSTEONECROSIS'

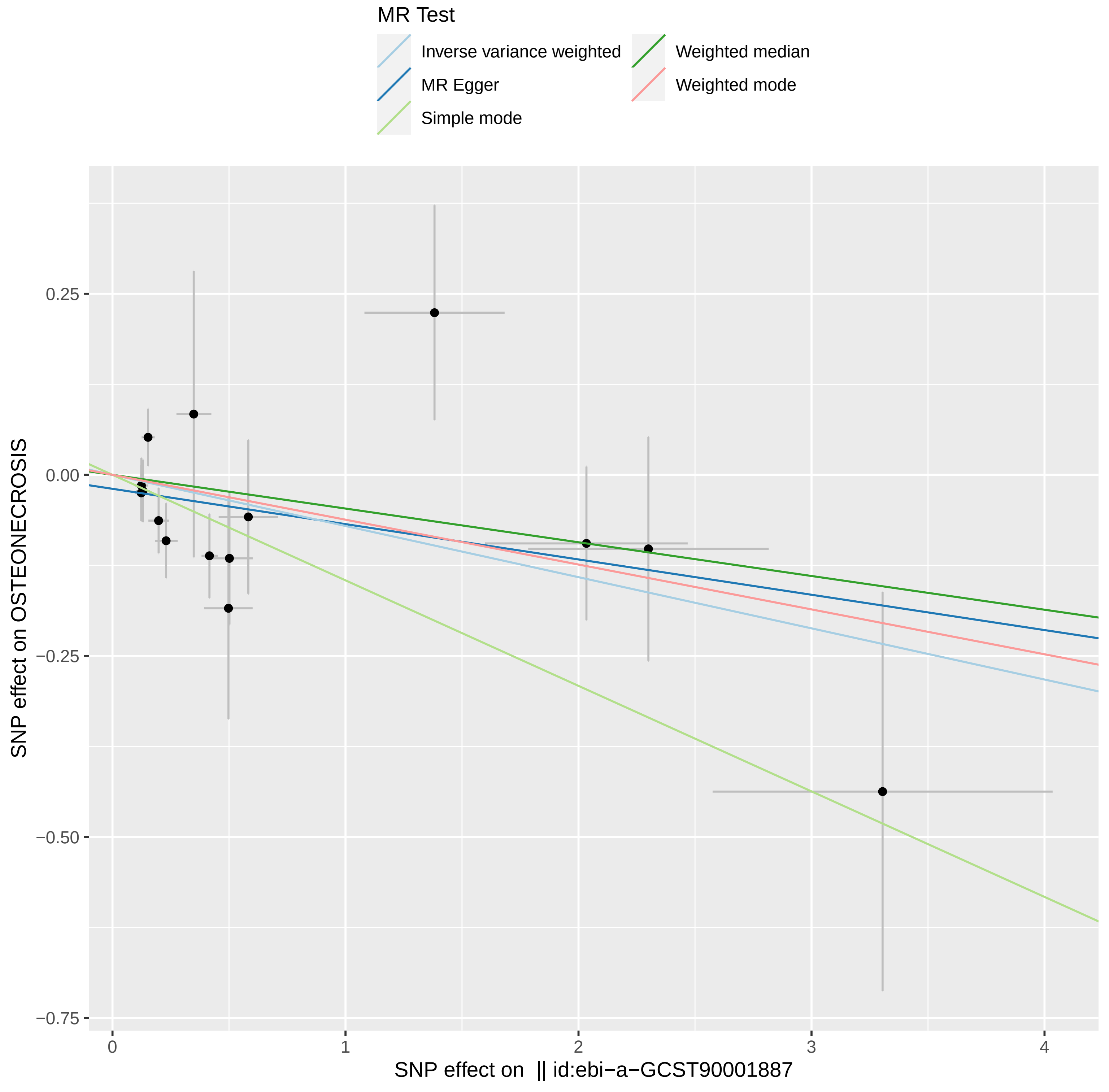
MR Test
Inverse variance weighted
MR Egger
Simple mode
Weighted median
Weighted mode
0.25
0.00
−0.25
−0.50
−0.75
SNP effect on OSTEONECROSIS
0
1
2
3
4
SNP effect on || id:ebi−a−GCST90001887

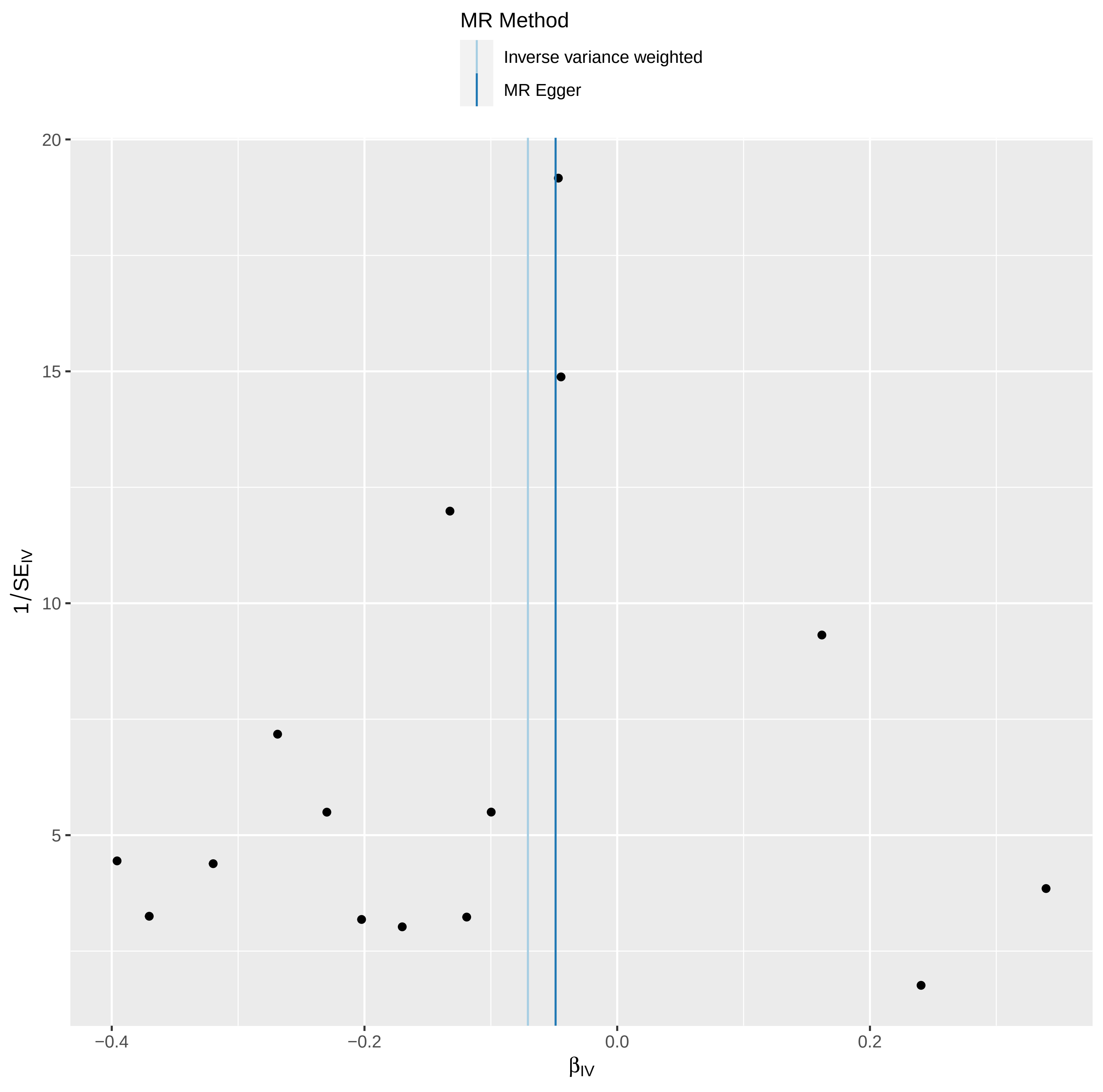
MR Method
Inverse variance weighted
MR Egger
20
15
10
5
$1/SE_{IV}$
−0.4
−0.2
0.0
0.2
β_{IV}

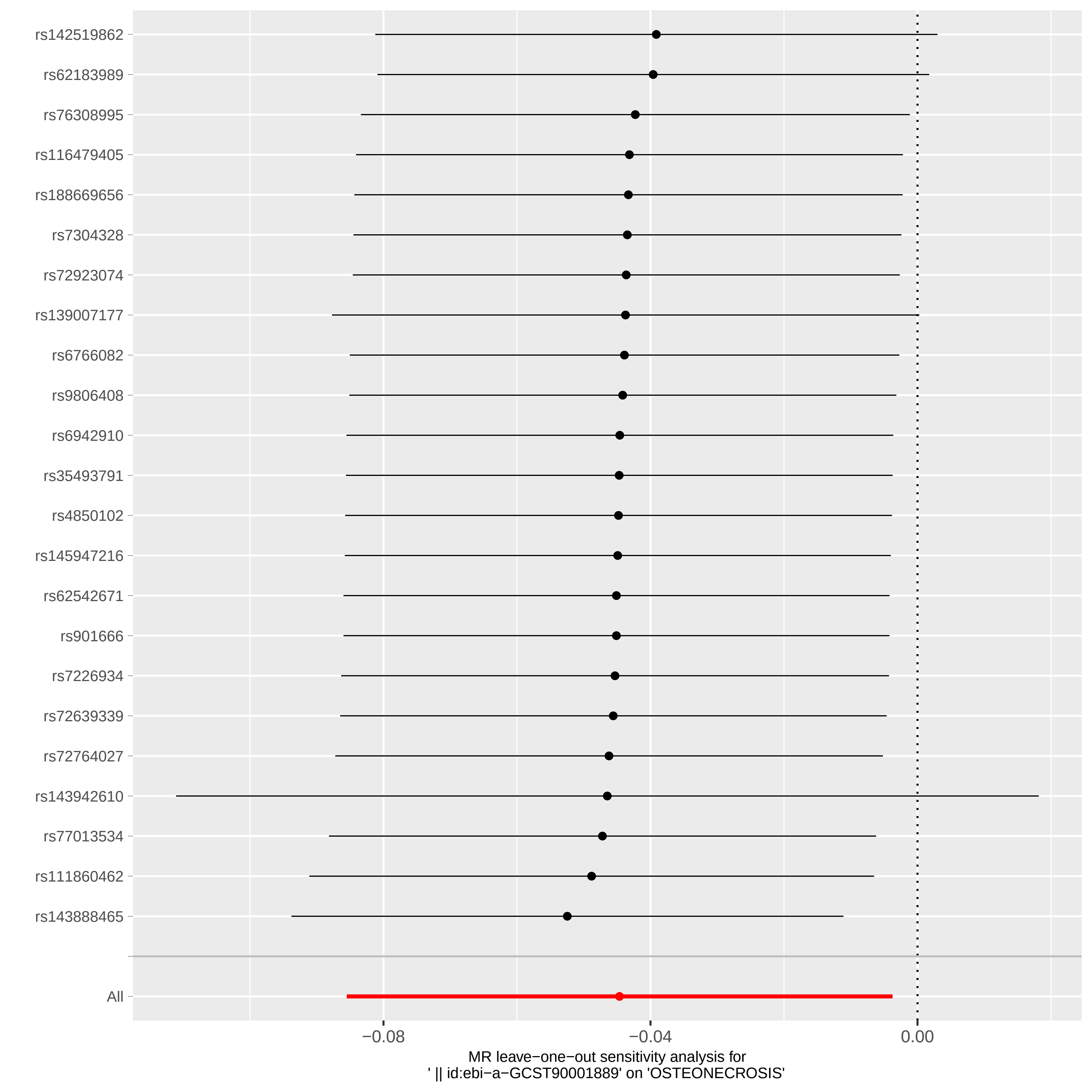
rs142519862
rs62183989
rs76308995
rs116479405
rs188669656
rs7304328
rs72923074
rs139007177
rs6766082
rs9806408
rs6942910
rs35493791
rs4850102
rs145947216
rs62542671
rs901666
rs7226934
rs72639339
rs72764027
rs143942610
rs77013534
rs111860462
rs143888465
All
−0.08
−0.04
0.00
MR leave−one−out sensitivity analysis for
' || id:ebi−a−GCST90001889' on 'OSTEONECROSIS'

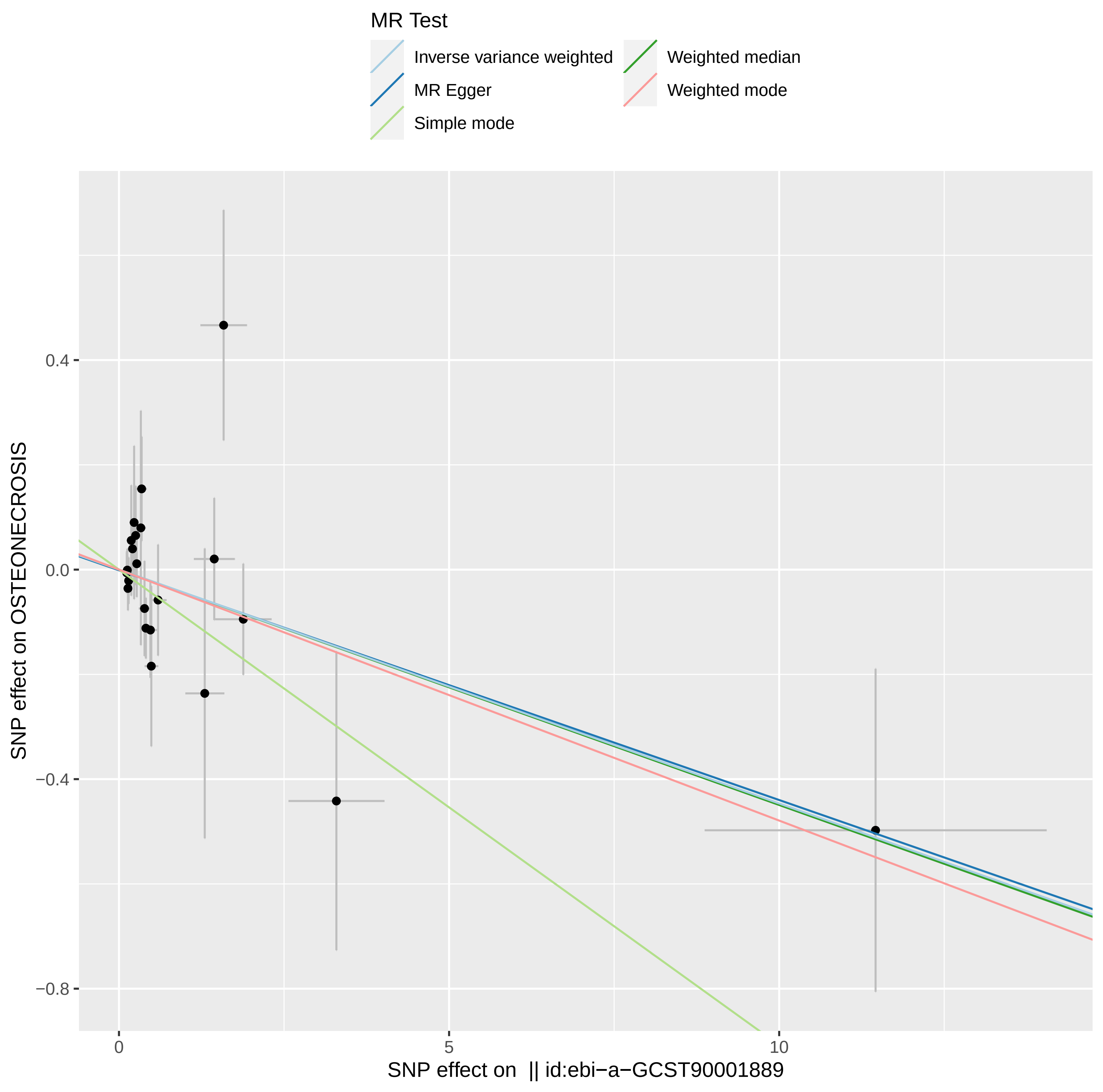

MR Test
Inverse variance weighted
MR Egger
Simple mode
Weighted median
Weighted mode
0.4
0.0
−0.4
−0.8
SNP effect on OSTEONECROSIS
0
5
10
SNP effect on || id:ebi−a−GCST90001889

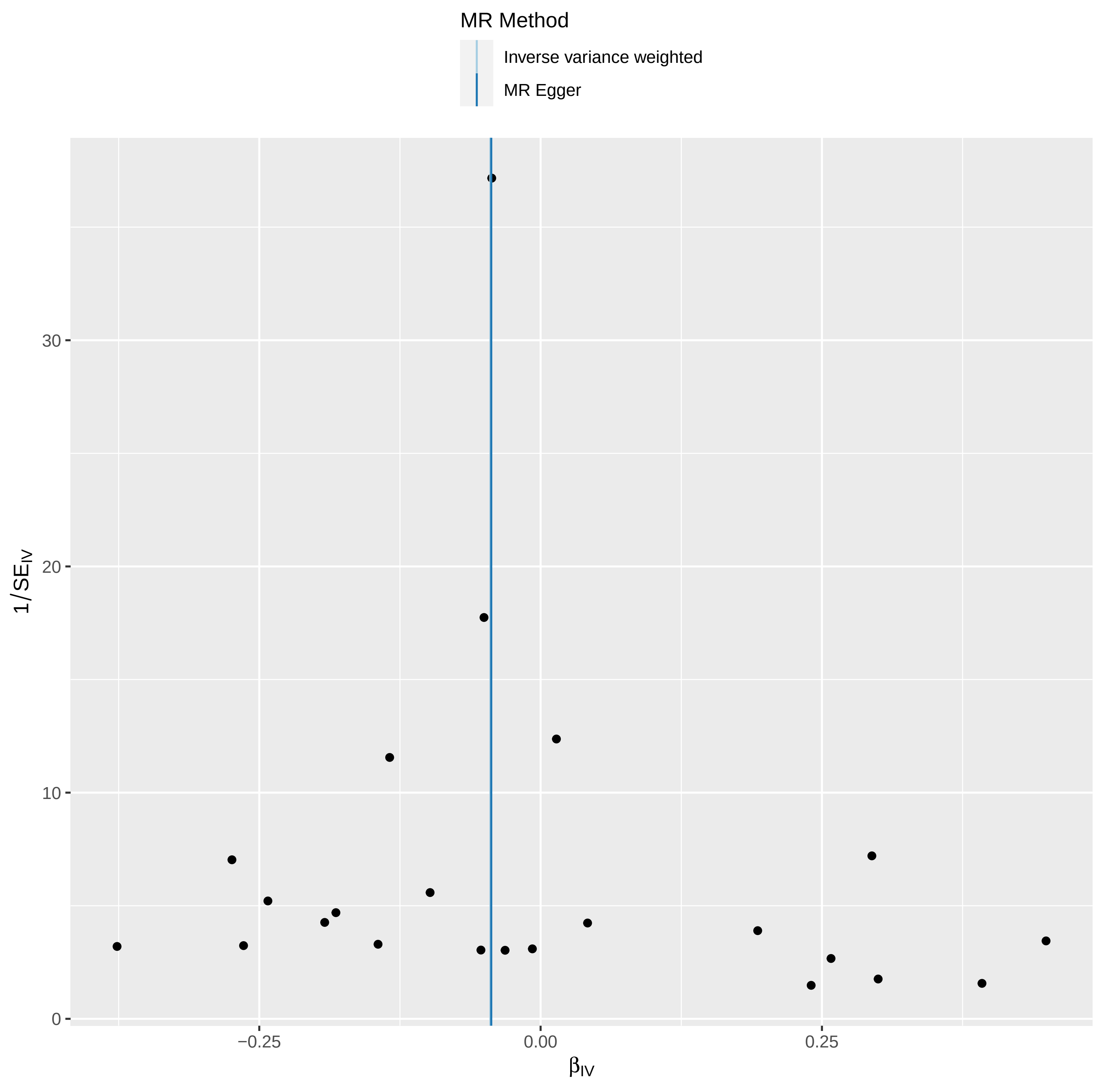

MR Method
Inverse variance weighted
MR Egger
30
20
10
0
1/SE_IV
−0.25
0.00
0.25
β_IV

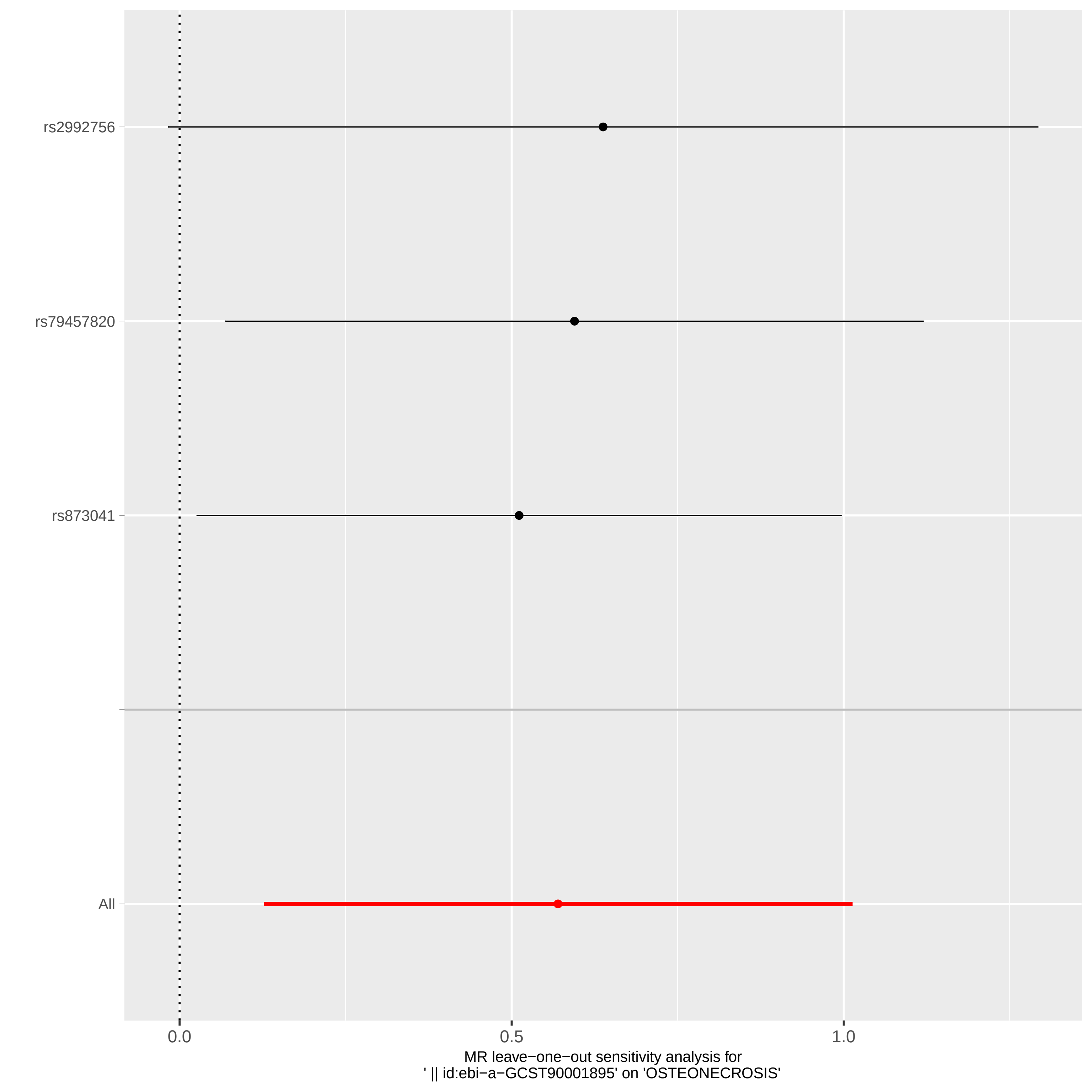
rs2992756
rs79457820
rs873041
All
0.0
0.5
1.0
MR leave−one−out sensitivity analysis for
' || id:ebi−a−GCST90001895' on 'OSTEONECROSIS'

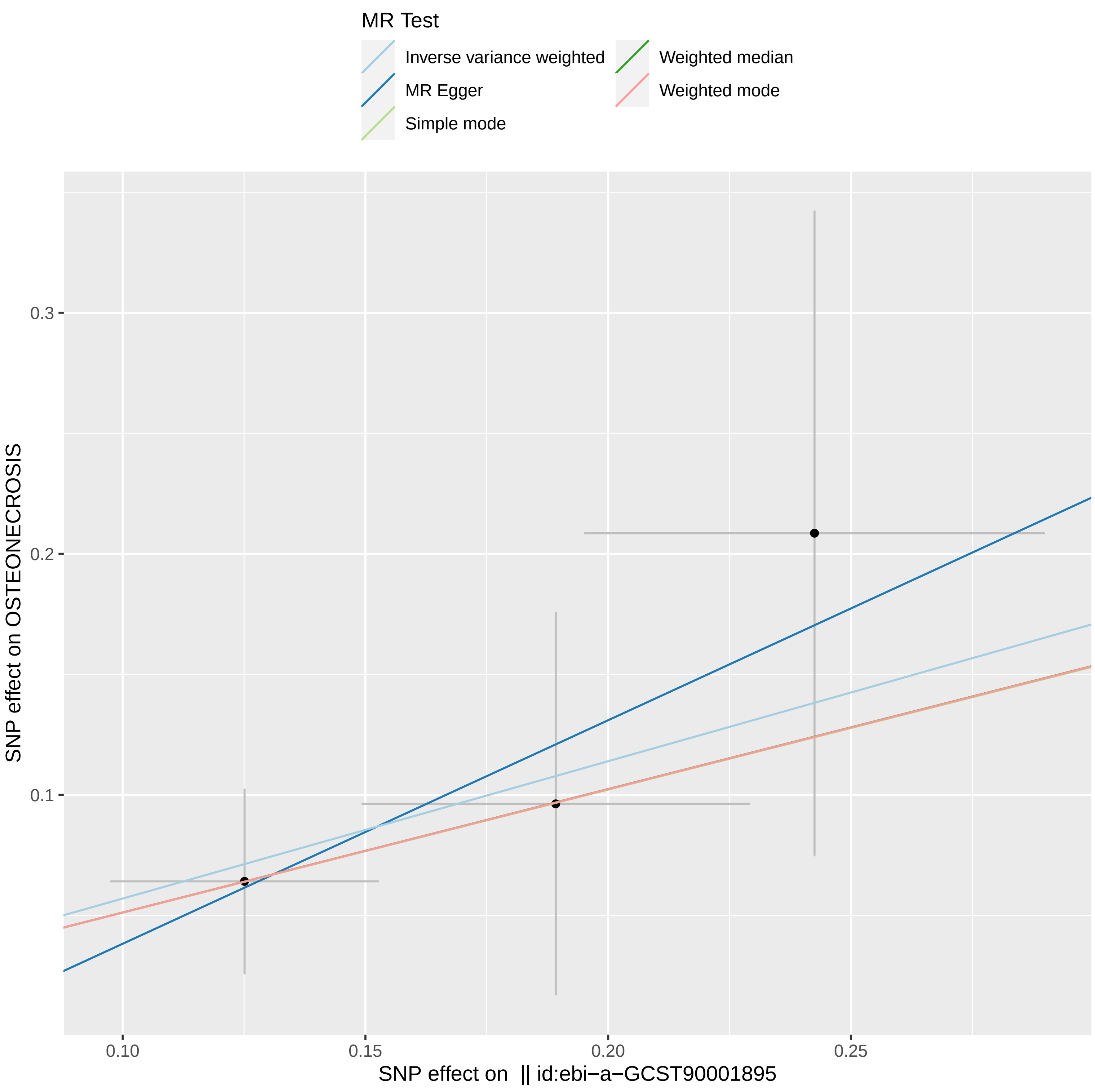
MR Test
Inverse variance weighted
MR Egger
Simple mode
Weighted median
Weighted mode
0.3
0.2
0.1
SNP effect on OSTEONECROSIS
0.10
0.15
0.20
0.25
SNP effect on || id:ebi−a−GCST90001895

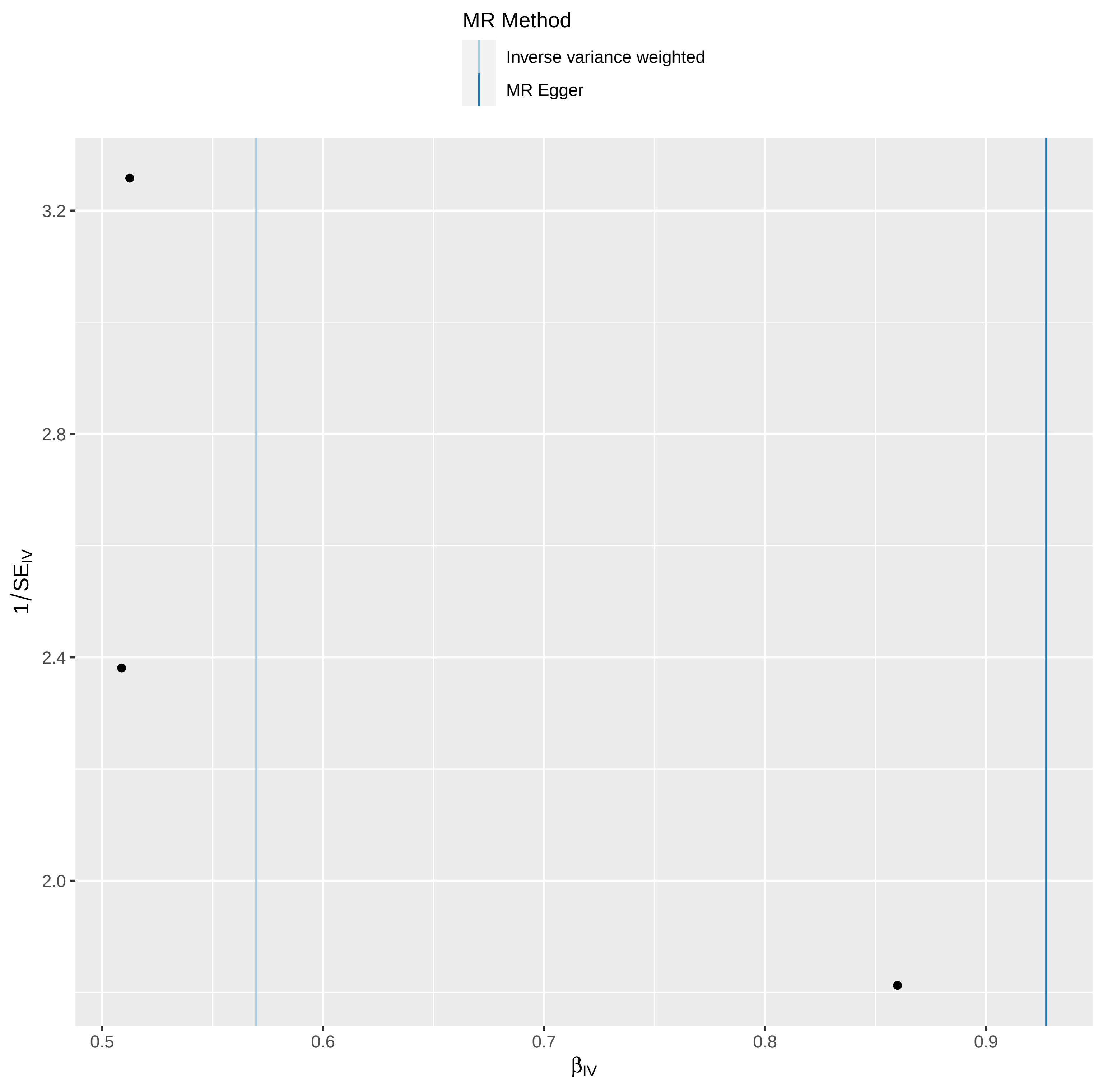
MR Method
Inverse variance weighted
MR Egger
3.2
2.8
2.4
2.0
1/SE$_{IV}$
0.5
0.6
0.7
0.8
0.9
β_{IV}

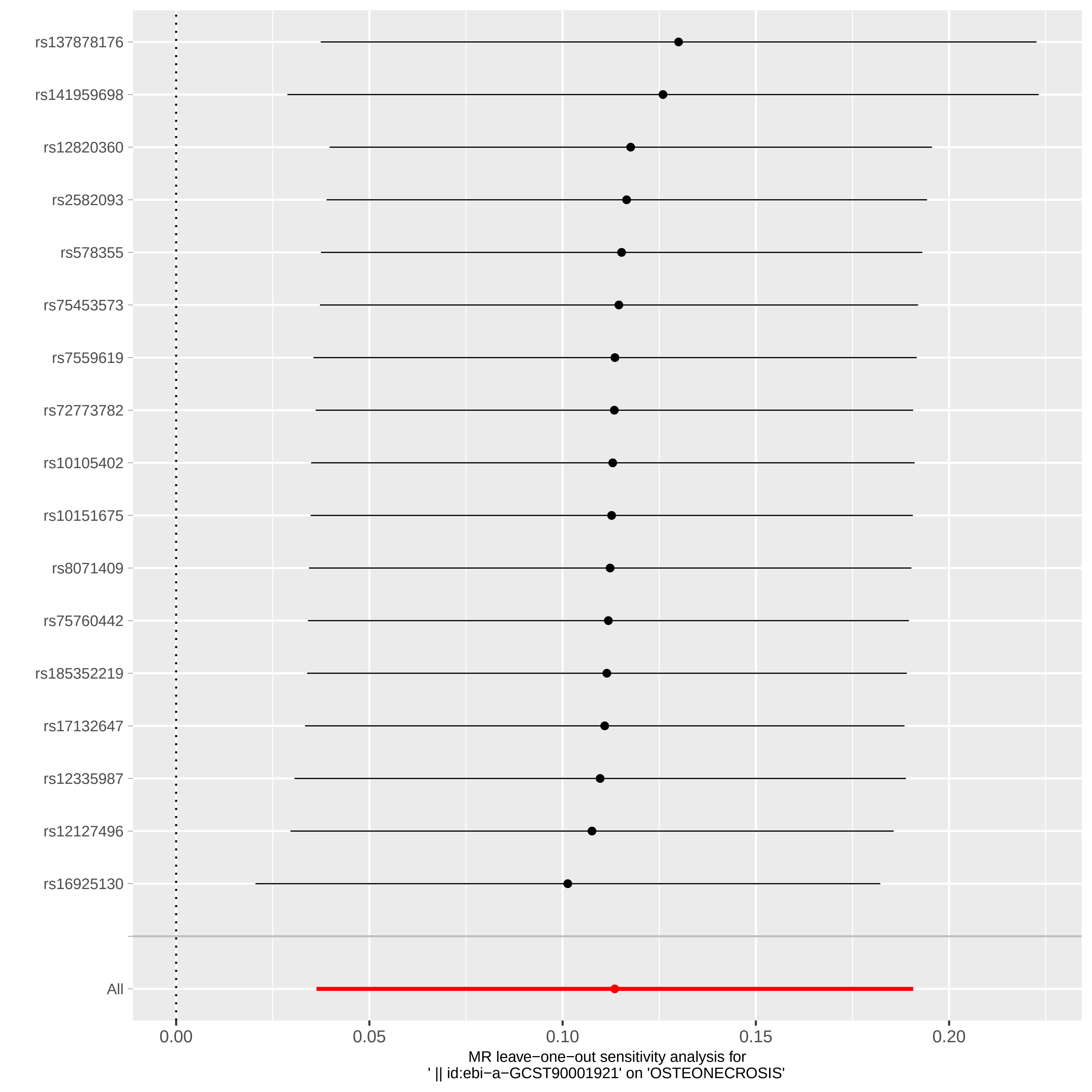
rs137878176
rs141959698
rs12820360
rs2582093
rs578355
rs75453573
rs7559619
rs72773782
rs10105402
rs10151675
rs8071409
rs75760442
rs185352219
rs17132647
rs12335987
rs12127496
rs16925130
All
0.00
0.05
0.10
0.15
0.20
MR leave−one−out sensitivity analysis for
' || id:ebi−a−GCST90001921' on 'OSTEONECROSIS'

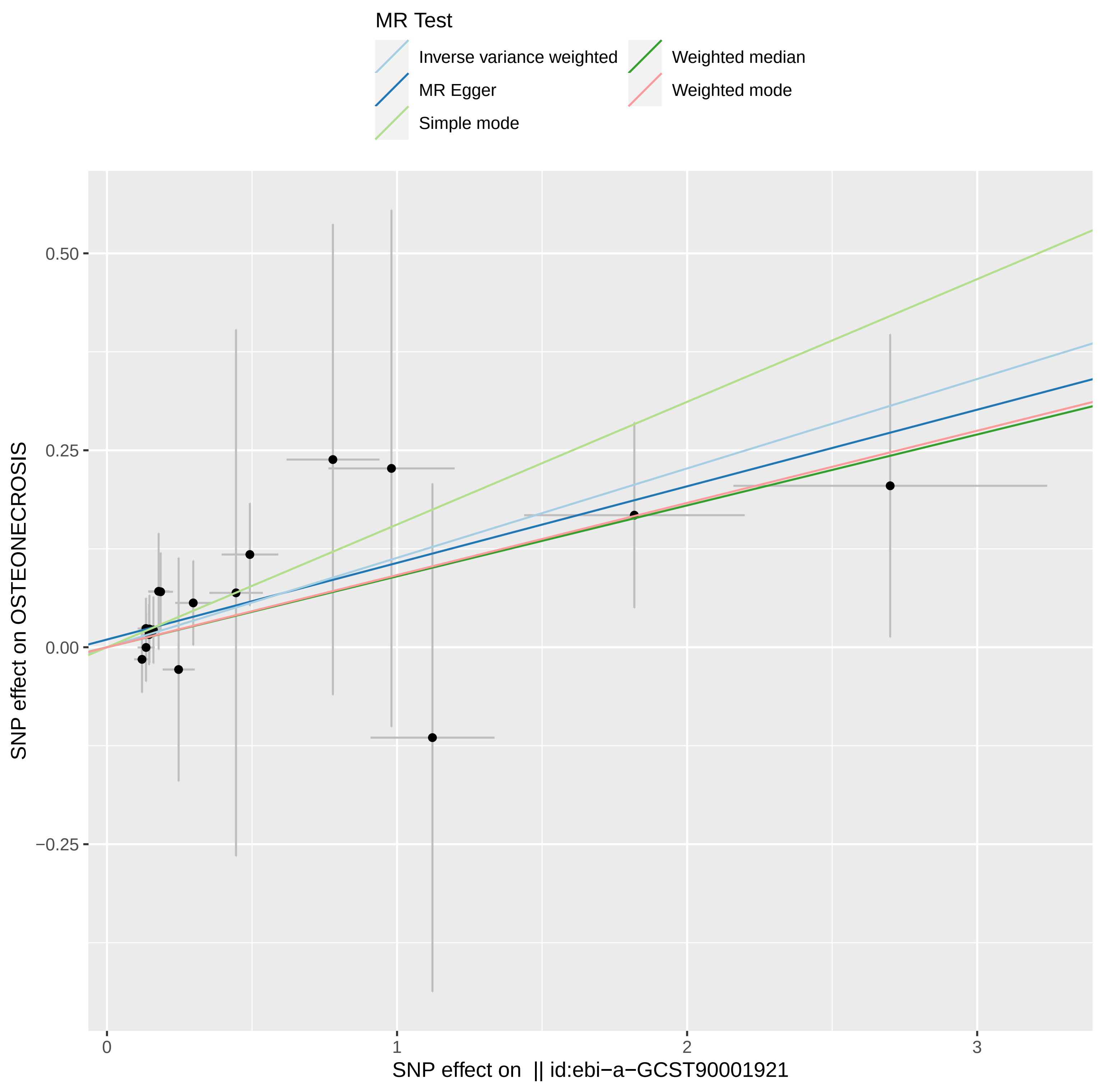

MR Test
Inverse variance weighted
MR Egger
Simple mode
Weighted median
Weighted mode
0.50
0.25
0.00
−0.25
SNP effect on OSTEONECROSIS
0
1
2
3
SNP effect on || id:ebi−a−GCST90001921

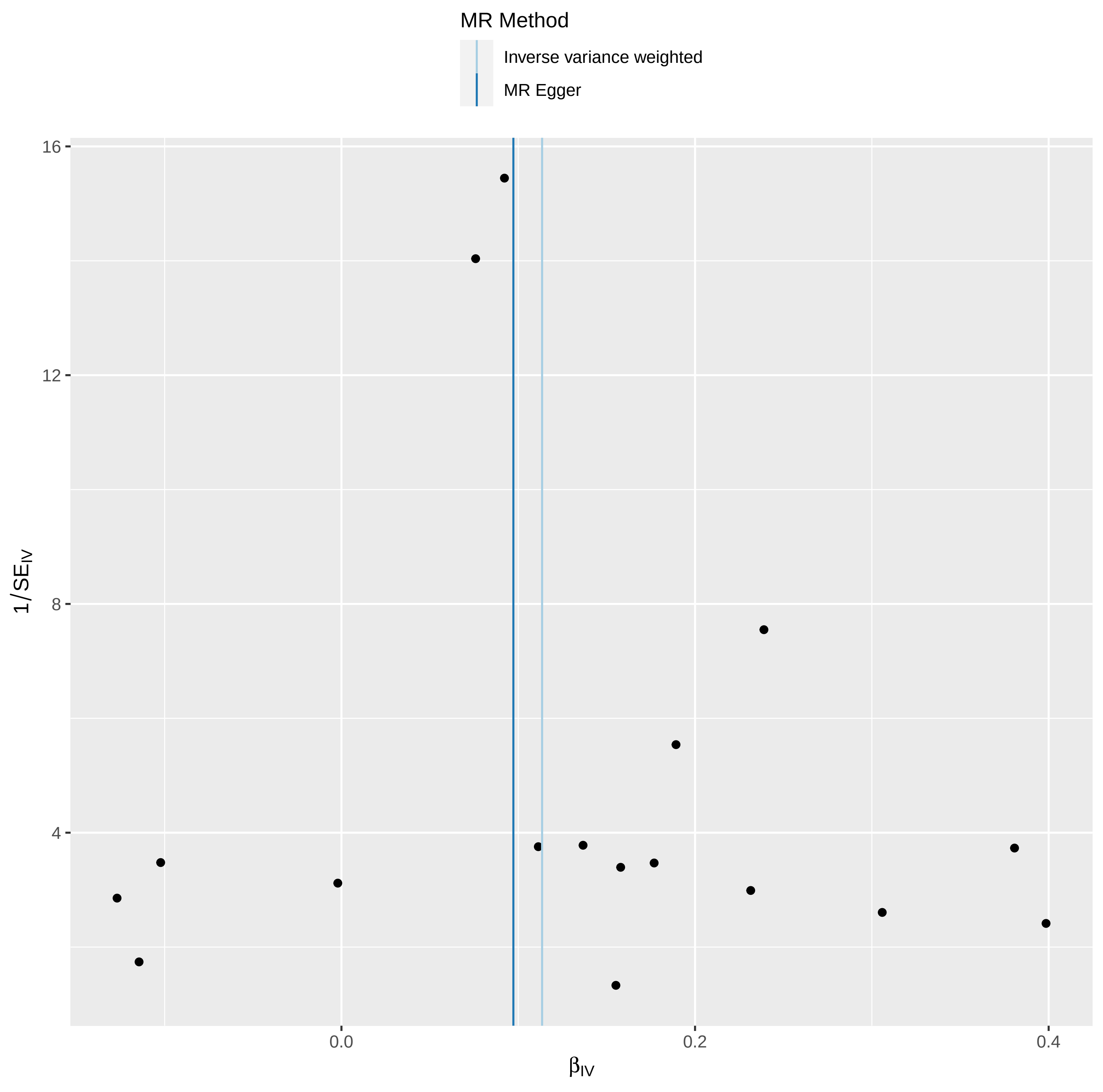
MR Method
Inverse variance weighted
MR Egger
16
12
8
4
1/SE_IV
0.0
0.2
0.4
β_IV

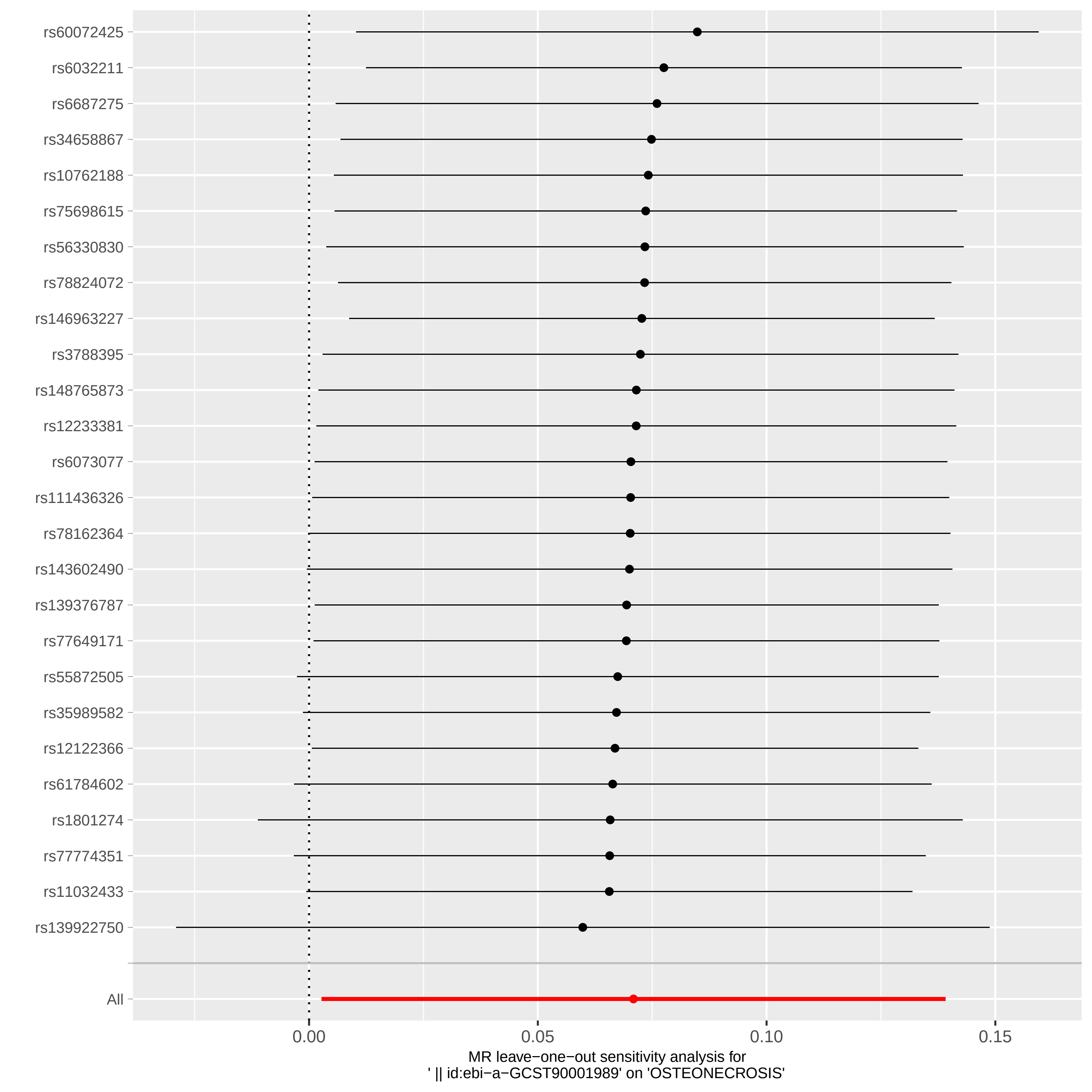
rs60072425
rs6032211
rs6687275
rs34658867
rs10762188
rs75698615
rs56330830
rs78824072
rs146963227
rs3788395
rs148765873
rs12233381
rs6073077
rs111436326
rs78162364
rs143602490
rs139376787
rs77649171
rs55872505
rs35989582
rs12122366
rs61784602
rs1801274
rs77774351
rs11032433
rs139922750
All
0.00
0.05
0.10
0.15
MR leave−one−out sensitivity analysis for
' || id:ebi−a−GCST90001989' on 'OSTEONECROSIS'

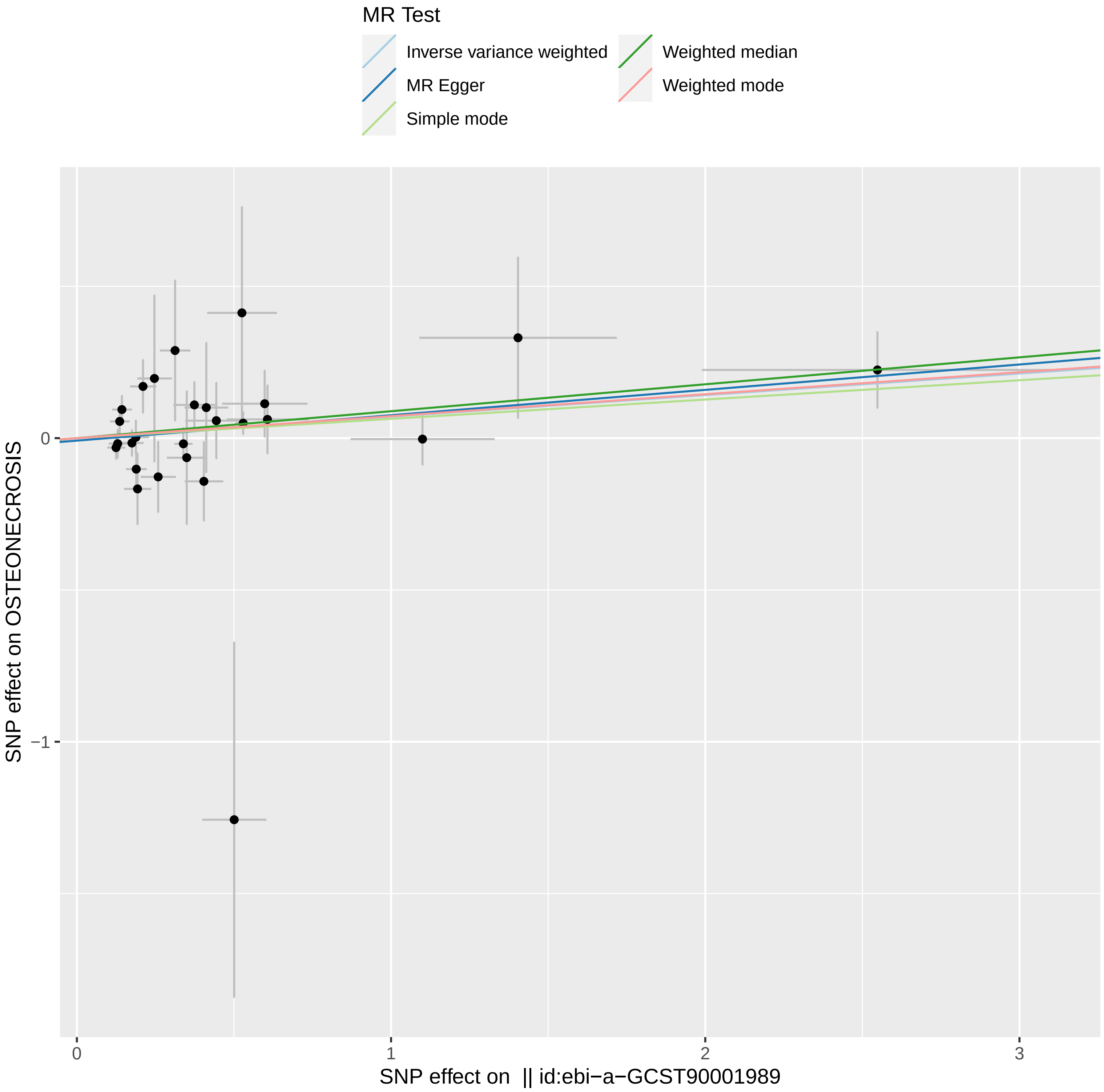
MR Test
Inverse variance weighted
MR Egger
Simple mode
Weighted median
Weighted mode
SNP effect on OSTEONECROSIS
0
−1
0
1
2
3
SNP effect on || id:ebi−a−GCST90001989

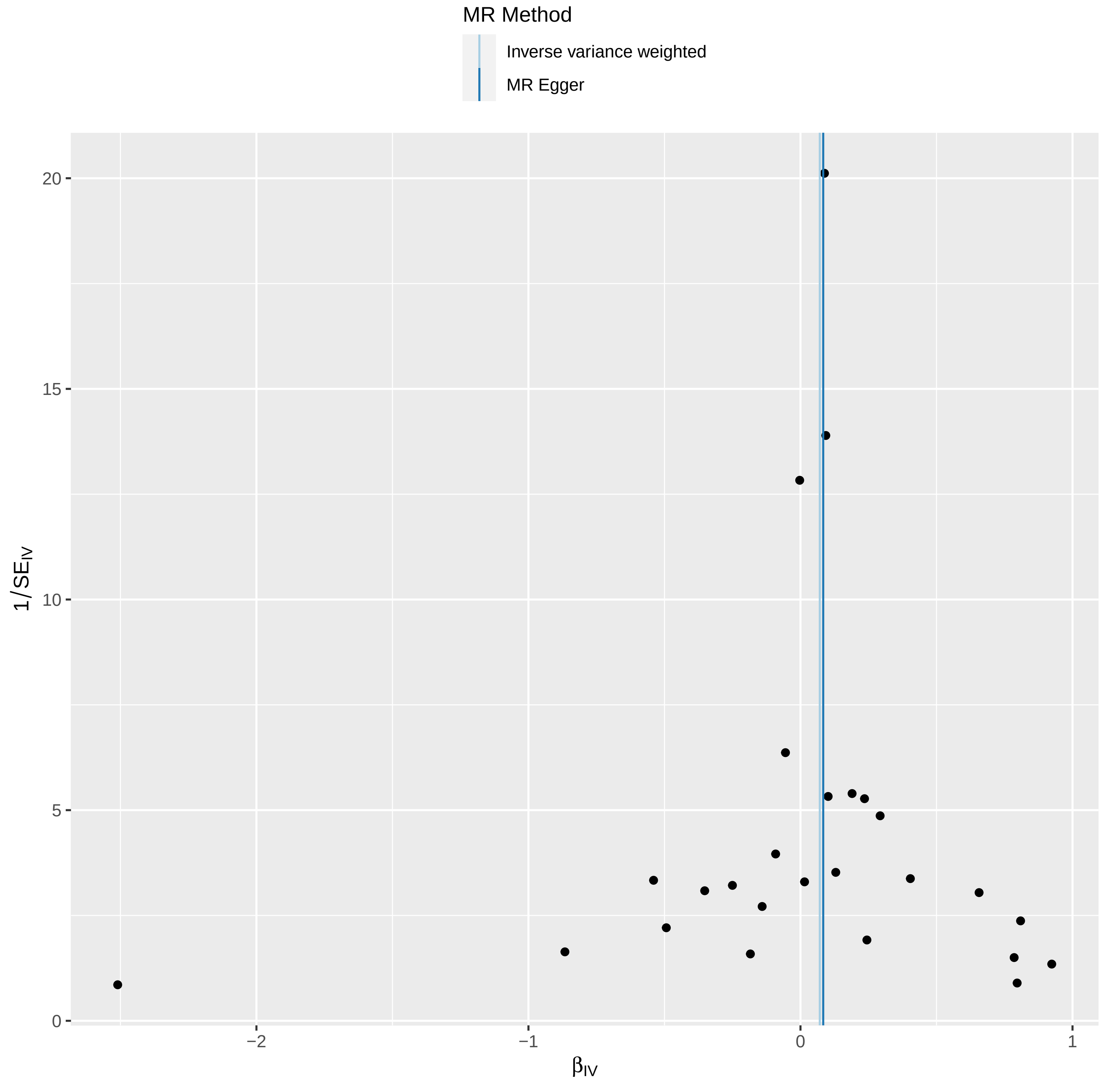

MR Method
Inverse variance weighted
MR Egger
20
15
10
5
0
1/SE$_{IV}$
−2
−1
0
1
β$_{IV}$

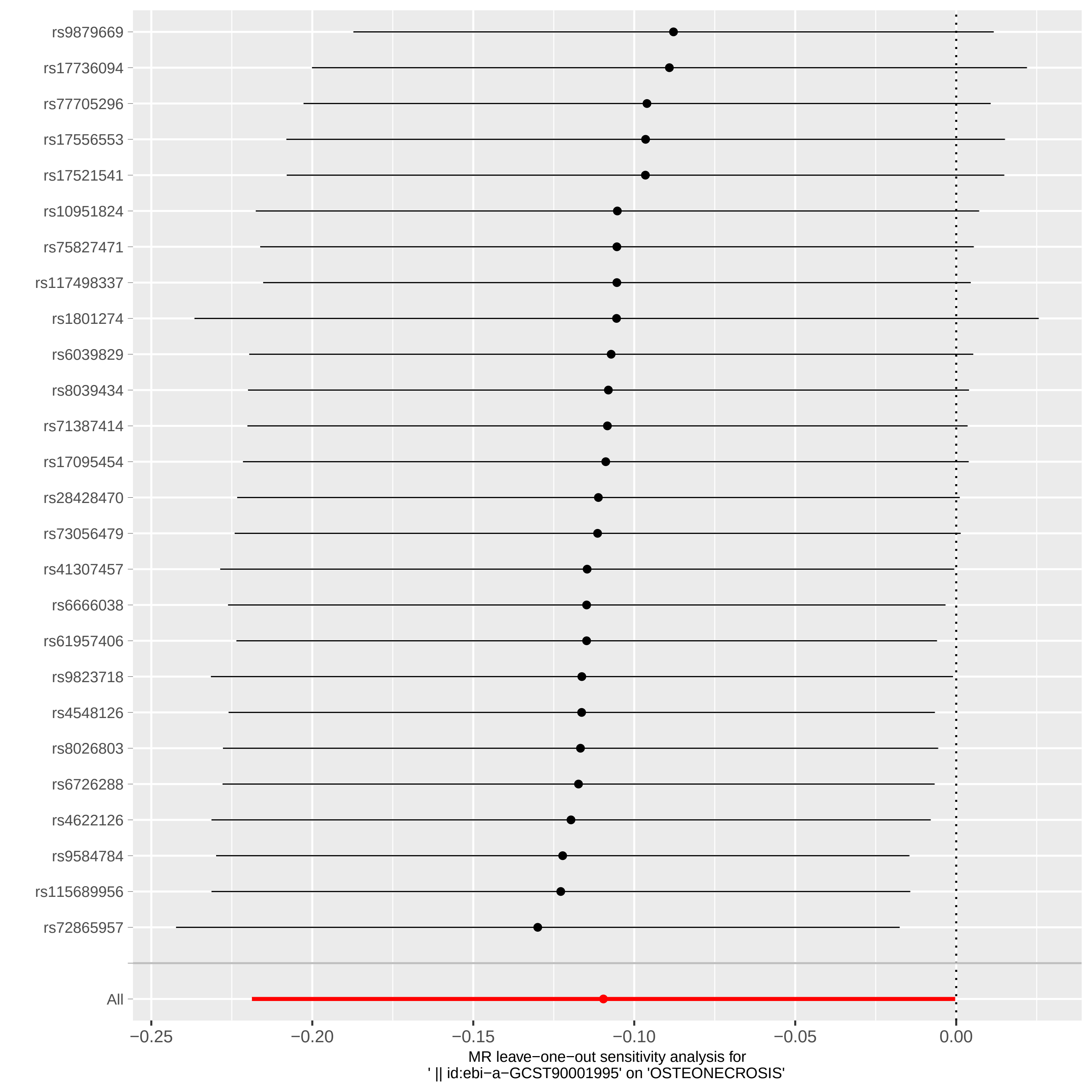
rs9879669
rs17736094
rs77705296
rs17556553
rs17521541
rs10951824
rs75827471
rs117498337
rs1801274
rs6039829
rs8039434
rs71387414
rs17095454
rs28428470
rs73056479
rs41307457
rs6666038
rs61957406
rs9823718
rs4548126
rs8026803
rs6726288
rs4622126
rs9584784
rs115689956
rs72865957
All
−0.25
−0.20
−0.15
−0.10
−0.05
0.00
MR leave−one−out sensitivity analysis for
' || id:ebi−a−GCST90001995' on 'OSTEONECROSIS'

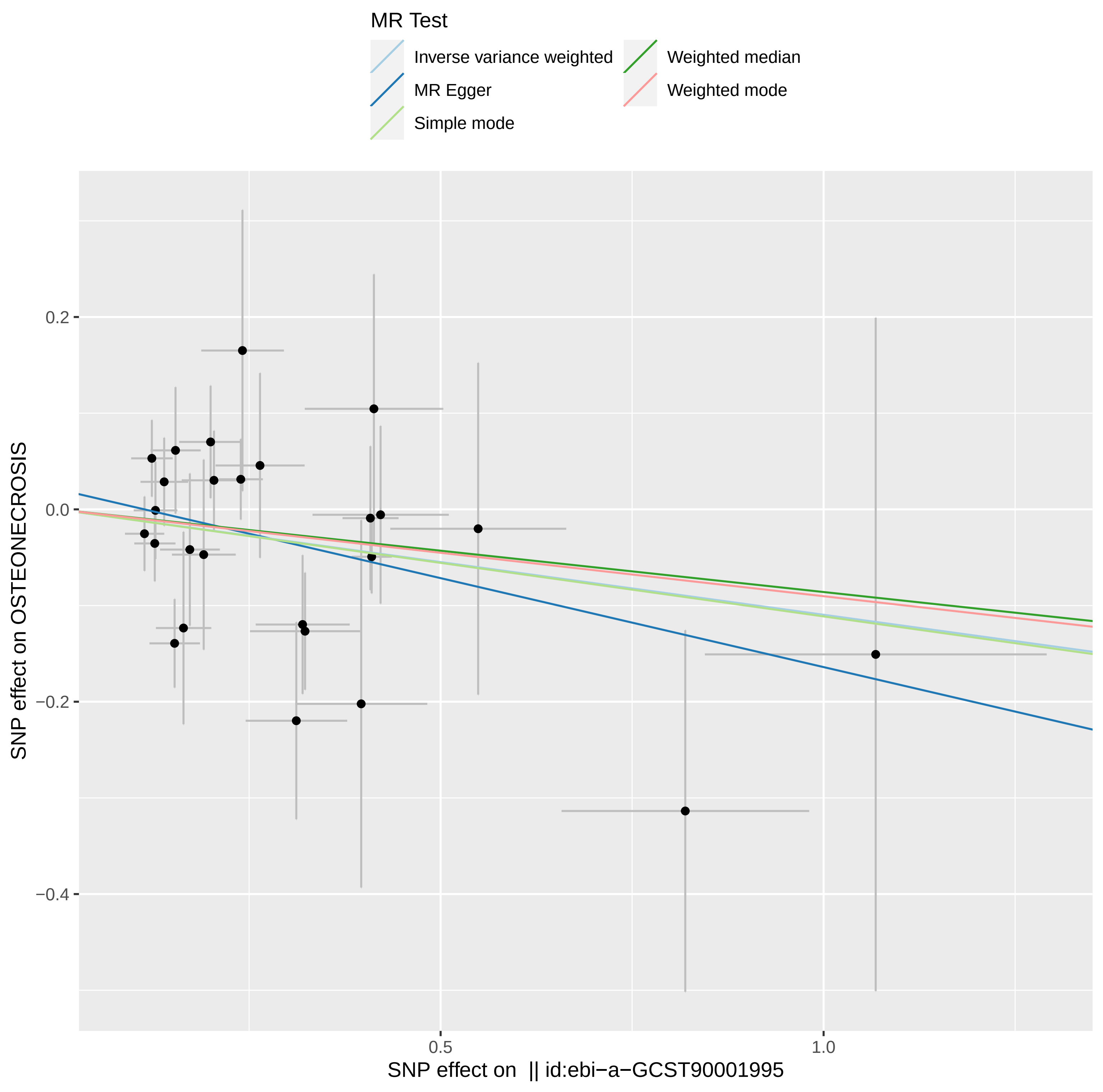

MR Test
Inverse variance weighted
MR Egger
Simple mode
Weighted median
Weighted mode
0.2
0.0
−0.2
−0.4
0.5
1.0
SNP effect on OSTEONECROSIS
SNP effect on || id:ebi−a−GCST90001995

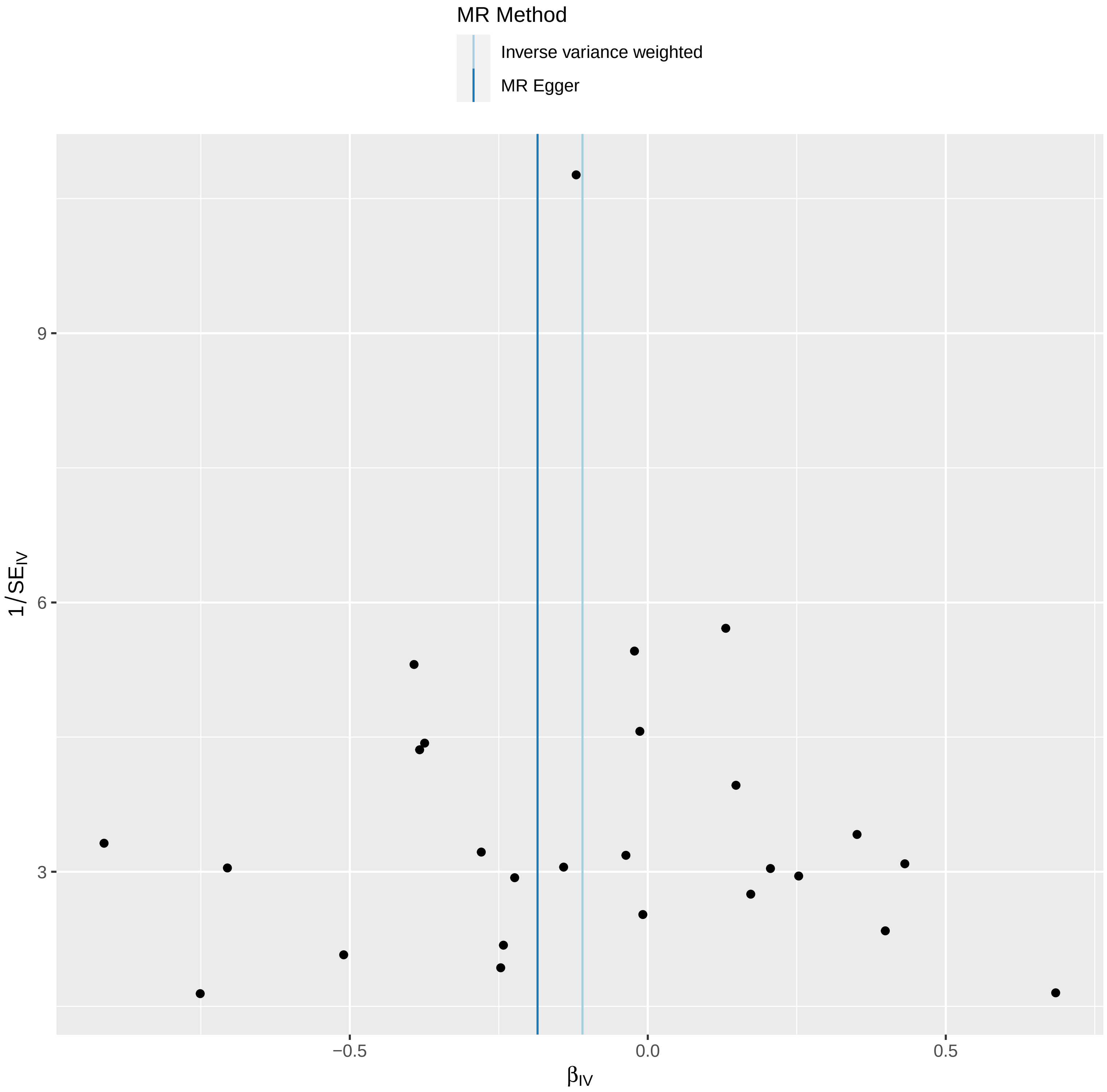
MR Method
Inverse variance weighted
MR Egger
1/SE_IV
9
6
3
−0.5
0.0
0.5
β_IV

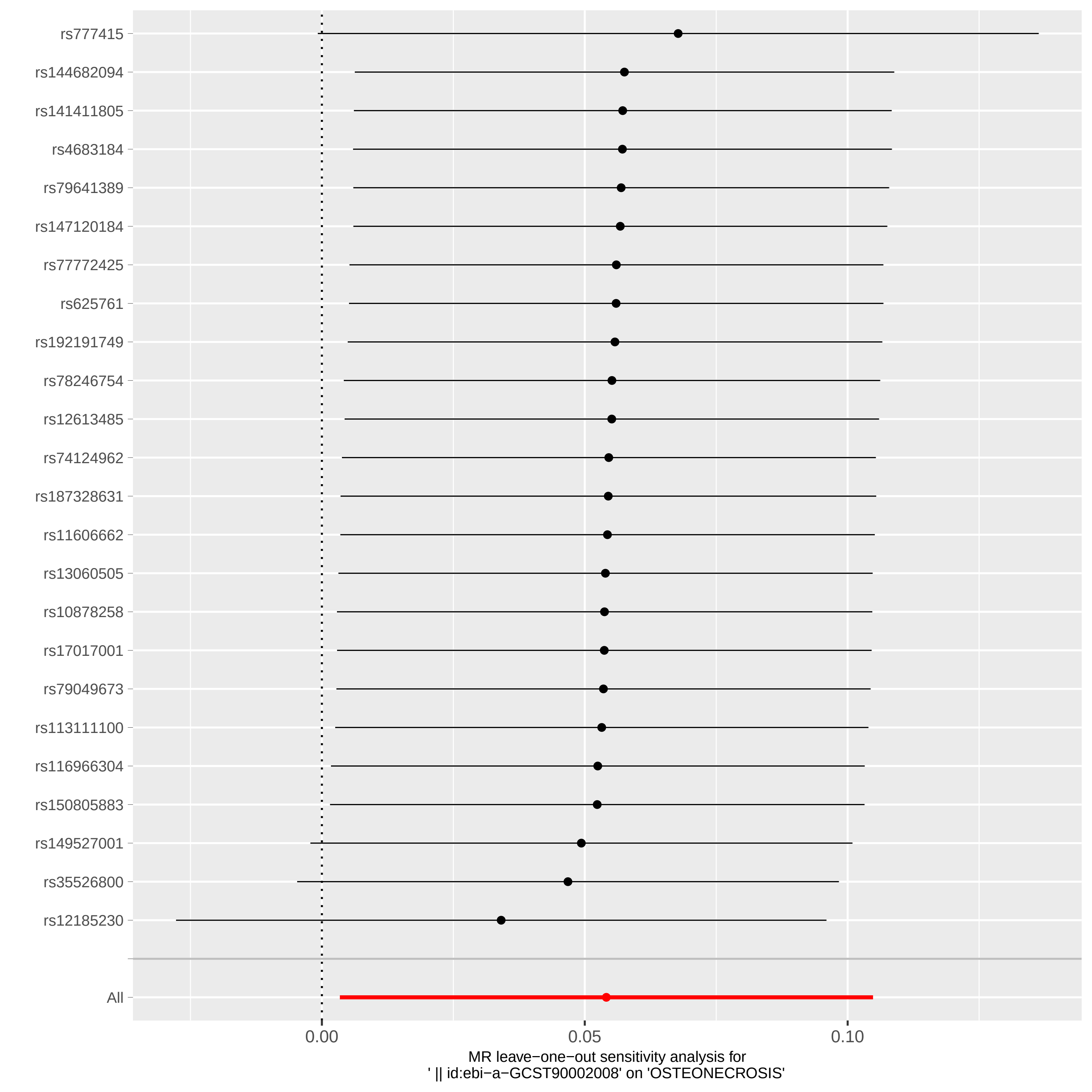

rs777415
rs144682094
rs141411805
rs4683184
rs79641389
rs147120184
rs77772425
rs625761
rs192191749
rs78246754
rs12613485
rs74124962
rs187328631
rs11606662
rs13060505
rs10878258
rs17017001
rs79049673
rs113111100
rs116966304
rs150805883
rs149527001
rs35526800
rs12185230
All
0.00
0.05
0.10
MR leave−one−out sensitivity analysis for
' || id:ebi−a−GCST90002008' on 'OSTEONECROSIS'

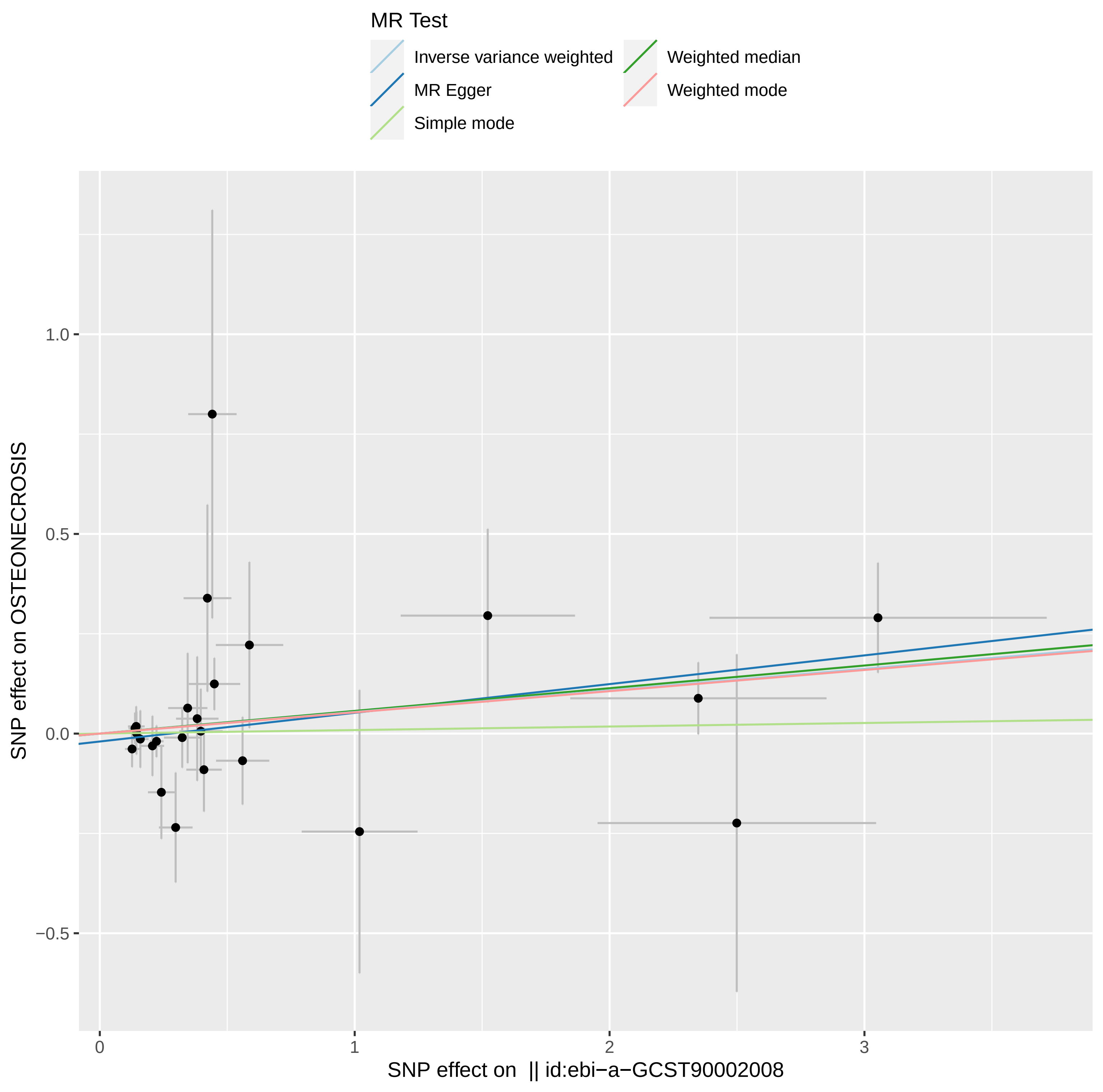
MR Test
Inverse variance weighted
MR Egger
Simple mode
Weighted median
Weighted mode
1.0
0.5
0.0
−0.5
0
1
2
3
SNP effect on OSTEONECROSIS
SNP effect on || id:ebi−a−GCST90002008

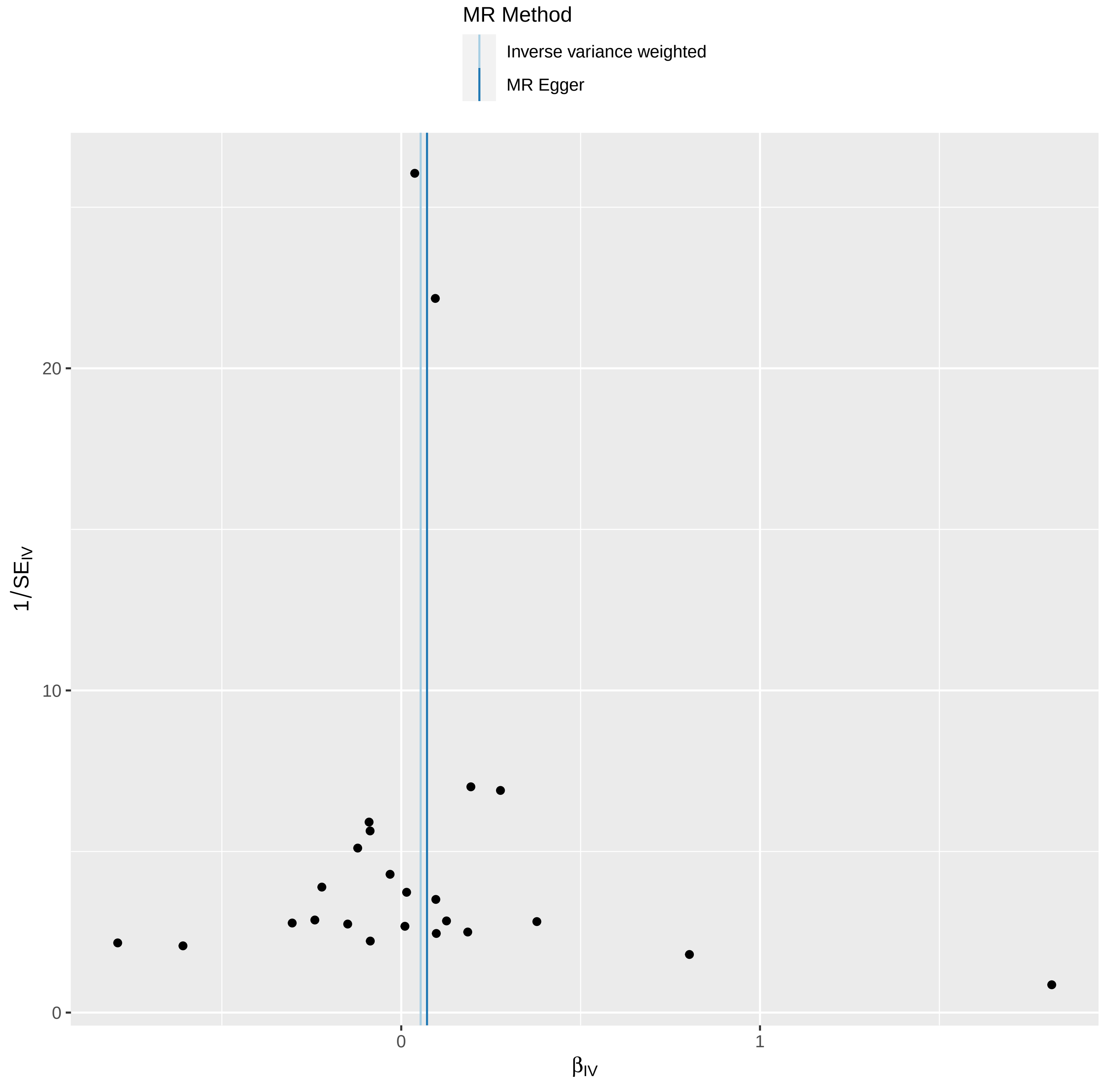
MR Method
Inverse variance weighted
MR Egger
20
10
0
1/SE$_{IV}$
0
1
β$_{IV}$
