# Supplementary material for: Complex causal association between genetically predicted 731 immunocyte phenotype and osteonecrosis: a bidirectional two-sample Mendelian randomization analysis
Source: Int J Surg. 2024 Mar 18;110(6):3285–93. doi: 10.1097/JS9.0000000000001327 (PMC11175804; doi:10.1097/JS9.0000000000001327)
Supplement: Supplementary file 4 [file js9-110-3285-s004.pdf]

| ID | method | nsnp | b | se | pval | OR(95%CI) |
|---|---|---|---|---|---|---|
| Sw mem %B cell | MR Egger | 20 | −0.05731485 | 0.02801807 | 0.055690103 | 0.94(0.89 to 1.00) |
| Sw mem %B cell | Weighted median | 20 | −0.04737803 | 0.02960026 | 0.109466608 | 0.95(0.90 to 1.01) |
| Sw mem %B cell | Inverse variance weighted | 20 | −0.04322006 | 0.02075484 | 0.037305162 | 0.96(0.92 to 1.00) |
| Sw mem %B cell | Simple mode | 20 | −0.02424207 | 0.04941802 | 0.629361225 | 0.98(0.89 to 1.08) |
| Sw mem %B cell | Weighted mode | 20 | −0.03795774 | 0.02986523 | 0.219081268 | 0.96(0.91 to 1.02) |
| IgD+ CD38br %B cell | MR Egger | 20 | 0.03015618 | 0.02701760 | 0.279029512 | 1.03(0.98 to 1.09) |
| IgD+ CD38br %B cell | Weighted median | 20 | 0.04218902 | 0.03217414 | 0.189766167 | 1.04(0.98 to 1.11) |
| IgD+ CD38br %B cell | Inverse variance weighted | 20 | 0.04826186 | 0.02000107 | 0.015823391 | 1.05(1.01 to 1.09) |
| IgD+ CD38br %B cell | Simple mode | 20 | 0.09056859 | 0.05283243 | 0.102747649 | 1.09(0.99 to 1.21) |
| IgD+ CD38br %B cell | Weighted mode | 20 | 0.05062541 | 0.03633102 | 0.179572033 | 1.05(0.98 to 1.13) |
| CD62L− monocyte AC | MR Egger | 20 | −0.06798541 | 0.03065734 | 0.039690606 | 0.93(0.88 to 0.99) |
| CD62L− monocyte AC | Weighted median | 20 | −0.07281590 | 0.03121466 | 0.019661420 | 0.93(0.87 to 0.99) |
| CD62L− monocyte AC | Inverse variance weighted | 20 | −0.05063867 | 0.02267360 | 0.025524201 | 0.95(0.91 to 0.99) |
| CD62L− monocyte AC | Simple mode | 20 | −0.05540208 | 0.04147573 | 0.197405477 | 0.95(0.87 to 1.03) |
| CD62L− monocyte AC | Weighted mode | 20 | −0.07184082 | 0.02767974 | 0.017757496 | 0.93(0.88 to 0.98) |
| CD62L− HLA DR++ monocyte AC | MR Egger | 20 | −0.08149514 | 0.02863537 | 0.010724529 | 0.92(0.87 to 0.97) |
| CD62L− HLA DR++ monocyte AC | Weighted median | 20 | −0.07760637 | 0.02952767 | 0.008582303 | 0.93(0.87 to 0.98) |
| CD62L− HLA DR++ monocyte AC | Inverse variance weighted | 20 | −0.05650765 | 0.02133336 | 0.008077998 | 0.95(0.91 to 0.99) |
| CD62L− HLA DR++ monocyte AC | Simple mode | 20 | −0.07019678 | 0.04669835 | 0.149228538 | 0.93(0.85 to 1.02) |
| CD62L− HLA DR++ monocyte AC | Weighted mode | 20 | −0.07702977 | 0.02829508 | 0.013519969 | 0.93(0.88 to 0.98) |
| CD62L− HLA DR++ monocyte %monocyte | MR Egger | 20 | −0.06184749 | 0.02919420 | 0.048305972 | 0.94(0.89 to 1.00) |
| CD62L− HLA DR++ monocyte %monocyte | Weighted median | 20 | −0.04467961 | 0.03177426 | 0.159677497 | 0.96(0.90 to 1.02) |
| CD62L− HLA DR++ monocyte %monocyte | Inverse variance weighted | 20 | −0.04475217 | 0.02174563 | 0.039591571 | 0.96(0.92 to 1.00) |
| CD62L− HLA DR++ monocyte %monocyte | Simple mode | 20 | −0.07721380 | 0.05123754 | 0.148263230 | 0.93(0.84 to 1.02) |
| CD62L− HLA DR++ monocyte %monocyte | Weighted mode | 20 | −0.04174465 | 0.02763400 | 0.147336449 | 0.96(0.91 to 1.01) |
| Naive DN (CD4−CD8−) %DN | MR Egger | 20 | 0.03278738 | 0.02509536 | 0.207826827 | 1.03(0.98 to 1.09) |
| Naive DN (CD4−CD8−) %DN | Weighted median | 20 | 0.03470539 | 0.02573802 | 0.177526767 | 1.04(0.98 to 1.09) |
| Naive DN (CD4−CD8−) %DN | Inverse variance weighted | 20 | 0.03958026 | 0.01856018 | 0.032962764 | 1.04(1.00 to 1.08) |
| Naive DN (CD4−CD8−) %DN | Simple mode | 20 | 0.05443078 | 0.03982421 | 0.187651801 | 1.06(0.98 to 1.14) |
| Naive DN (CD4−CD8−) %DN | Weighted mode | 20 | 0.03729787 | 0.02433030 | 0.141763391 | 1.04(0.99 to 1.09) |
| CD28+ CD45RA+ CD8dim %CD8dim | MR Egger | 20 | 0.05446331 | 0.02707564 | 0.059487097 | 1.06(1.00 to 1.11) |
| CD28+ CD45RA+ CD8dim %CD8dim | Weighted median | 20 | 0.03559100 | 0.03090874 | 0.249532182 | 1.04(0.98 to 1.10) |
| CD28+ CD45RA+ CD8dim %CD8dim | Inverse variance weighted | 20 | 0.04472626 | 0.02016986 | 0.026590319 | 1.05(1.01 to 1.09) |
| CD28+ CD45RA+ CD8dim %CD8dim | Simple mode | 20 | 0.01776663 | 0.04540907 | 0.699960149 | 1.02(0.93 to 1.11) |
| CD28+ CD45RA+ CD8dim %CD8dim | Weighted mode | 20 | 0.03799059 | 0.03391030 | 0.276536988 | 1.04(0.97 to 1.11) |
| CD28+ CD45RA+ CD8dim AC | MR Egger | 20 | 0.05202402 | 0.02434137 | 0.046552430 | 1.05(1.00 to 1.10) |
| CD28+ CD45RA+ CD8dim AC | Weighted median | 20 | 0.05657923 | 0.02586377 | 0.028699767 | 1.06(1.01 to 1.11) |
| CD28+ CD45RA+ CD8dim AC | Inverse variance weighted | 20 | 0.03644414 | 0.01812089 | 0.044307805 | 1.04(1.00 to 1.07) |
| CD28+ CD45RA+ CD8dim AC | Simple mode | 20 | 0.02607889 | 0.04174257 | 0.539562680 | 1.03(0.95 to 1.11) |
| CD28+ CD45RA+ CD8dim AC | Weighted mode | 20 | 0.04872757 | 0.02410838 | 0.057577703 | 1.05(1.00 to 1.10) |
| CD45RA+ CD28− CD8br %T cell | MR Egger | 20 | 0.33062813 | 0.17064430 | 0.068533174 | 1.39(1.00 to 1.94) |
| CD45RA+ CD28− CD8br %T cell | Weighted median | 20 | 0.22167940 | 0.19433273 | 0.253986090 | 1.25(0.85 to 1.83) |
| CD45RA+ CD28− CD8br %T cell | Inverse variance weighted | 20 | 0.27293705 | 0.12464037 | 0.028538998 | 1.31(1.03 to 1.68) |
| CD45RA+ CD28− CD8br %T cell | Simple mode | 20 | 0.51899975 | 0.32699255 | 0.128971887 | 1.68(0.89 to 3.19) |
| CD45RA+ CD28− CD8br %T cell | Weighted mode | 20 | 0.11274714 | 0.21110777 | 0.599484292 | 1.12(0.74 to 1.69) |
| CD24 on IgD+ CD38− | MR Egger | 20 | −0.05394394 | 0.02801096 | 0.070072567 | 0.95(0.90 to 1.00) |
| CD24 on IgD+ CD38− | Weighted median | 20 | −0.03288365 | 0.02995272 | 0.272269158 | 0.97(0.91 to 1.03) |
| CD24 on IgD+ CD38− | Inverse variance weighted | 20 | −0.04448109 | 0.02074511 | 0.032019048 | 0.96(0.92 to 1.00) |
| CD24 on IgD+ CD38− | Simple mode | 20 | −0.04471373 | 0.04609687 | 0.344236010 | 0.96(0.87 to 1.05) |
| CD24 on IgD+ CD38− | Weighted mode | 20 | −0.02906498 | 0.02910014 | 0.330446715 | 0.97(0.92 to 1.03) |
| CD24 on IgD− CD38− | MR Egger | 20 | −0.07062590 | 0.02802573 | 0.021396888 | 0.93(0.88 to 0.98) |
| CD24 on IgD− CD38− | Weighted median | 20 | −0.04703973 | 0.02966017 | 0.112749272 | 0.95(0.90 to 1.01) |
| CD24 on IgD− CD38− | Inverse variance weighted | 20 | −0.05262742 | 0.02075347 | 0.011217877 | 0.95(0.91 to 0.99) |
| CD24 on IgD− CD38− | Simple mode | 20 | −0.07071653 | 0.04195746 | 0.108258769 | 0.93(0.86 to 1.01) |
| CD24 on IgD− CD38− | Weighted mode | 20 | −0.05269408 | 0.02891283 | 0.084154685 | 0.95(0.90 to 1.00) |
| CD24 on memory B cell | MR Egger | 20 | −0.03972844 | 0.02762093 | 0.167496742 | 0.96(0.91 to 1.01) |
| CD24 on memory B cell | Weighted median | 20 | −0.03247633 | 0.02856009 | 0.255486988 | 0.97(0.92 to 1.02) |
| CD24 on memory B cell | Inverse variance weighted | 20 | −0.04254815 | 0.02045347 | 0.037503471 | 0.96(0.92 to 1.00) |
| CD24 on memory B cell | Simple mode | 20 | −0.04983071 | 0.04149025 | 0.244501453 | 0.95(0.88 to 1.03) |
| CD24 on memory B cell | Weighted mode | 20 | −0.03501220 | 0.02736646 | 0.216165880 | 0.97(0.92 to 1.02) |
| CD24 on unsw mem | MR Egger | 20 | −0.06066552 | 0.02768288 | 0.041814867 | 0.94(0.89 to 0.99) |
| CD24 on unsw mem | Weighted median | 20 | −0.05235191 | 0.02884397 | 0.069523368 | 0.95(0.90 to 1.00) |
| CD24 on unsw mem | Inverse variance weighted | 20 | −0.04762180 | 0.02050074 | 0.020182870 | 0.95(0.92 to 0.99) |
| CD24 on unsw mem | Simple mode | 20 | −0.06961801 | 0.04222919 | 0.115671541 | 0.93(0.86 to 1.01) |
| CD24 on unsw mem | Weighted mode | 20 | −0.05067087 | 0.03161660 | 0.125502990 | 0.95(0.89 to 1.01) |
| CD25 on CD20− CD38− | MR Egger | 20 | 0.03815809 | 0.02766872 | 0.184758732 | 1.04(0.98 to 1.10) |
| CD25 on CD20− CD38− | Weighted median | 20 | 0.03917247 | 0.03047048 | 0.198586937 | 1.04(0.98 to 1.10) |
| CD25 on CD20− CD38− | Inverse variance weighted | 20 | 0.04948667 | 0.02048424 | 0.015698896 | 1.05(1.01 to 1.09) |
| CD25 on CD20− CD38− | Simple mode | 20 | 0.06216962 | 0.04293428 | 0.163913175 | 1.06(0.98 to 1.16) |
| CD25 on CD20− CD38− | Weighted mode | 20 | 0.04147090 | 0.02805471 | 0.155736951 | 1.04(0.99 to 1.10) |
| CD25 on IgD+ CD24+ | MR Egger | 20 | −0.06157210 | 0.02744911 | 0.037711264 | 0.94(0.89 to 0.99) |
| CD25 on IgD+ CD24+ | Weighted median | 20 | −0.05663832 | 0.02865050 | 0.048056287 | 0.94(0.89 to 1.00) |
| CD25 on IgD+ CD24+ | Inverse variance weighted | 20 | −0.05068334 | 0.02033343 | 0.012680764 | 0.95(0.91 to 0.99) |
| CD25 on IgD+ CD24+ | Simple mode | 20 | −0.04965541 | 0.04534317 | 0.287160178 | 0.95(0.87 to 1.04) |
| CD25 on IgD+ CD24+ | Weighted mode | 20 | −0.05621114 | 0.02636548 | 0.046277546 | 0.95(0.90 to 1.00) |
| CD27 on IgD− CD38− | MR Egger | 20 | −0.04978757 | 0.02787452 | 0.090934651 | 0.95(0.90 to 1.00) |
| CD27 on IgD− CD38− | Weighted median | 20 | −0.04372160 | 0.03016131 | 0.147172222 | 0.96(0.90 to 1.02) |
| CD27 on IgD− CD38− | Inverse variance weighted | 20 | −0.04547310 | 0.02064823 | 0.027645786 | 0.96(0.92 to 1.00) |
| CD27 on IgD− CD38− | Simple mode | 20 | −0.12553955 | 0.04798057 | 0.016977509 | 0.88(0.80 to 0.97) |
| CD27 on IgD− CD38− | Weighted mode | 20 | −0.04095395 | 0.02702017 | 0.146060800 | 0.96(0.91 to 1.01) |
| CD27 on IgD− CD38br | MR Egger | 20 | −0.06465225 | 0.02792748 | 0.032621182 | 0.94(0.89 to 0.99) |
| CD27 on IgD− CD38br | Weighted median | 20 | −0.06594086 | 0.03151179 | 0.036386913 | 0.94(0.88 to 1.00) |
| CD27 on IgD− CD38br | Inverse variance weighted | 20 | −0.05658026 | 0.02066409 | 0.006179596 | 0.94(0.91 to 0.98) |
| CD27 on IgD− CD38br | Simple mode | 20 | −0.04889097 | 0.04937748 | 0.334545189 | 0.95(0.86 to 1.05) |
| CD27 on IgD− CD38br | Weighted mode | 20 | −0.06588299 | 0.03585918 | 0.081862733 | 0.94(0.87 to 1.00) |
| CD38 on IgD+ CD38br | MR Egger | 20 | 0.05068053 | 0.03412442 | 0.154802220 | 1.05(0.98 to 1.12) |
| CD38 on IgD+ CD38br | Weighted median | 20 | 0.06429372 | 0.03015062 | 0.032972532 | 1.07(1.01 to 1.13) |
| CD38 on IgD+ CD38br | Inverse variance weighted | 20 | 0.05050261 | 0.02458379 | 0.039946103 | 1.05(1.00 to 1.10) |
| CD38 on IgD+ CD38br | Simple mode | 20 | 0.07028182 | 0.04693188 | 0.150685242 | 1.07(0.98 to 1.18) |
| CD38 on IgD+ CD38br | Weighted mode | 20 | 0.05522444 | 0.03105502 | 0.091365830 | 1.06(0.99 to 1.12) |
| CD38 on transitional | MR Egger | 20 | 0.04176515 | 0.02770764 | 0.149070663 | 1.04(0.99 to 1.10) |
| CD38 on transitional | Weighted median | 20 | 0.02878407 | 0.03125783 | 0.357123838 | 1.03(0.97 to 1.09) |
| CD38 on transitional | Inverse variance weighted | 20 | 0.05281998 | 0.02050982 | 0.010013850 | 1.05(1.01 to 1.10) |
| CD38 on transitional | Simple mode | 20 | 0.01865736 | 0.04801293 | 0.701899365 | 1.02(0.93 to 1.12) |
| CD38 on transitional | Weighted mode | 20 | 0.03647399 | 0.03042252 | 0.245301065 | 1.04(0.98 to 1.10) |
| HLA DR on CD14− CD16− | MR Egger | 20 | −0.03966037 | 0.02789819 | 0.172234715 | 0.96(0.91 to 1.02) |
| HLA DR on CD14− CD16− | Weighted median | 20 | −0.05054115 | 0.02941705 | 0.085780170 | 0.95(0.90 to 1.01) |
| HLA DR on CD14− CD16− | Inverse variance weighted | 20 | −0.05366989 | 0.02066038 | 0.009384477 | 0.95(0.91 to 0.99) |
| HLA DR on CD14− CD16− | Simple mode | 20 | −0.08440553 | 0.04743698 | 0.091190024 | 0.92(0.84 to 1.01) |
| HLA DR on CD14− CD16− | Weighted mode | 20 | −0.05314407 | 0.02983684 | 0.090880780 | 0.95(0.89 to 1.01) |
| CD39 on CD39+ activated Treg | MR Egger | 20 | −0.04871754 | 0.03323544 | 0.159945755 | 0.95(0.89 to 1.02) |
| CD39 on CD39+ activated Treg | Weighted median | 20 | −0.09487614 | 0.03509155 | 0.006857739 | 0.91(0.85 to 0.97) |
| CD39 on CD39+ activated Treg | Inverse variance weighted | 20 | −0.05741748 | 0.02428086 | 0.018043612 | 0.94(0.90 to 0.99) |
| CD39 on CD39+ activated Treg | Simple mode | 20 | −0.10071297 | 0.06596250 | 0.143281095 | 0.90(0.79 to 1.03) |
| CD39 on CD39+ activated Treg | Weighted mode | 20 | −0.08852413 | 0.04022763 | 0.040333868 | 0.92(0.85 to 0.99) |
| CD45 on CD33br HLA DR+ CD14− | MR Egger | 20 | 0.08765184 | 0.04273851 | 0.055125851 | 1.09(1.00 to 1.19) |
| CD45 on CD33br HLA DR+ CD14− | Weighted median | 20 | 0.06057439 | 0.04397185 | 0.168335522 | 1.06(0.97 to 1.16) |
| CD45 on CD33br HLA DR+ CD14− | Inverse variance weighted | 20 | 0.06754249 | 0.03142005 | 0.031581943 | 1.07(1.01 to 1.14) |
| CD45 on CD33br HLA DR+ CD14− | Simple mode | 20 | 0.10282895 | 0.06647989 | 0.138413996 | 1.11(0.97 to 1.26) |
| CD45 on CD33br HLA DR+ CD14− | Weighted mode | 20 | 0.06992455 | 0.04409902 | 0.129327196 | 1.07(0.98 to 1.17) |
| CD45 on CD33br HLA DR+ | MR Egger | 20 | 0.07086028 | 0.04267223 | 0.114118751 | 1.07(0.99 to 1.17) |
| CD45 on CD33br HLA DR+ | Weighted median | 20 | 0.02995657 | 0.04661705 | 0.520477267 | 1.03(0.94 to 1.13) |
| CD45 on CD33br HLA DR+ | Inverse variance weighted | 20 | 0.06603835 | 0.03137095 | 0.035284367 | 1.07(1.00 to 1.14) |
| CD45 on CD33br HLA DR+ | Simple mode | 20 | 0.08461507 | 0.07614056 | 0.280304240 | 1.09(0.94 to 1.26) |
| CD45 on CD33br HLA DR+ | Weighted mode | 20 | 0.03939850 | 0.04441725 | 0.386164469 | 1.04(0.95 to 1.13) |
| CD8 on CM CD8br | MR Egger | 20 | −0.03857112 | 0.02987166 | 0.212961440 | 0.96(0.91 to 1.02) |
| CD8 on CM CD8br | Weighted median | 20 | −0.04183405 | 0.03103618 | 0.177686581 | 0.96(0.90 to 1.02) |
| CD8 on CM CD8br | Inverse variance weighted | 20 | −0.05262466 | 0.02228084 | 0.018182538 | 0.95(0.91 to 0.99) |
| CD8 on CM CD8br | Simple mode | 20 | −0.02293355 | 0.05272075 | 0.668462968 | 0.98(0.88 to 1.08) |
| CD8 on CM CD8br | Weighted mode | 20 | −0.03962241 | 0.03201615 | 0.230949944 | 0.96(0.90 to 1.02) |

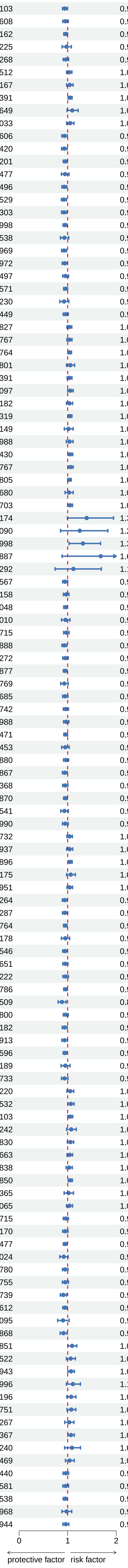


*P<0.05 was considered statistically significant*
