# Supplementary material for: Complex causal association between genetically predicted 731 immunocyte phenotype and osteonecrosis: a bidirectional two-sample Mendelian randomization analysis
Source: Int J Surg. 2024 Mar 18;110(6):3285–93. doi: 10.1097/JS9.0000000000001327 (PMC11175804; doi:10.1097/JS9.0000000000001327)
Supplement: Supplementary file 5 [file js9-110-3285-s005.pdf]

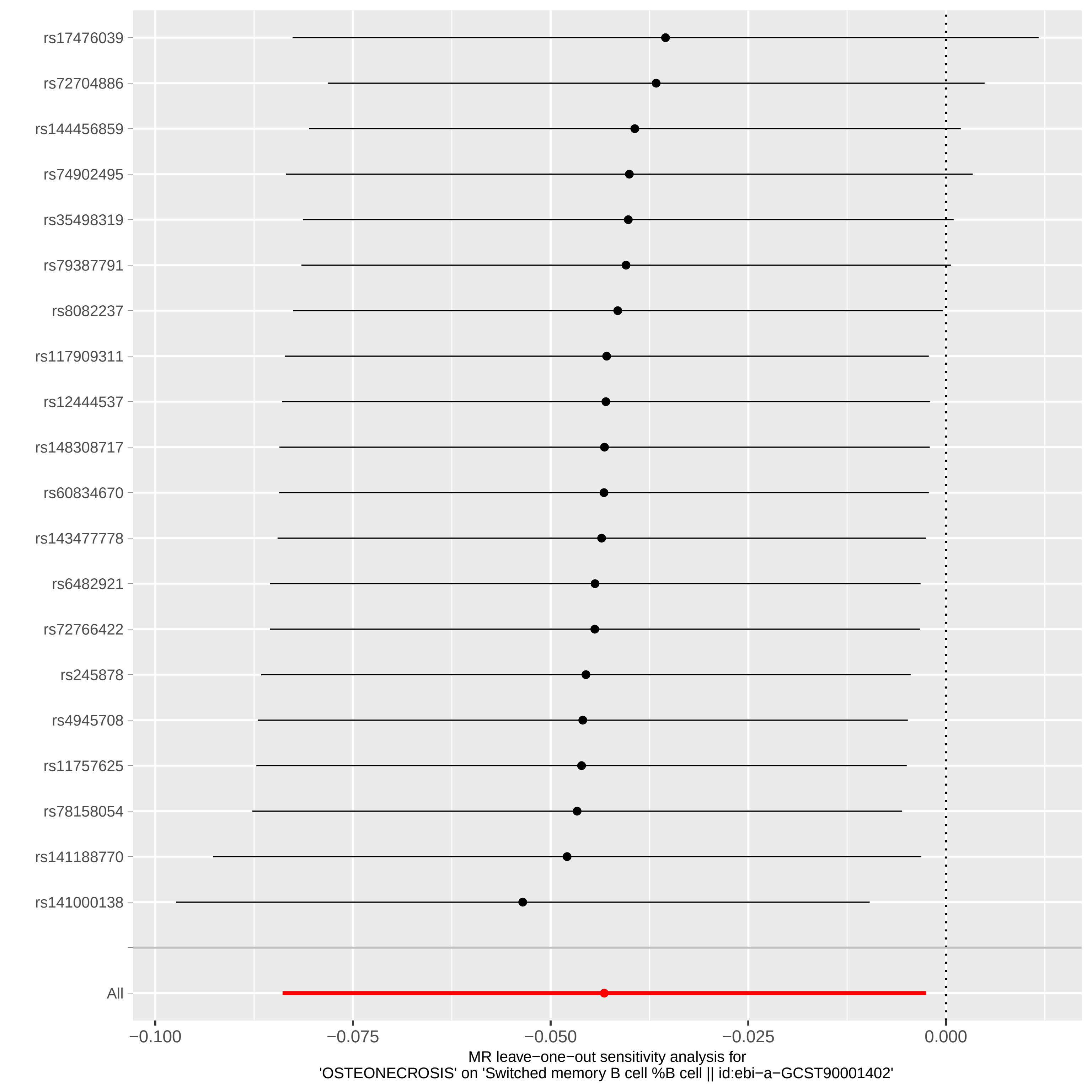
rs17476039
rs72704886
rs144456859
rs74902495
rs35498319
rs79387791
rs8082237
rs117909311
rs12444537
rs148308717
rs60834670
rs143477778
rs6482921
rs72766422
rs245878
rs4945708
rs11757625
rs78158054
rs141188770
rs141000138
All
−0.100
−0.075
−0.050
−0.025
0.000
MR leave−one−out sensitivity analysis for
'OSTEONECROSIS' on 'Switched memory B cell %B cell || id:ebi−a−GCST90001402'

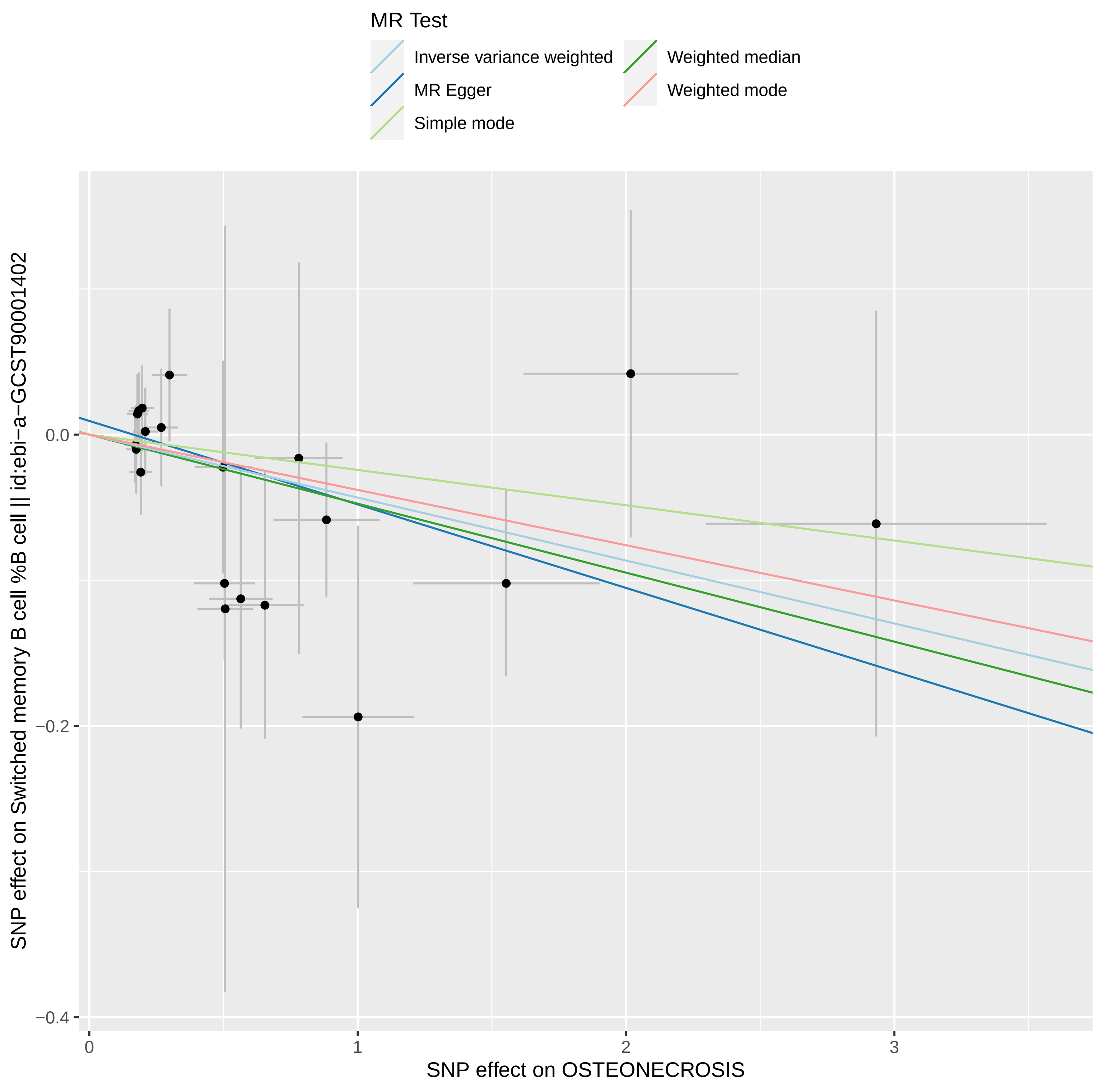

MR Test
Inverse variance weighted
MR Egger
Simple mode
Weighted median
Weighted mode
SNP effect on Switched memory B cell %B cell || id:ebi-a-GCST90001402
0.0
-0.2
-0.4
0
1
2
3
SNP effect on OSTEONECROSIS

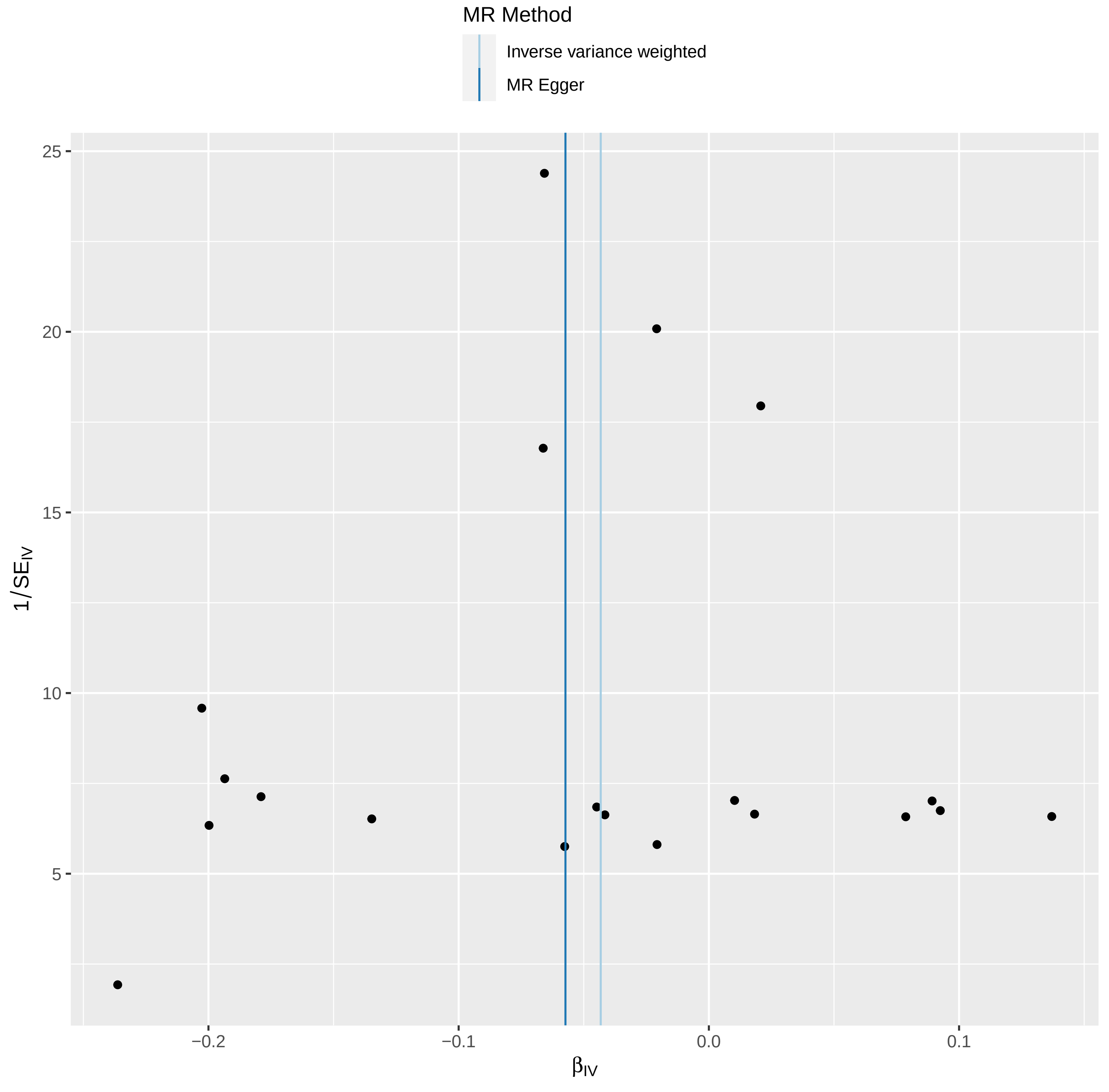
MR Method
Inverse variance weighted
MR Egger
25
20
15
10
5
1/SE_IV
−0.2
−0.1
0.0
0.1
β_IV

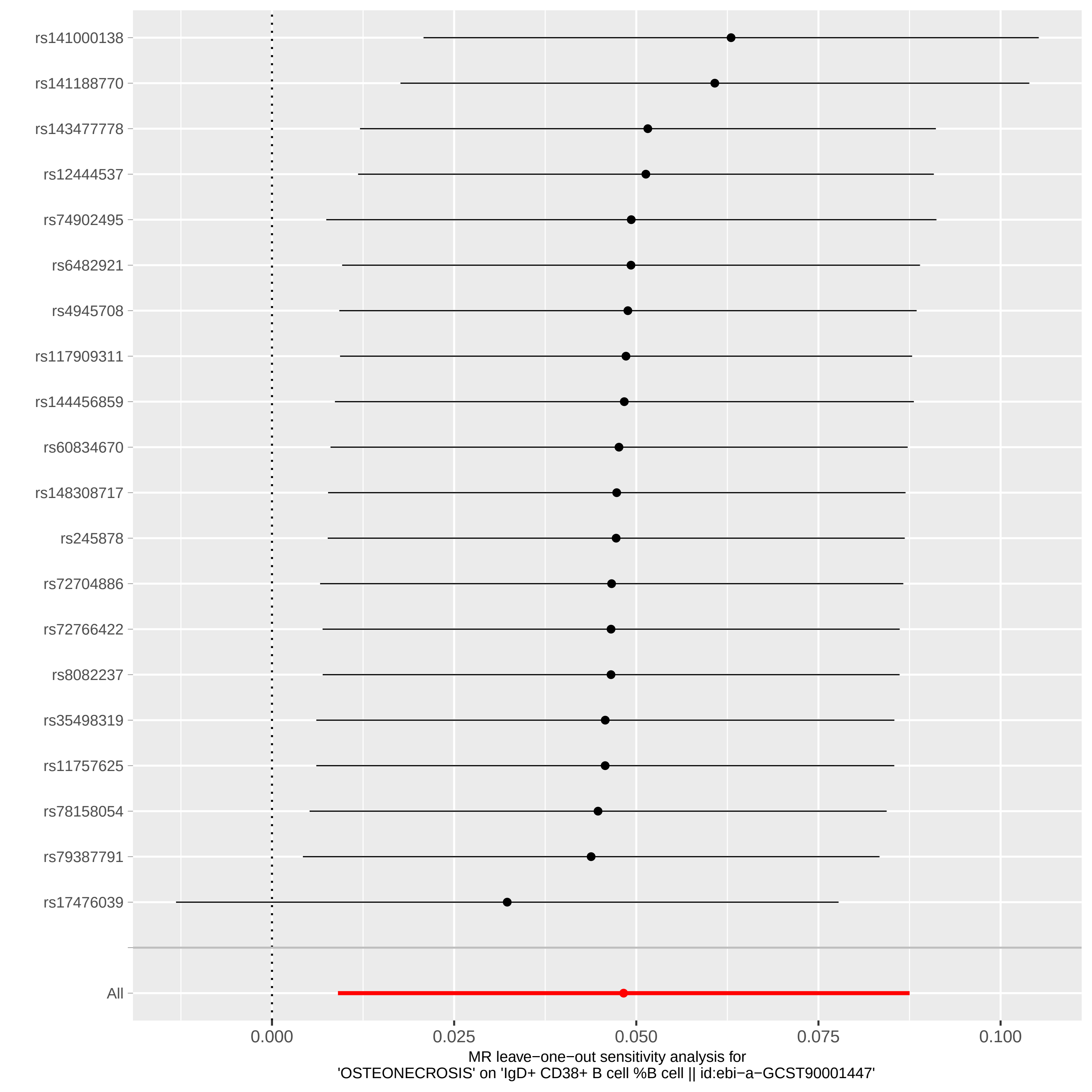
rs141000138
rs141188770
rs143477778
rs12444537
rs74902495
rs6482921
rs4945708
rs117909311
rs144456859
rs60834670
rs148308717
rs245878
rs72704886
rs72766422
rs8082237
rs35498319
rs11757625
rs78158054
rs79387791
rs17476039
All
0.000
0.025
0.050
0.075
0.100
MR leave−one−out sensitivity analysis for
'OSTEONECROSIS' on 'IgD+ CD38+ B cell %B cell || id:ebi−a−GCST90001447'

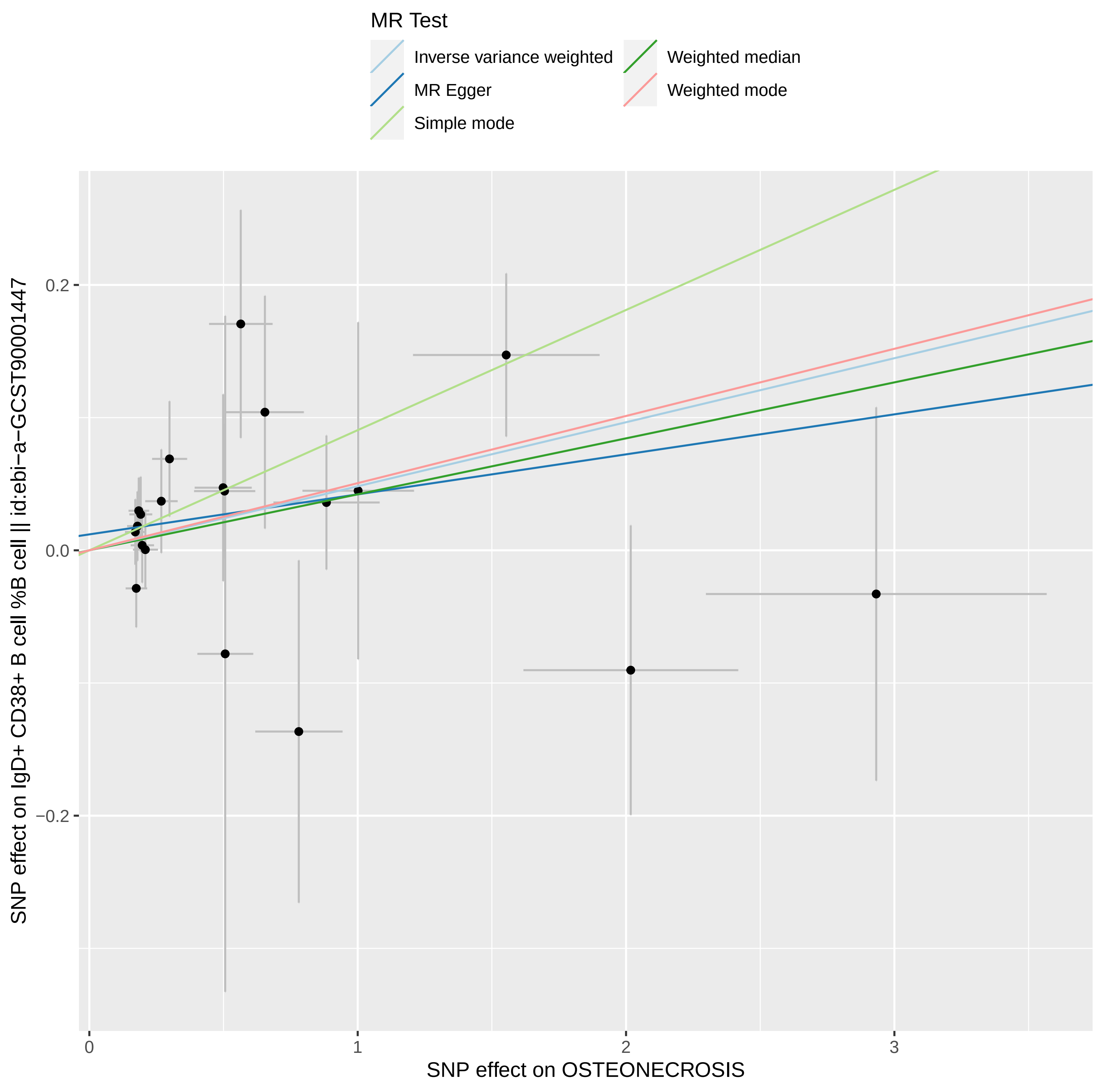

MR Test
Inverse variance weighted
MR Egger
Simple mode
Weighted median
Weighted mode
0.2
0.0
−0.2
0
1
2
3
SNP effect on IgD+ CD38+ B cell %B cell || id:ebi−a−GCST90001447
SNP effect on OSTEONECROSIS

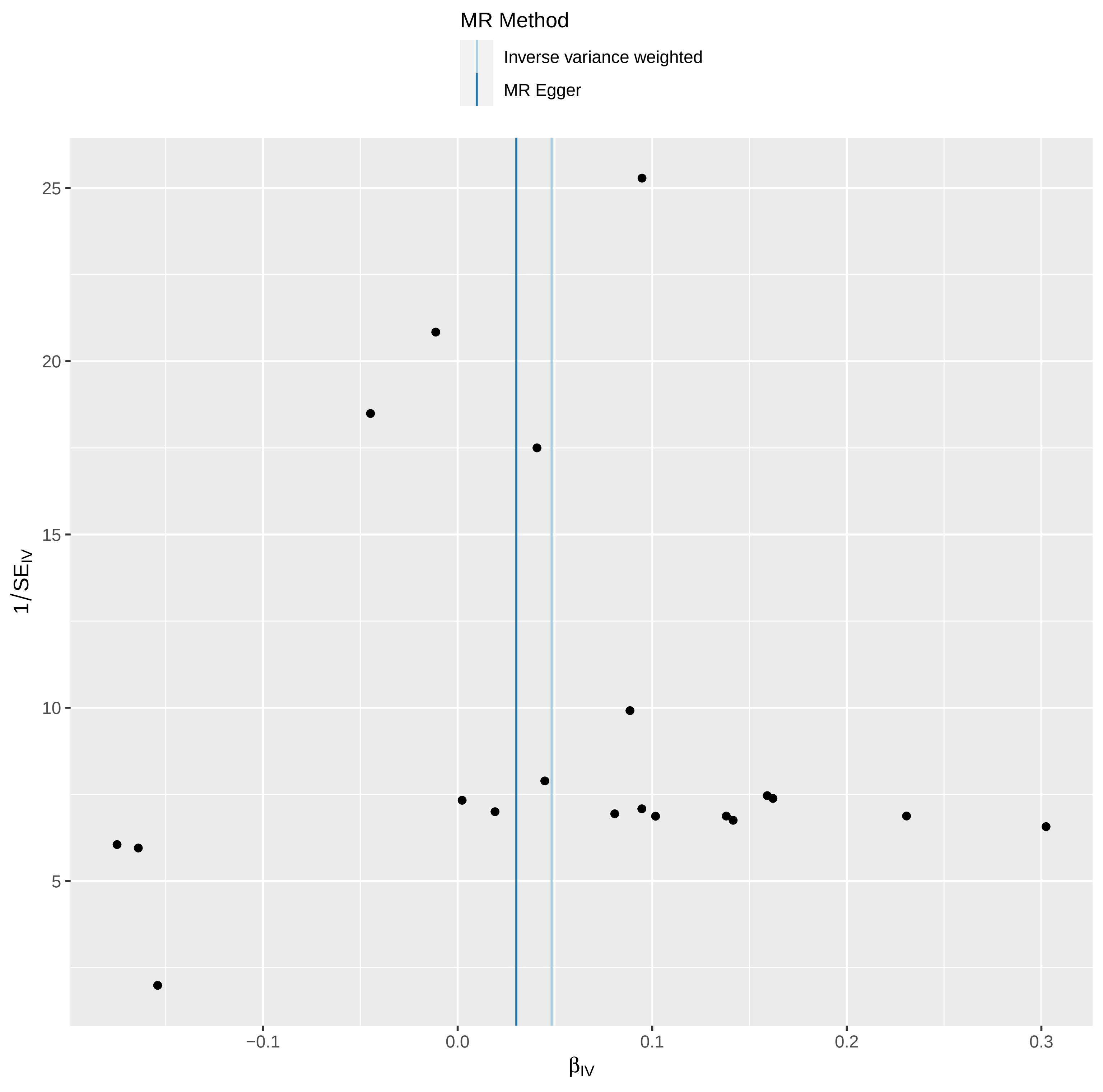

MR Method
Inverse variance weighted
MR Egger
25
20
15
10
5
1/SE_IV
−0.1
0.0
0.1
0.2
0.3
β_IV

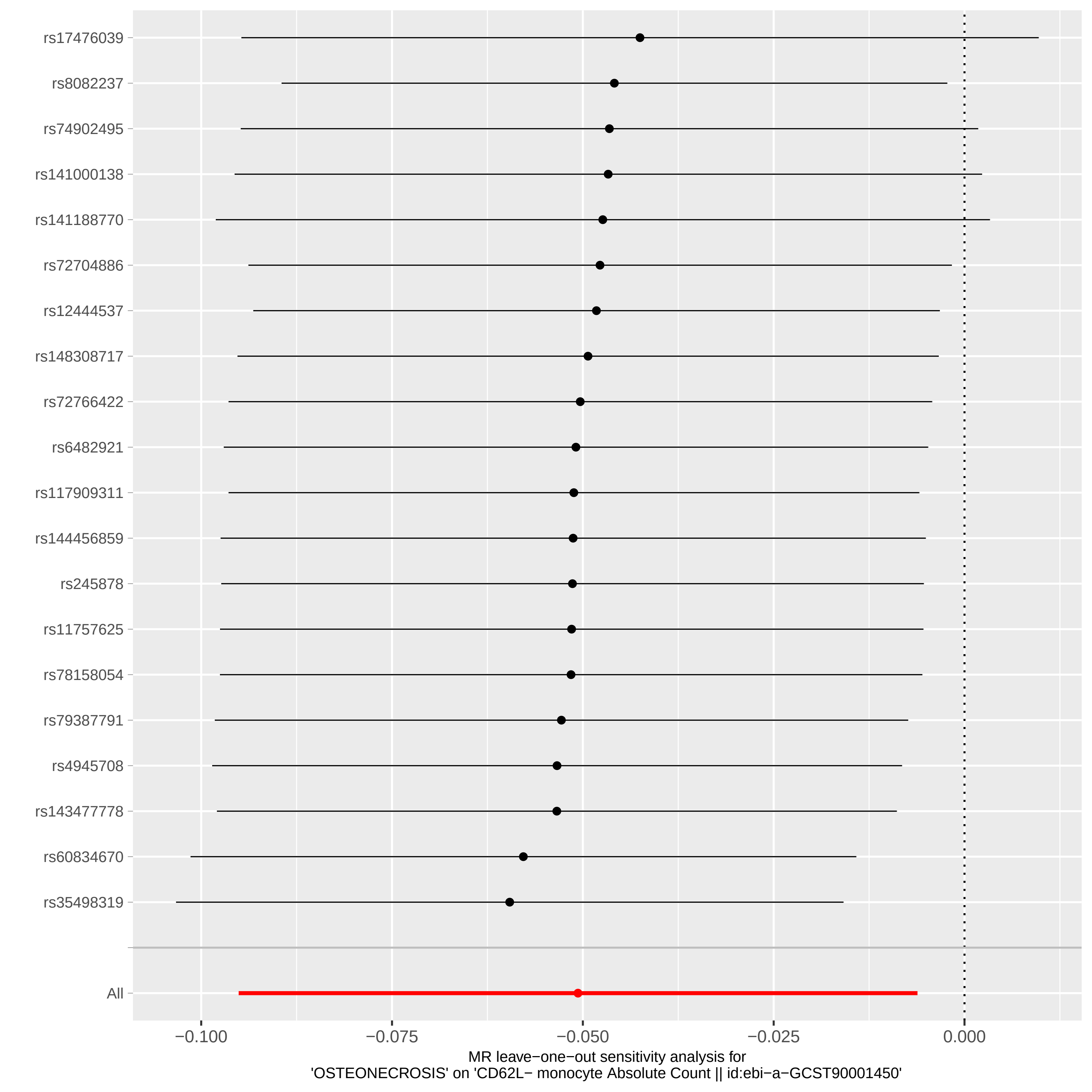
rs17476039
rs8082237
rs74902495
rs141000138
rs141188770
rs72704886
rs12444537
rs148308717
rs72766422
rs6482921
rs117909311
rs144456859
rs245878
rs11757625
rs78158054
rs79387791
rs4945708
rs143477778
rs60834670
rs35498319
All
−0.100
−0.075
−0.050
−0.025
0.000
MR leave−one−out sensitivity analysis for
'OSTEONECROSIS' on 'CD62L− monocyte Absolute Count || id:ebi−a−GCST90001450'

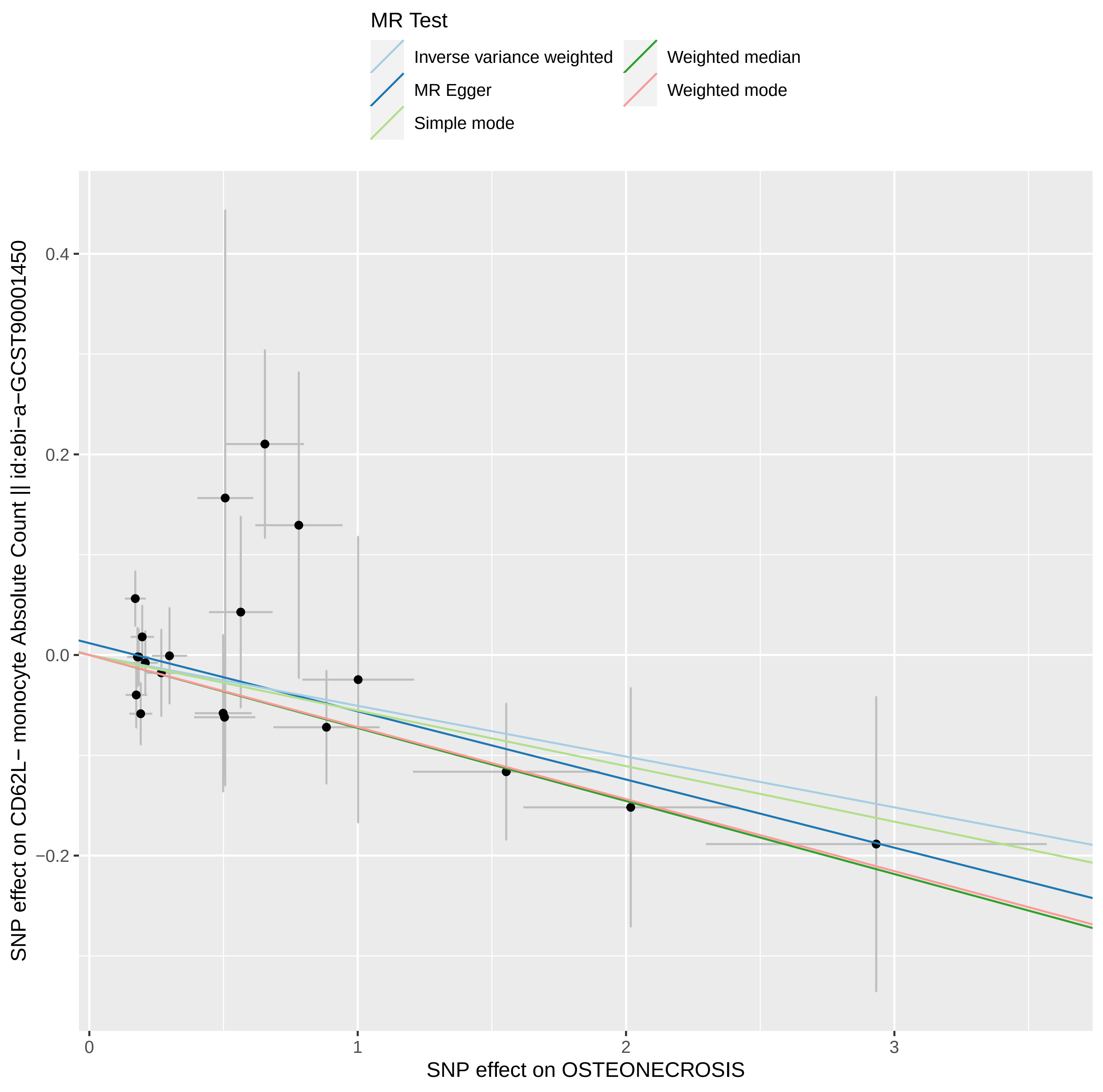
MR Test
Inverse variance weighted
MR Egger
Simple mode
Weighted median
Weighted mode
0.4
0.2
0.0
−0.2
0
1
2
3
SNP effect on CD62L− monocyte Absolute Count || id:ebi−a−GCST90001450
SNP effect on OSTEONECROSIS

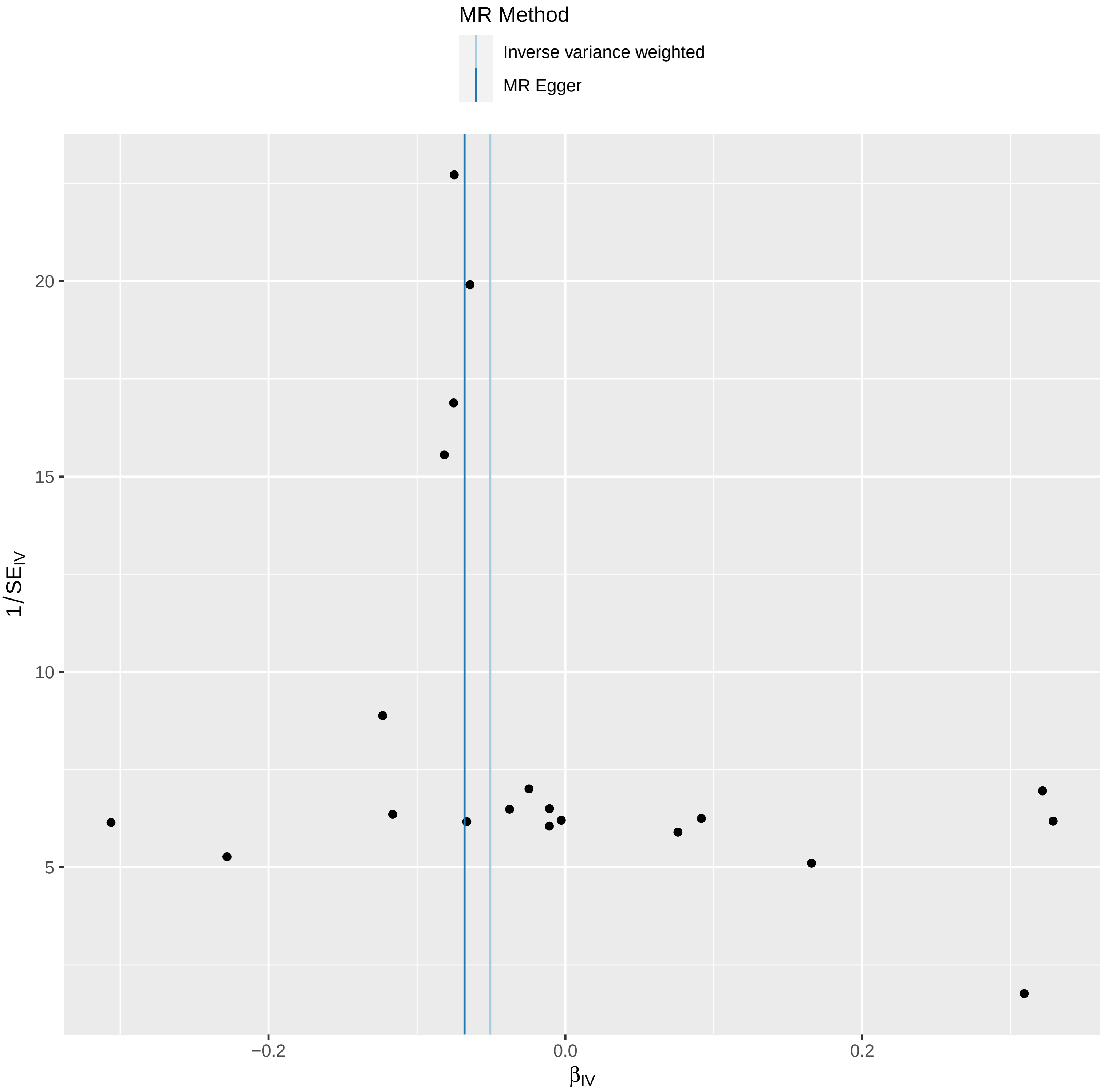
MR Method
Inverse variance weighted
MR Egger
1/SE_IV
20
15
10
5
−0.2
0.0
0.2
β_IV

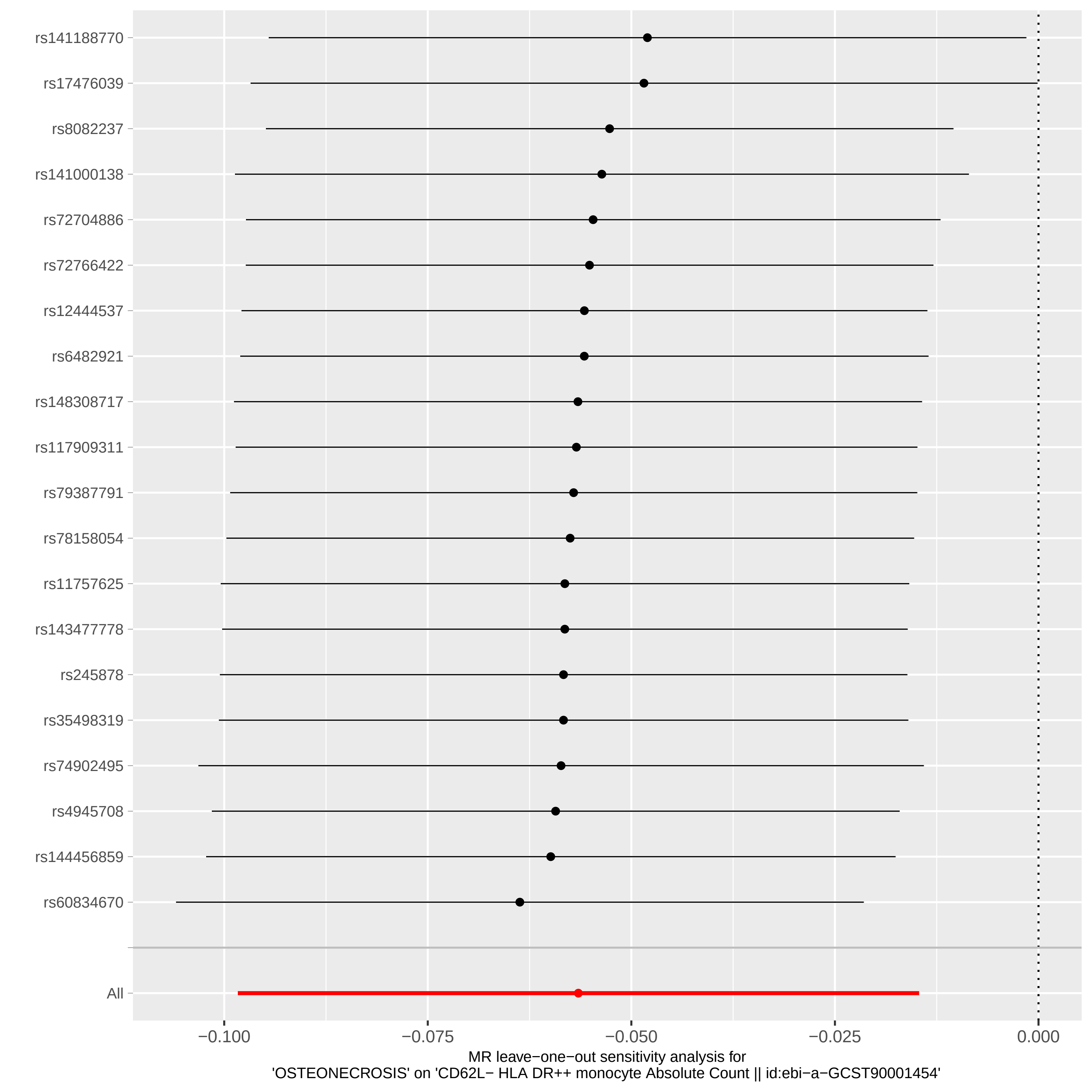

rs141188770
rs17476039
rs8082237
rs141000138
rs72704886
rs72766422
rs12444537
rs6482921
rs148308717
rs117909311
rs79387791
rs78158054
rs11757625
rs143477778
rs245878
rs35498319
rs74902495
rs4945708
rs144456859
rs60834670
All
−0.100
−0.075
−0.050
−0.025
0.000
MR leave−one−out sensitivity analysis for
'OSTEONECROSIS' on 'CD62L− HLA DR++ monocyte Absolute Count || id:ebi−a−GCST90001454'

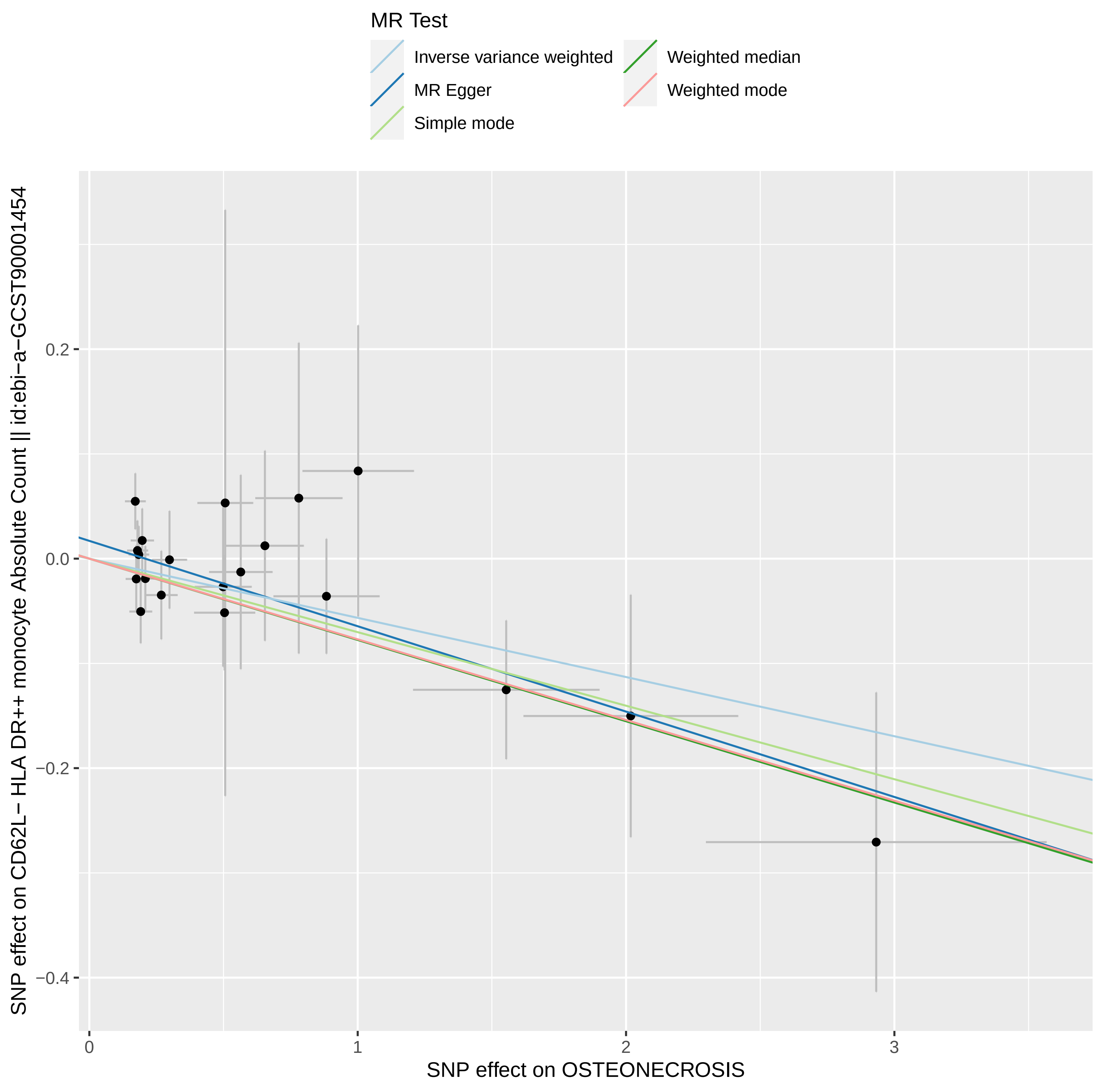
MR Test
Inverse variance weighted
MR Egger
Simple mode
Weighted median
Weighted mode
SNP effect on CD62L− HLA DR++ monocyte Absolute Count || id:ebi−a−GCST90001454
0.2
0.0
−0.2
−0.4
0
1
2
3
SNP effect on OSTEONECROSIS

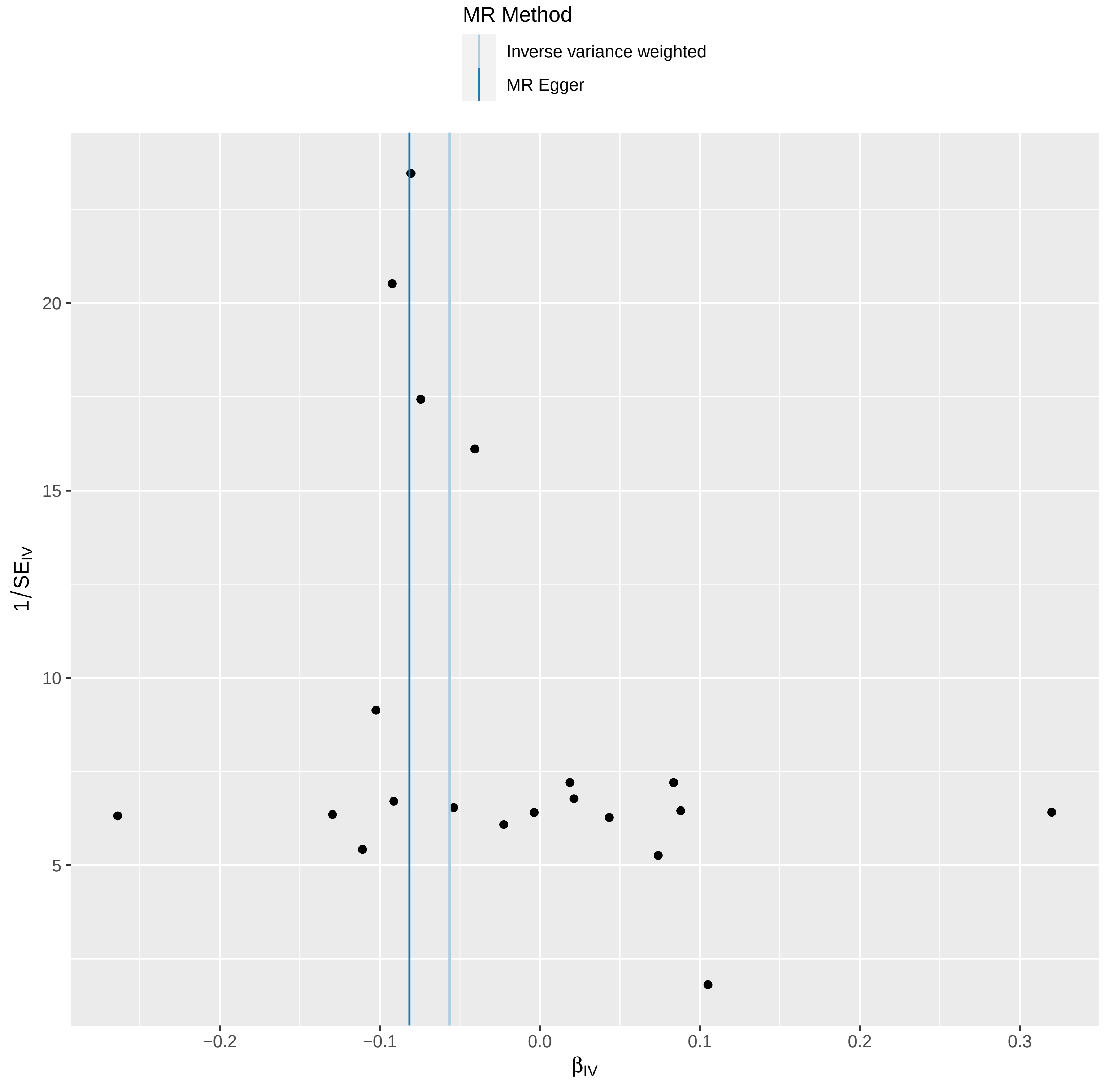
MR Method
Inverse variance weighted
MR Egger
20
15
10
5
−0.2
−0.1
0.0
0.1
0.2
0.3
$1/SE_{IV}$
β_{IV}

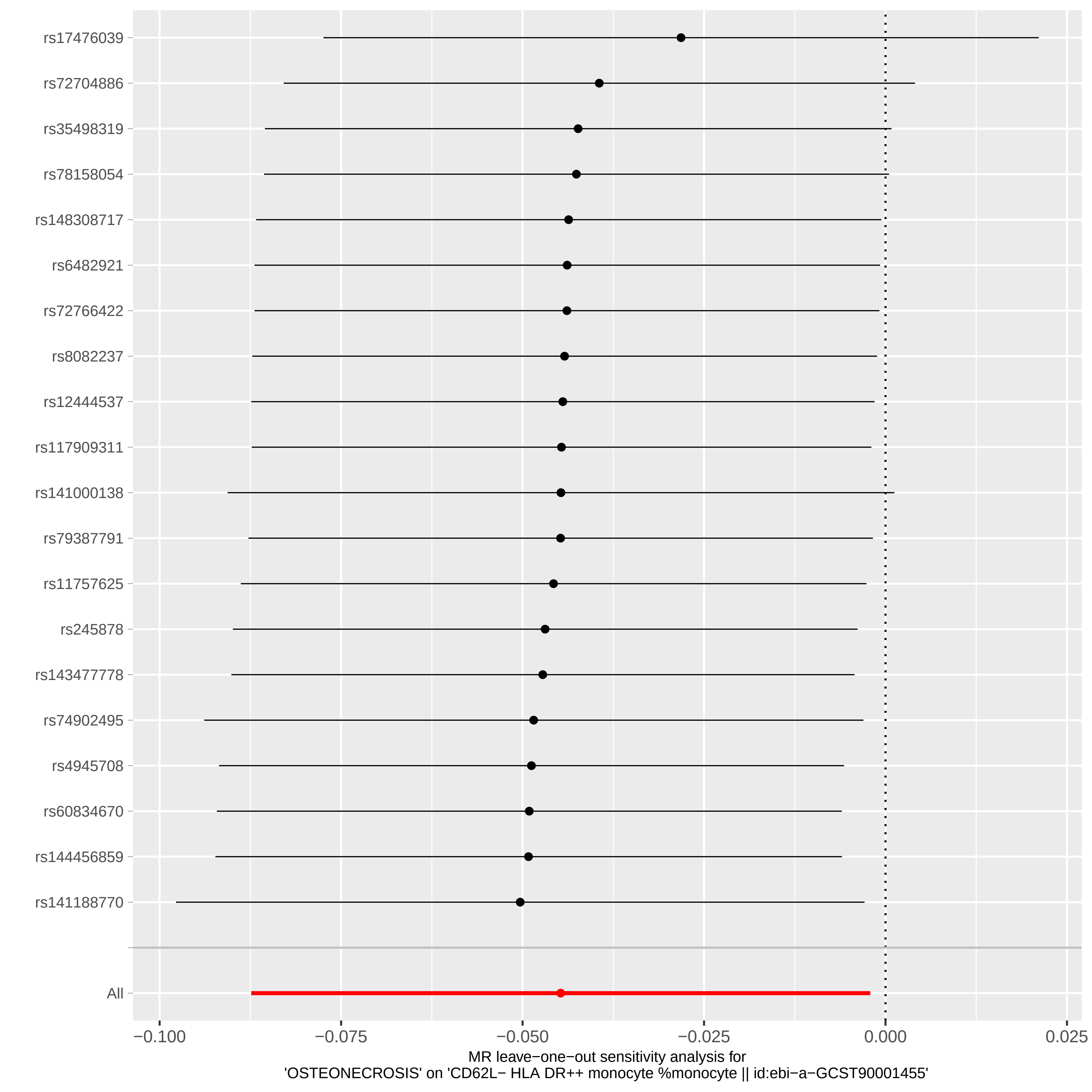
rs17476039
rs72704886
rs35498319
rs78158054
rs148308717
rs6482921
rs72766422
rs8082237
rs12444537
rs117909311
rs141000138
rs79387791
rs11757625
rs245878
rs143477778
rs74902495
rs4945708
rs60834670
rs144456859
rs141188770
All
−0.100
−0.075
−0.050
−0.025
0.000
0.025
MR leave−one−out sensitivity analysis for
'OSTEONECROSIS' on 'CD62L− HLA DR++ monocyte %monocyte || id:ebi−a−GCST90001455'

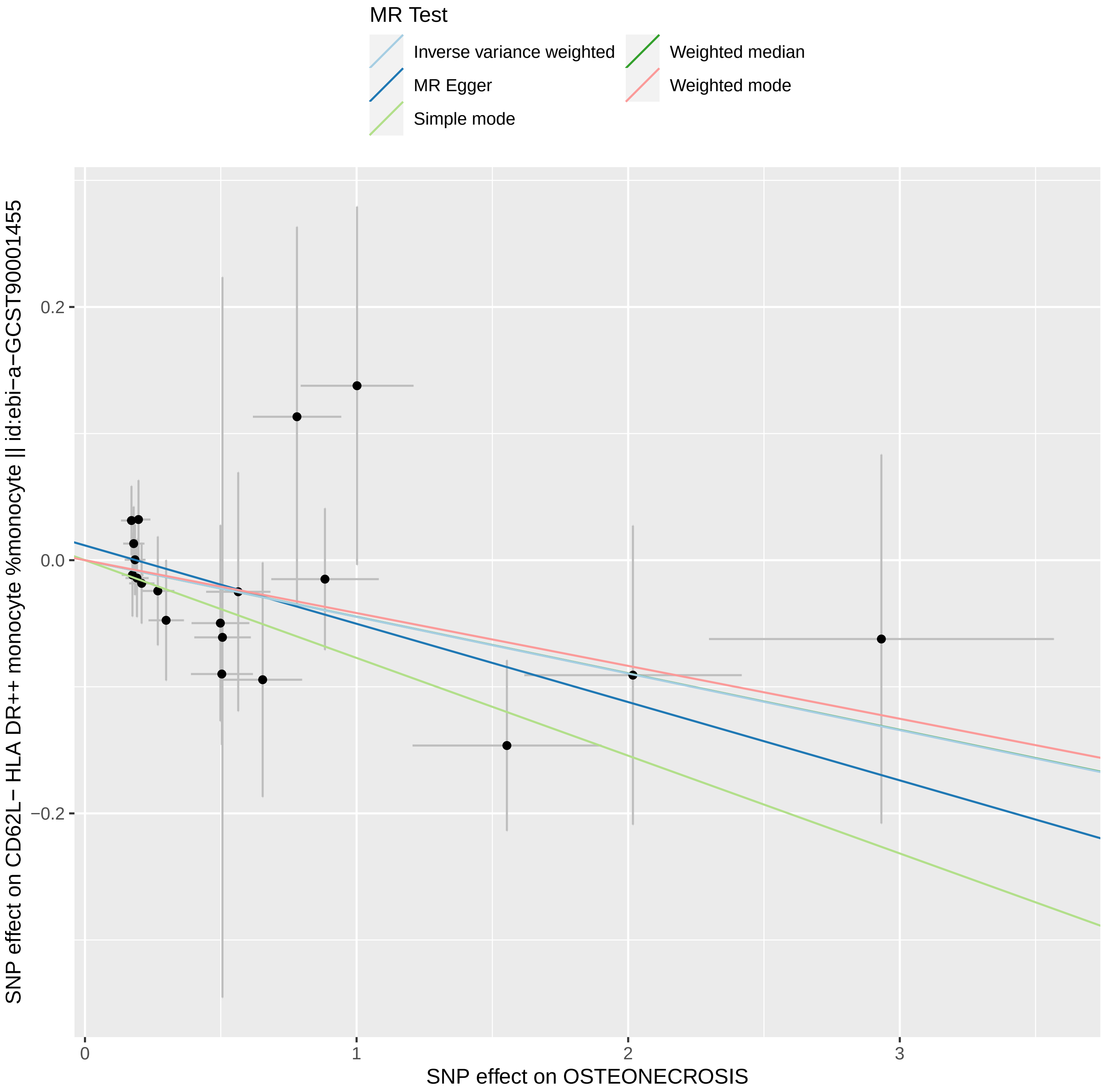
MR Test
Inverse variance weighted
MR Egger
Simple mode
Weighted median
Weighted mode
SNP effect on CD62L− HLA DR++ monocyte %monocyte || id:ebi−a−GCST90001455
0.2
0.0
−0.2
0
1
2
3
SNP effect on OSTEONECROSIS

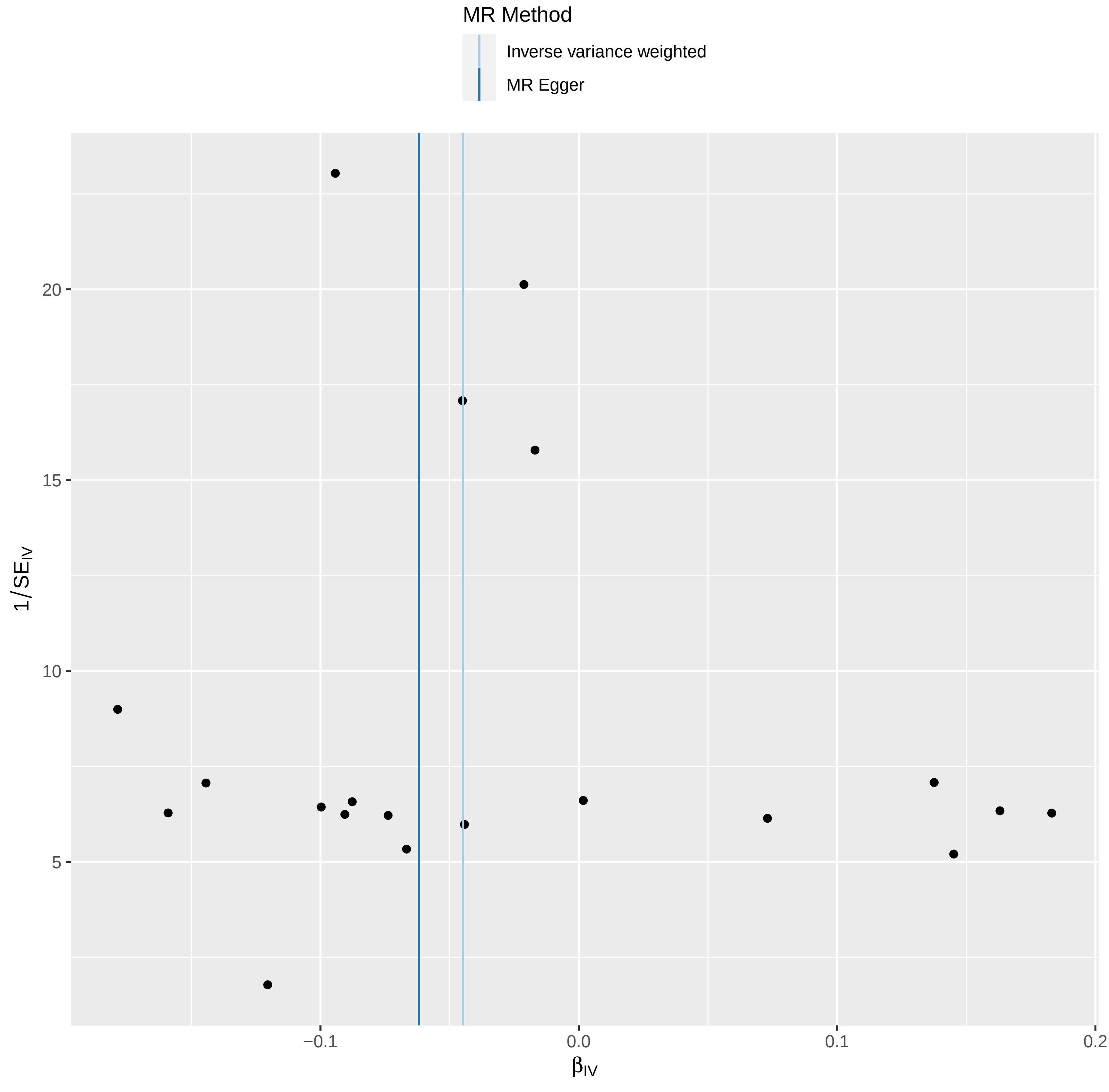
MR Method
Inverse variance weighted
MR Egger
20
15
10
5
1/SE_IV
−0.1
0.0
0.1
0.2
β_IV

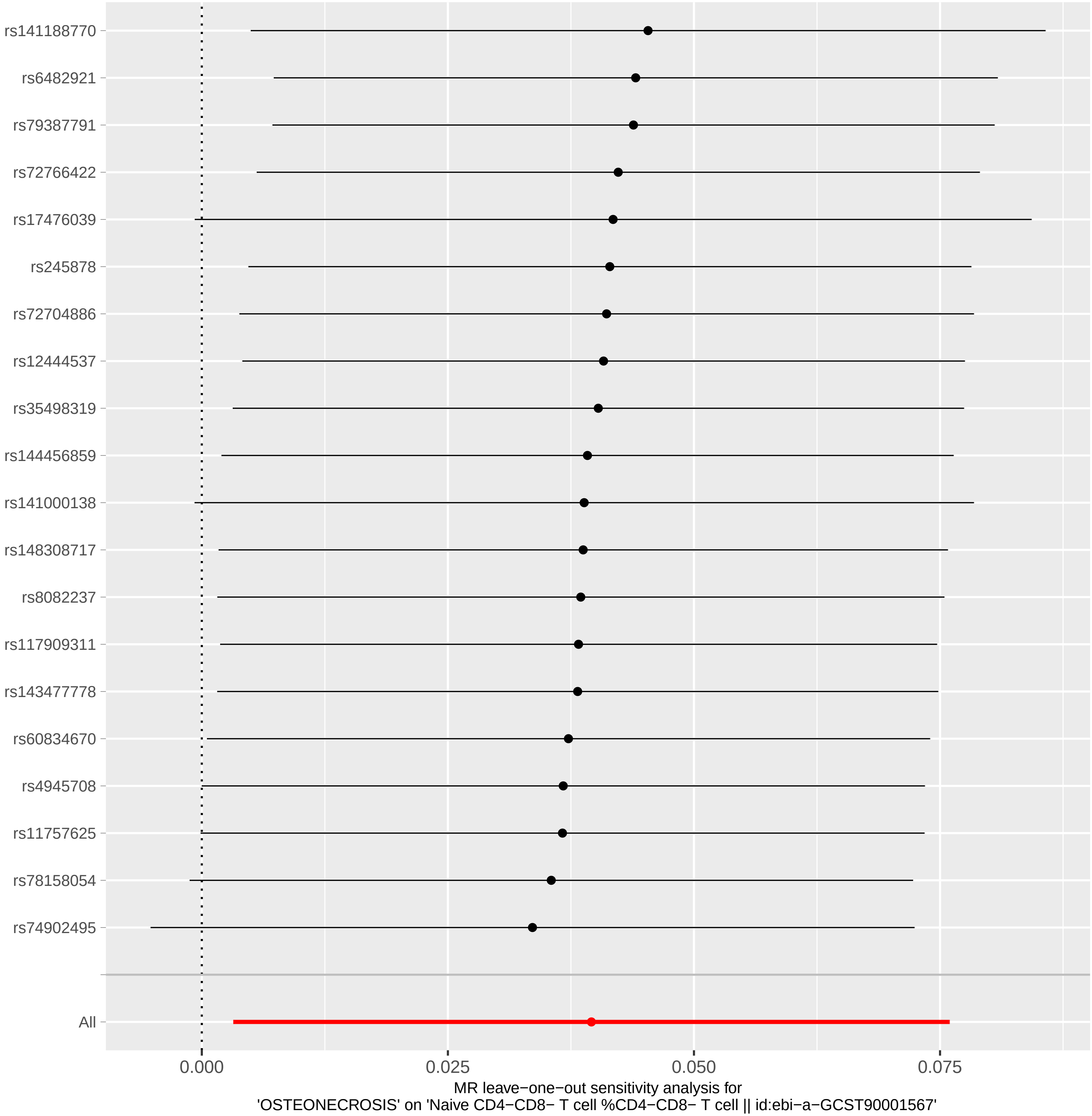
rs141188770
rs6482921
rs79387791
rs72766422
rs17476039
rs245878
rs72704886
rs12444537
rs35498319
rs144456859
rs141000138
rs148308717
rs8082237
rs117909311
rs143477778
rs60834670
rs4945708
rs11757625
rs78158054
rs74902495
All
0.000
0.025
0.050
0.075
MR leave−one−out sensitivity analysis for
'OSTEONECROSIS' on 'Naive CD4−CD8− T cell %CD4−CD8− T cell || id:ebi−a−GCST90001567'

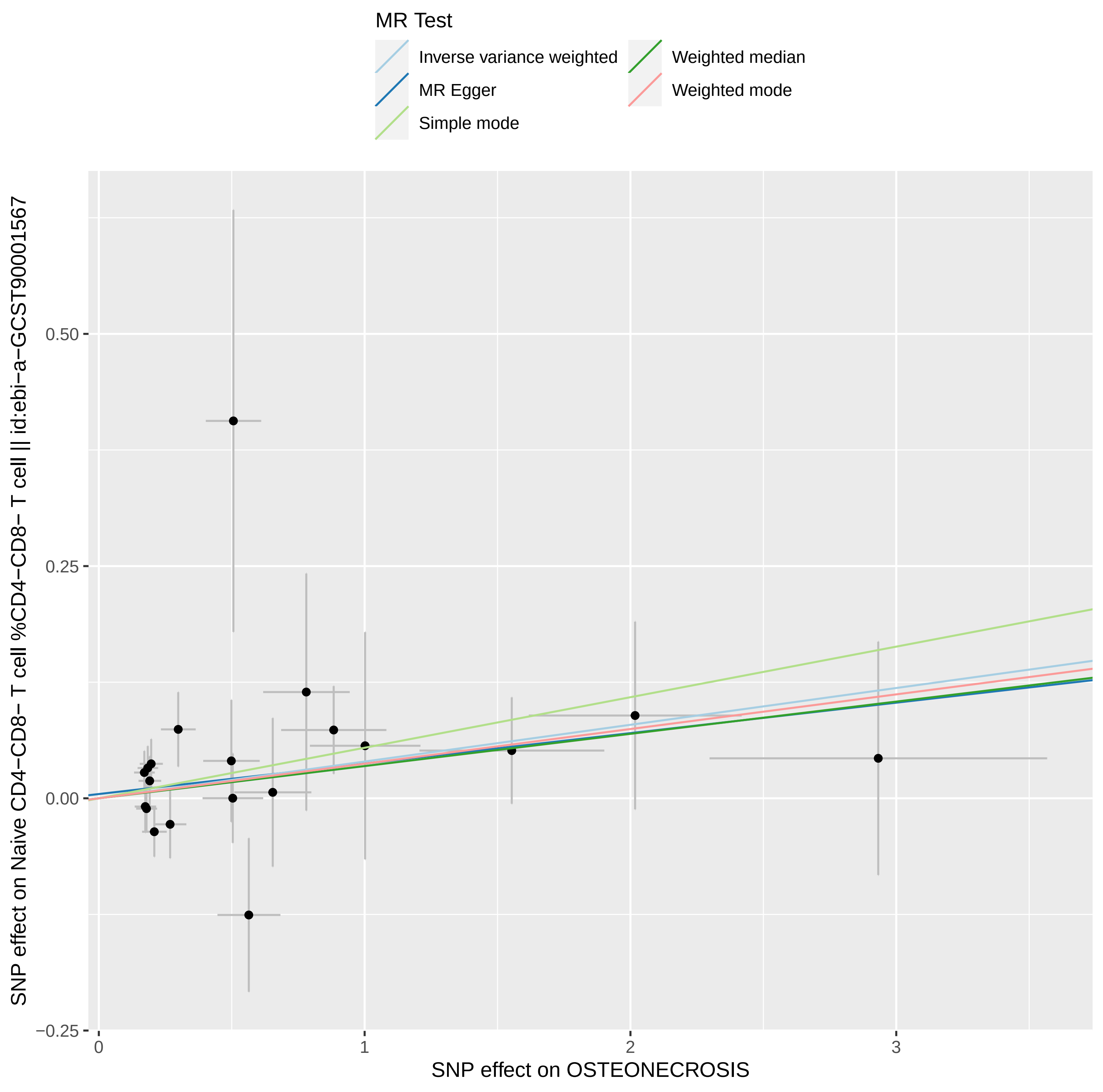
MR Test
Inverse variance weighted
MR Egger
Simple mode
Weighted median
Weighted mode
SNP effect on Naive CD4−CD8− T cell %CD4−CD8− T cell || id:ebi−a−GCST90001567
0.50
0.25
0.00
−0.25
0
1
2
3
SNP effect on OSTEONECROSIS

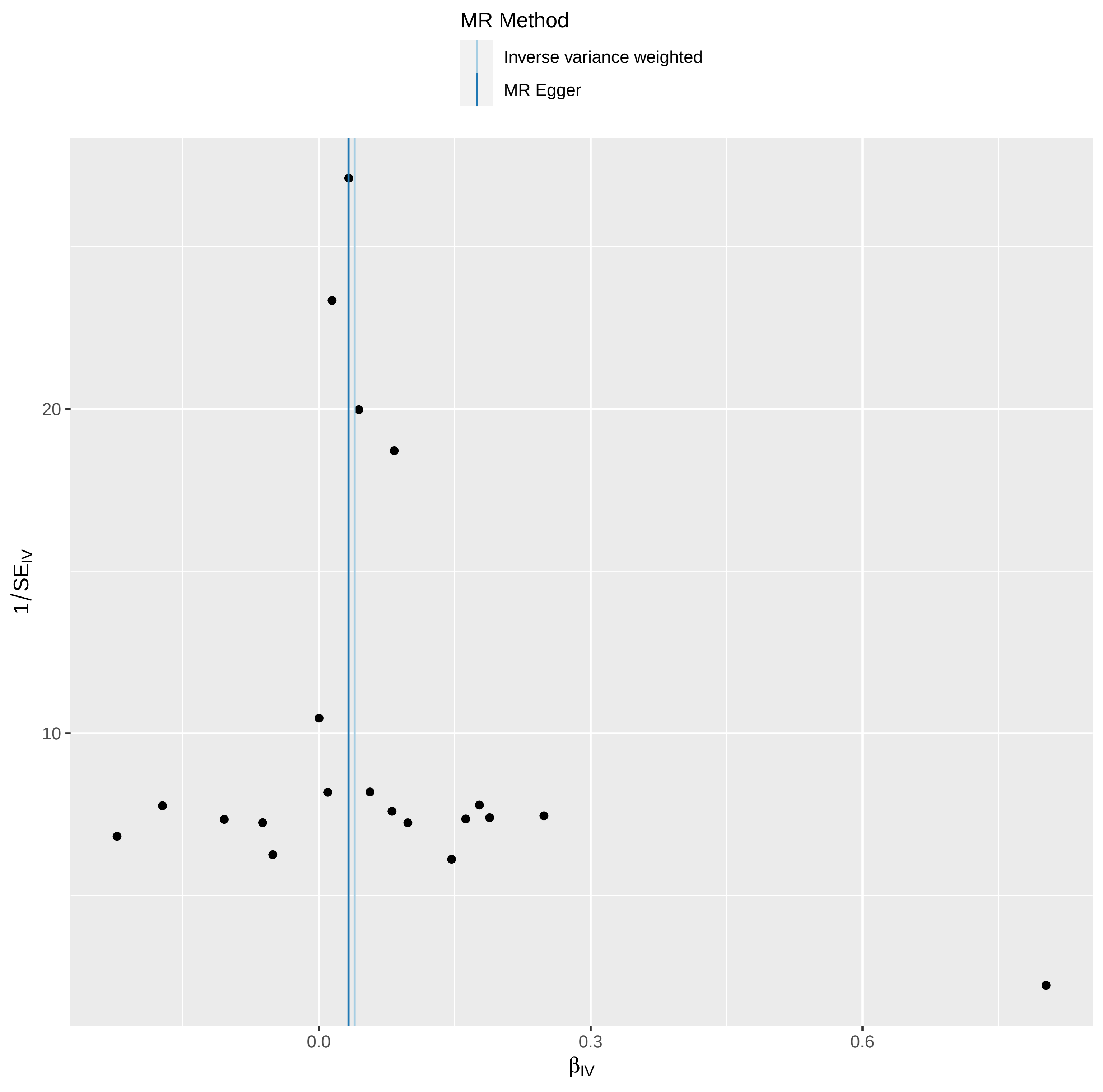

MR Method
Inverse variance weighted
MR Egger
20
10
1/SE_IV
0.0
0.3
0.6
β_IV

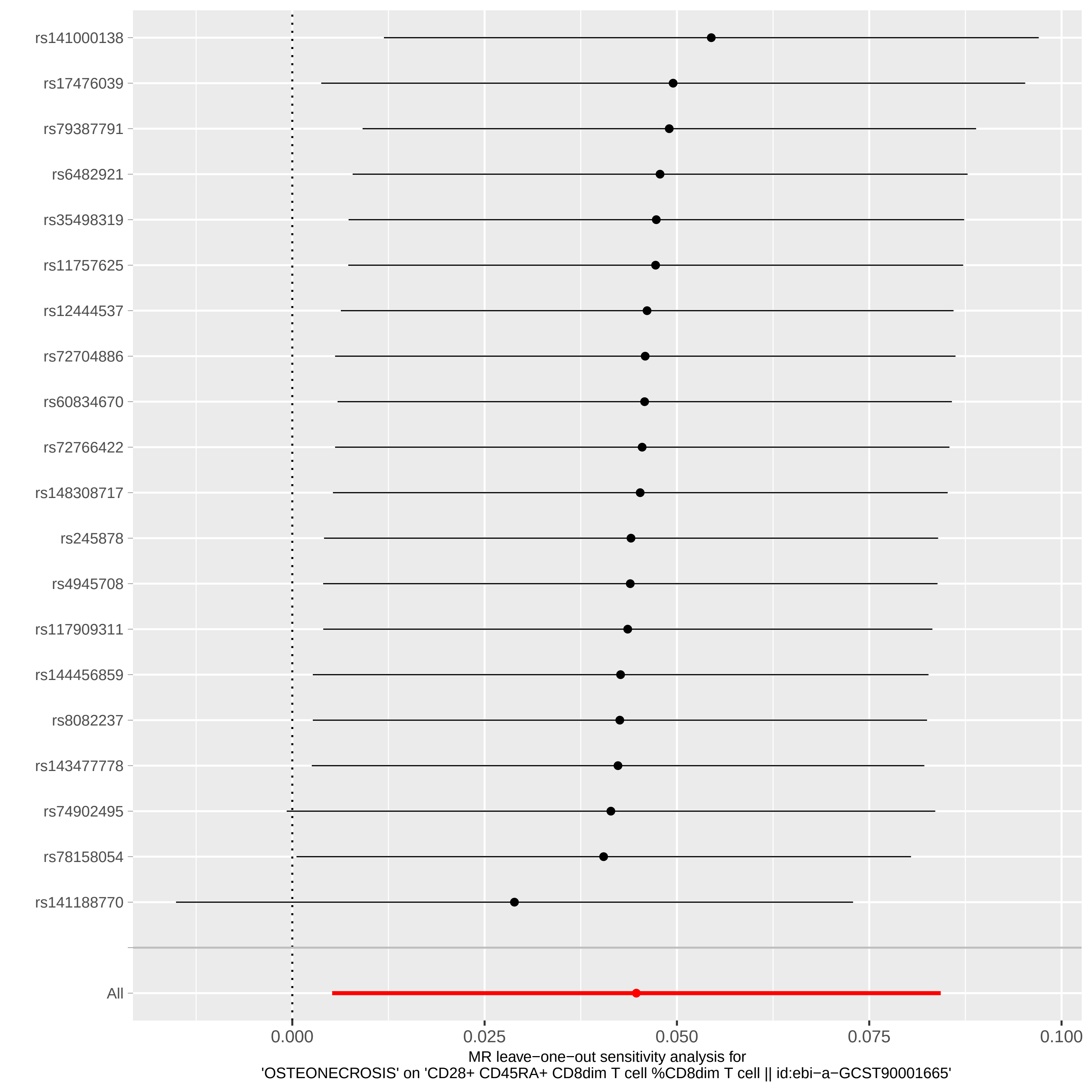

rs141000138
rs17476039
rs79387791
rs6482921
rs35498319
rs11757625
rs12444537
rs72704886
rs60834670
rs72766422
rs148308717
rs245878
rs4945708
rs117909311
rs144456859
rs8082237
rs143477778
rs74902495
rs78158054
rs141188770
All
0.000
0.025
0.050
0.075
0.100
MR leave−one−out sensitivity analysis for
'OSTEONECROSIS' on 'CD28+ CD45RA+ CD8dim T cell %CD8dim T cell || id:ebi−a−GCST90001665'

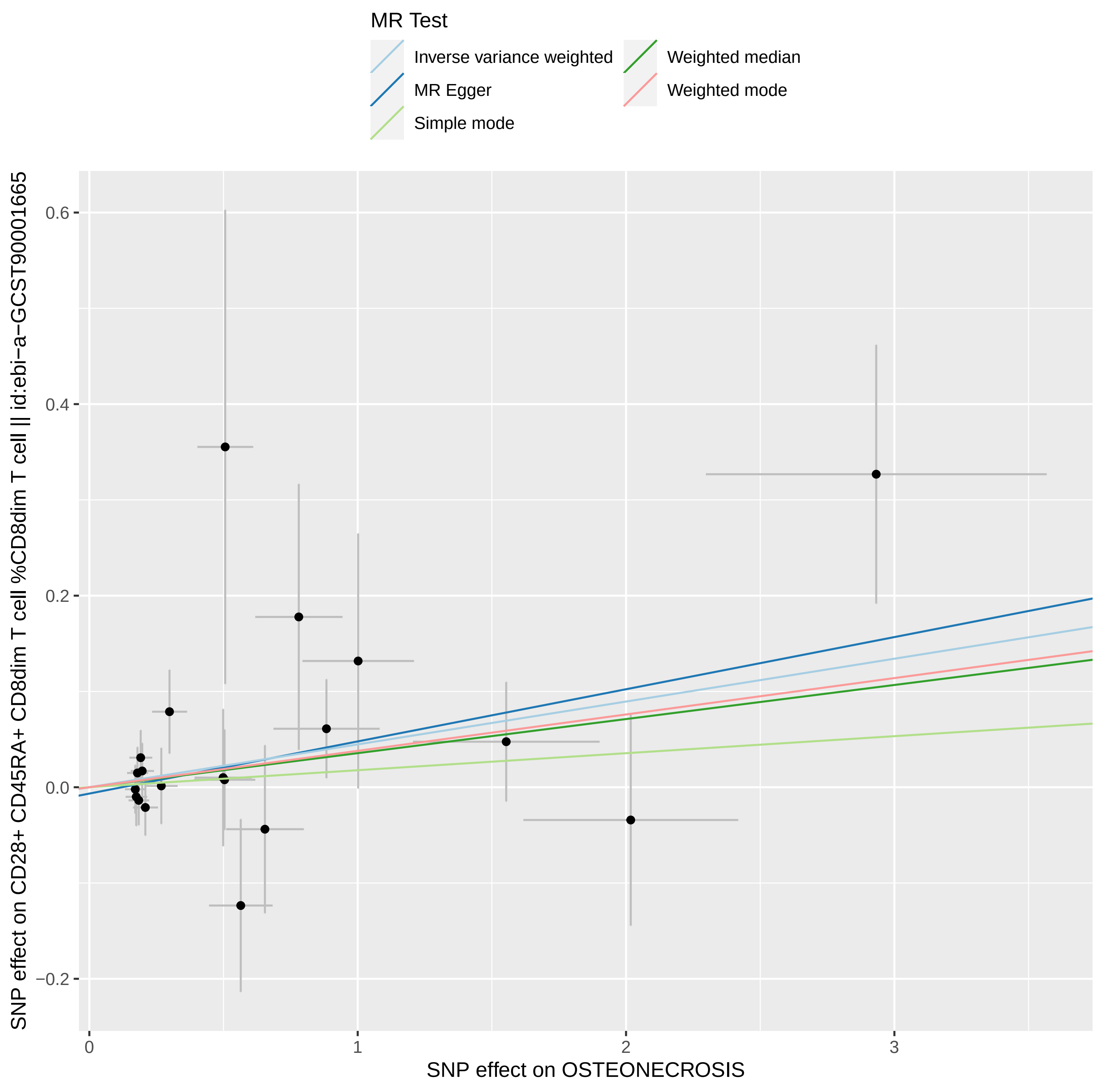
MR Test
Inverse variance weighted
MR Egger
Simple mode
Weighted median
Weighted mode
SNP effect on CD28+ CD45RA+ CD8dim T cell %CD8dim T cell || id:ebi−a−GCST90001665
0.6
0.4
0.2
0.0
−0.2
0
1
2
3
SNP effect on OSTEONECROSIS

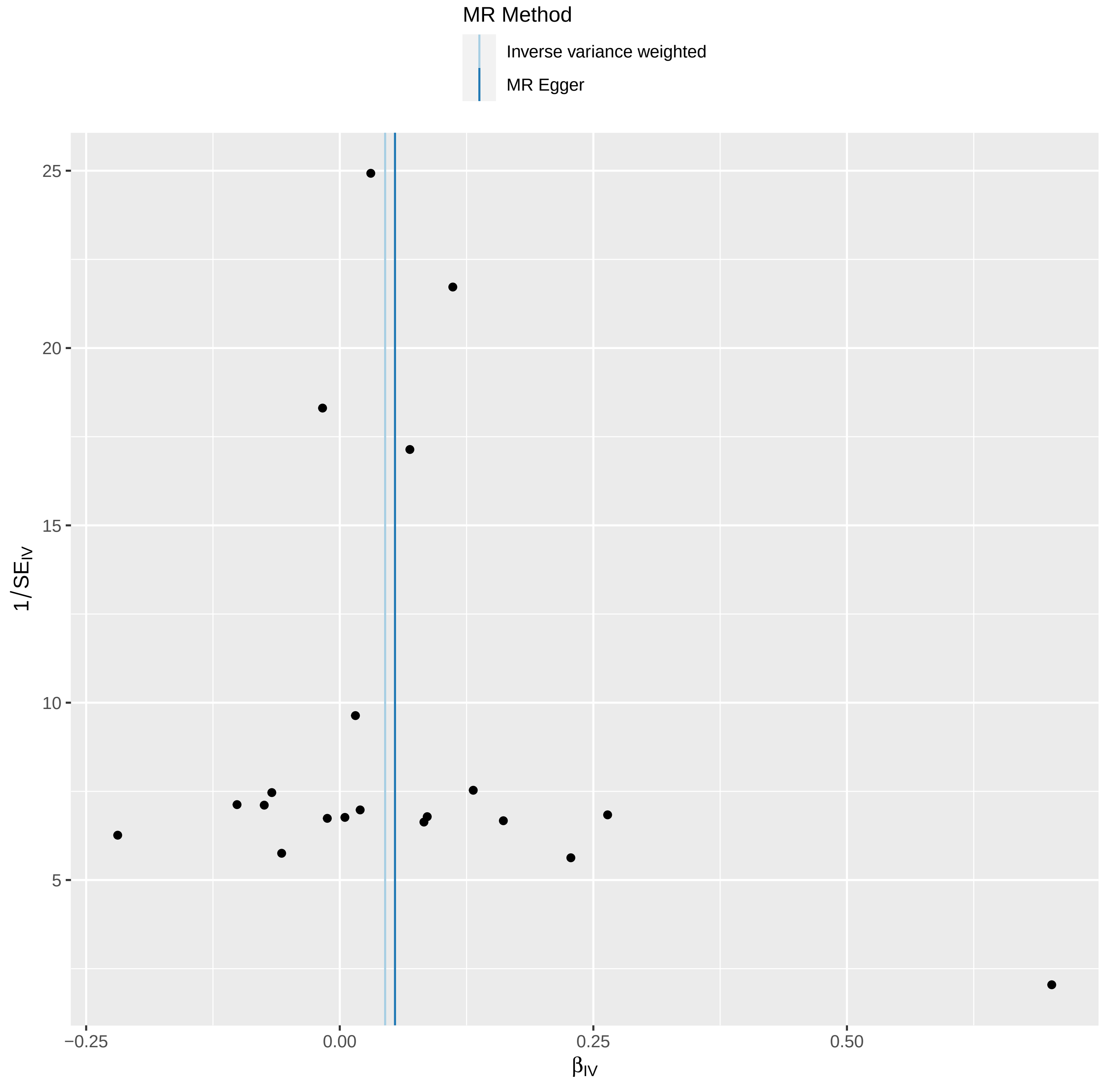

MR Method
Inverse variance weighted
MR Egger
25
20
15
10
5
1/SE_IV
−0.25
0.00
0.25
0.50
β_IV

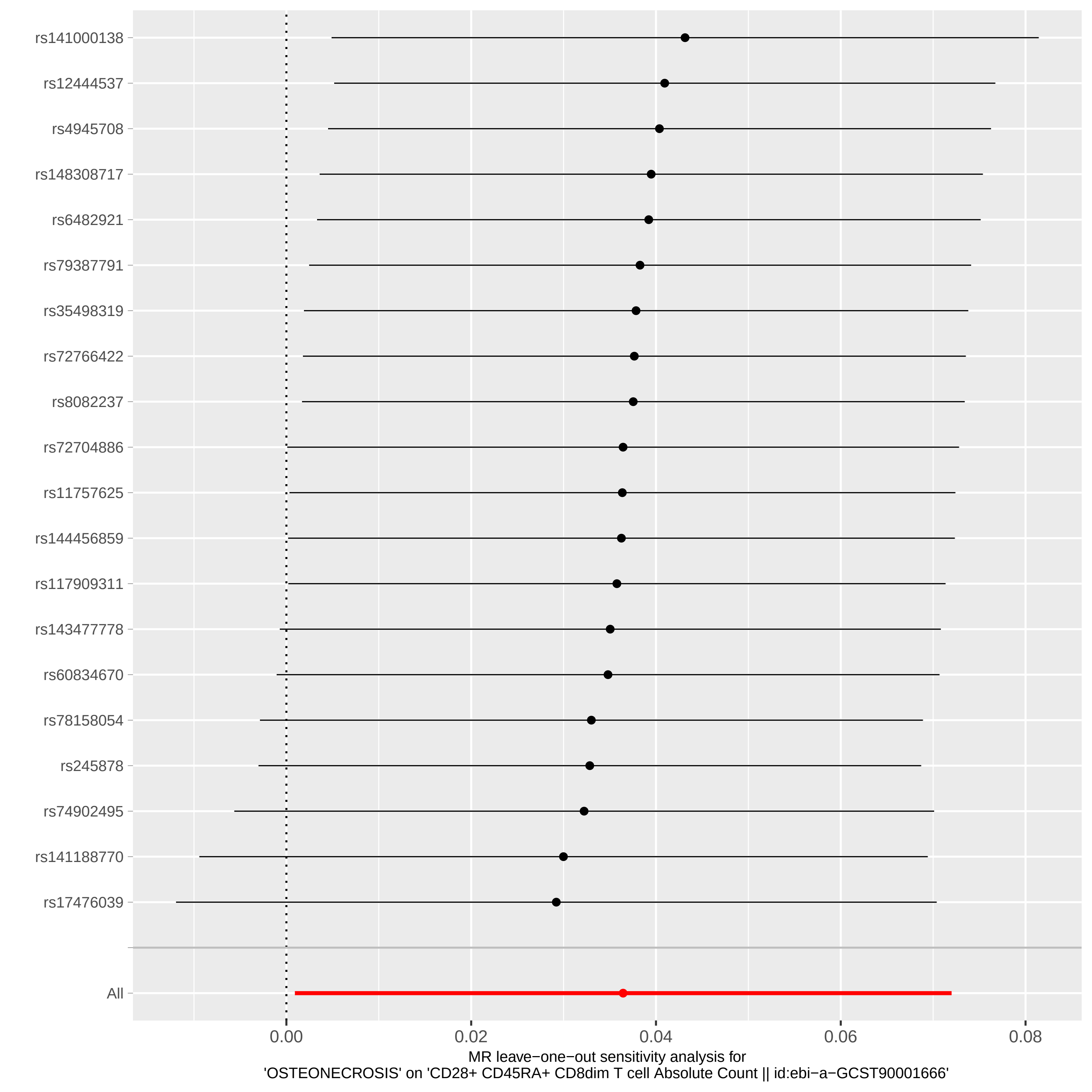

rs141000138
rs12444537
rs4945708
rs148308717
rs6482921
rs79387791
rs35498319
rs72766422
rs8082237
rs72704886
rs11757625
rs144456859
rs117909311
rs143477778
rs60834670
rs78158054
rs245878
rs74902495
rs141188770
rs17476039
All
0.00
0.02
0.04
0.06
0.08
MR leave−one−out sensitivity analysis for
'OSTEONECROSIS' on 'CD28+ CD45RA+ CD8dim T cell Absolute Count || id:ebi−a−GCST90001666'

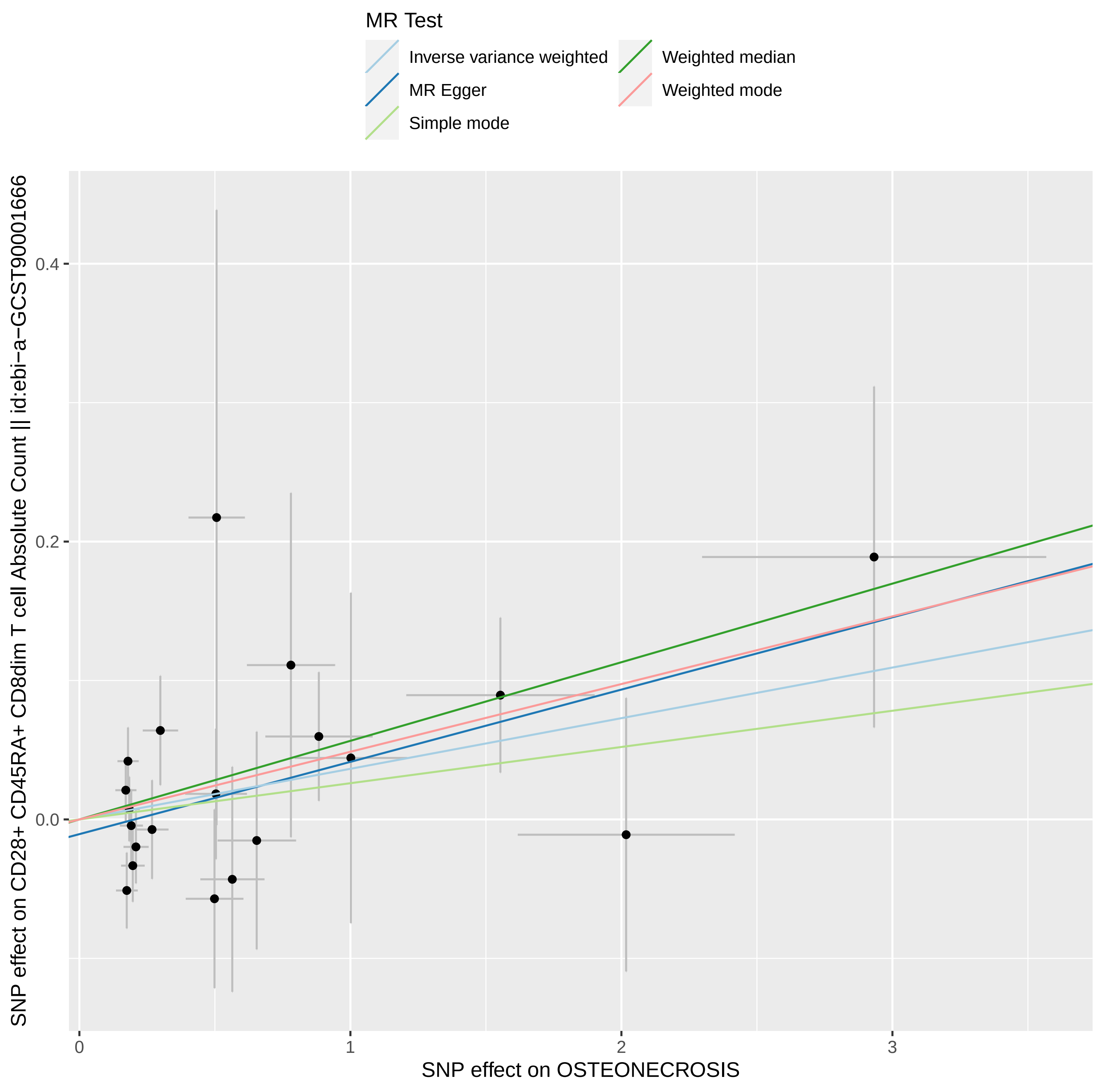

MR Test
Inverse variance weighted
MR Egger
Simple mode
Weighted median
Weighted mode
0.4
0.2
0.0
0
1
2
3
SNP effect on CD28+ CD45RA+ CD8dim T cell Absolute Count || id:ebi−a−GCST90001666
SNP effect on OSTEONECROSIS

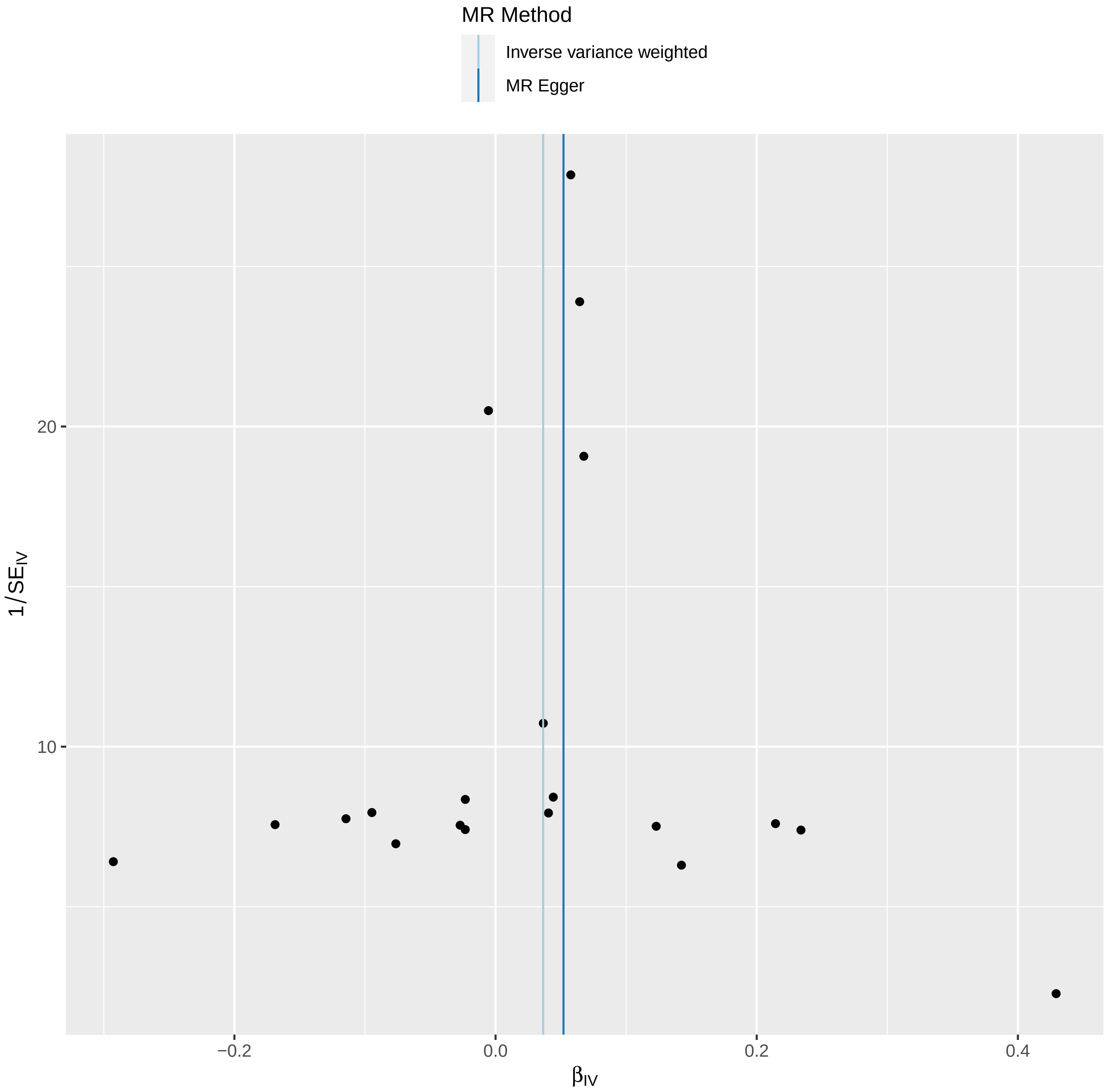
MR Method
Inverse variance weighted
MR Egger
20
10
1/SE$_{IV}$
−0.2
0.0
0.2
0.4
β$_{IV}$

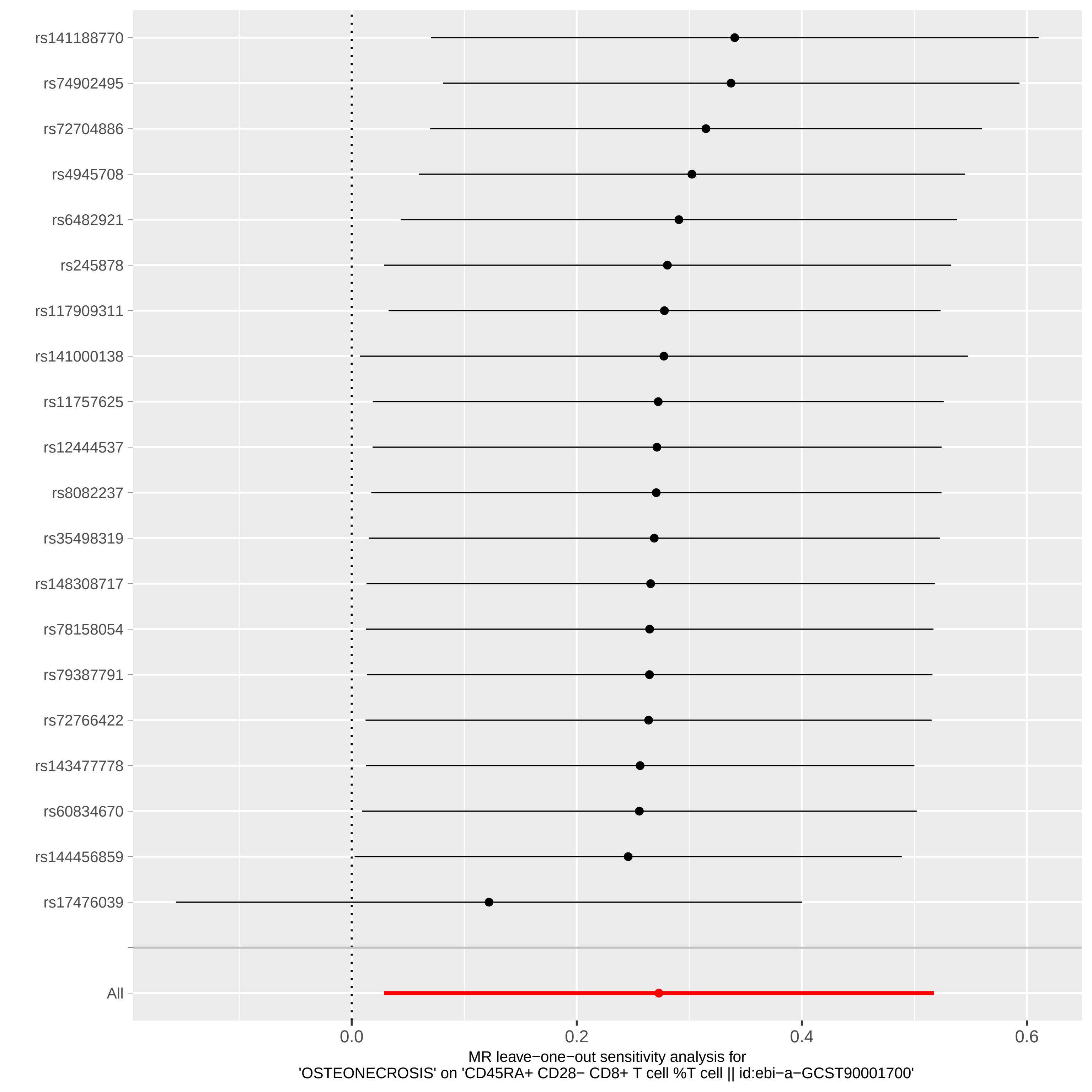
rs141188770
rs74902495
rs72704886
rs4945708
rs6482921
rs245878
rs117909311
rs141000138
rs11757625
rs12444537
rs8082237
rs35498319
rs148308717
rs78158054
rs79387791
rs72766422
rs143477778
rs60834670
rs144456859
rs17476039
All
0.0
0.2
0.4
0.6
MR leave−one−out sensitivity analysis for
'OSTEONECROSIS' on 'CD45RA+ CD28− CD8+ T cell %T cell || id:ebi−a−GCST90001700'

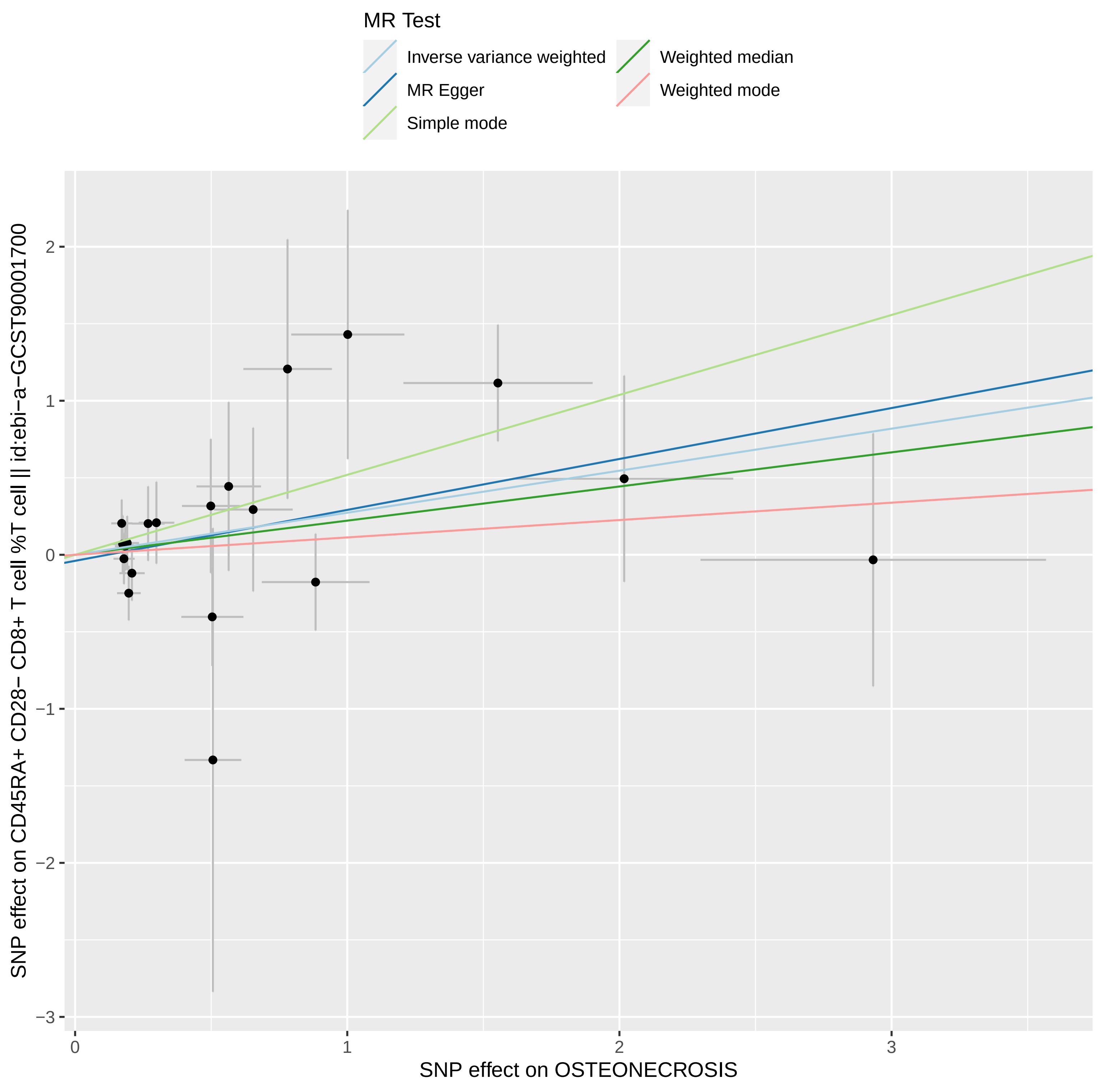
MR Test
Inverse variance weighted
MR Egger
Simple mode
Weighted median
Weighted mode
SNP effect on CD45RA+ CD28− CD8+ T cell %T cell || id:ebi−a−GCST90001700
SNP effect on OSTEONECROSIS
2
1
0
−1
−2
−3
0
1
2
3

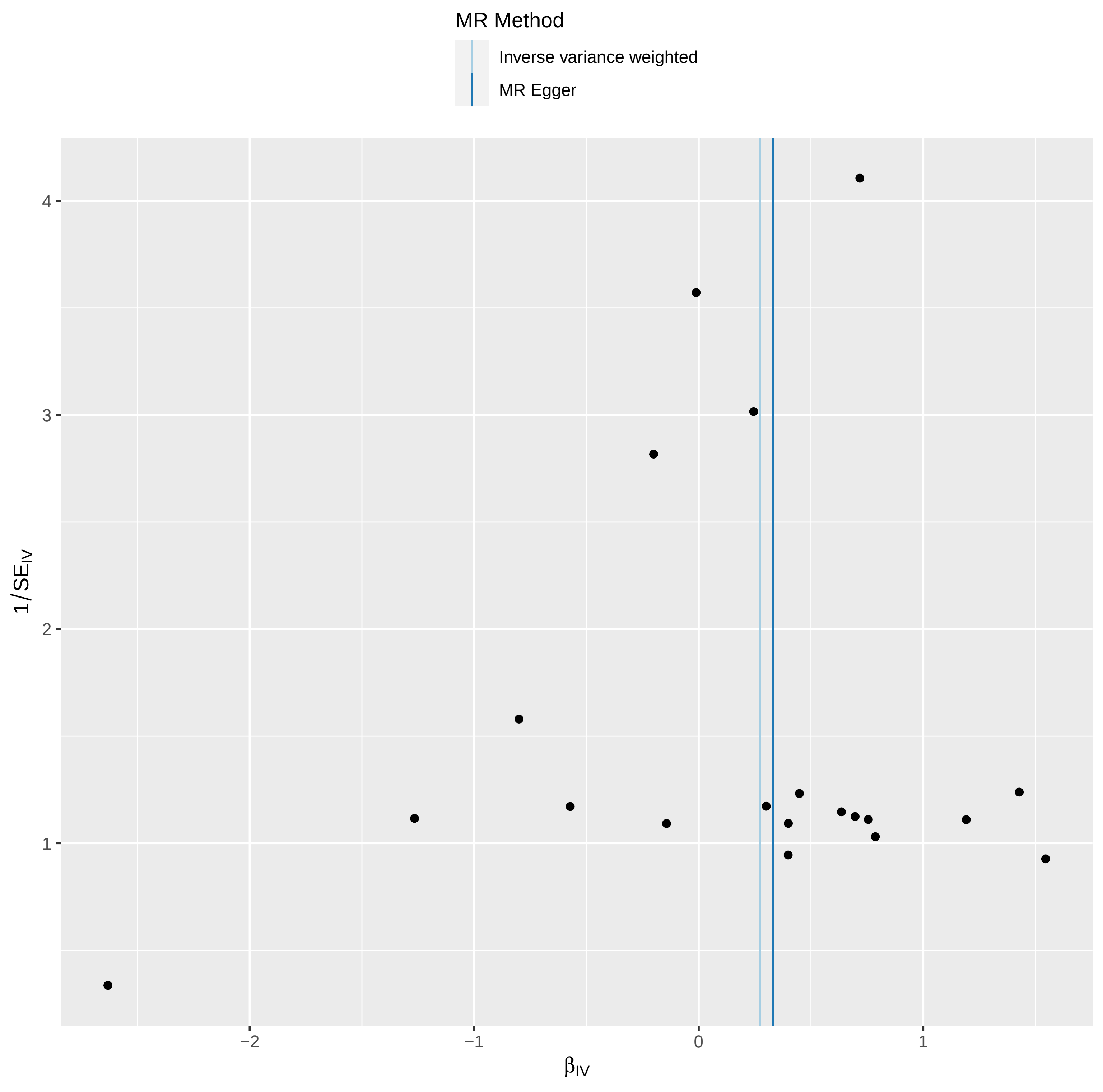

MR Method
Inverse variance weighted
MR Egger
4
3
2
1
−2
−1
0
1
$1/SE_{IV}$
β_{IV}

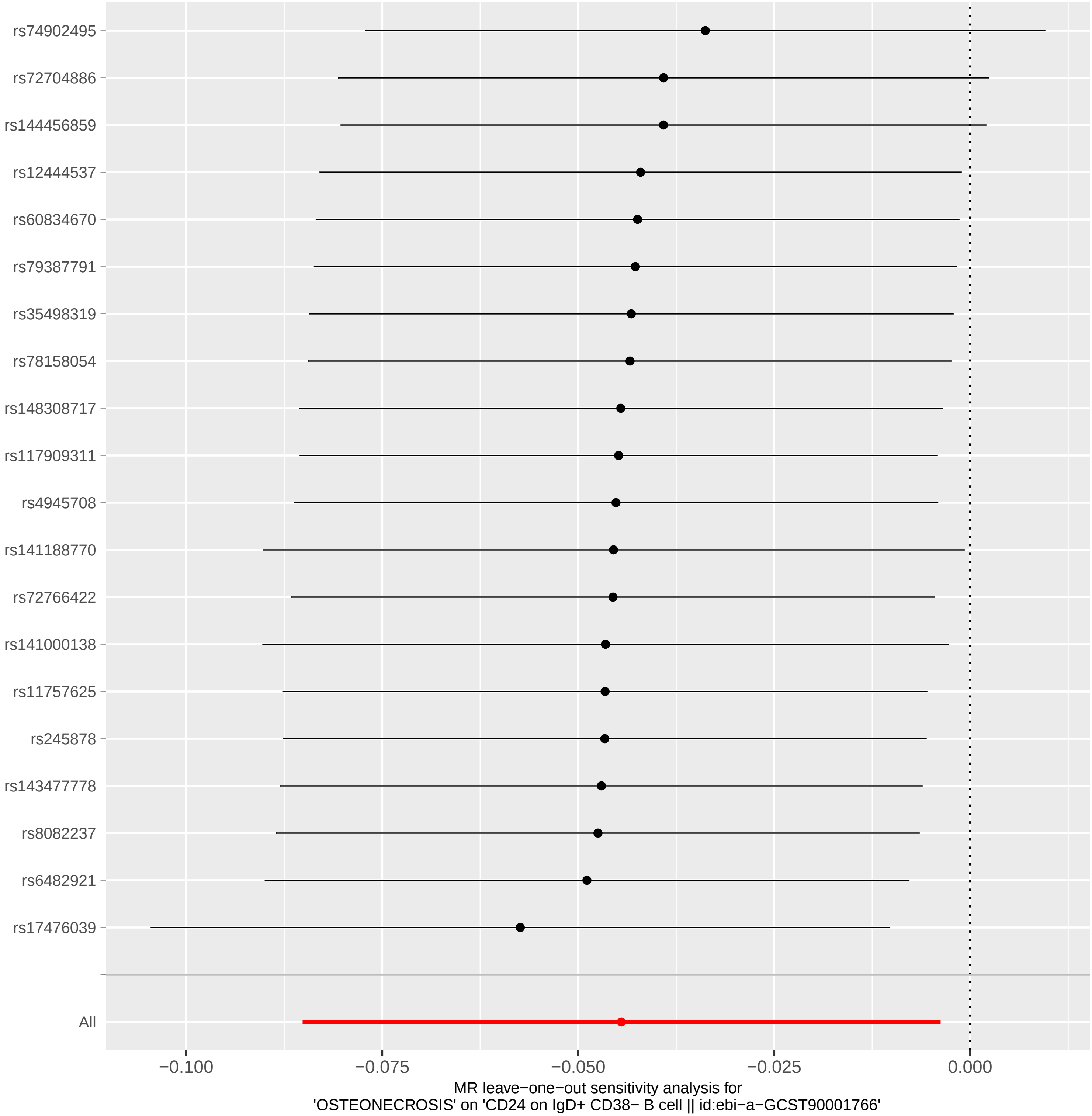

rs74902495
rs72704886
rs144456859
rs12444537
rs60834670
rs79387791
rs35498319
rs78158054
rs148308717
rs117909311
rs4945708
rs141188770
rs72766422
rs141000138
rs11757625
rs245878
rs143477778
rs8082237
rs6482921
rs17476039
All
−0.100
−0.075
−0.050
−0.025
0.000
MR leave−one−out sensitivity analysis for
'OSTEONECROSIS' on 'CD24 on IgD+ CD38− B cell || id:ebi−a−GCST90001766'

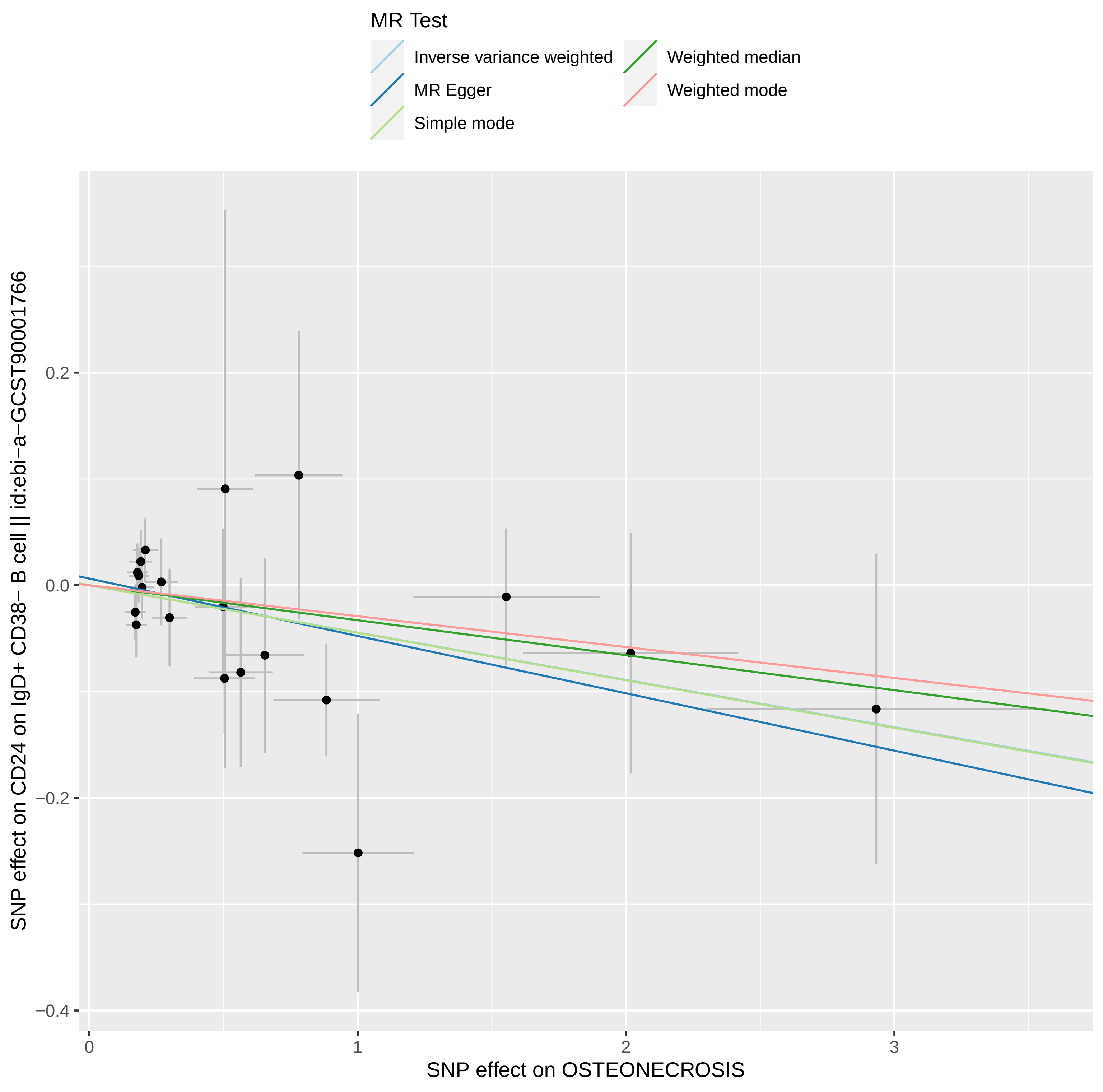
MR Test
Inverse variance weighted
MR Egger
Simple mode
Weighted median
Weighted mode
0.2
0.0
−0.2
−0.4
0
1
2
3
SNP effect on CD24 on IgD+ CD38− B cell || id:ebi−a−GCST90001766
SNP effect on OSTEONECROSIS

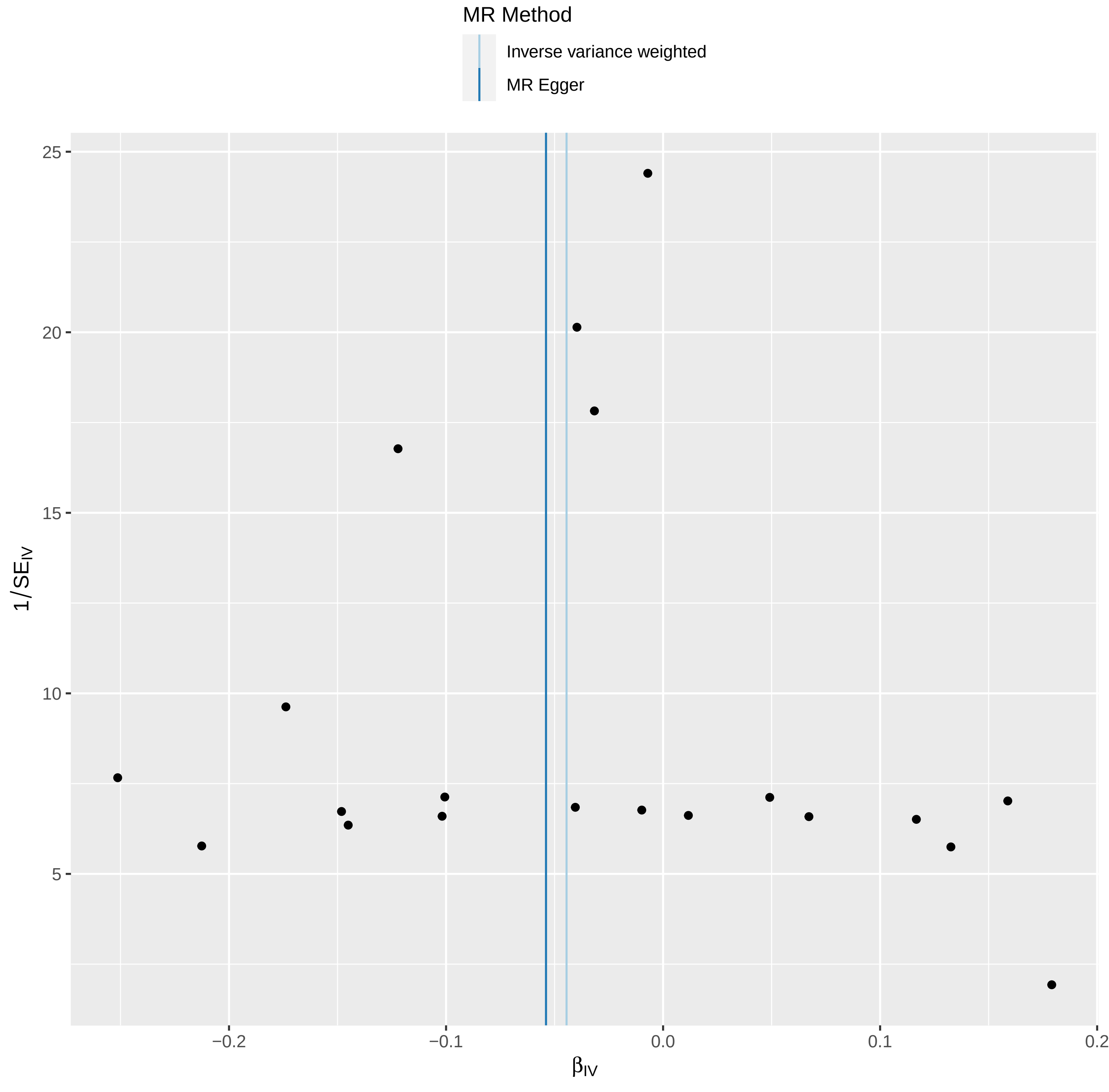

MR Method
Inverse variance weighted
MR Egger
25
20
15
10
5
1/SE_IV
−0.2
−0.1
0.0
0.1
0.2
β_IV

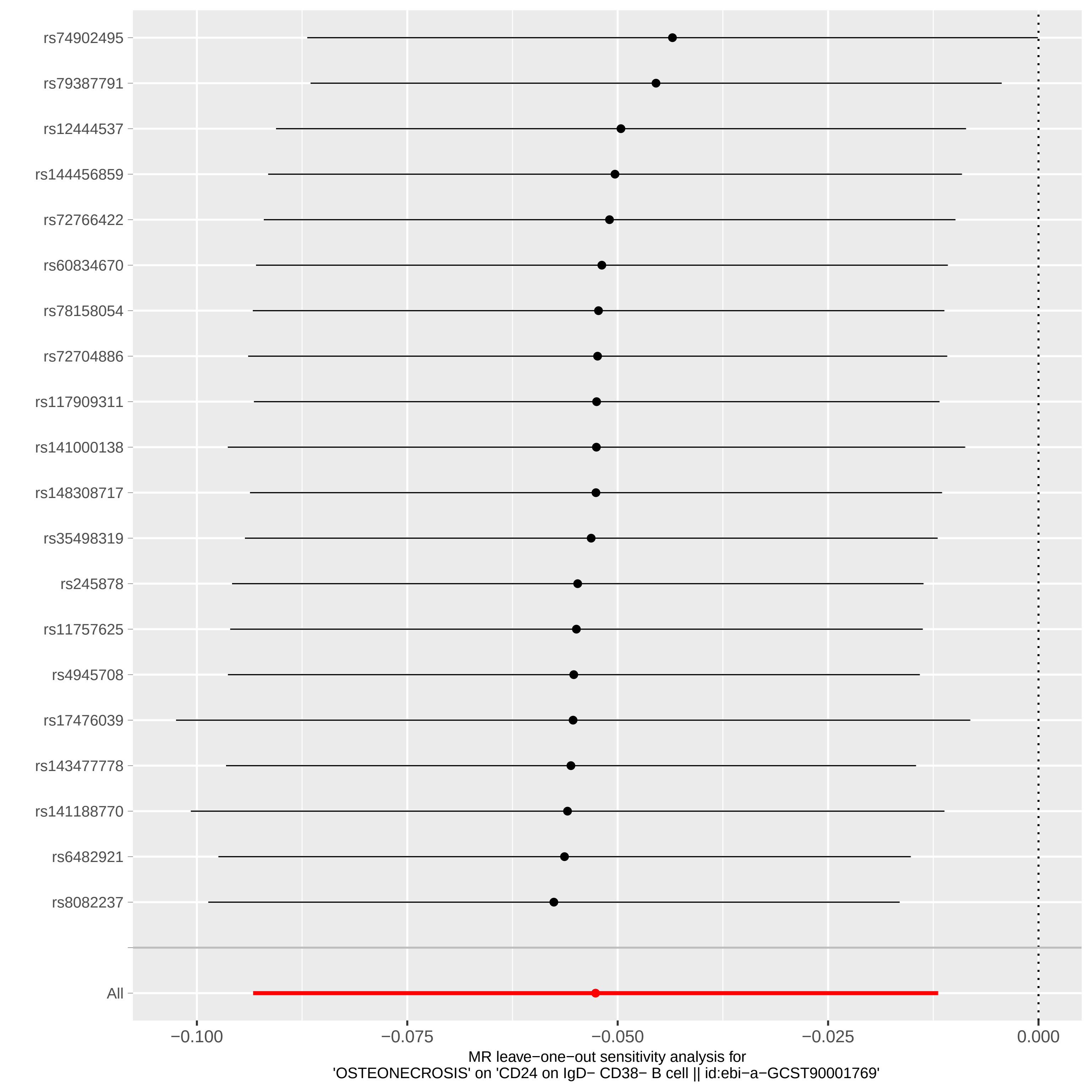
rs74902495
rs79387791
rs12444537
rs144456859
rs72766422
rs60834670
rs78158054
rs72704886
rs117909311
rs141000138
rs148308717
rs35498319
rs245878
rs11757625
rs4945708
rs17476039
rs143477778
rs141188770
rs6482921
rs8082237
All
−0.100
−0.075
−0.050
−0.025
0.000
MR leave−one−out sensitivity analysis for
'OSTEONECROSIS' on 'CD24 on IgD− CD38− B cell || id:ebi−a−GCST90001769'

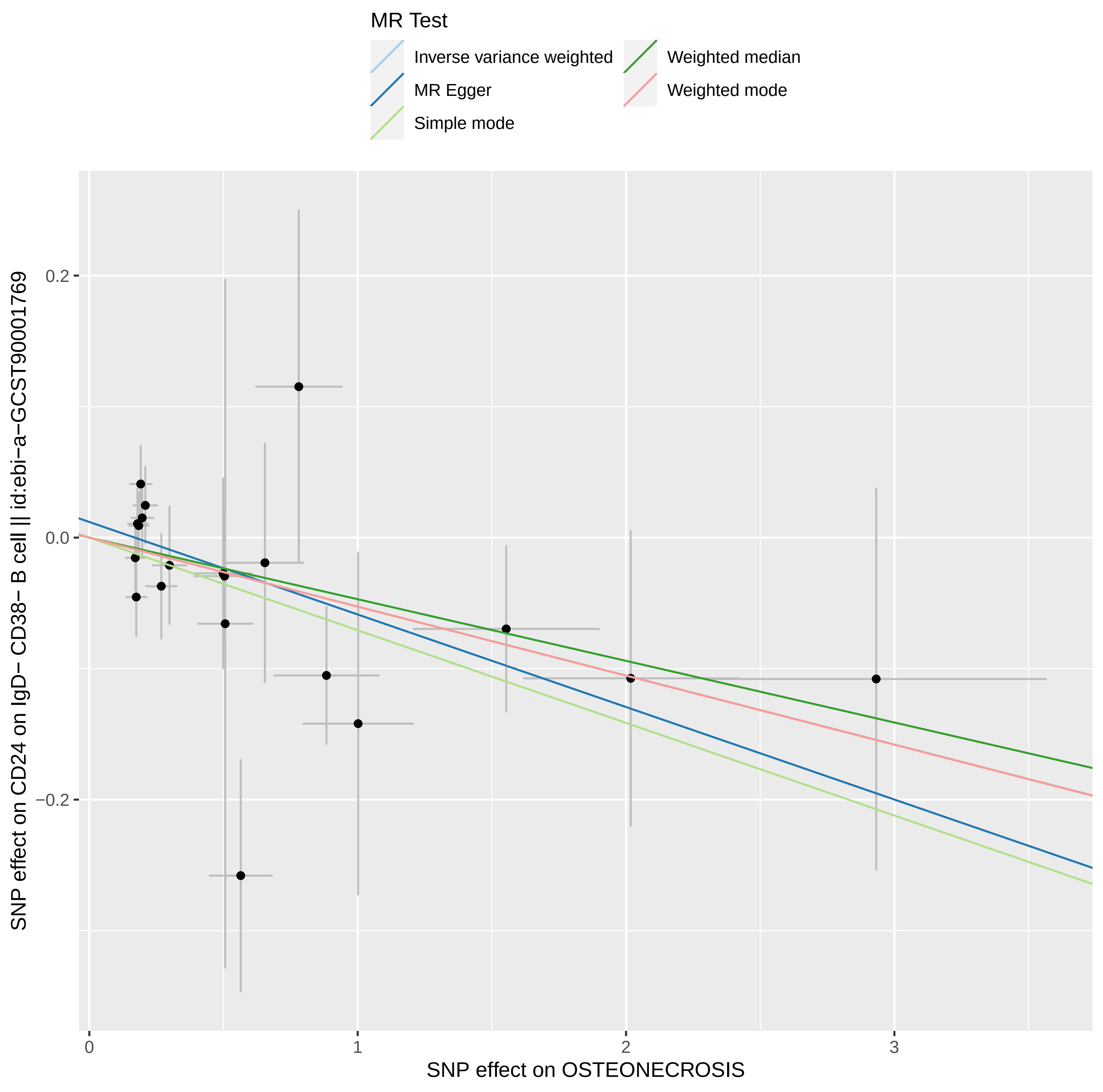
MR Test
Inverse variance weighted
MR Egger
Simple mode
Weighted median
Weighted mode
0.2
0.0
−0.2
0
1
2
3
SNP effect on CD24 on IgD− CD38− B cell || id:ebi−a−GCST90001769
SNP effect on OSTEONECROSIS

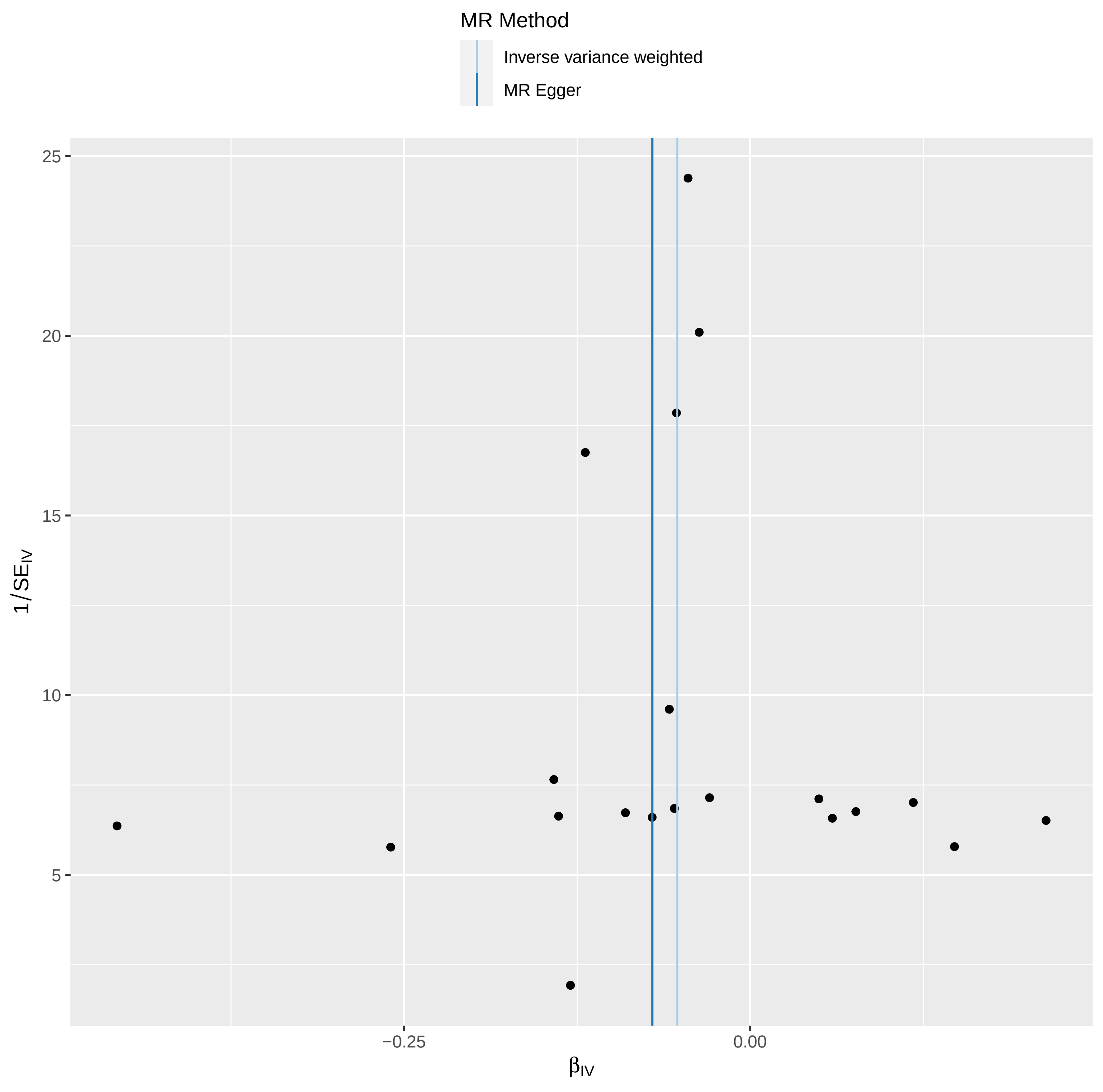
MR Method
Inverse variance weighted
MR Egger
25
20
15
10
5
1/SE$_{IV}$
−0.25
0.00
β$_{IV}$

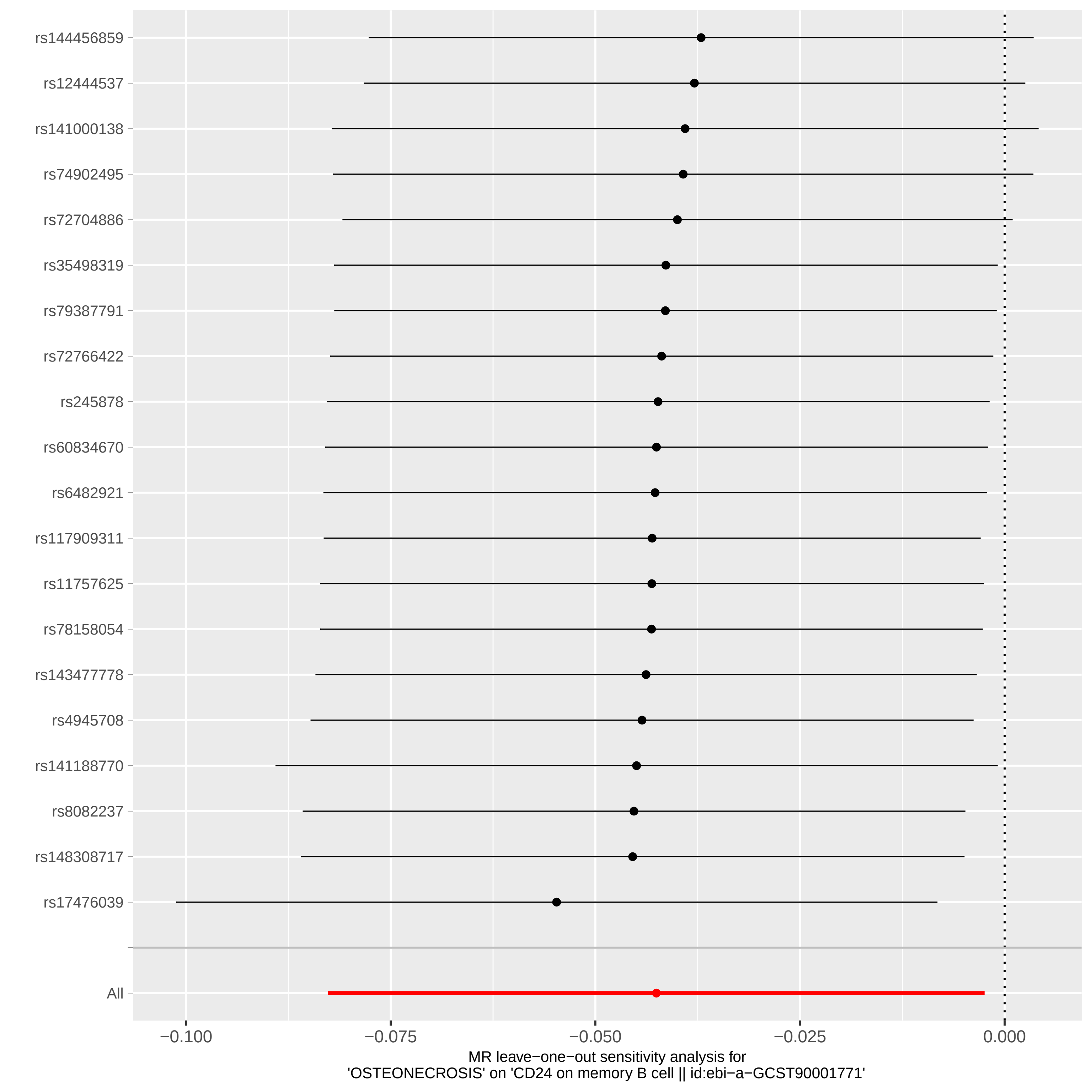
rs144456859
rs12444537
rs141000138
rs74902495
rs72704886
rs35498319
rs79387791
rs72766422
rs245878
rs60834670
rs6482921
rs117909311
rs11757625
rs78158054
rs143477778
rs4945708
rs141188770
rs8082237
rs148308717
rs17476039
All
−0.100
−0.075
−0.050
−0.025
0.000
MR leave−one−out sensitivity analysis for
'OSTEONECROSIS' on 'CD24 on memory B cell || id:ebi−a−GCST90001771'

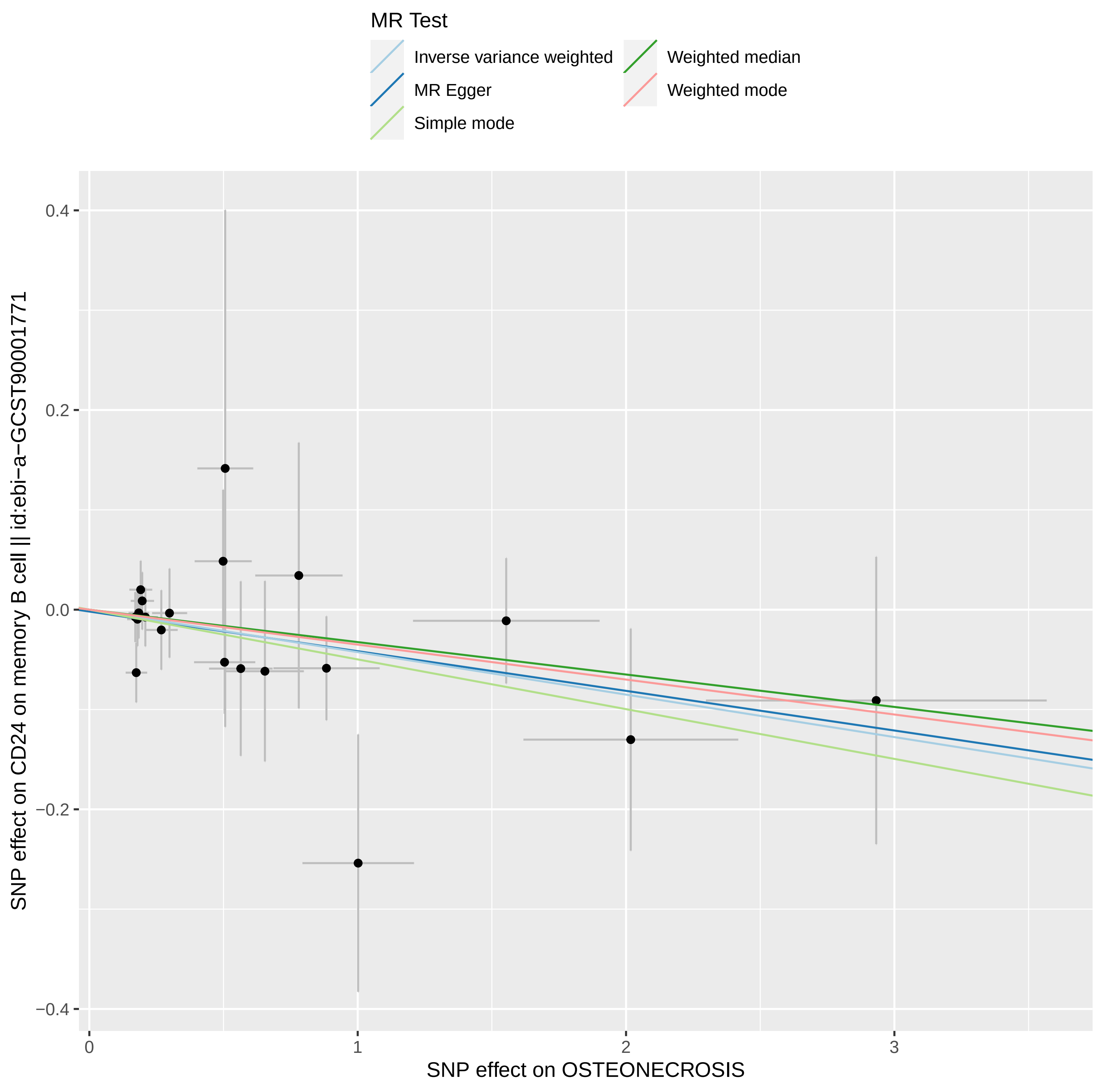
MR Test
Inverse variance weighted
MR Egger
Simple mode
Weighted median
Weighted mode
0.4
0.2
0.0
−0.2
−0.4
0
1
2
3
SNP effect on CD24 on memory B cell || id:ebi−a−GCST90001771
SNP effect on OSTEONECROSIS

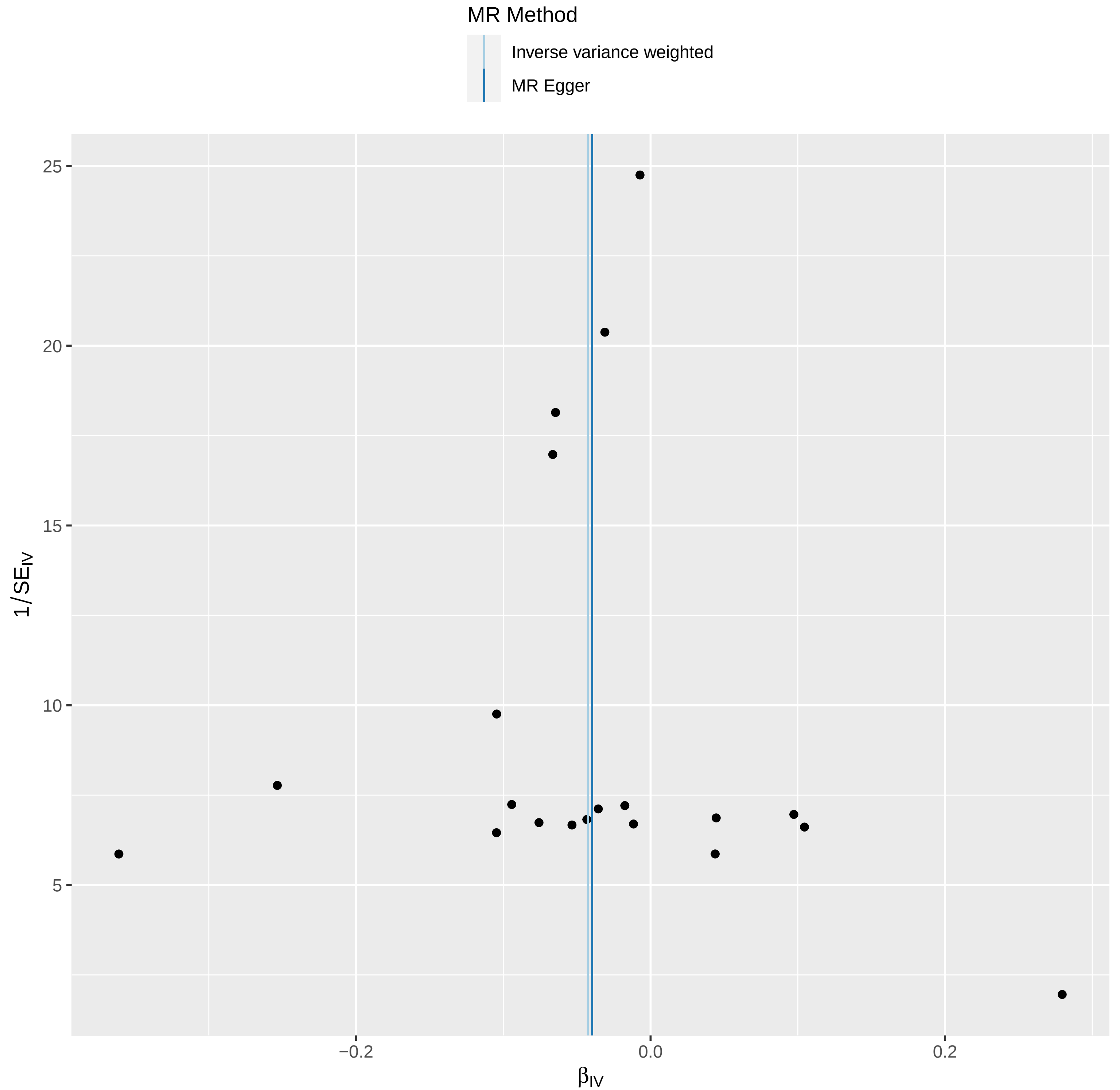
MR Method
Inverse variance weighted
MR Egger
25
20
15
10
5
1/SE_IV
−0.2
0.0
0.2
β_IV

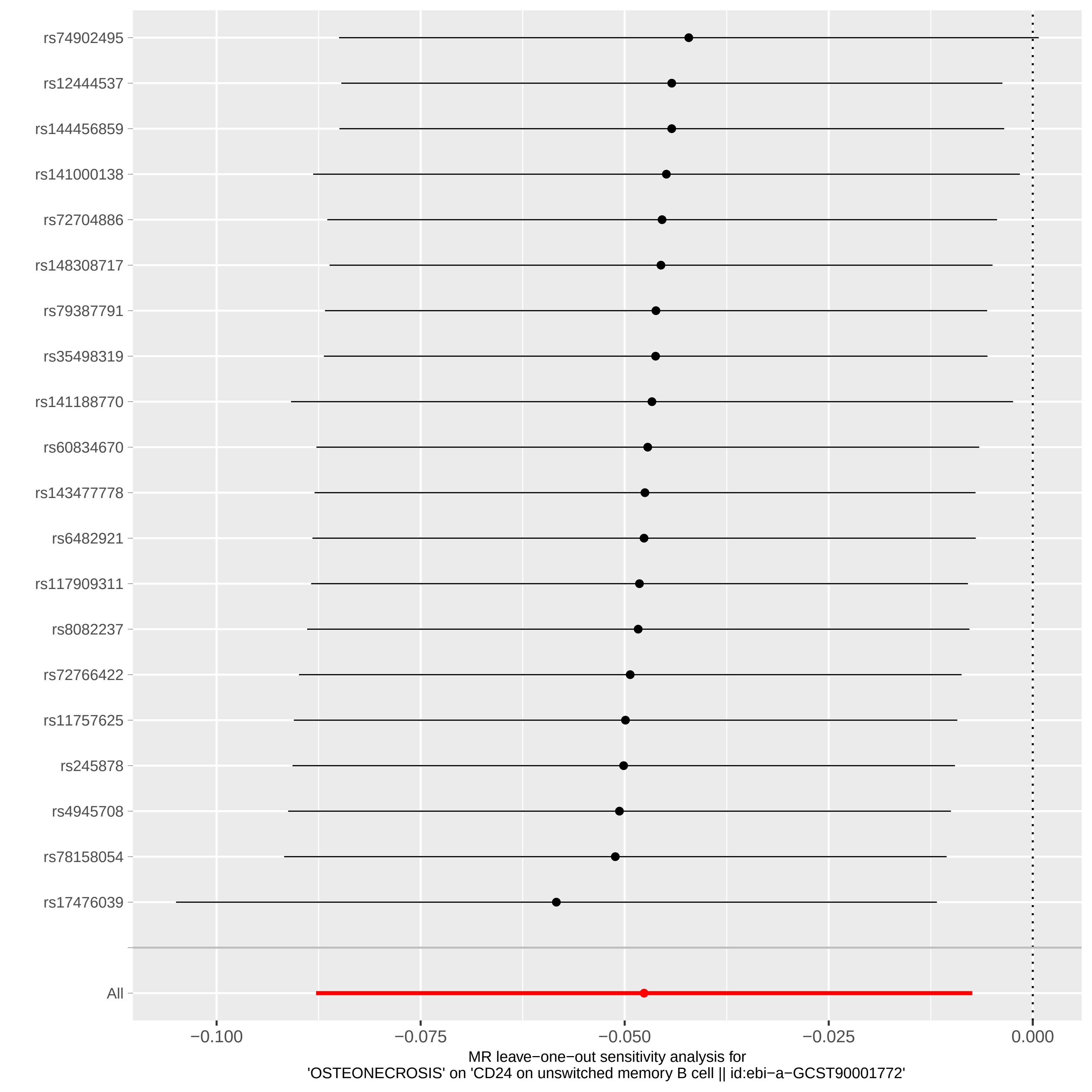
rs74902495
rs12444537
rs144456859
rs141000138
rs72704886
rs148308717
rs79387791
rs35498319
rs141188770
rs60834670
rs143477778
rs6482921
rs117909311
rs8082237
rs72766422
rs11757625
rs245878
rs4945708
rs78158054
rs17476039
All
−0.100
−0.075
−0.050
−0.025
0.000
MR leave−one−out sensitivity analysis for
'OSTEONECROSIS' on 'CD24 on unswitched memory B cell || id:ebi−a−GCST90001772'

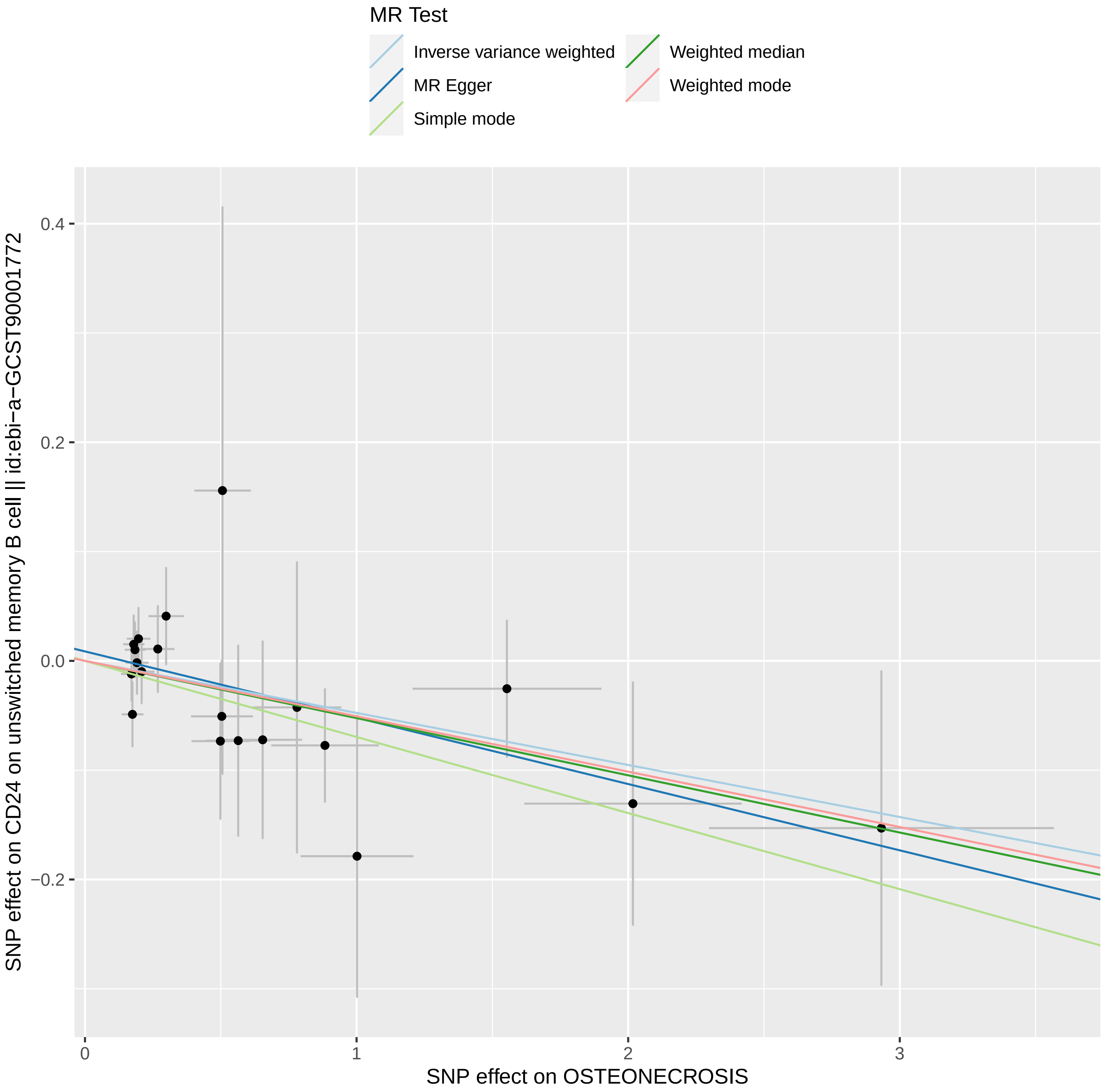
MR Test
Inverse variance weighted
MR Egger
Simple mode
Weighted median
Weighted mode
0.4
0.2
0.0
−0.2
0
1
2
3
SNP effect on CD24 on unswitched memory B cell || id:ebi−a−GCST90001772
SNP effect on OSTEONECROSIS

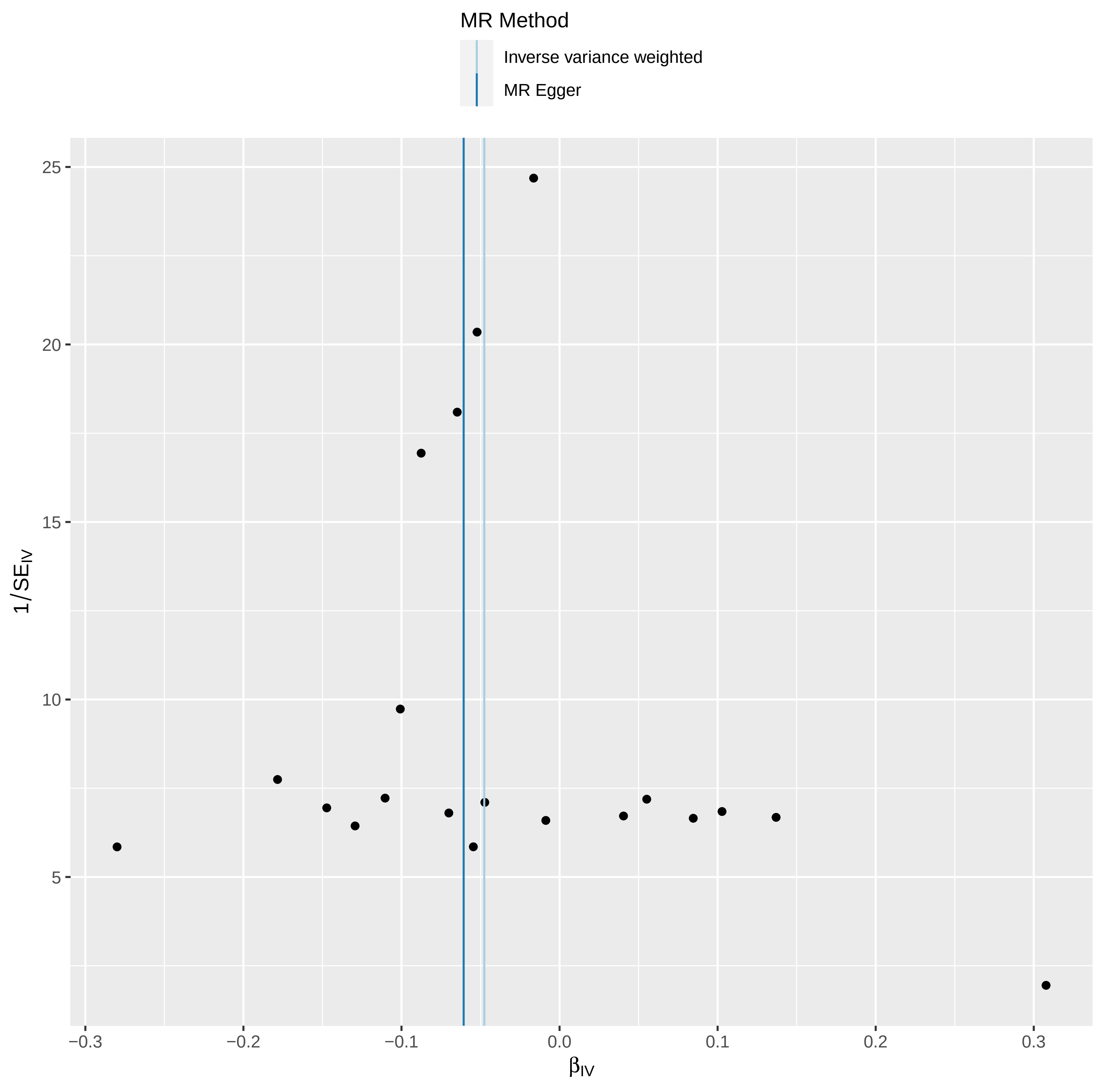
MR Method
Inverse variance weighted
MR Egger
25
20
15
10
5
1/SE_IV
−0.3
−0.2
−0.1
0.0
0.1
0.2
0.3
β_IV

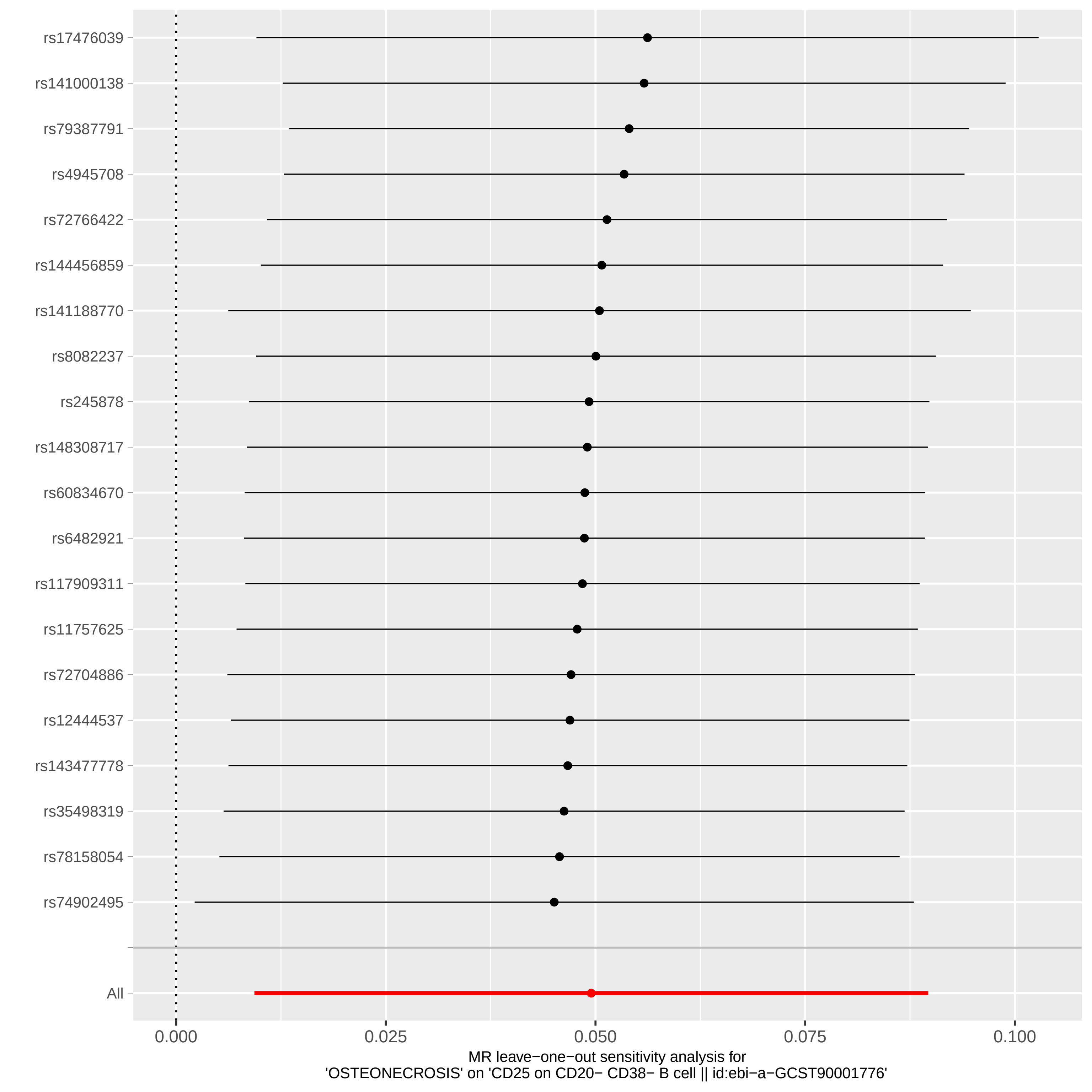
rs17476039
rs141000138
rs79387791
rs4945708
rs72766422
rs144456859
rs141188770
rs8082237
rs245878
rs148308717
rs60834670
rs6482921
rs117909311
rs11757625
rs72704886
rs12444537
rs143477778
rs35498319
rs78158054
rs74902495
All
0.000
0.025
0.050
0.075
0.100
MR leave−one−out sensitivity analysis for
'OSTEONECROSIS' on 'CD25 on CD20− CD38− B cell || id:ebi−a−GCST90001776'

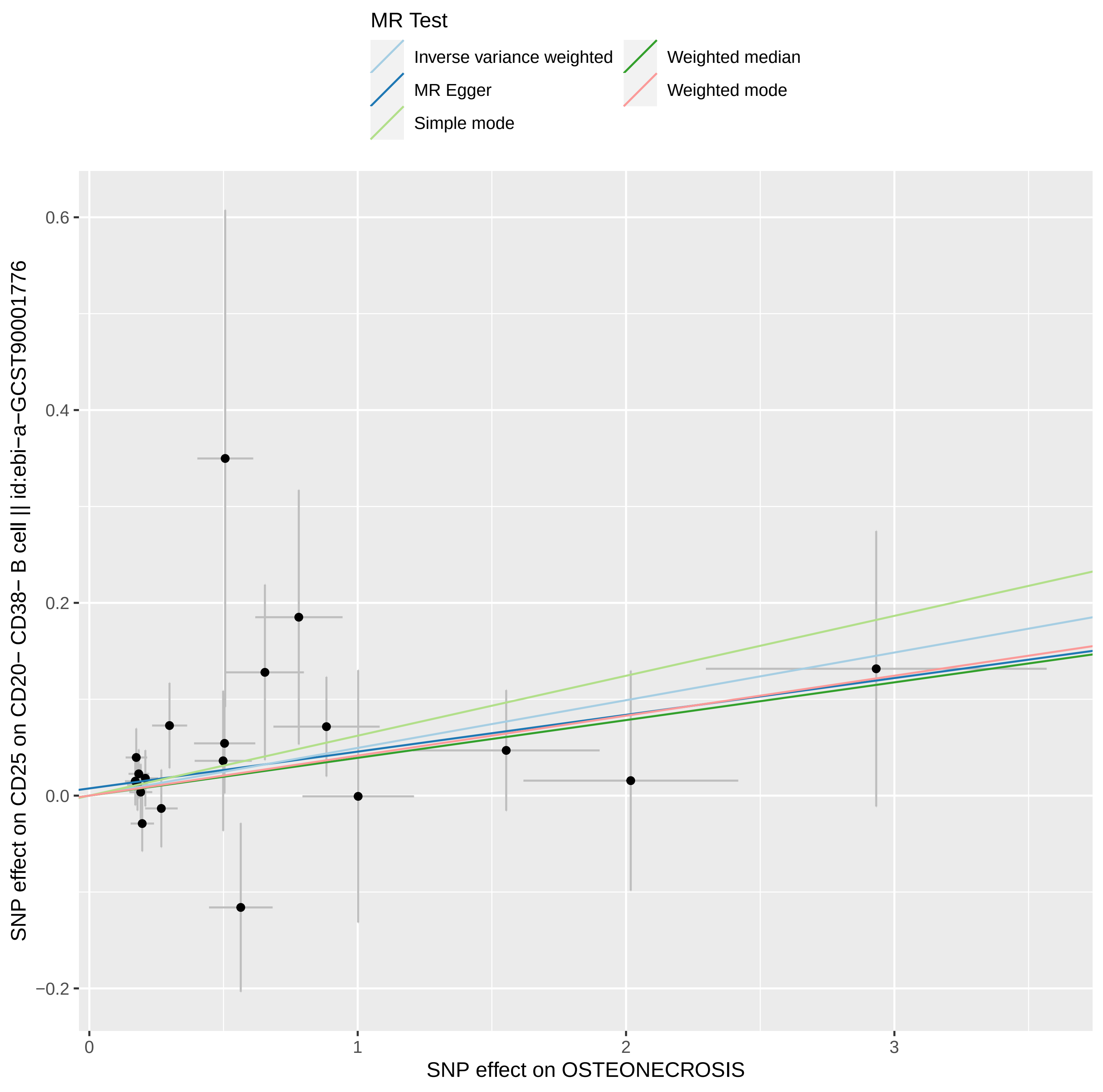

MR Test
Inverse variance weighted
MR Egger
Simple mode
Weighted median
Weighted mode
SNP effect on CD25 on CD20− CD38− B cell || id:ebi−a−GCST90001776
0.6
0.4
0.2
0.0
−0.2
0
1
2
3
SNP effect on OSTEONECROSIS

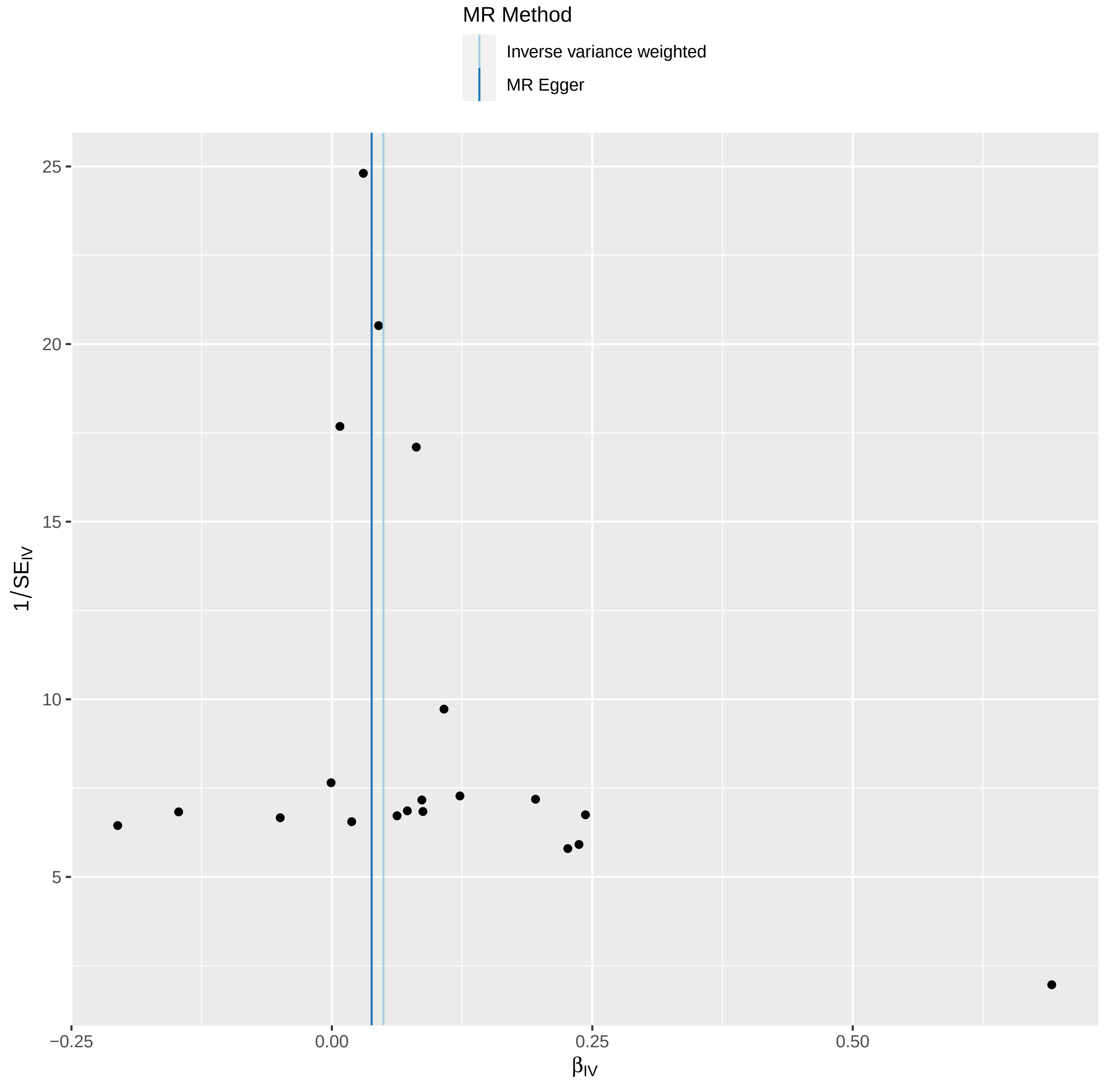
MR Method
Inverse variance weighted
MR Egger
25
20
15
10
5
1/SE$_{IV}$
−0.25
0.00
0.25
0.50
β$_{IV}$

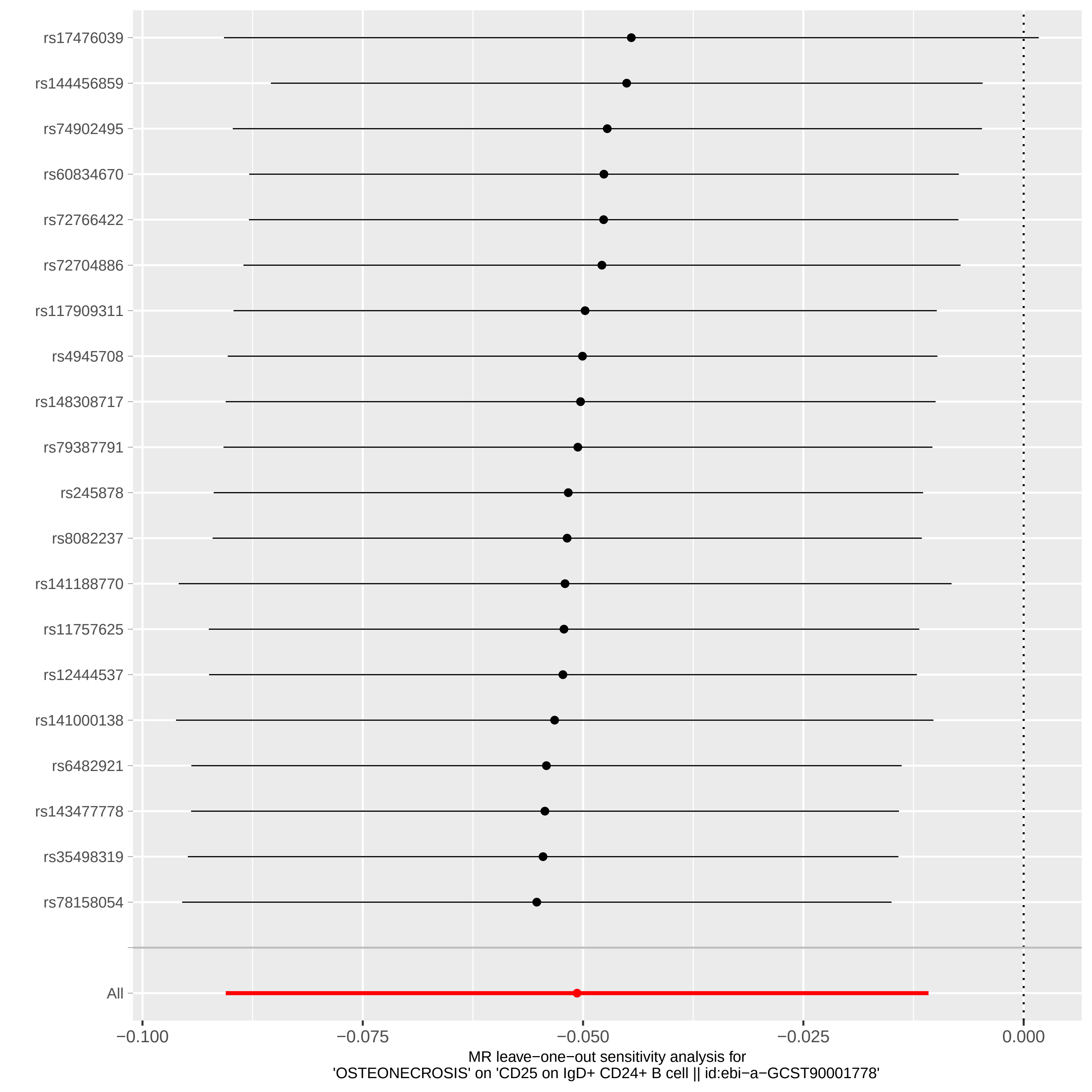
rs17476039
rs144456859
rs74902495
rs60834670
rs72766422
rs72704886
rs117909311
rs4945708
rs148308717
rs79387791
rs245878
rs8082237
rs141188770
rs11757625
rs12444537
rs141000138
rs6482921
rs143477778
rs35498319
rs78158054
All
−0.100
−0.075
−0.050
−0.025
0.000
MR leave−one−out sensitivity analysis for
'OSTEONECROSIS' on 'CD25 on IgD+ CD24+ B cell || id:ebi−a−GCST90001778'

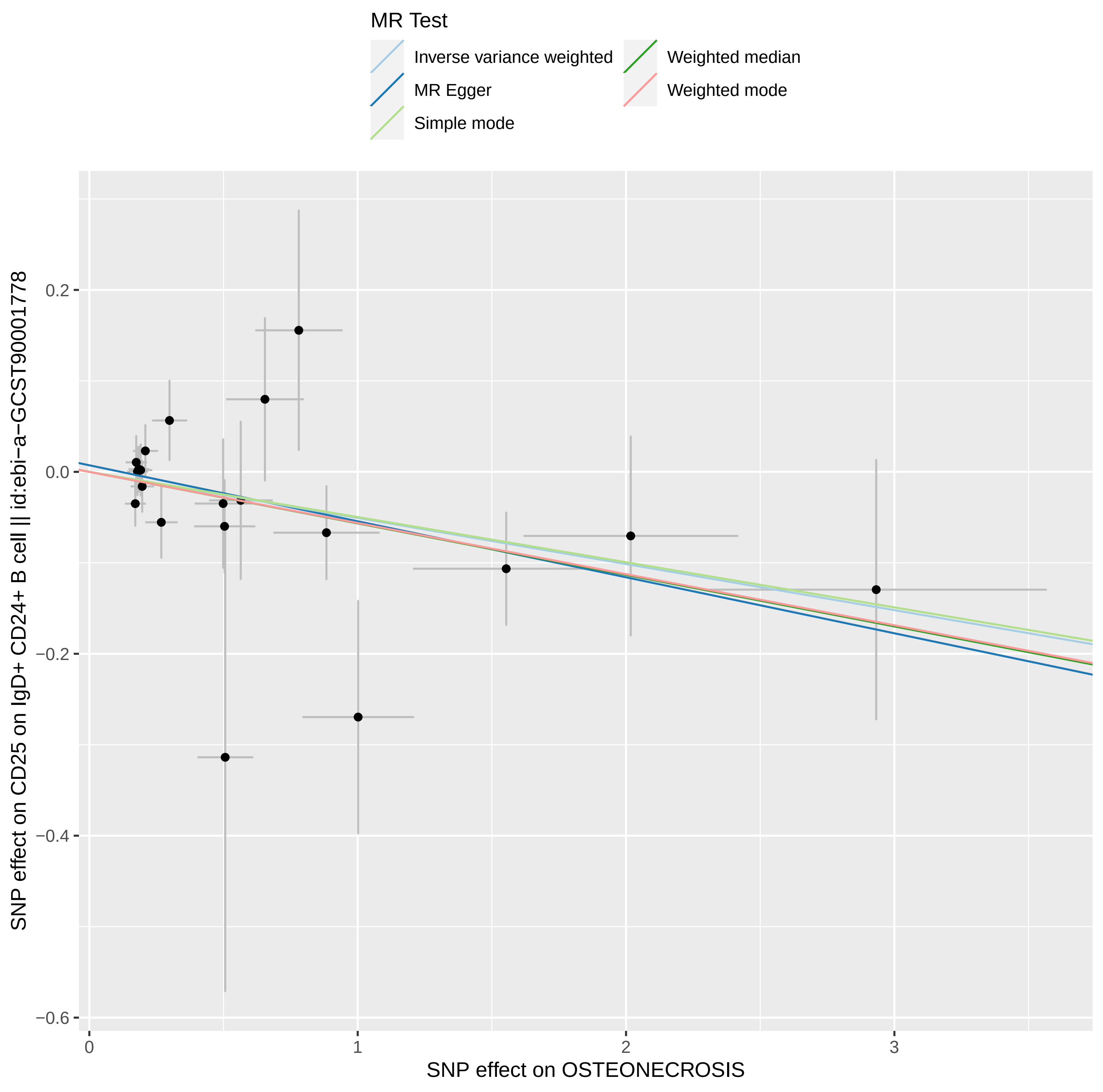

MR Test
Inverse variance weighted
MR Egger
Simple mode
Weighted median
Weighted mode
SNP effect on CD25 on IgD+ CD24+ B cell || id:ebi−a−GCST90001778
0.2
0.0
−0.2
−0.4
−0.6
0
1
2
3
SNP effect on OSTEONECROSIS

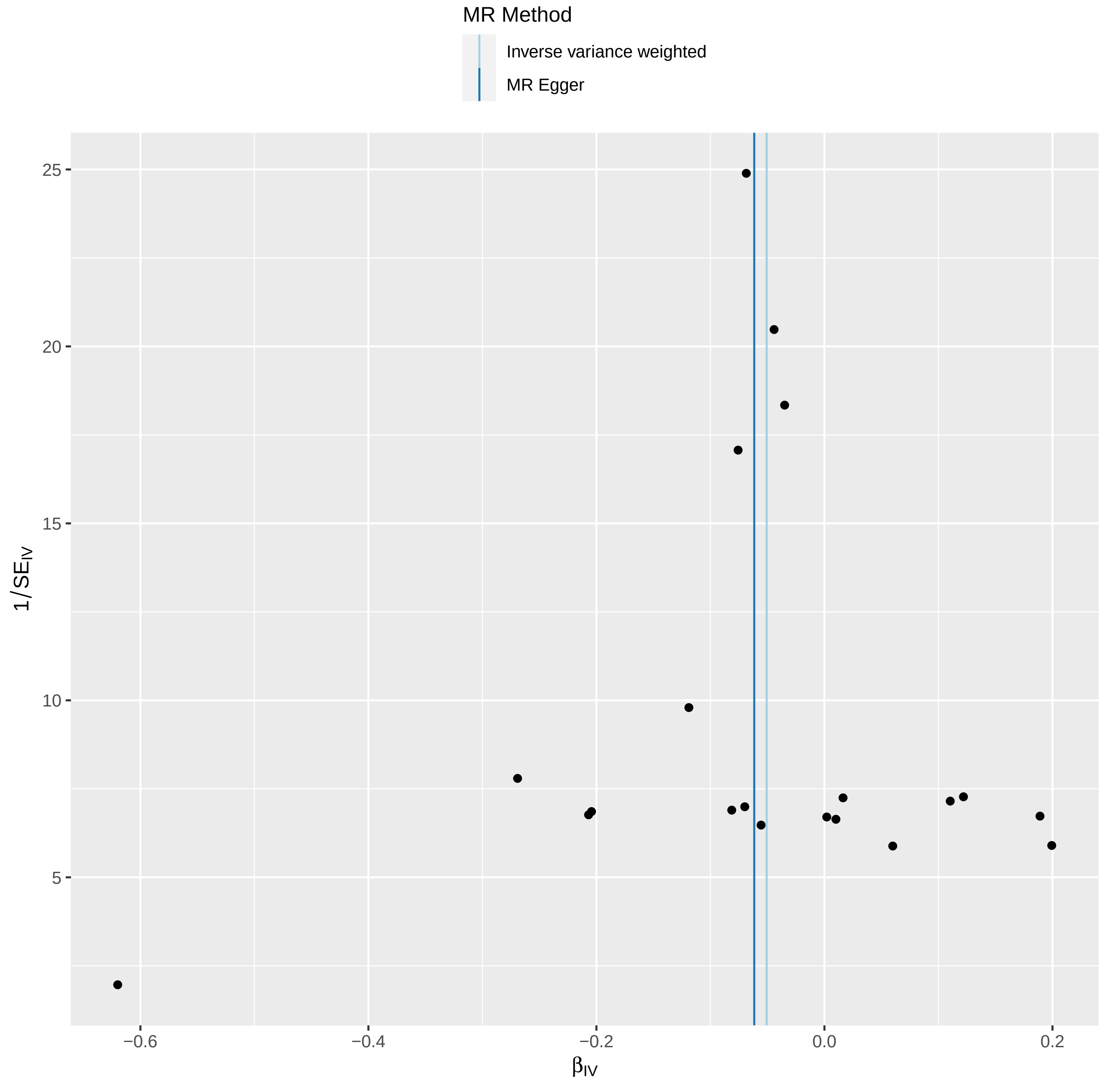

MR Method
Inverse variance weighted
MR Egger
25
20
15
10
5
1/SE_IV
−0.6
−0.4
−0.2
0.0
0.2
β_IV

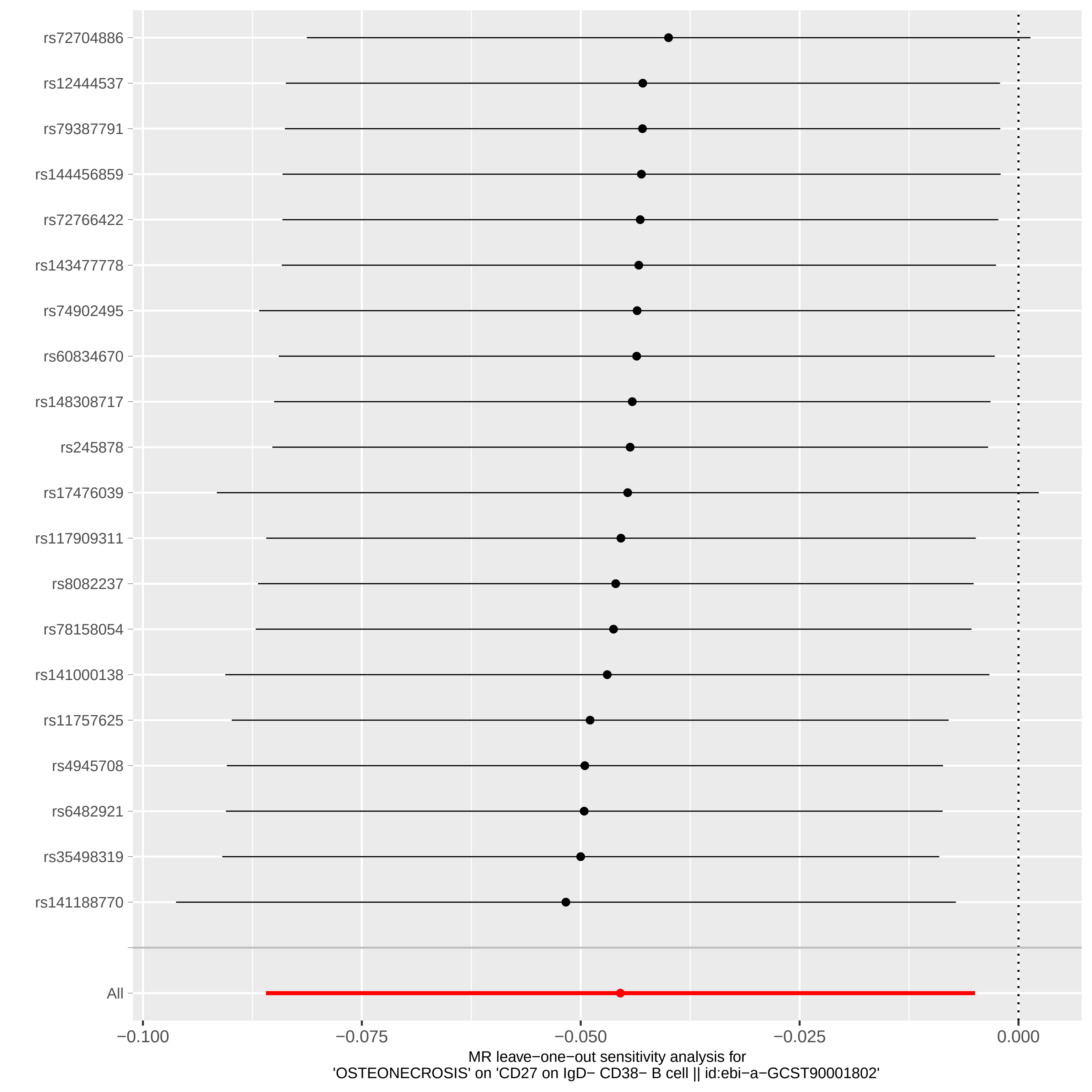

rs72704886
rs12444537
rs79387791
rs144456859
rs72766422
rs143477778
rs74902495
rs60834670
rs148308717
rs245878
rs17476039
rs117909311
rs8082237
rs78158054
rs141000138
rs11757625
rs4945708
rs6482921
rs35498319
rs141188770
All
−0.100
−0.075
−0.050
−0.025
0.000
MR leave−one−out sensitivity analysis for
'OSTEONECROSIS' on 'CD27 on IgD− CD38− B cell || id:ebi−a−GCST90001802'

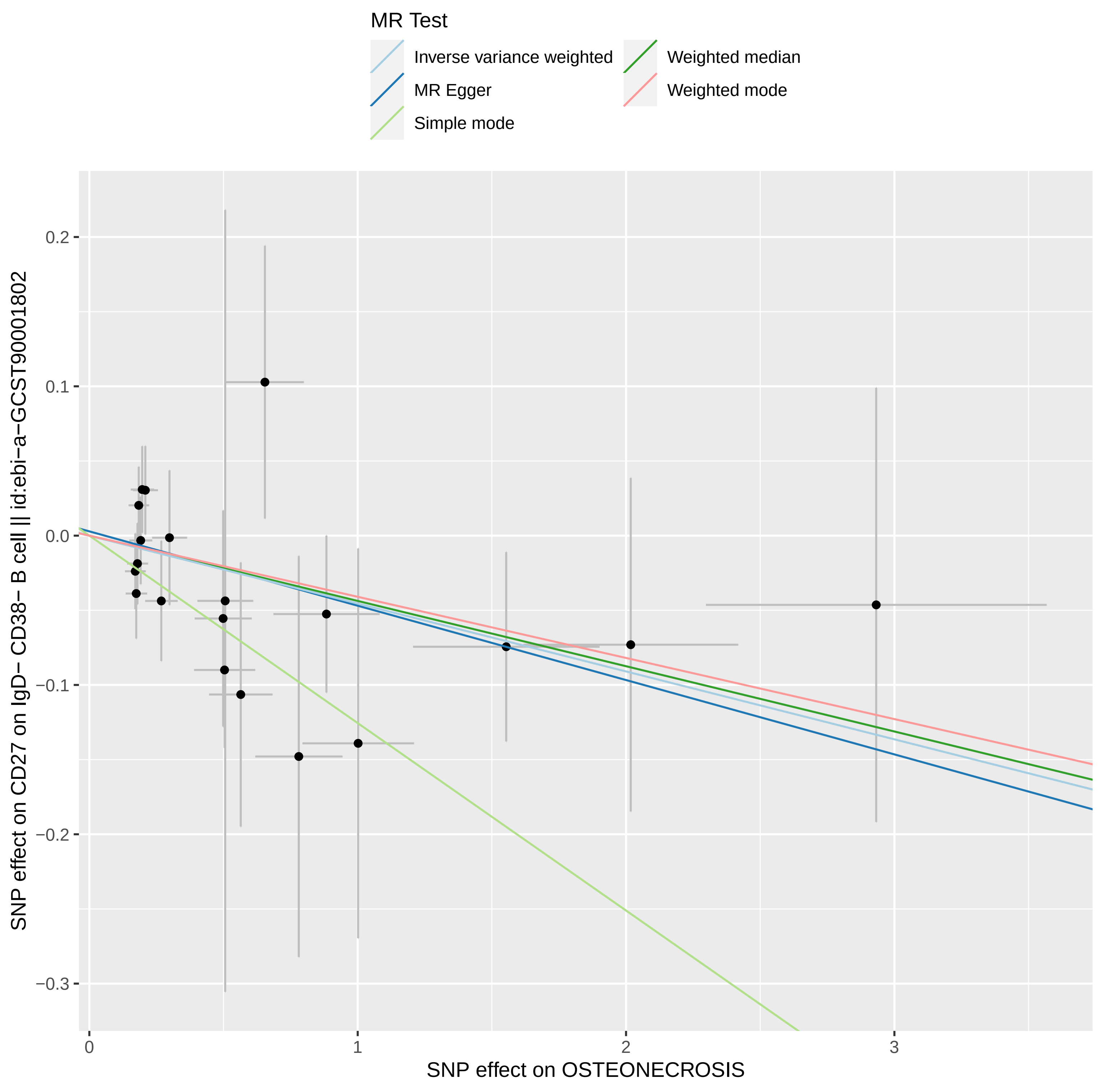

MR Test
Inverse variance weighted
MR Egger
Simple mode
Weighted median
Weighted mode
SNP effect on CD27 on IgD− CD38− B cell || id:ebi−a−GCST90001802
0.2
0.1
0.0
−0.1
−0.2
−0.3
0
1
2
3
SNP effect on OSTEONECROSIS

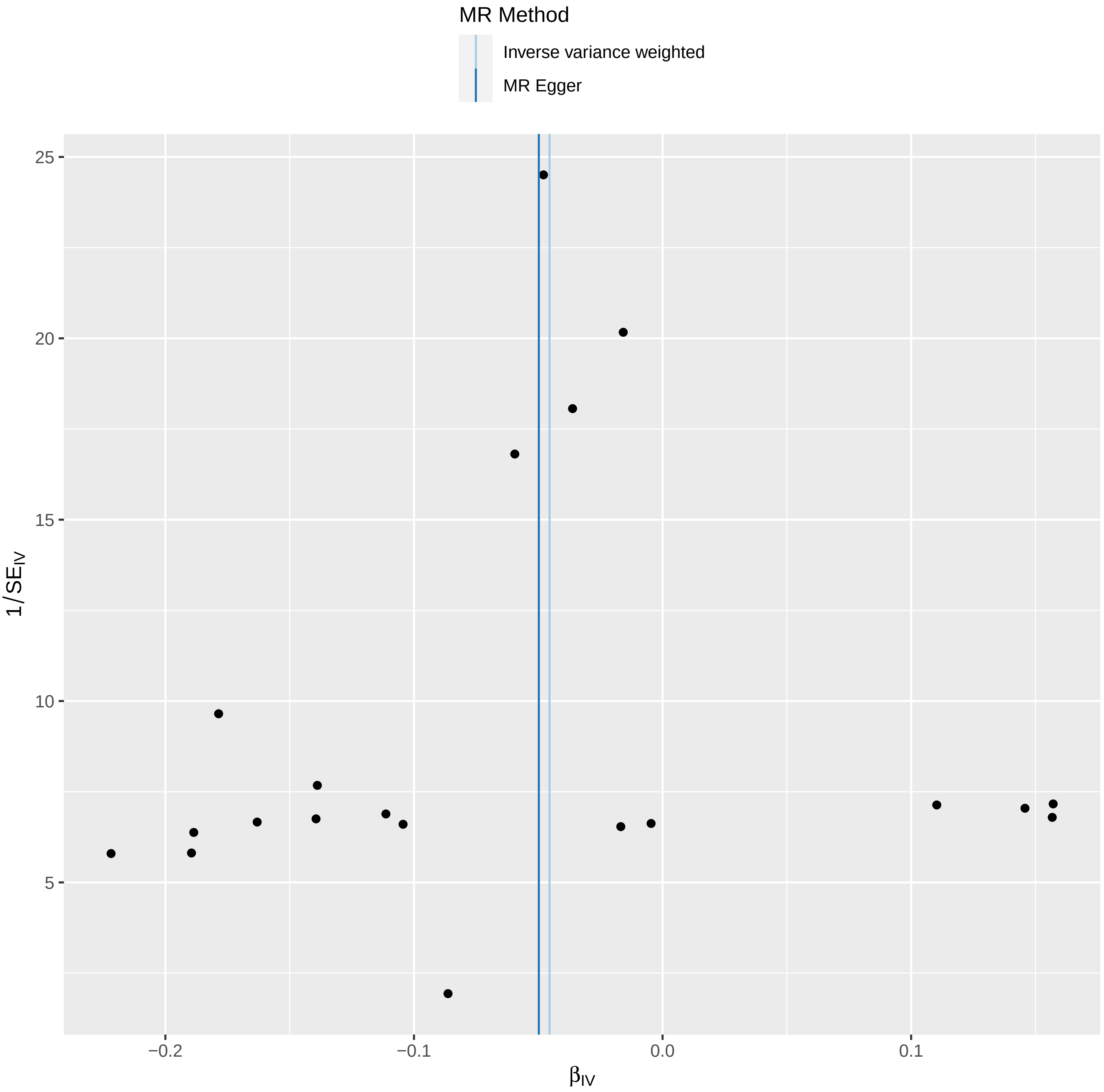

MR Method
Inverse variance weighted
MR Egger
25
20
15
10
5
1/SE_IV
−0.2
−0.1
0.0
0.1
β_IV

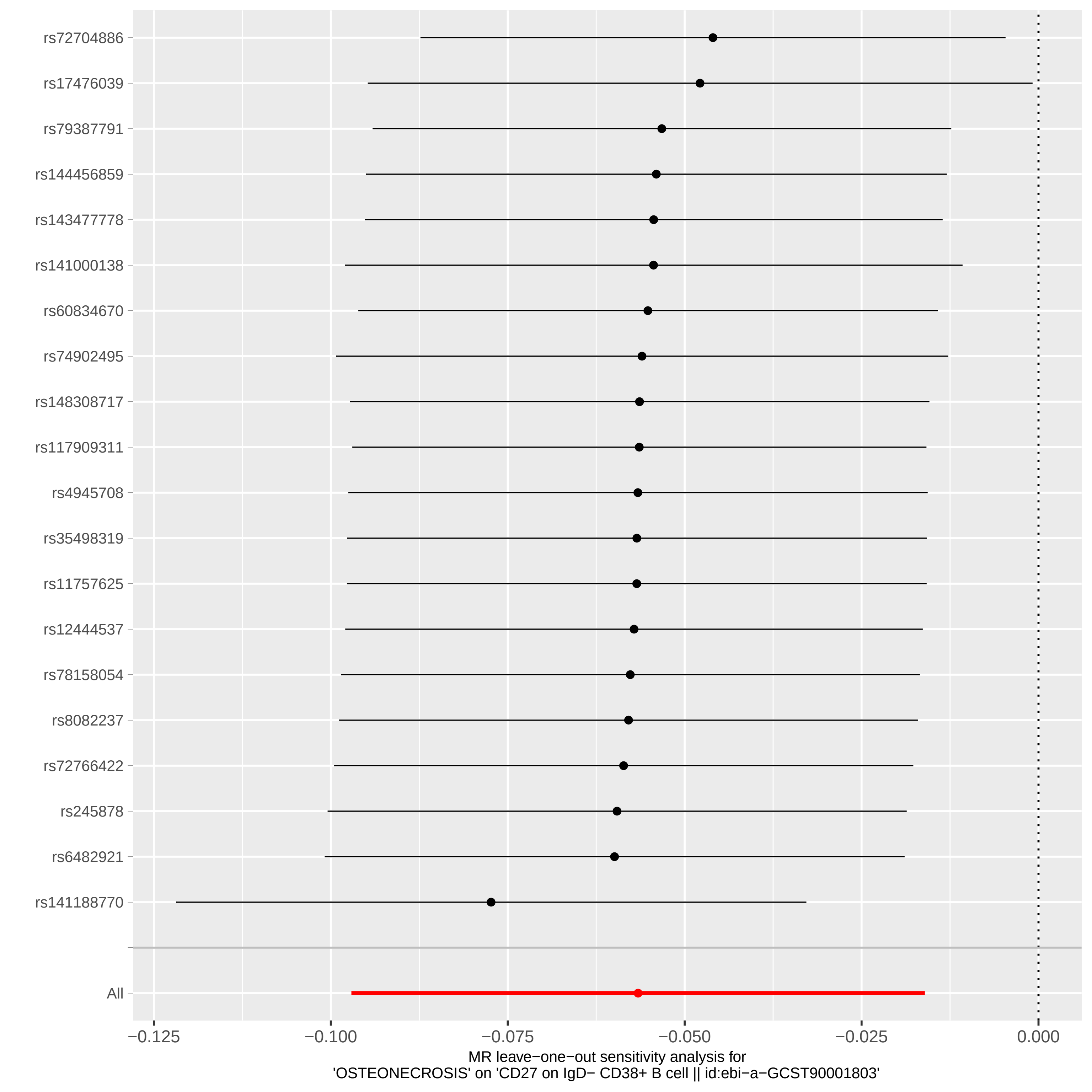
rs72704886
rs17476039
rs79387791
rs144456859
rs143477778
rs141000138
rs60834670
rs74902495
rs148308717
rs117909311
rs4945708
rs35498319
rs11757625
rs12444537
rs78158054
rs8082237
rs72766422
rs245878
rs6482921
rs141188770
All
−0.125
−0.100
−0.075
−0.050
−0.025
0.000
MR leave−one−out sensitivity analysis for
'OSTEONECROSIS' on 'CD27 on IgD− CD38+ B cell || id:ebi−a−GCST90001803'

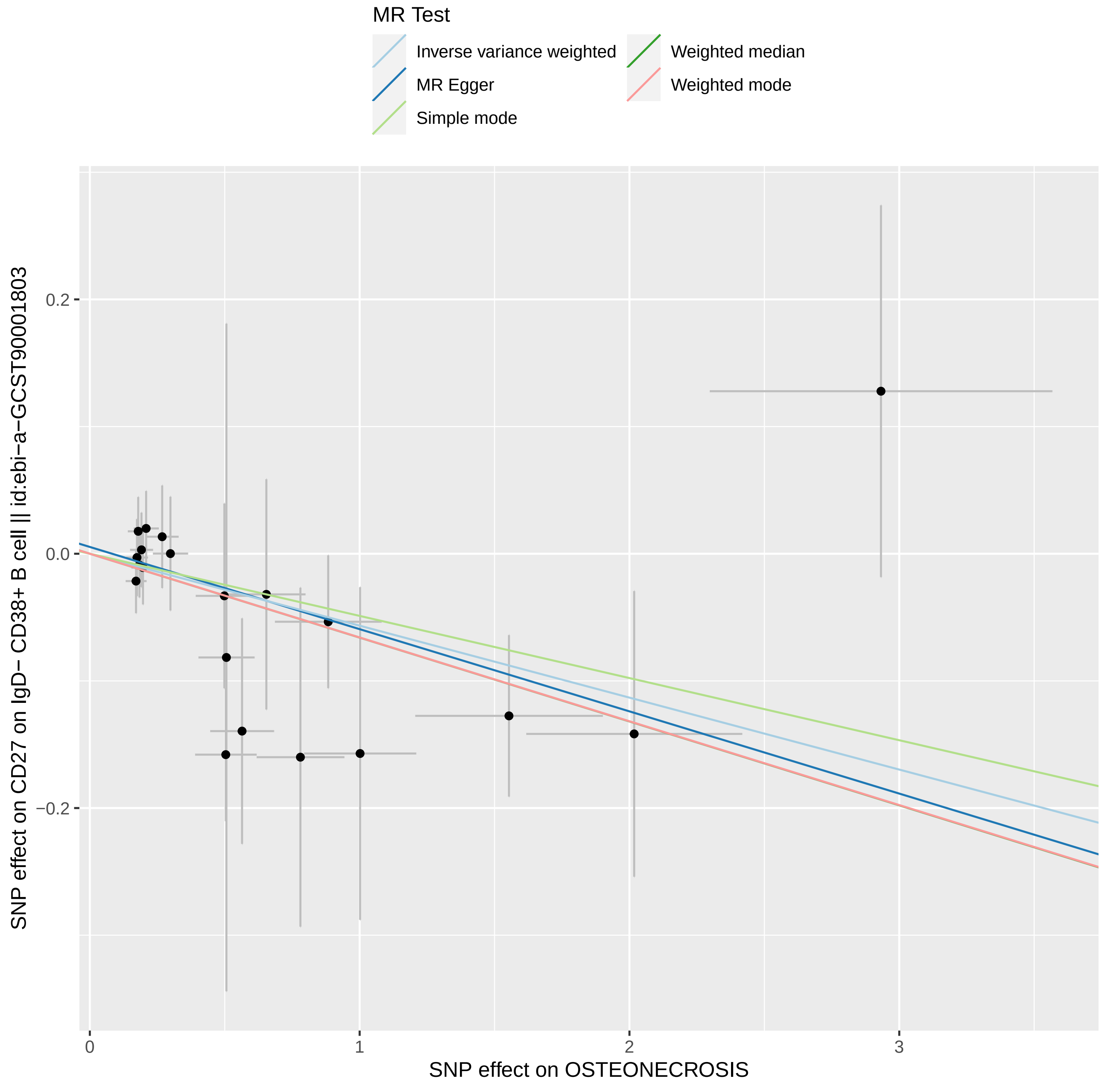

MR Test
Inverse variance weighted
MR Egger
Simple mode
Weighted median
Weighted mode
0.2
0.0
−0.2
0
1
2
3
SNP effect on CD27 on IgD− CD38+ B cell || id:ebi−a−GCST90001803
SNP effect on OSTEONECROSIS

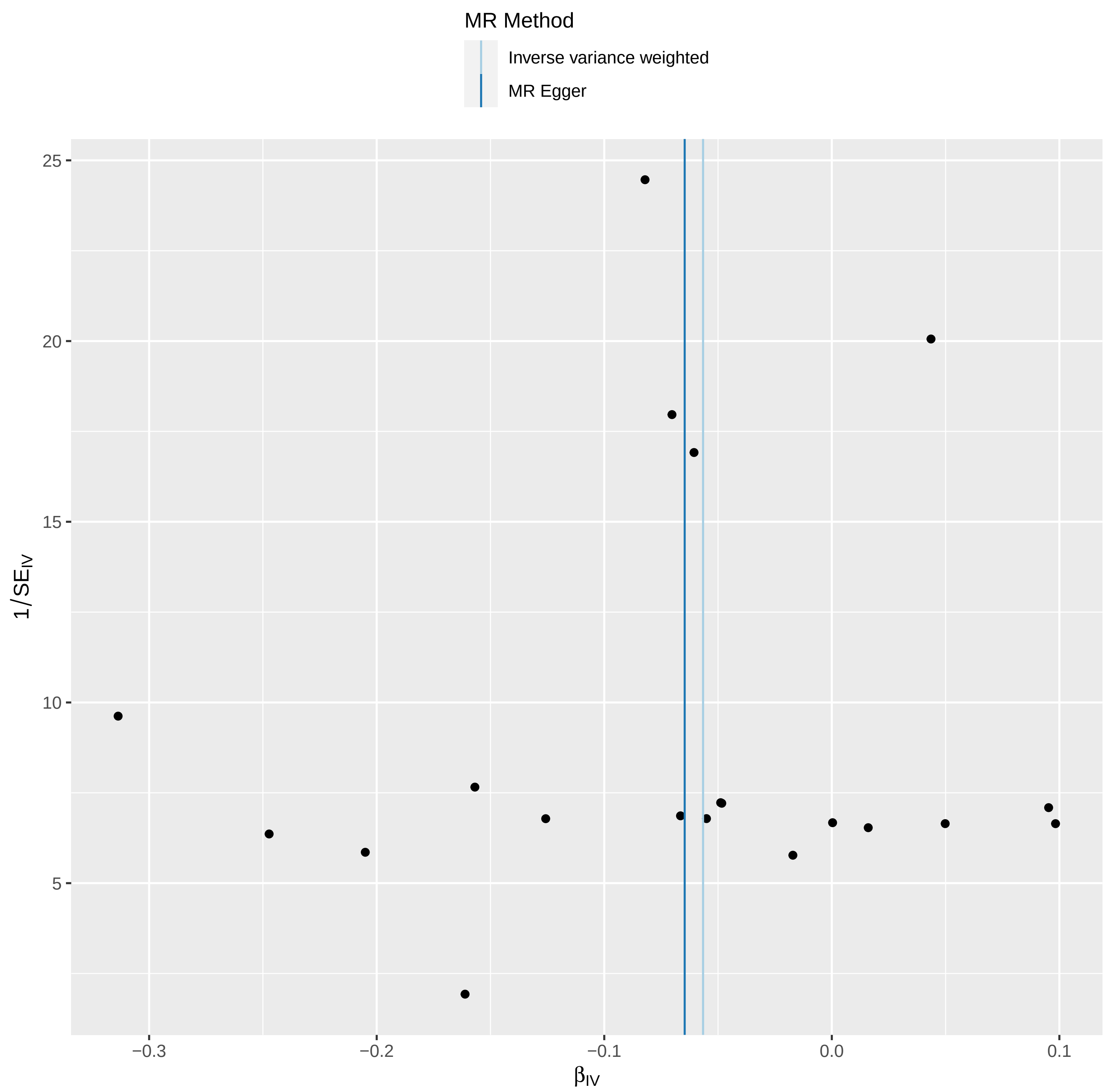
MR Method
Inverse variance weighted
MR Egger
25
20
15
10
5
1/SE_IV
−0.3
−0.2
−0.1
0.0
0.1
β_IV

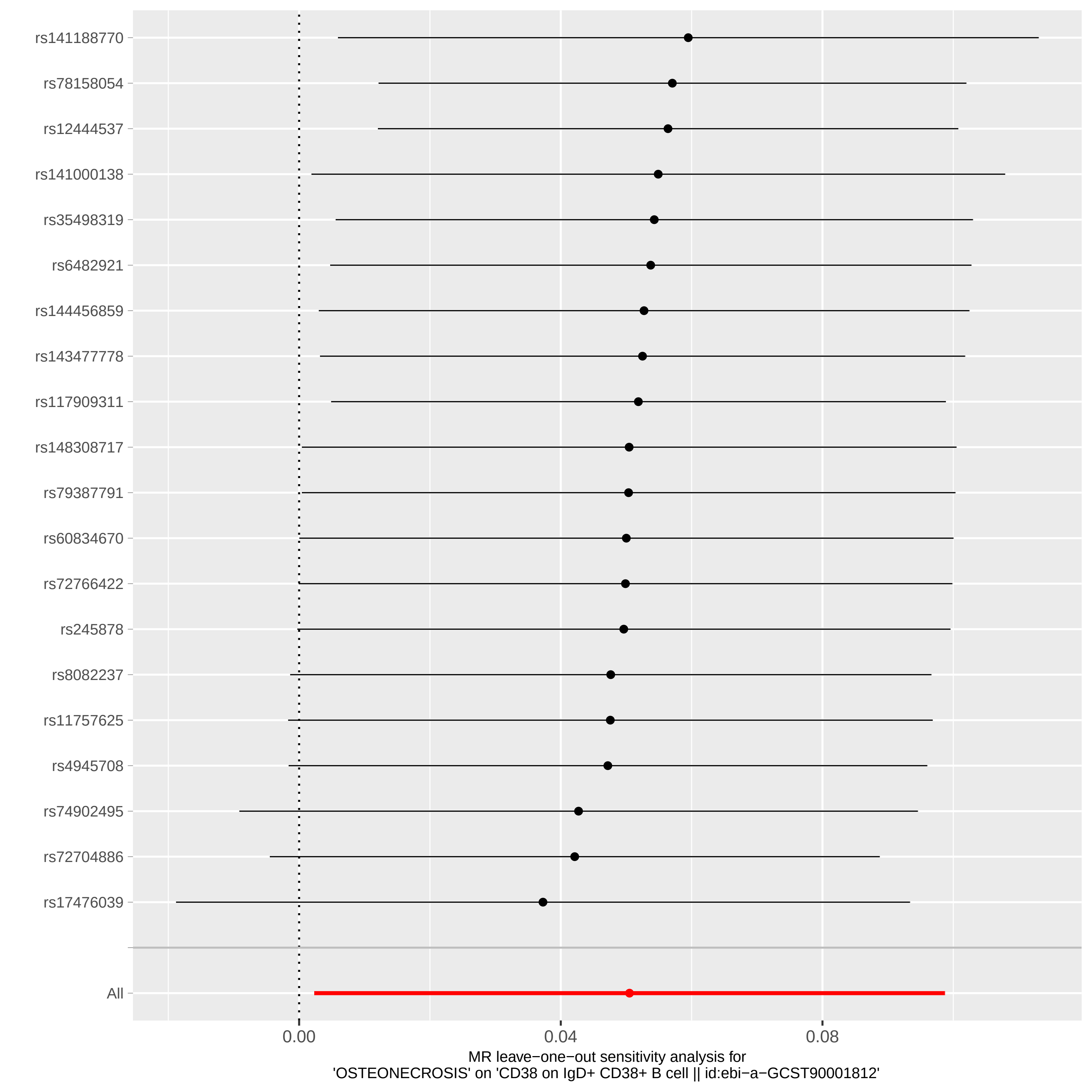
rs141188770
rs78158054
rs12444537
rs141000138
rs35498319
rs6482921
rs144456859
rs143477778
rs117909311
rs148308717
rs79387791
rs60834670
rs72766422
rs245878
rs8082237
rs11757625
rs4945708
rs74902495
rs72704886
rs17476039
All
0.00
0.04
0.08
MR leave−one−out sensitivity analysis for
'OSTEONECROSIS' on 'CD38 on IgD+ CD38+ B cell || id:ebi−a−GCST90001812'

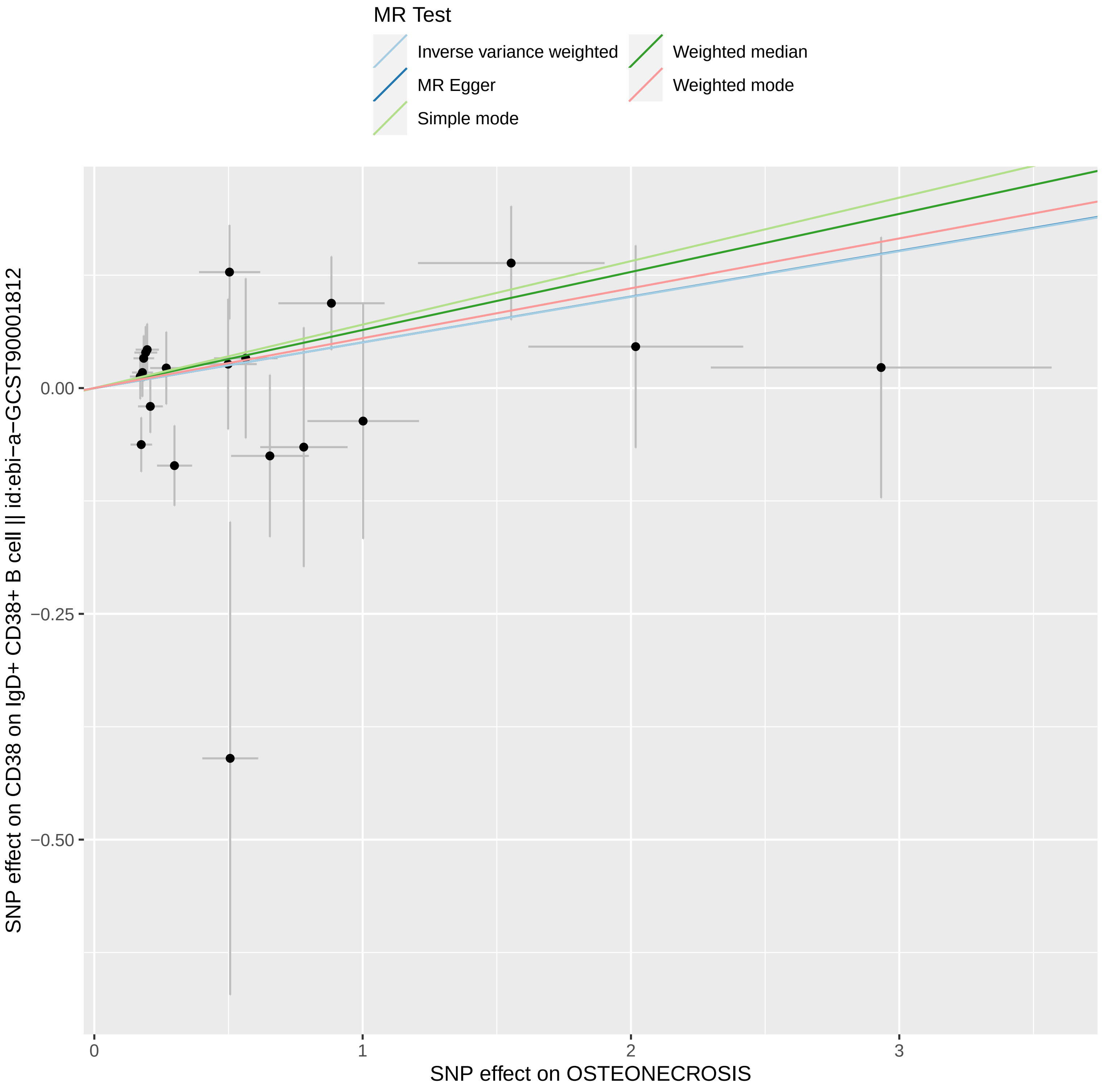
MR Test
Inverse variance weighted
MR Egger
Simple mode
Weighted median
Weighted mode
SNP effect on CD38 on IgD+ CD38+ B cell || id:ebi−a−GCST90001812
0.00
−0.25
−0.50
0
1
2
3
SNP effect on OSTEONECROSIS

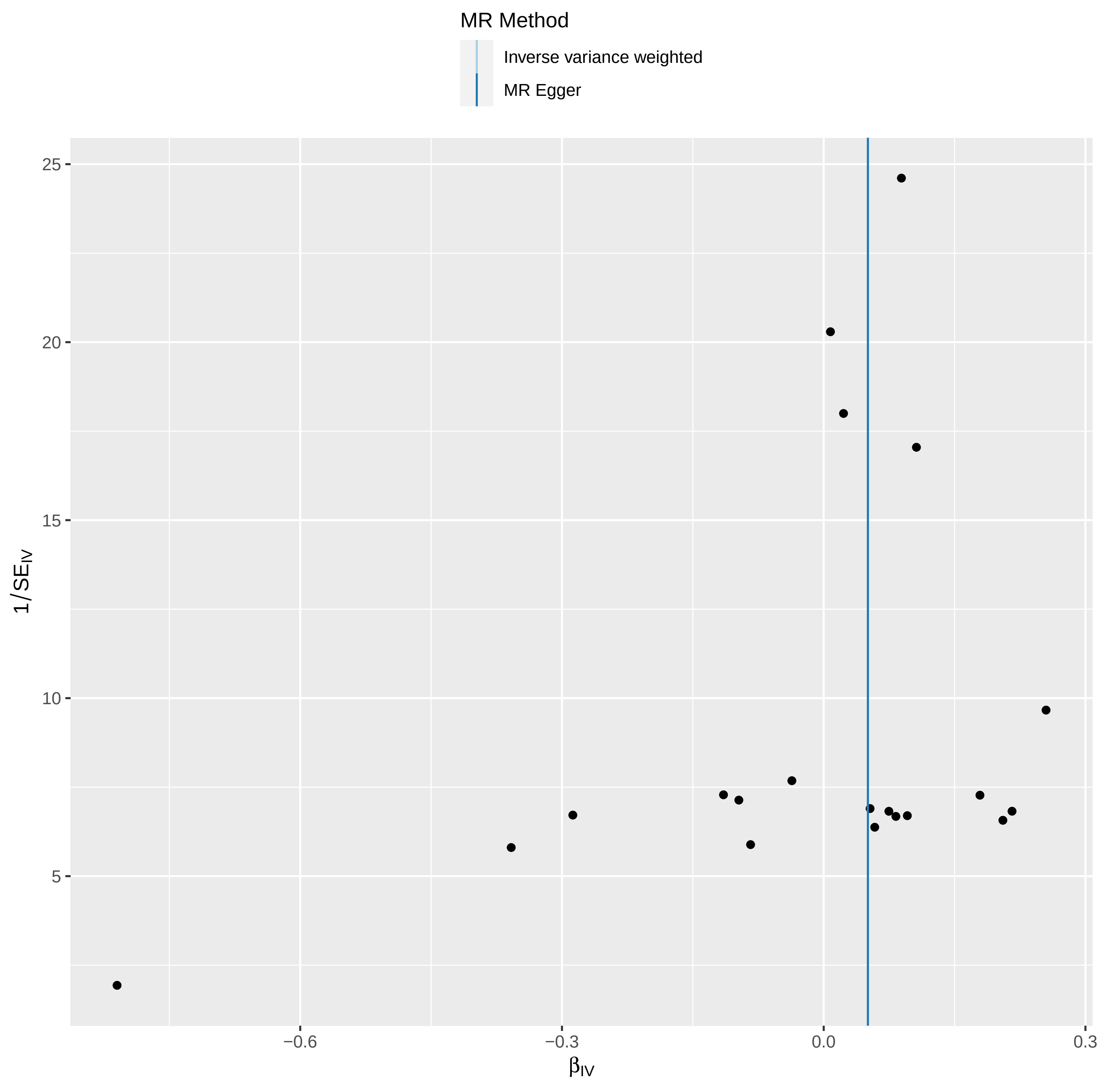
MR Method
Inverse variance weighted
MR Egger
25
20
15
10
5
1/SE$_{IV}$
−0.6
−0.3
0.0
0.3
β$_{IV}$

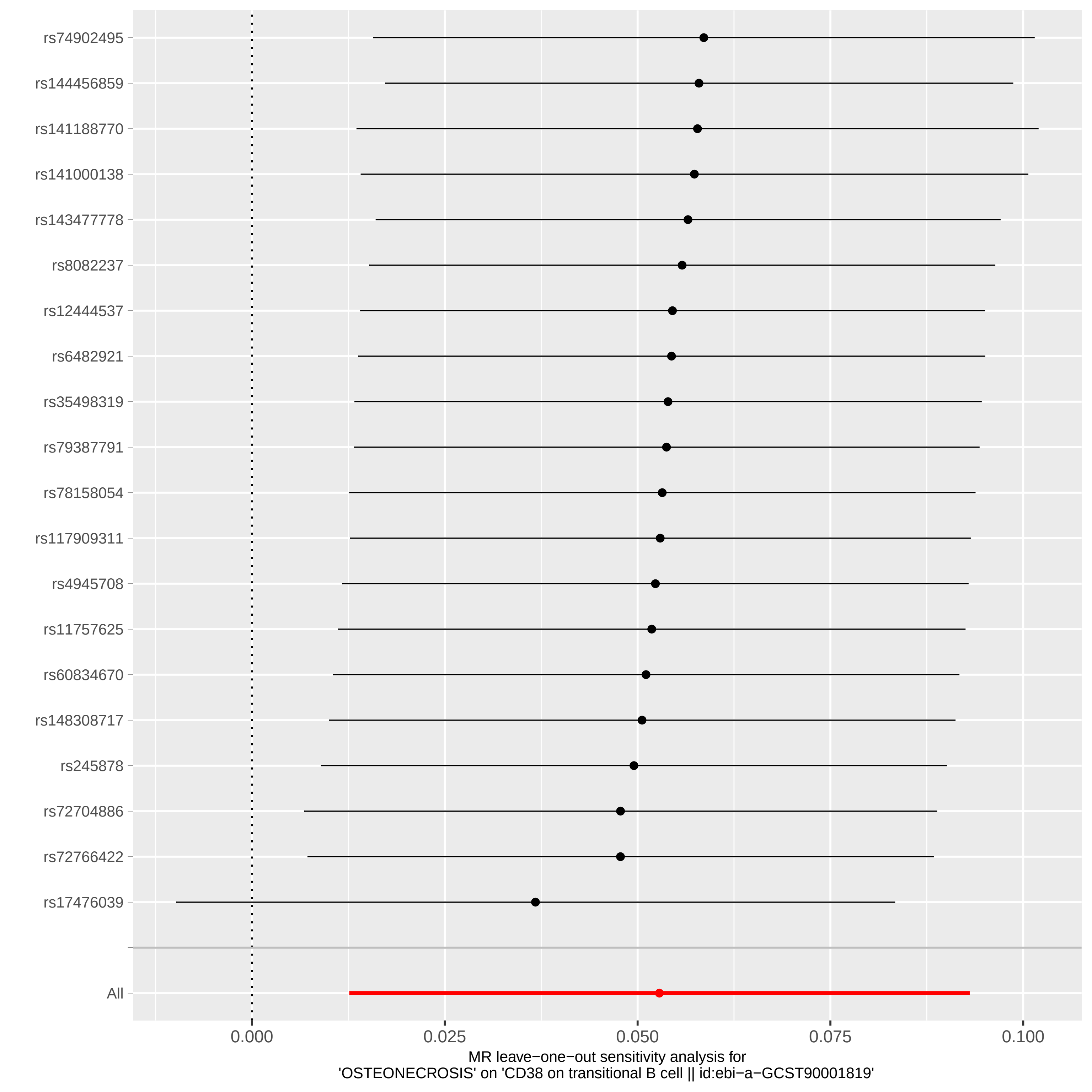
rs74902495
rs144456859
rs141188770
rs141000138
rs143477778
rs8082237
rs12444537
rs6482921
rs35498319
rs79387791
rs78158054
rs117909311
rs4945708
rs11757625
rs60834670
rs148308717
rs245878
rs72704886
rs72766422
rs17476039
All
0.000
0.025
0.050
0.075
0.100
MR leave−one−out sensitivity analysis for
'OSTEONECROSIS' on 'CD38 on transitional B cell || id:ebi−a−GCST90001819'

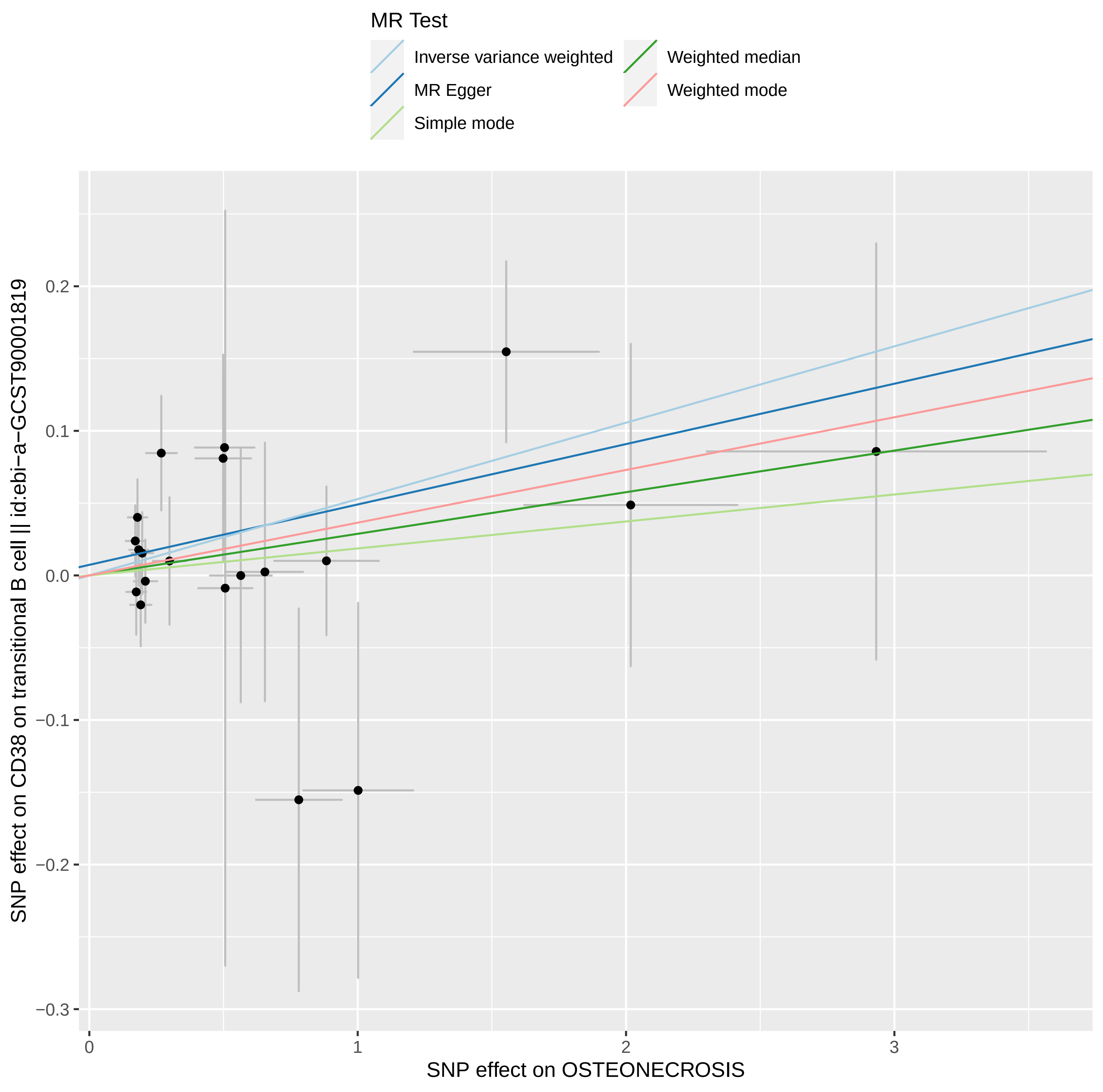
MR Test
Inverse variance weighted
MR Egger
Simple mode
Weighted median
Weighted mode
SNP effect on CD38 on transitional B cell || id:ebi−a−GCST90001819
0.2
0.1
0.0
−0.1
−0.2
−0.3
0
1
2
3
SNP effect on OSTEONECROSIS

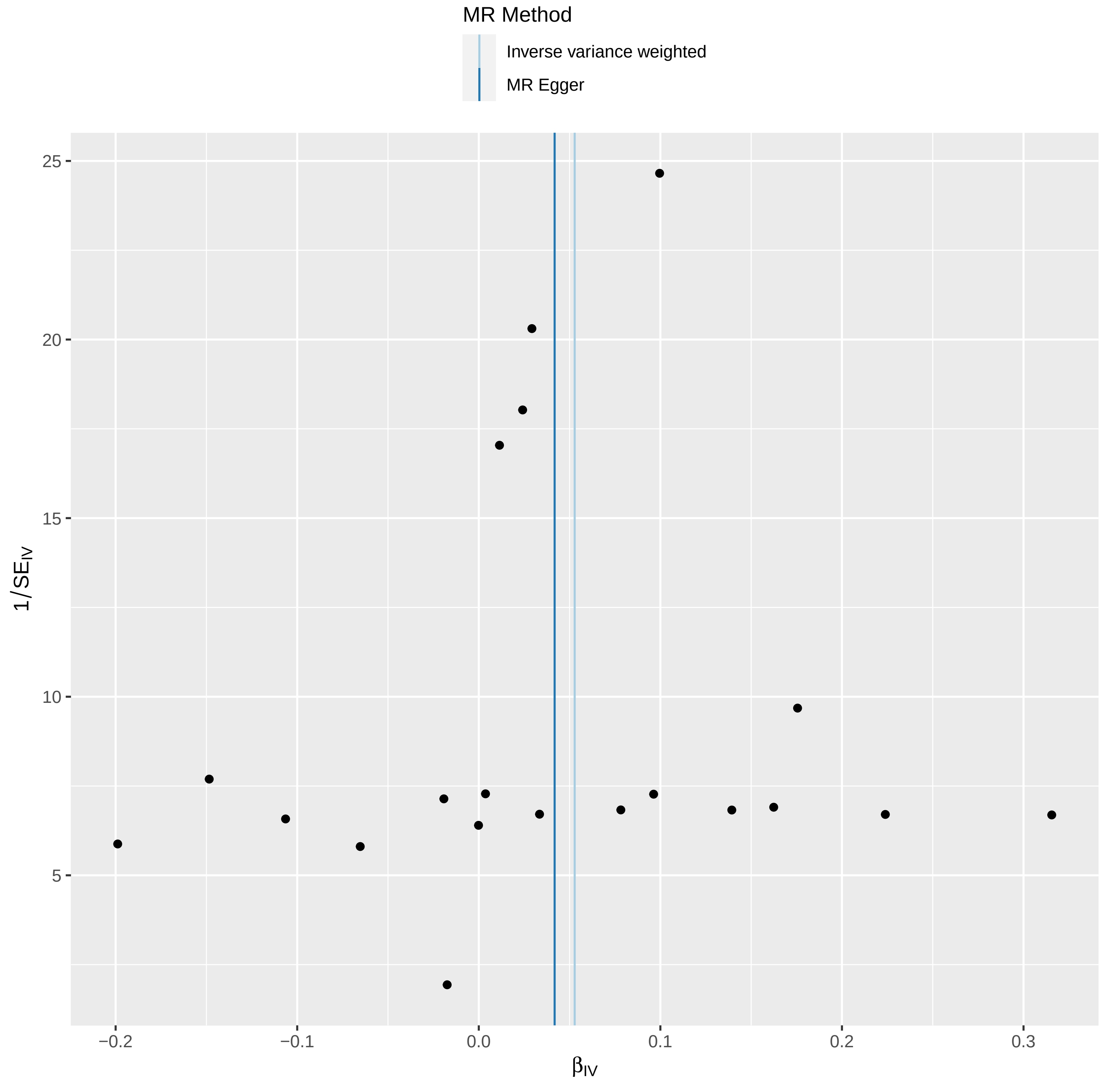
MR Method
Inverse variance weighted
MR Egger
25
20
15
10
5
1/SE$_{IV}$
−0.2
−0.1
0.0
0.1
0.2
0.3
β$_{IV}$

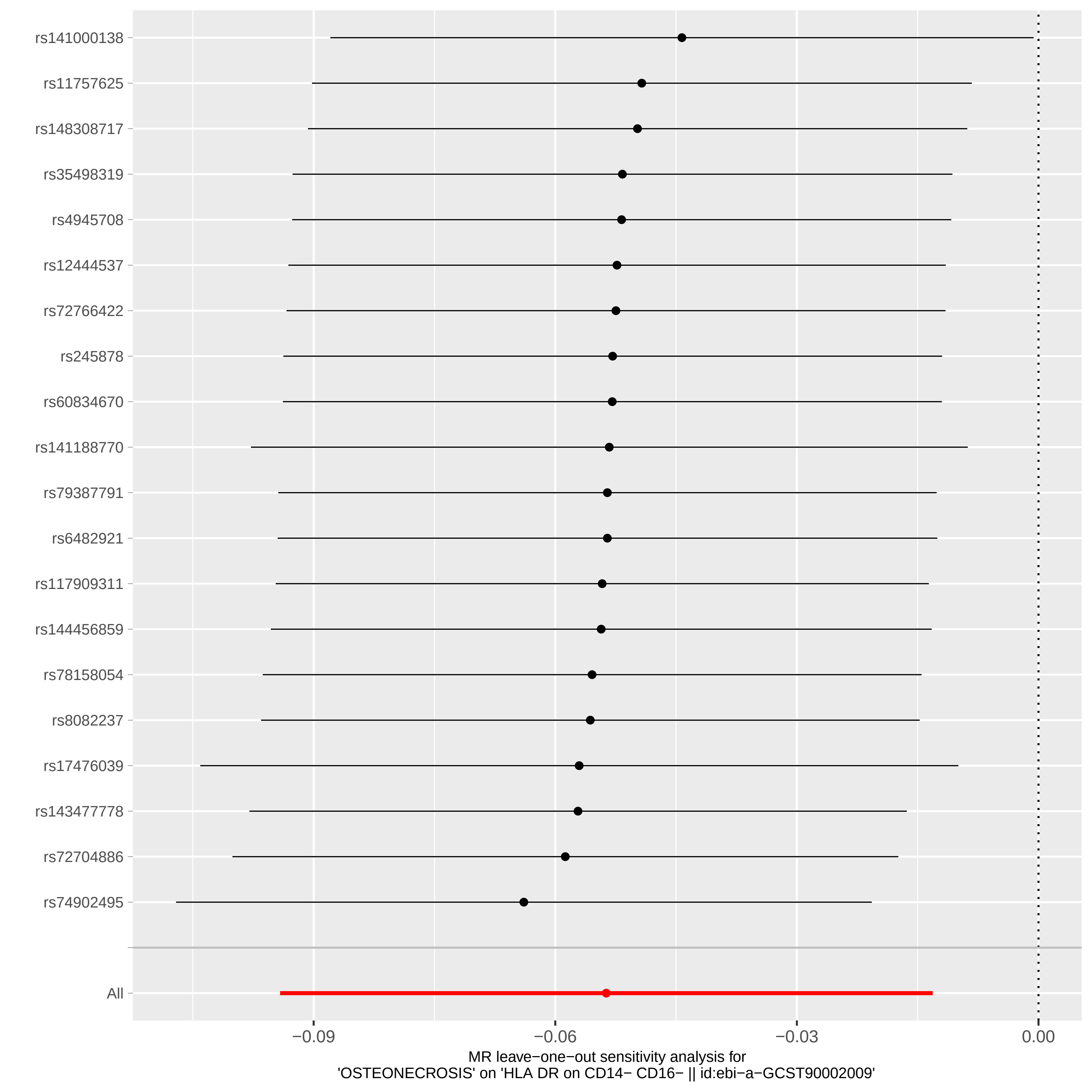
rs141000138
rs11757625
rs148308717
rs35498319
rs4945708
rs12444537
rs72766422
rs245878
rs60834670
rs141188770
rs79387791
rs6482921
rs117909311
rs144456859
rs78158054
rs8082237
rs17476039
rs143477778
rs72704886
rs74902495
All
−0.09
−0.06
−0.03
0.00
MR leave−one−out sensitivity analysis for
'OSTEONECROSIS' on 'HLA DR on CD14− CD16− || id:ebi−a−GCST90002009'

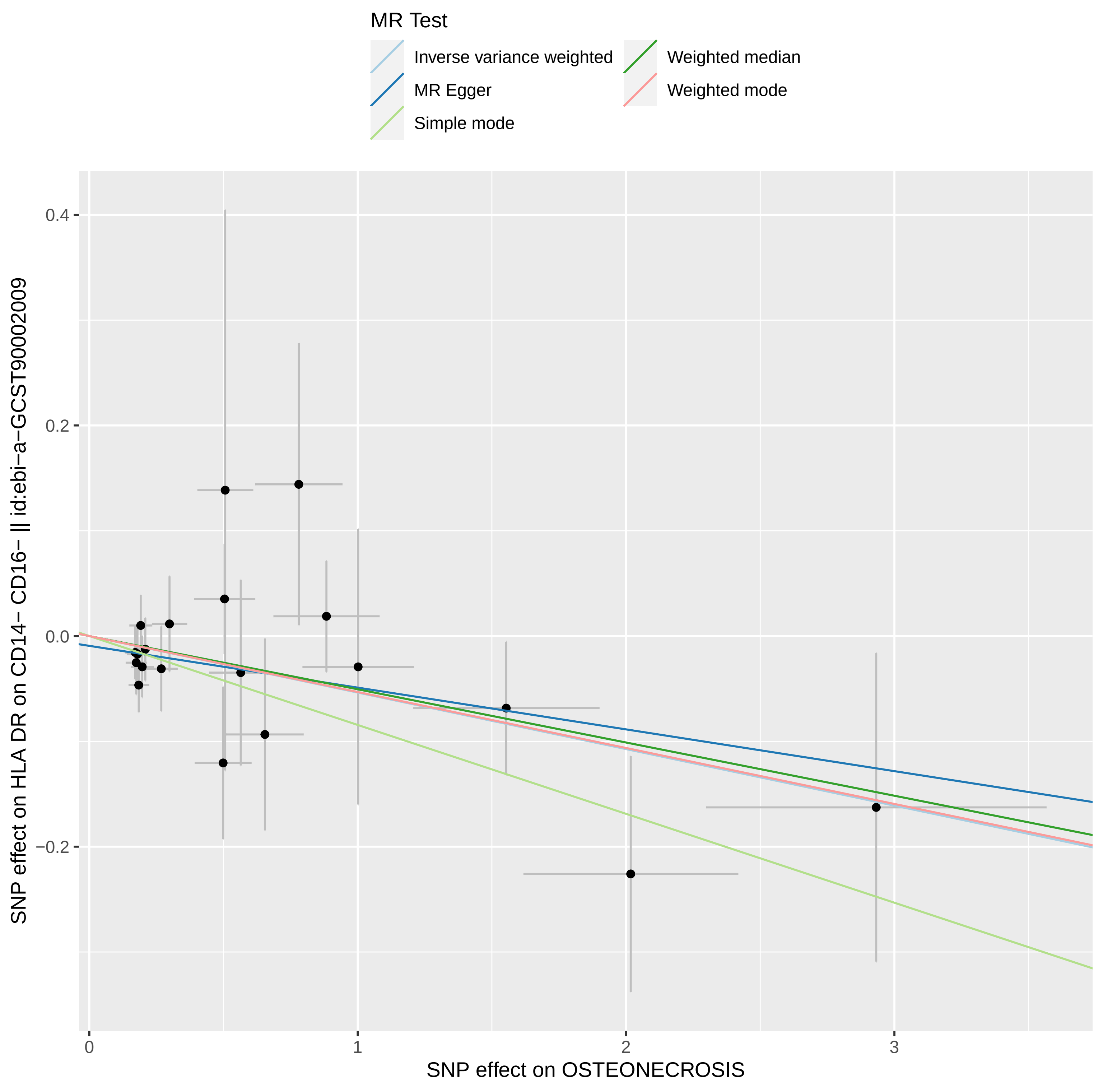
MR Test
Inverse variance weighted
MR Egger
Simple mode
Weighted median
Weighted mode
0.4
0.2
0.0
−0.2
0
1
2
3
SNP effect on HLA DR on CD14− CD16− || id:ebi−a−GCST90002009
SNP effect on OSTEONECROSIS

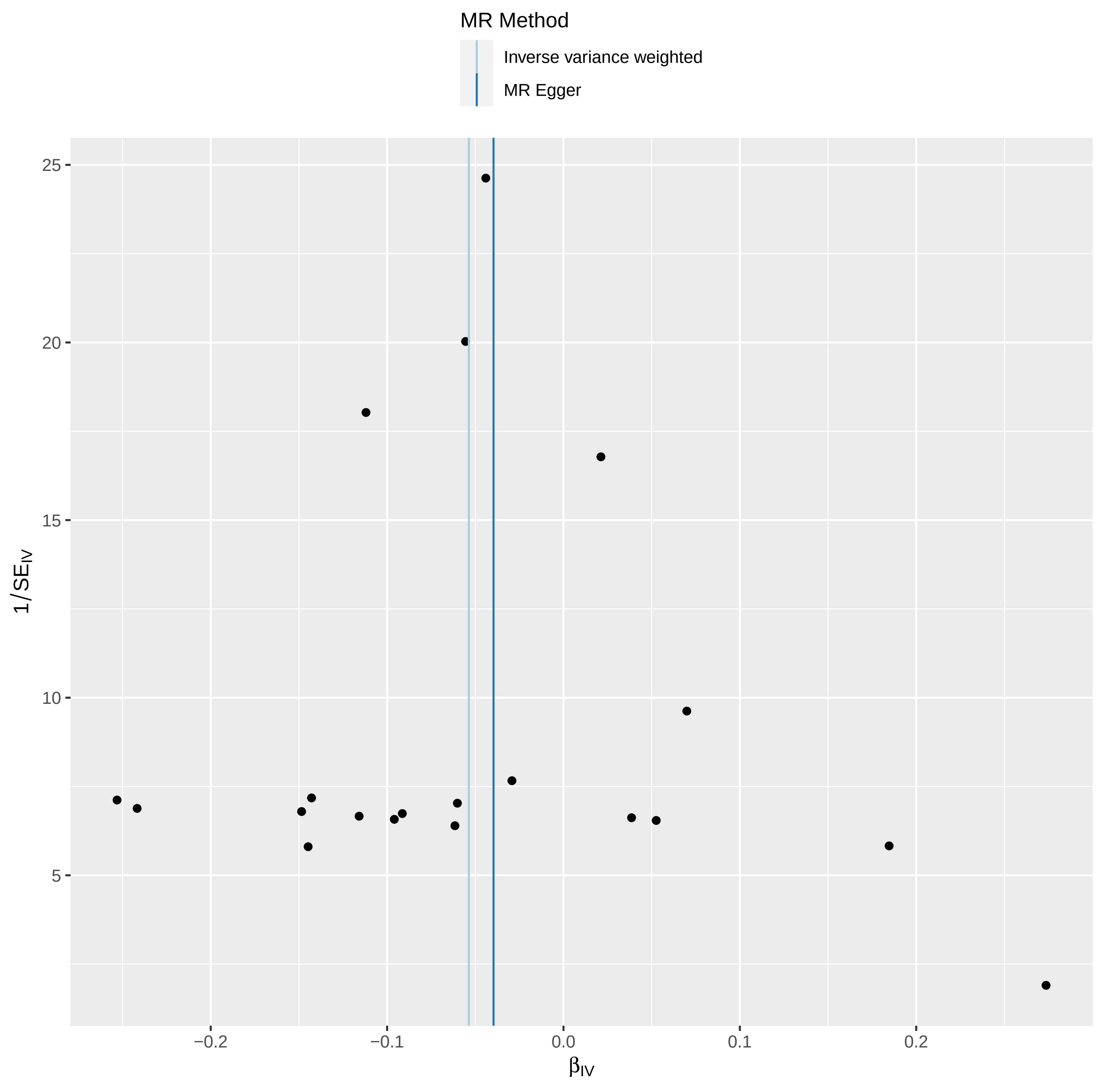
MR Method
Inverse variance weighted
MR Egger
1/SE$_{IV}$
25
20
15
10
5
−0.2
−0.1
0.0
0.1
0.2
β$_{IV}$

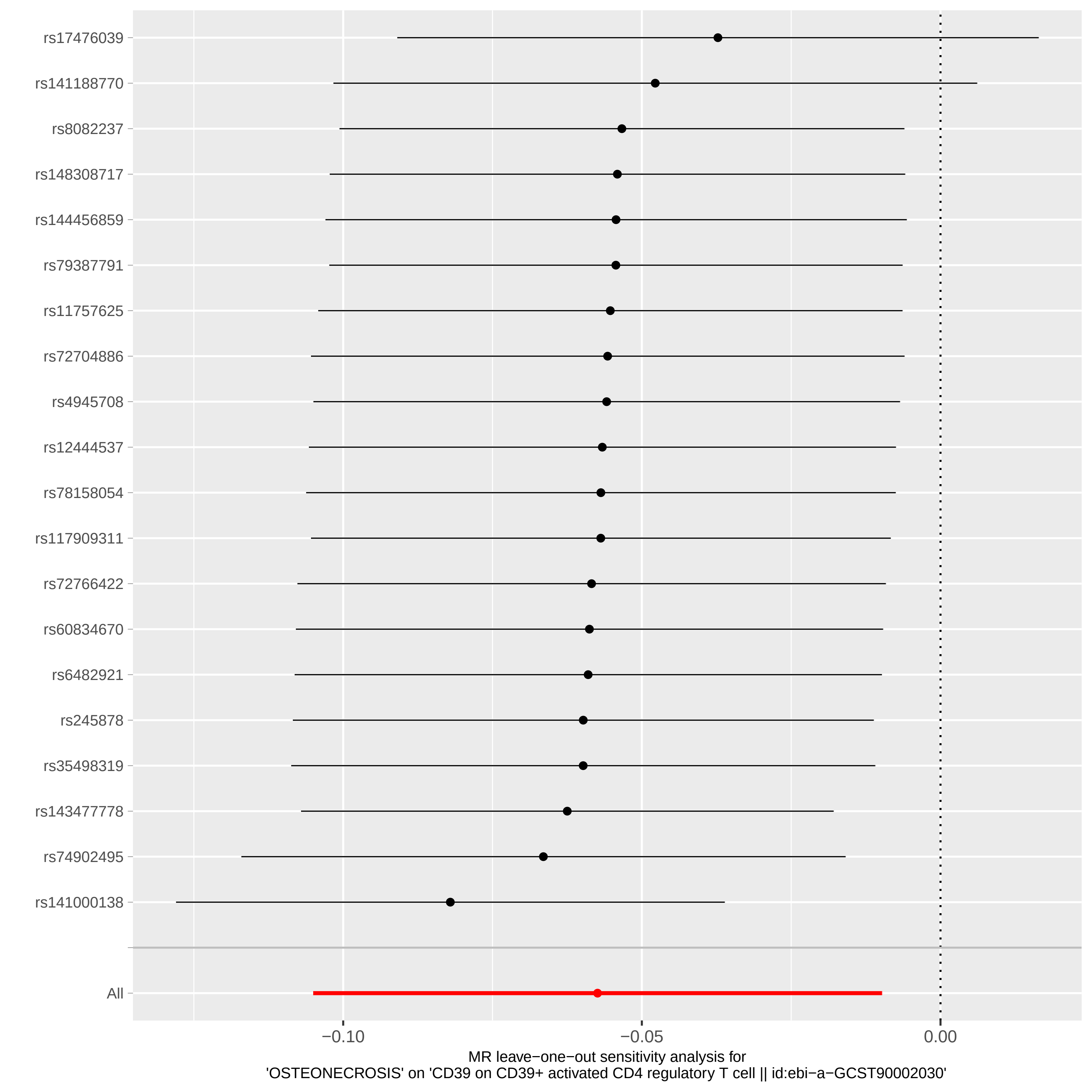
rs17476039
rs141188770
rs8082237
rs148308717
rs144456859
rs79387791
rs11757625
rs72704886
rs4945708
rs12444537
rs78158054
rs117909311
rs72766422
rs60834670
rs6482921
rs245878
rs35498319
rs143477778
rs74902495
rs141000138
All
−0.10
−0.05
0.00
MR leave−one−out sensitivity analysis for
'OSTEONECROSIS' on 'CD39 on CD39+ activated CD4 regulatory T cell || id:ebi−a−GCST90002030'

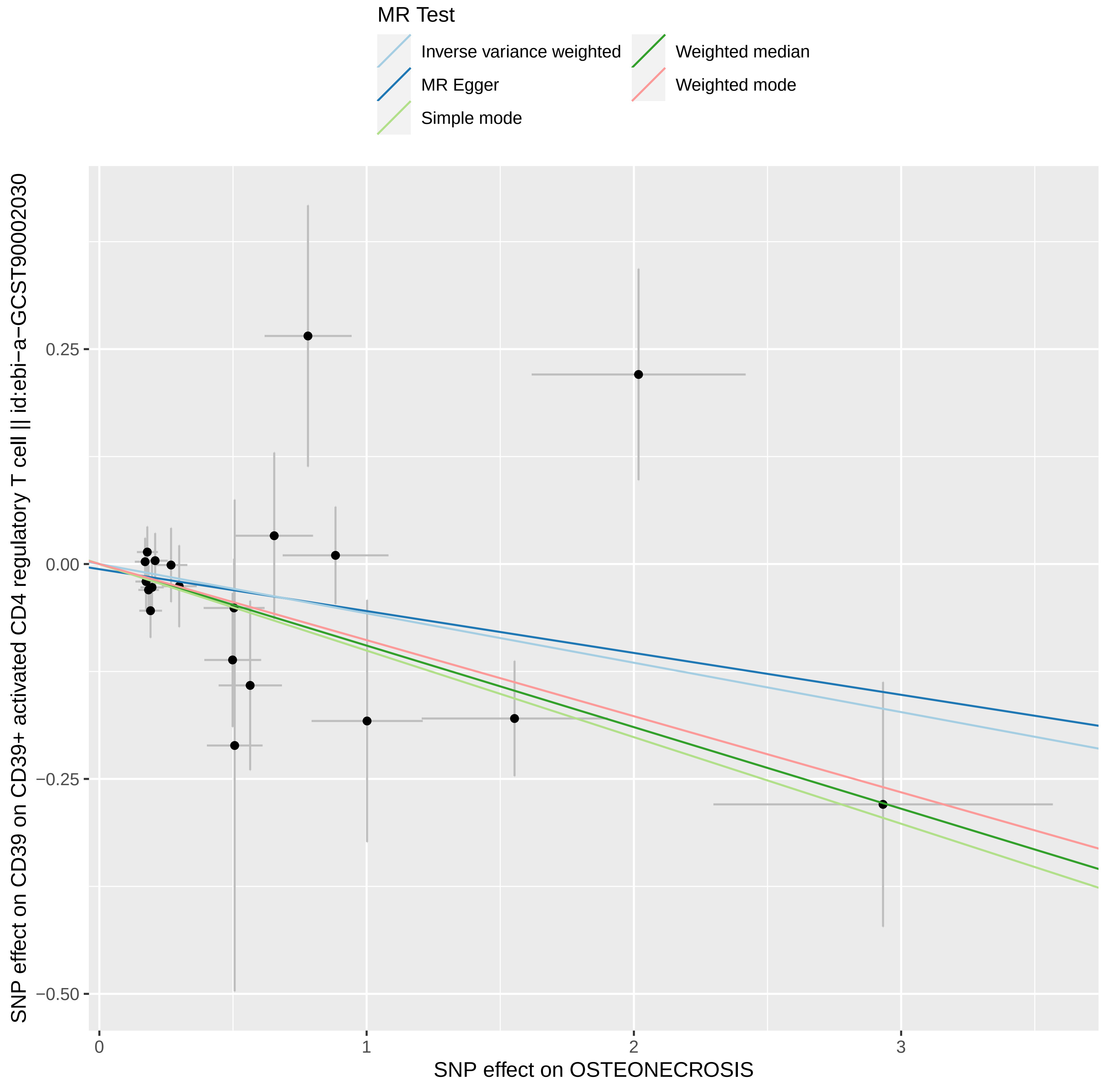

MR Test
Inverse variance weighted
MR Egger
Simple mode
Weighted median
Weighted mode
SNP effect on CD39 on CD39+ activated CD4 regulatory T cell || id:ebi−a−GCST90002030
0.25
0.00
−0.25
−0.50
0
1
2
3
SNP effect on OSTEONECROSIS

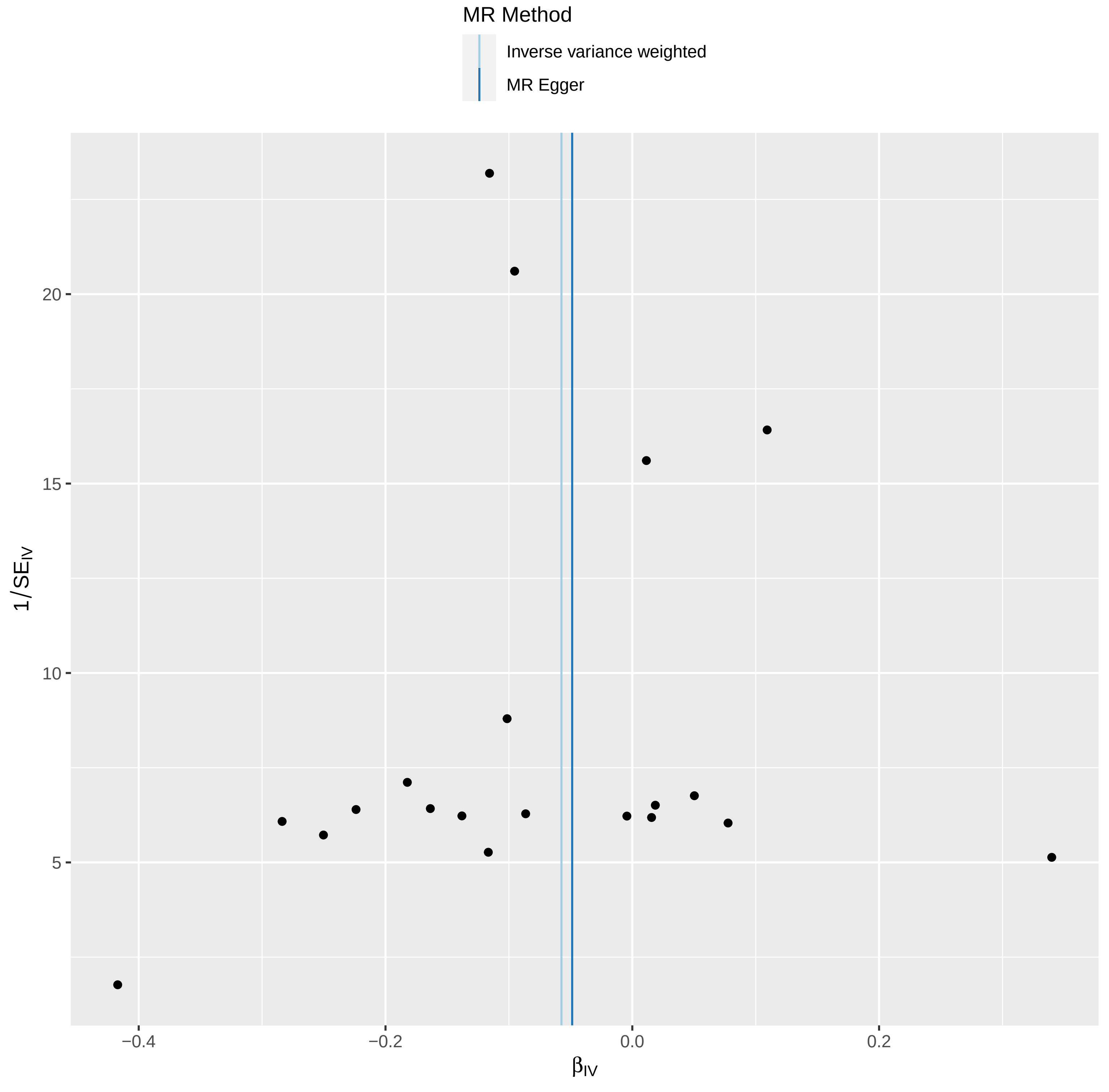
MR Method
Inverse variance weighted
MR Egger
20
15
10
5
1/SE_IV
−0.4
−0.2
0.0
0.2
β_IV

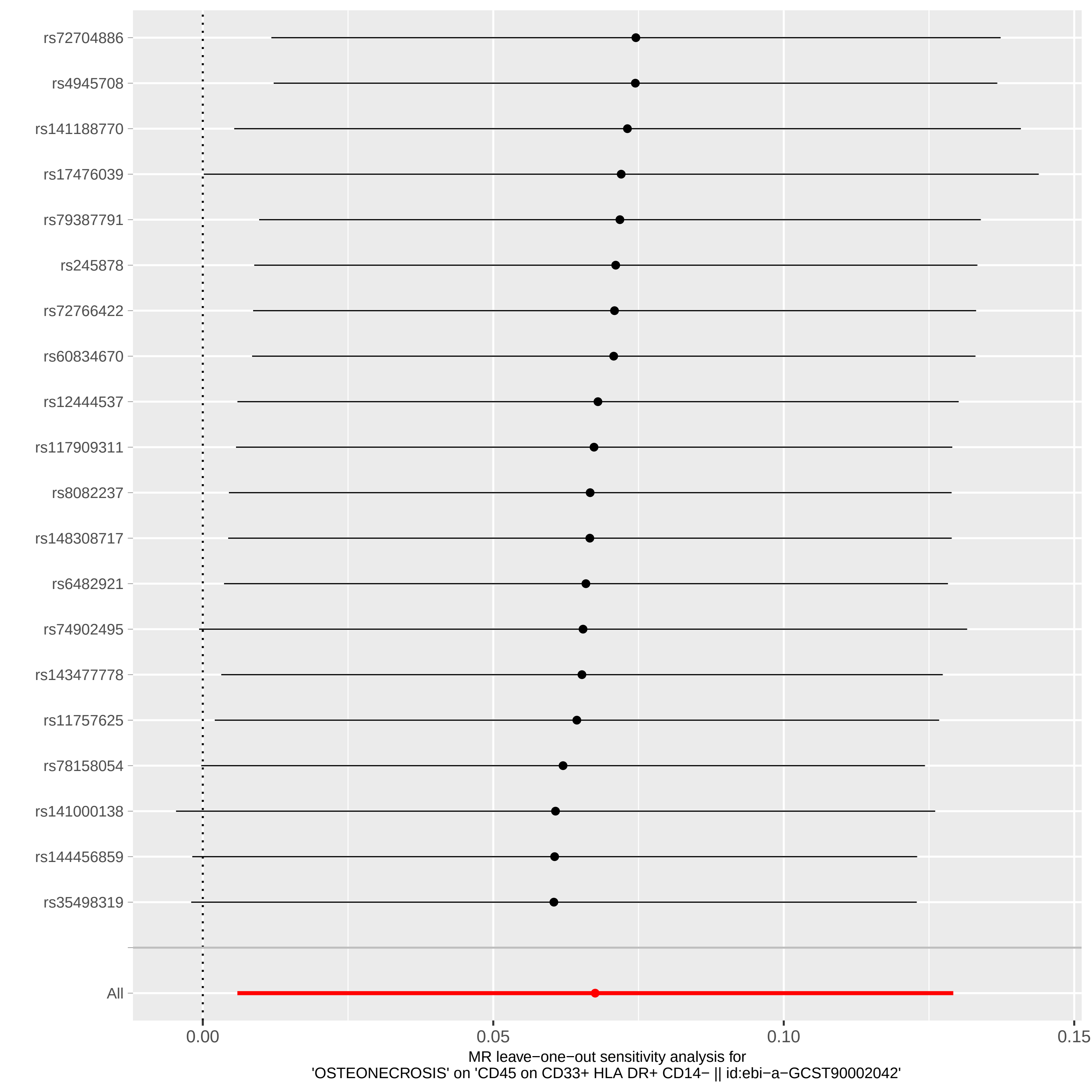

rs72704886
rs4945708
rs141188770
rs17476039
rs79387791
rs245878
rs72766422
rs60834670
rs12444537
rs117909311
rs8082237
rs148308717
rs6482921
rs74902495
rs143477778
rs11757625
rs78158054
rs141000138
rs144456859
rs35498319
All
0.00
0.05
0.10
0.15
MR leave−one−out sensitivity analysis for
'OSTEONECROSIS' on 'CD45 on CD33+ HLA DR+ CD14− || id:ebi−a−GCST90002042'

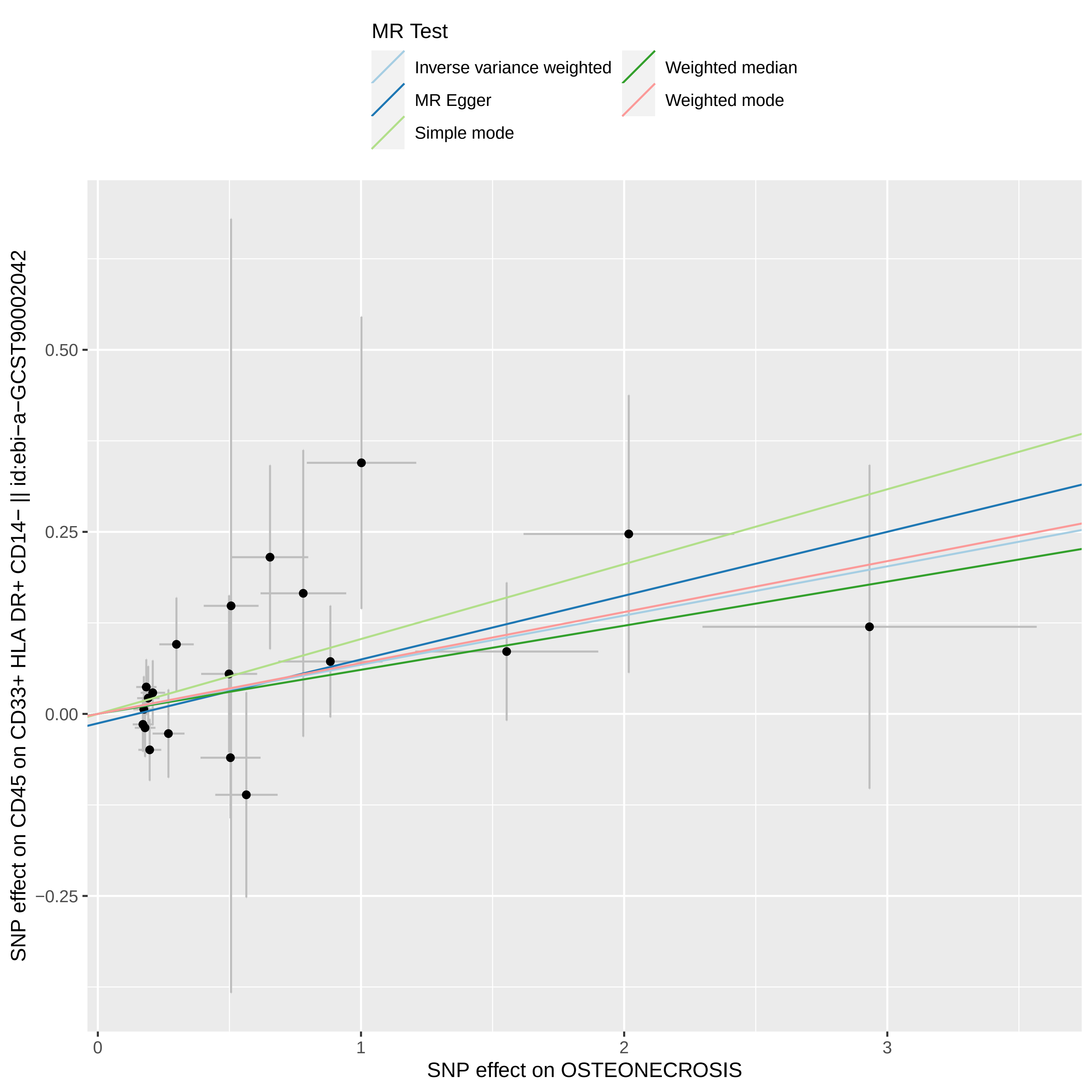
MR Test
Inverse variance weighted
MR Egger
Simple mode
Weighted median
Weighted mode
SNP effect on CD45 on CD33+ HLA DR+ CD14− || id:ebi−a−GCST90002042
0.50
0.25
0.00
−0.25
0
1
2
3
SNP effect on OSTEONECROSIS

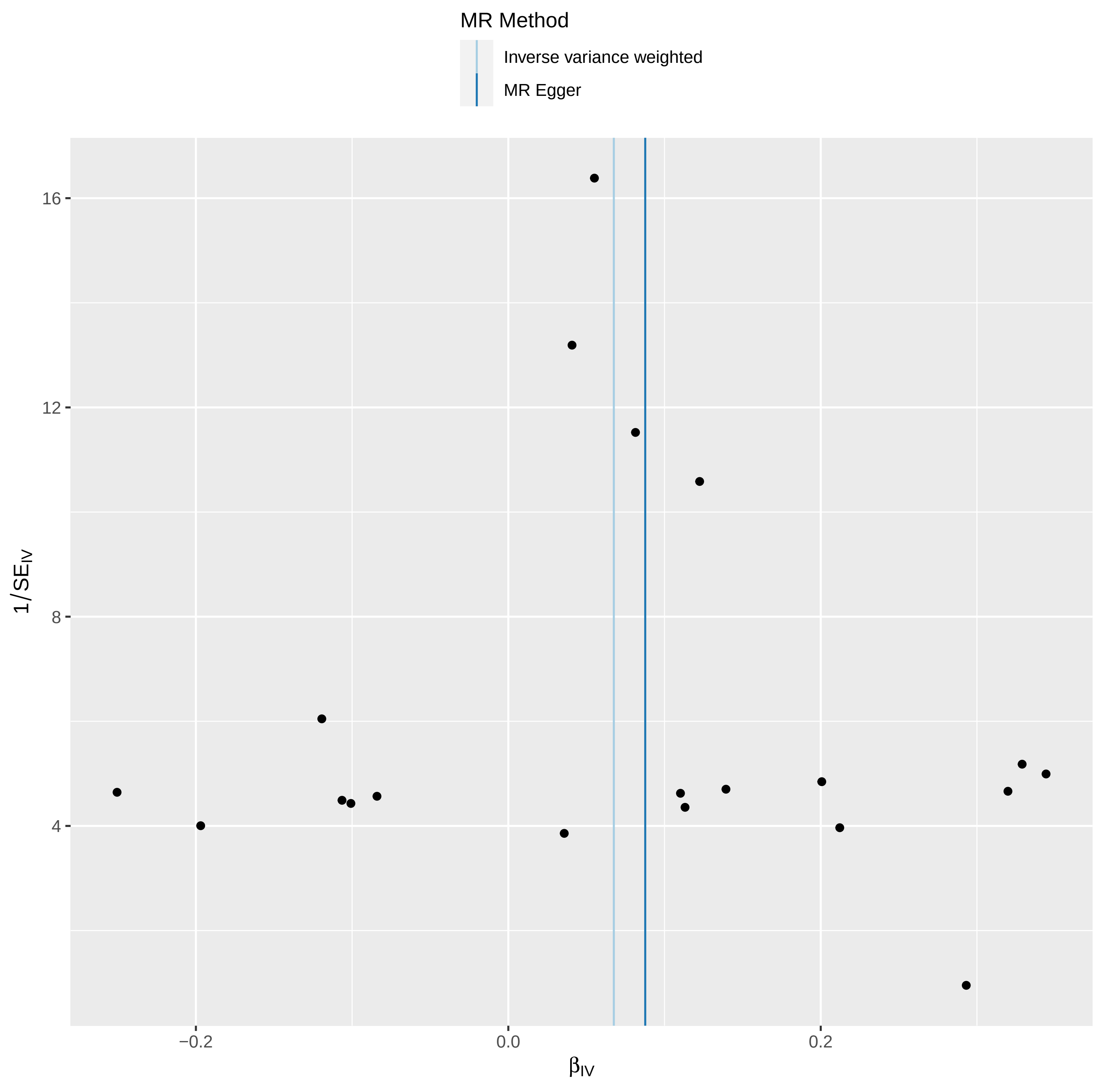

MR Method
Inverse variance weighted
MR Egger
16
12
8
4
1/SE$_{IV}$
−0.2
0.0
0.2
β$_{IV}$

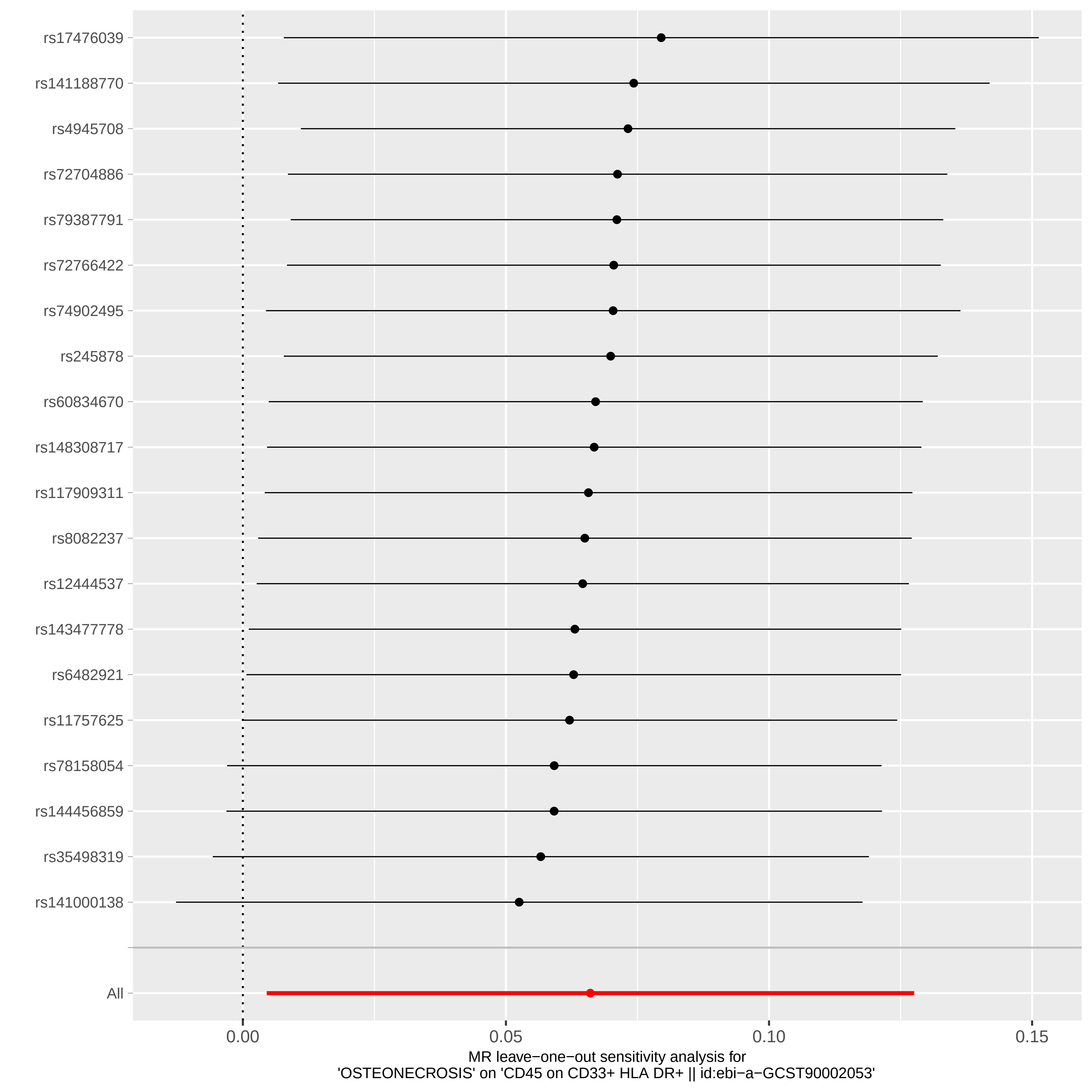
rs17476039
rs141188770
rs4945708
rs72704886
rs79387791
rs72766422
rs74902495
rs245878
rs60834670
rs148308717
rs117909311
rs8082237
rs12444537
rs143477778
rs6482921
rs11757625
rs78158054
rs144456859
rs35498319
rs141000138
All
0.00
0.05
0.10
0.15
MR leave−one−out sensitivity analysis for
'OSTEONECROSIS' on 'CD45 on CD33+ HLA DR+ || id:ebi−a−GCST90002053'

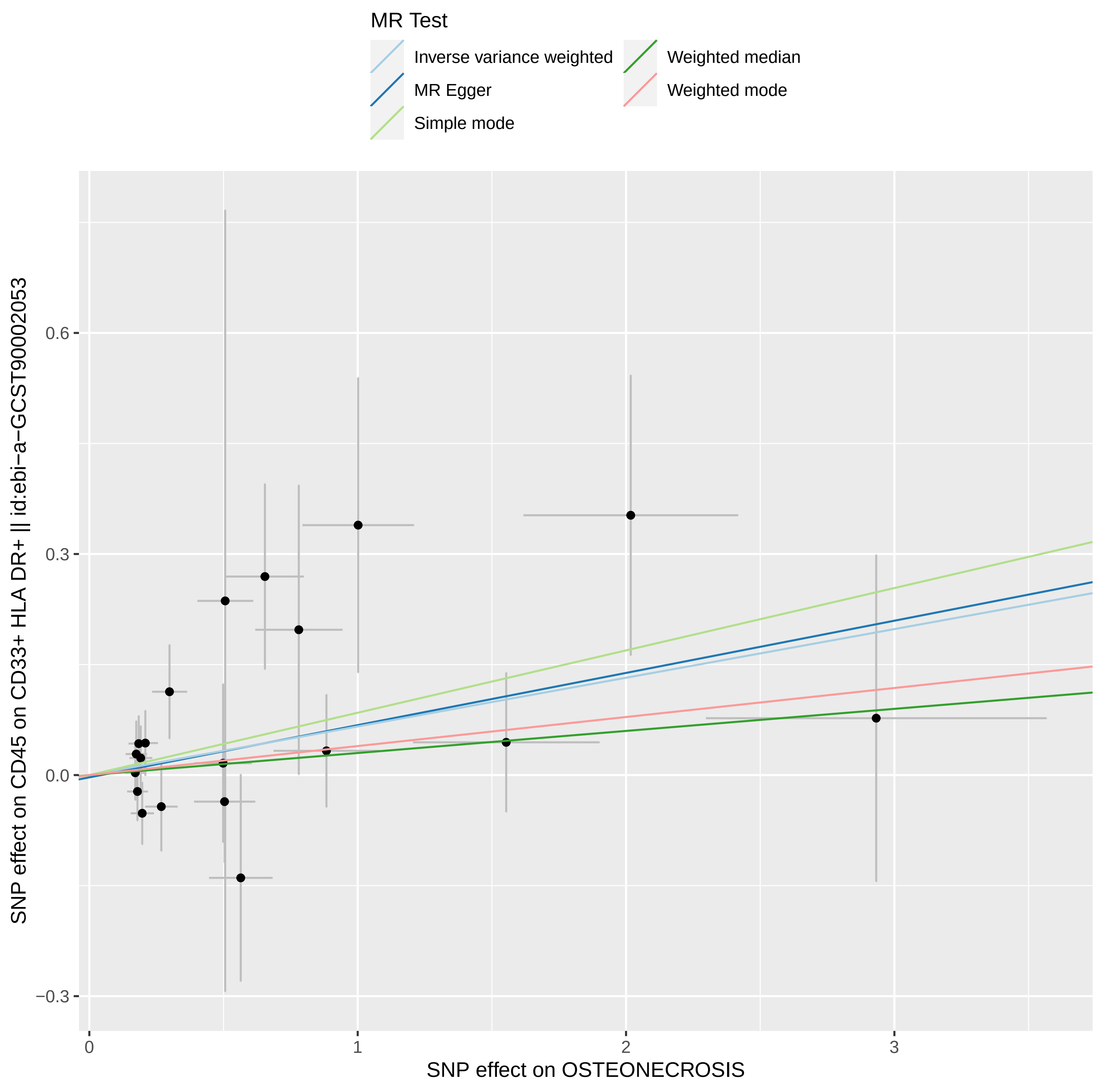
MR Test
Inverse variance weighted
MR Egger
Simple mode
Weighted median
Weighted mode
SNP effect on CD45 on CD33+ HLA DR+ || id:ebi−a−GCST90002053
0.6
0.3
0.0
−0.3
0
1
2
3
SNP effect on OSTEONECROSIS

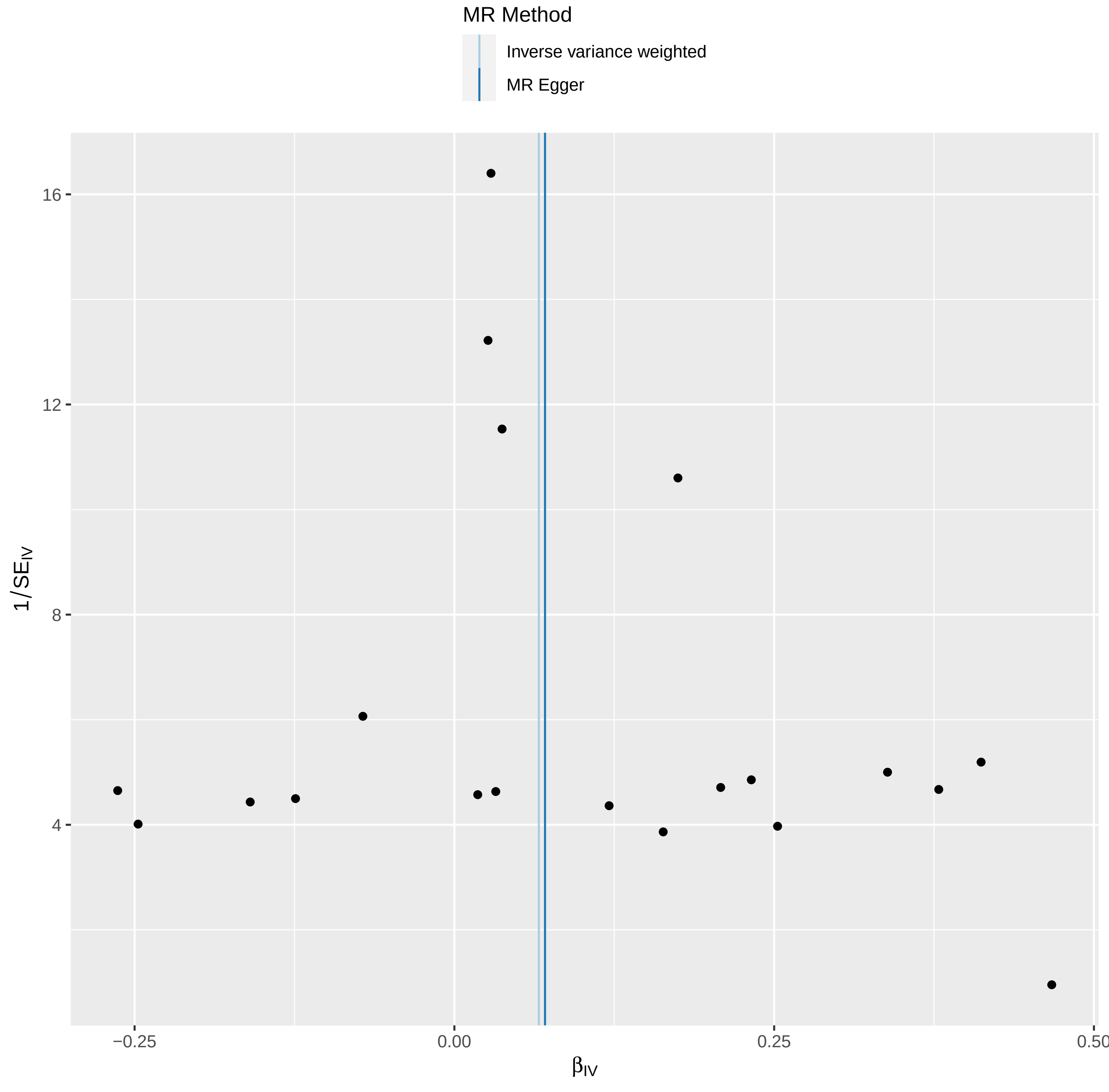
MR Method
Inverse variance weighted
MR Egger
16
12
8
4
1/SE_IV
−0.25
0.00
0.25
0.50
β_IV

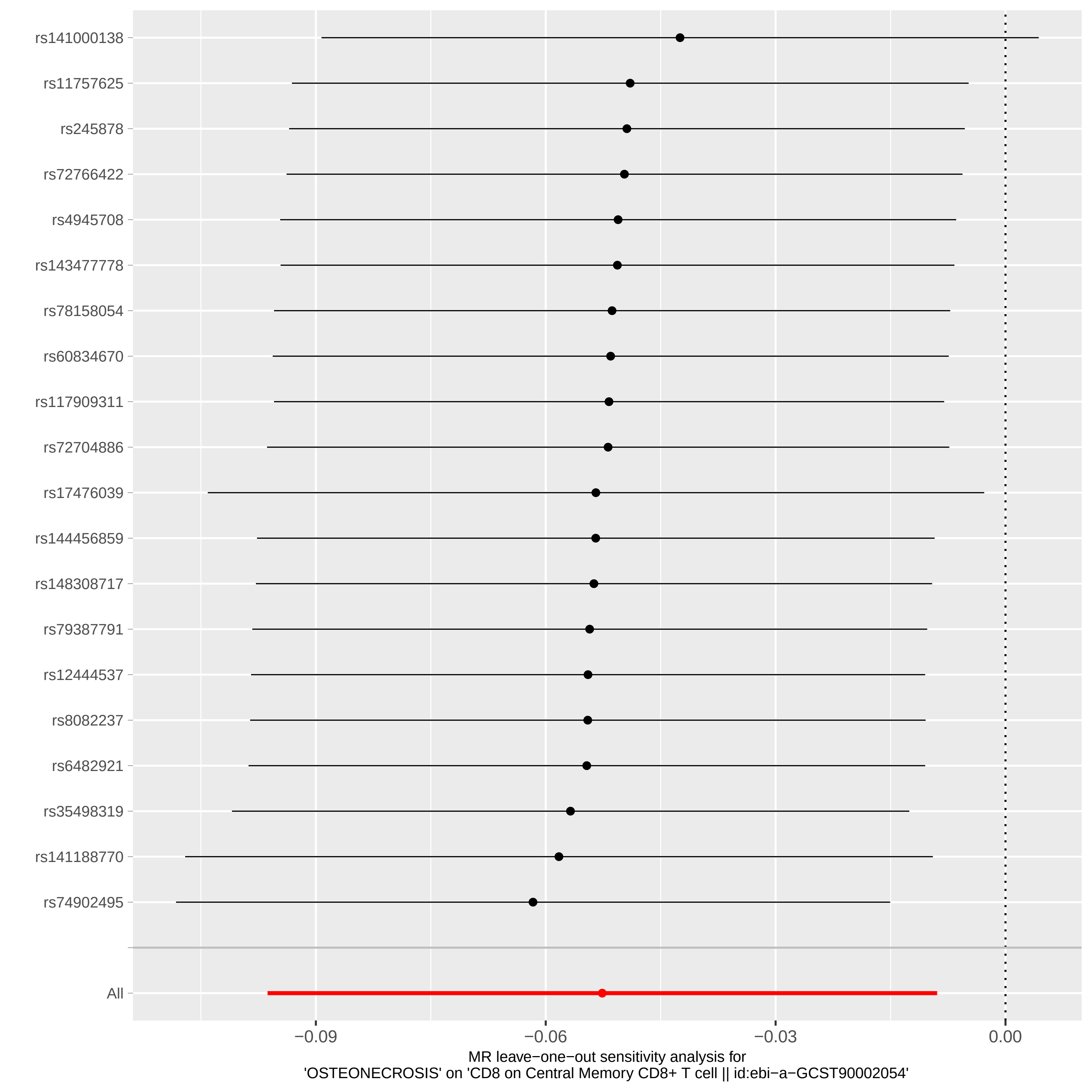
rs141000138
rs11757625
rs245878
rs72766422
rs4945708
rs143477778
rs78158054
rs60834670
rs117909311
rs72704886
rs17476039
rs144456859
rs148308717
rs79387791
rs12444537
rs8082237
rs6482921
rs35498319
rs141188770
rs74902495
All
−0.09
−0.06
−0.03
0.00
MR leave−one−out sensitivity analysis for
'OSTEONECROSIS' on 'CD8 on Central Memory CD8+ T cell || id:ebi−a−GCST90002054'

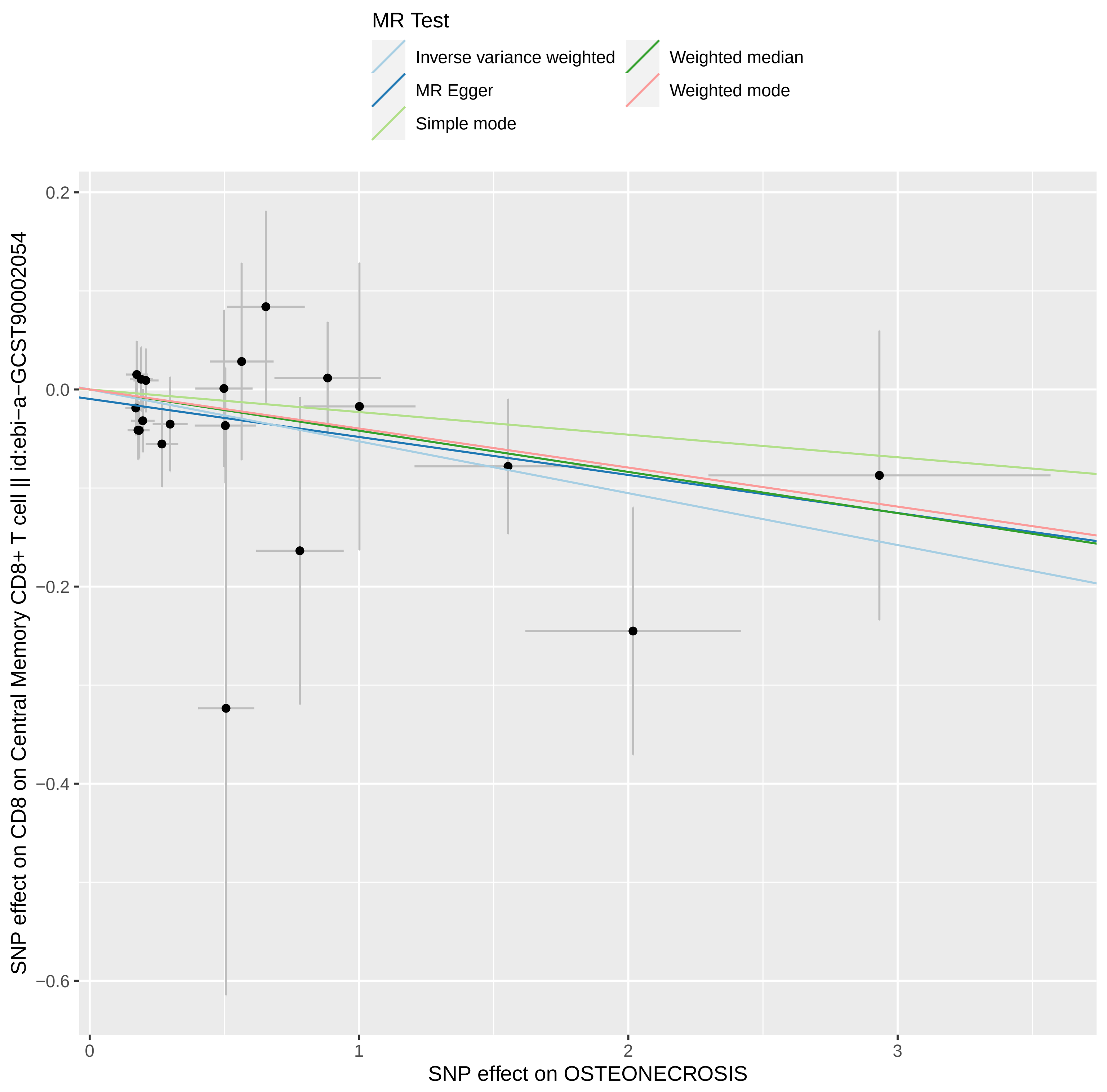
MR Test
Inverse variance weighted
MR Egger
Simple mode
Weighted median
Weighted mode
0.2
0.0
−0.2
−0.4
−0.6
0
1
2
3
SNP effect on CD8 on Central Memory CD8+ T cell || id:ebi−a−GCST90002054
SNP effect on OSTEONECROSIS

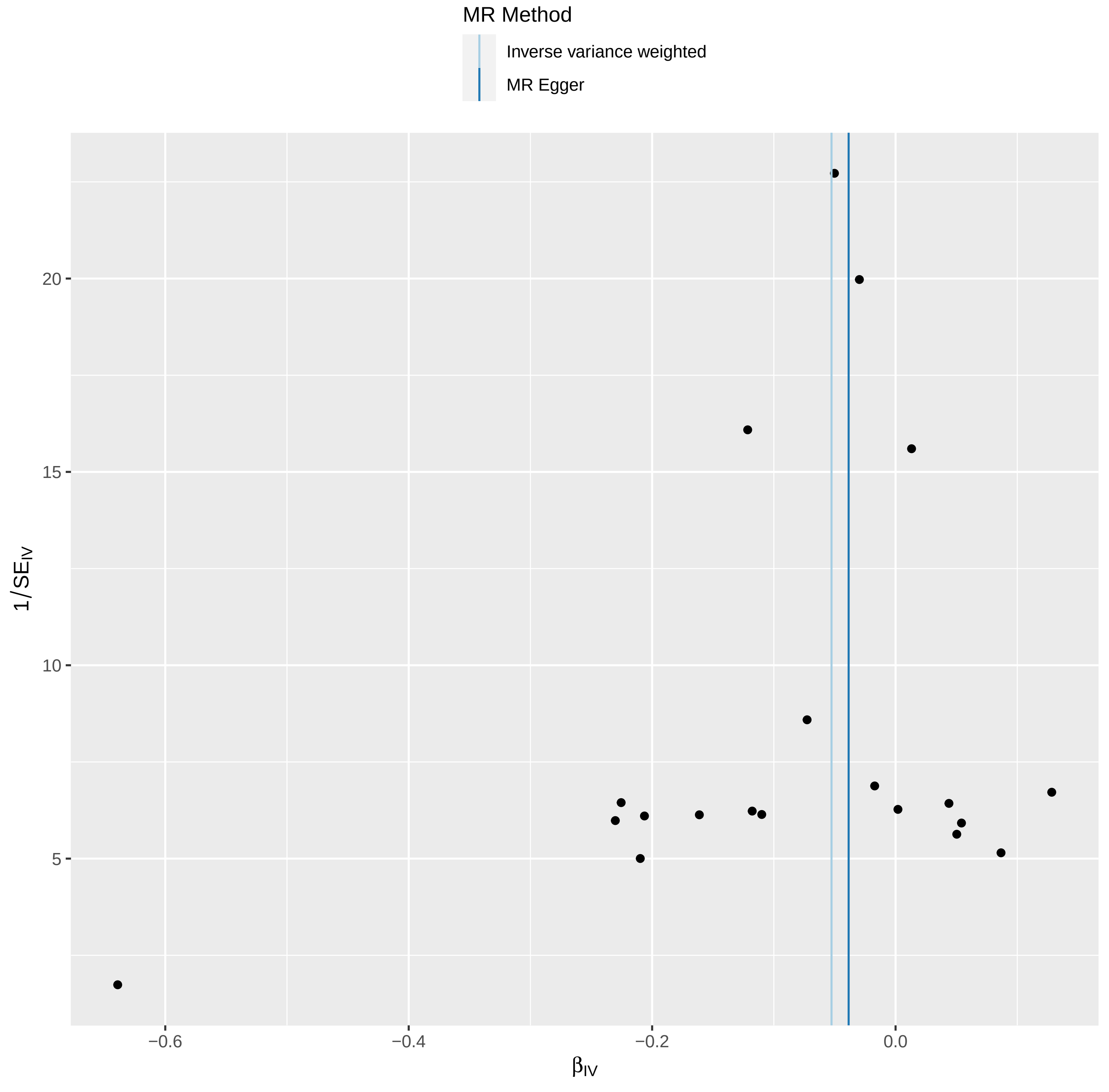
MR Method
Inverse variance weighted
MR Egger
1/SE$_{IV}$
20
15
10
5
−0.6
−0.4
−0.2
0.0
β$_{IV}$
